# Global Gene Expression Analysis of Murine Limb Development

**DOI:** 10.1371/journal.pone.0028358

**Published:** 2011-12-09

**Authors:** Leila Taher, Nicole M. Collette, Deepa Murugesh, Evan Maxwell, Ivan Ovcharenko, Gabriela G. Loots

**Affiliations:** 1 Biology and Biotechnology Division, Lawrence Livermore National Laboratory, Livermore, California, United States of America; 2 School of Natural Sciences, University of California Merced, Merced, California, United States of America; 3 Computational Biology Branch, National Center for Biotechnology Information, Bethesda, Maryland, United States of America; 4 Bioinformatics Program, Boston University, Boston, Massachusetts, United States of America; University of Bristol, United Kingdom

## Abstract

Detailed information about stage-specific changes in gene expression is crucial for understanding the gene regulatory networks underlying development and the various signal transduction pathways contributing to morphogenesis. Here we describe the global gene expression dynamics during early murine limb development, when cartilage, tendons, muscle, joints, vasculature and nerves are specified and the musculoskeletal system of limbs is established. We used whole-genome microarrays to identify genes with differential expression at 5 stages of limb development (E9.5 to 13.5), during fore- and hind-limb patterning. We found that the onset of limb formation is characterized by an up-regulation of transcription factors, which is followed by a massive activation of genes during E10.5 and E11.5 which levels off at later time points. Among the 3520 genes identified as significantly up-regulated in the limb, we find ∼30% to be novel, dramatically expanding the repertoire of candidate genes likely to function in the limb. Hierarchical and stage-specific clustering identified expression profiles that are likely to correlate with functional programs during limb development and further characterization of these transcripts will provide new insights into specific tissue patterning processes. Here, we provide for the first time a comprehensive analysis of developmentally regulated genes during murine limb development, and provide some novel insights into the expression dynamics governing limb morphogenesis.

## Introduction

The developing limb serves as an ideal model for studying differentiation of pluripotent mesenchymal cells into several distinct tissues, including cartilage, muscle, blood vessels, tendons and endochondral bone [1]. While the limb is a well established model system for studying genetic factors that regulate tissue patterning, differentiation and growth, the last decade of limb development research has been dominated by the same collection of widely studied genes, with little introduction of new candidate contributors. However, malformations affecting the limbs, more specifically the number of digits with which an infant is born, are among the most frequent congenital defects recorded in humans, occurring as often as 1 in 1000 live births. Currently, there are about 221 syndromes described with polydactyly and 120 syndromes with oligodactyly [2]. Despite this wide prevalence of limb abnormalities, to date only ∼84 genes have been associated with syndromes that include limb defects, 15 of which have described polydactyly [2]. Similarly, the sequential and tightly interconnected cellular events that lead to the establishment of each individual tissue type, as well as the three-dimensional molecular interplay within the vertebrate limb are not yet fully understood [3]. Finally, we have yet to grasp how the genome specifies fore- and hind-limb patterning to establish limb identity and ultimately result in the formation of complex homologous structures that are distinct in shape and function, such as the human hand and foot [4], [5].

The availability of DNA microarray technologies has provided the opportunity to comprehensively examine gene expression in serially homologous structures, such as the developing limbs. In this study, we took a genome-wide transcript analysis approach to obtain insights into the temporal and spatial pattern of gene expression during limb formation from embryonic day 9 (E9.5; shortly after limb bud initiates) to E13.5 when the cartilage template of future bone formation is established, to determine previously unidentified transcripts likely to contribute to limb patterning and limb identity, as well as to describe cohorts of genes that share the same stage-specific clustering, onset or peak expression profiles and are likely to contribute to shared developmental functions. Studies in *Drosophila* have determined that periods of rapid morphological changes during embryogenesis are associated with large-scale gene expression changes and have suggested that cataloging tissue specific gene expression at various stages of development would improve our understanding of factors involved in the development and function of each tissue [6], [7], therefore, our study aimed to gain novel insights into the molecular events underlying limb development.

Most of our current knowledge of vertebrate limb development has been acquired by observing the normal development of chicken embryos, *in ovo*; by ectopically administering molecules to the chicken limb and documenting the resulting morphological changes; or more recently, through the characterization of mutant mice with limb defects [8]. Forelimb buds start first, as small bilateral protrusions near the anterior end of the torso. About half a day later, when the forelimb is already visible as a tissue bulge, the hindlimb buds begin to protrude near the posterior end of the embryo. Each limb bud is composed of lateral plate mesoderm (LPM) covered by the surface ectoderm. The mesoderm contains the progenitors of skeletal elements, tendons, and other connective tissue of the fully-formed limb. The surface ectoderm gives rise to the skin and other cutaneous structures such as feathers on birds or hair on mammals. Shortly after the limb buds emerge, changes in cell shape and position within the surface ectoderm result in the appearance of a ridge, the apical ectodermal ridge (AER), which runs along the distal margin of each limb bud. Immediately after the AER has formed, a second control region emerges in the posterior region of the limb bud, the zone of polarizing activity (ZPA). The interplay between the AER, ZPA, ectoderm and the underlying mesenchyme control the elongation along the proximo-distal axis (shoulder to fingers), the flattening along the dorso-ventral axis (back of hand to palm), the number, and the identity of digits along the antero-posterior axis (thumb to little finger).

In an effort to learn more about the genes involved in limb morphogenesis, we have determined the global gene expression profiles in a time course from limb initiation, at embryonic day 9.5 to E13.5 when the limbs are fully patterned. This study identified 3520 genes up-regulated in the mouse limb that comprise 20 distinct expression profiles and determined that more than 1000 of these transcripts are novel, or have not yet been characterized in the limb, dramatically expanding the repertoire of candidate genes likely to function during limb pattering and development.

## Results

### Transcriptome Analysis Identifies 3520 Genes Up-regulated in the Developing Mouse Limb

**Figure 1:**
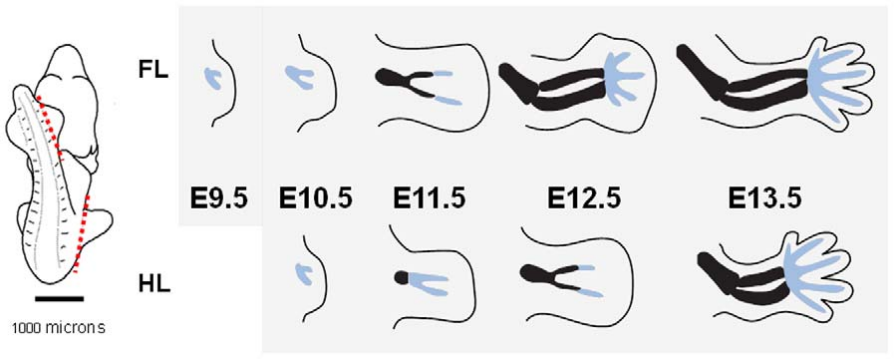
Limb Bud Morphology. Fore- and hind-limb buds were cut away from the embryo body as indicated by the red dash and RNA was processed from the full appendages outlined for stages E9.5 to E13.5. Light blue indicate mesenchymal condensations, and cartilage as determined by alcian blue staining is marked by thick black lines. Hindlimbs are morphologically delayed by ∼half a day.

To identify novel molecules responsible for limb patterning processes and determinants of limb identity, as well as to characterize the expression profiles of genes co-transcribed at high levels during limb development, we generated mouse whole-genome microarray gene expression data for fore- and hind-limbs from five consecutive developmental time points, from E9.5 to E13.5 (Figure 1), accompanied by data for whole embryos as controls. Fore- and hind-limb samples were compared to whole embryo samples at each time point to determine which transcripts were differentially expressed in the limb. Comprehensively, 6216 unique genes (31.4% of the mouse genome) were differentially expressed in at least one of the comparisons (with 3520 being significantly up-regulated), therefore, unless specified all percentages herein are based on this total count of differentially expressed genes (6216). We found E10.5–E11.5 to have the largest number of significantly up-regulated genes, with 30–36% of the transcripts up-regulated (1840–2263 genes). The least number of differentially expressed genes was found in the E9.5 fore-limb, where only 8% of the genes were up-regulated (Table 1), consistent with the very early stage.

**Table 1:** Differentially Expressed Genes.

*Differentially Expressed Genes from Limb vs. Whole Embryo Comparisons*													
	FL 9.5		HL 10.5	FL 10.5		HL 11.5	FL 11.5		HL 12.5	FL 12.5		HL 13.5	FL 13.5
Up-regulated	468 (8%)		1840 (30%)	1931 (31%)		2228 (36%)	2263 (36%)		1230 (20%)	1512 (24%)		1077 (17%)	953 (15%)
Down-regulated	347 (6%)		816 (13%)	796 (13%)		960 (15%)	868 (14%)		1194 (19%)	1189 (19%)		1799 (29%)	1765 (28%)

*Up-regulated Genes differeing between Successive Limb Stage Comparisons*													
	E9.5	*vs*	E10.5	E10.5	*vs*	E11.5	E11.5	*vs*	E12.5	E12.5	*vs*	E13.5	
Forelimb	212 (3%)		1675 (27%)	328 (5%)		660 (11%)	1066 (17%)		315 (5%)	816 (13%)		257 (4%)	
Hindlimb	n/a		n/a	283 (5%)		671 (11%)	1193 (19%)		195 (3%)	506 (8%)		353 (6%)	

*Stage-specific Up-regulated Genes*													
	E9.5		E10.5	E11.5		E12.5	E13.5						
Forelimb	186 (3%)		190 (3%)	405 (7%)		159 (3%)	200 (3%)						
Hindlimb	n/a		241 (4%)	473 (8%)		87 (1%)	259 (4%)						

A total of 6216 or 31.4% of gene symbols represented on the Affymetrix mouse array displayed expression changes in at least one pairwise comparison. All percentages displayed here are based on this total 6216 gene count. For both forelimbs (FL) and hindlimbs (HL) at each time point the number of up-regulated (>2X) and down-regulated (<2X) genes in relationship to whole embryo total RNA levels are indicated. Gene cohorts relevant to particular developmental stages were determined by comparing up-regulated genes between pairs of successive time points; genes exclusively up-regulated at each of the two time points considered (i.e., down-regulated or unchanged at the other) are indicated for each comparison (E9.5 vs E10.5; E10.5 vs E11.5; E11.5 vs E12.5 and E12.5 vs E13.5). Genes up-regulated only at one particular time point were identified by comparing the genes up-regulated at a given limb stage with those up-regulated at any other stage.

For the top 100 forelimb genes significantly up-regulated at each developmental time point we examined the known molecular function (GO category), description of available knock-out mouse phenotypes (http://www.informatics.jax.org/phenotypes.shtml) and the documented limb expression in whole mount mouse embryos (http://genome.ucsc.edu/cgi-bin/hgVisiGene). Most genes were represented in more than one developmental stage, corresponding to a total set of 279 non-redundant transcripts (Table S1). Thirty genes were up-regulated at all 5 time points, 126 were up-regulated at 4 time points (primarily E10.5–E13.5), 51 were up-regulated at 3 time points, 28 were up-regulated at 2 time points, and 44 transcripts were up-regulated at only one time point, either E9.5 (28 genes) or E13.5 (16 genes).

**Figure 2:**
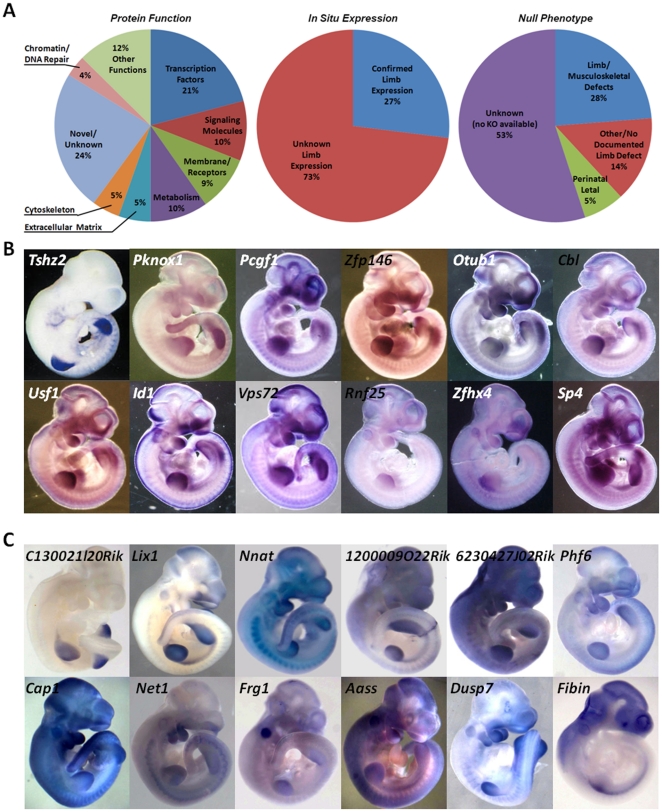
Analysis of the Top 100 Up-regulated Genes. 279 genes were examined for their putative protein function, available *in situ* expression in the limb, and description of null mouse phenotype (A). Most known genes previously uncharacterized in the limb with (B), or novel genes (C) showed unique and restrictive expression pattern in the limb. A survey of *in situs* of known genes with musculoskeletal defects in the knockout mouse are in Figure S1.

Among the 279 transcripts significantly up-regulated in at least one developmental stage, we found 21% to be transcription factors, 10% signaling molecules, 9% membrane-associated proteins (including receptors and channels) and 24% of them to be of unknown or novel function. Twenty-eight percent of these transcripts had confirmed musculoskeletal knockout phenotypes (79 genes; highlighted in brown in Table S1), and a similar percentage (27%; 76 genes) have been previously shown to be expressed in the limb by *in situ* hybridization (Figure 2A). These two sets of genes were highly overlapping (67%), and 23 genes with confirmed limb expression patterns corresponded either to previously uncharacterized genes, genes that resulted in no phenotypes (normal mice) when knocked out from the mouse genome, or the available knockout had no reported limb or musculoskeletal defect. Of the genes previously uncharacterized in the limb, 12 were perinatal lethal prior to E11.5 (highlighted in purple in Table S1), 14 had *in situ* expression patterns available and 137 or ∼50% were novel, lacking any prior knowledge of their putative limb function (Table S1; highlighted in grey). In contrast, the available knockout mice for 38 genes had ‘normal’ or no reported limb/musculoskeletal defects described, suggesting that these genes may be functionally redundant and the loss of their proteins is compensated by the presence of another, closely related transcript. Table S1 provides details for all the 279 transcripts. When analyzing the putative gene function off all 279 limb-enriched genes, the largest number of transcription factors (33%) and growth factors (15%) were found enriched at E9.5. In addition, 45 of the total 77 genes with reported limb or musculoskeletal defects in the documented knockout mouse strains were up-regulated at E9.5 suggesting that limb morphogenesis may be driven by very early signaling/transcriptional events that commence immediately after limb bud initiation.

### More than 100 Novel Genes are likely to Contribute to Limb Morphogenesis

**Figure 3:**
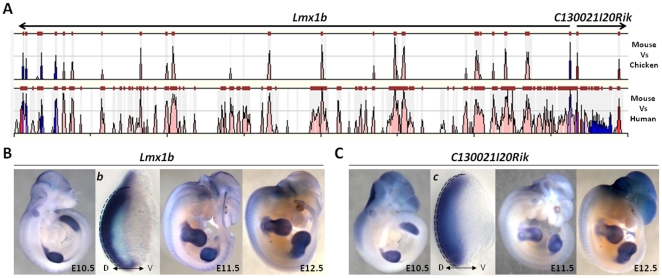
C130021I20Rik is a Novel Gene Co-transcribed from the Lmx1b Promoter, a well Known Limb Gene. *C130021I20Rik* was mapped to the *Lmx1b* gene locus (A) and found to be transcribed on the opposite strand. Time course *in situ* analysis revealed that *C130021I20Rik* (C) expression pattern is indistinguishable from its neighbor, *Lmx1b* (B), in the dorsal mesenchyme (b–c) suggesting that they are transcribed from the same divergent promoter and share limb-specific regulatory elements.

Among the 279 transcripts identified as the most highly expressed genes in the murine limb relative to the whole embryo, about half were novel (49% or 137 genes; Table S1; highlighted in grey). We defined novel genes as genes with no available knockout information and no published reports that would suggest the gene is essential to limb patterning or function. A few of the novel genes, primarily transcription factors, had known embryonic *in situs* patterns and those were noted in Table 2. We surveyed *in situ* expression patterns among 3 groups of transcripts: 1) genes known to function during limb development (Figure S1), 2) known genes previously uncharacterized in the limb (Figure 2B), and 3) novel genes (Table S1; highlighted in grey and Figure 2C), and found that while the majority of genes appeared to be expressed at E10.5 throughout the limb bud (without differentiating between superficial ectodermal staining and mesenchymal deep staining), a large number of genes had restricted and unique limb expression patterns. For example, among the novel genes, *C130021l20Rik* was expressed in dorsal ectoderm, *Nnat* and *Cap1* were primarily expressed in distal mesenchyme, *Fibin* was expressed at the base of the limb corresponding to cells migrating from the somites into the limb anlage, while the rest were expressed throughout the limb. The novel genes in Figure 2C are highlighted in grey in Table S1.

**Table 2:** Enrichment of functionally related genes in groups of co-expressed genes.

Cluster	Gene Count	Total # Genes	GO Category	Ontology	Description of GO Category	*p*-value	Enrichment
**cluster 1 (44 genes)**	4	47	GO:0048704	BP	embryonic skeletal system morphogenesis	0.0002	36.83
	19	682	GO:0003700	MF	sequence-specific DNA binding transcription factor activity	0.0000	12.06
	3	114	GO:0045892	BP	negative regulation of transcription, DNA-dependent	0.0259	11.39
	8	406	GO:0045944	BP	positive regulation of transcription from RNA polymerase II promoter	0.0001	8.53
	4	211	GO:0016564	MF	transcription repressor activity	0.0187	8.20
	18	1499	GO:0003677	MF	DNA binding	0.0000	5.20
**cluster 2 (51 genes)**	2	6	GO:0070700	MF	BMP receptor binding	0.0056	124.44
	2	6	GO:0060272	BP	embryonic skeletal joint morphogenesis	0.0056	124.44
	***4***	***33***	***GO:0001649***	***BP***	***osteoblast differentiation***	***0.0008***	***45.25***
	***3***	***28***	***GO:0030855***	***BP***	***epithelial cell differentiation***	***0.0033***	***40.00***
	4	42	GO:0030509	BP	BMP signaling pathway	0.0014	35.56
	4	55	GO:0030326	BP	embryonic limb morphogenesis	0.0022	27.15
	5	144	GO:0007507	BP	heart development	0.0029	12.96
	7	211	GO:0016564	MF	transcription repressor activity	0.0008	12.39
	6	406	GO:0045944	BP	positive regulation of transcription from RNA polymerase II promoter	0.0124	5.52
**cluster 5 (186 genes)**	4	33	GO:0048706	BP	embryonic skeletal system development	0.0425	12.54
	***5***	***47***	***GO:0048704***	***BP***	***embryonic skeletal system morphogenesis***	***0.0194***	***11.01***
	6	99	GO:0004888	MF	transmembrane receptor activity	0.0471	6.27
	***6***	***101***	***GO:0009952***	***BP***	***anterior/posterior pattern formation***	***0.0473***	***6.15***
	18	488	GO:0043565	MF	sequence-specific DNA binding	0.0008	3.82
	10	274	GO:0045941	BP	positive regulation of transcription	0.0425	3.78
**cluster 6 (475 genes)**	***4***	***8***	***GO:0048699***	***BP***	***generation of neurons***	***0.0186***	***20.52***
	***5***	***22***	***GO:0035108***	***BP***	***limb morphogenesis***	***0.0405***	***9.33***
	***7***	***38***	***GO:0042733***	***BP***	***embryonic digit morphogenesis***	***0.0150***	***7.56***
	7	45	GO:0060021	BP	palate development	0.0279	6.38
	11	103	GO:0003735	MF	structural constituent of ribosome	0.0150	4.38
	10	100	GO:0006414	BP	translational elongation	0.0351	4.10
	20	242	GO:0006397	BP	mRNA processing	0.0037	3.39
	17	236	GO:0008380	BP	RNA splicing	0.0186	2.96
**cluster 7 (73 genes)**	***3***	***18***	***GO:0001837***	***BP***	***epithelial to mesenchymal transition***	***0.0288***	***45.33***
**cluster 11 (405 genes)**	4	6	GO:0042273	BP	ribosomal large subunit biogenesis	0.0057	31.58
	29	100	GO:0006414	BP	translational elongation	0.0000	13.74
	21	103	GO:0003735	MF	structural constituent of ribosome	0.0000	9.66
	19	214	GO:0006412	BP	translation	0.0001	4.21
**cluster 12 (491 genes)**	5	17	GO:0070491	MF	repressing transcription factor binding	0.0373	11.43
	8	56	GO:0016566	MF	specific transcriptional repressor activity	0.0373	5.55
	21	261	GO:0000122	BP	negative regulation of transcription from RNA polymerase II promoter	0.0058	3.13
	16	209	GO:0016481	BP	negative regulation of transcription	0.0373	2.97
	16	211	GO:0016564	MF	transcription repressor activity	0.0373	2.95
	18	262	GO:0051301	BP	cell division	0.0437	2.67
	29	468	GO:0015031	BP	protein transport	0.0112	2.41
**cluster 14 (151 genes)**	***5***	***49***	***GO:0051216***	***BP***	***cartilage development***	***0.0224***	***12.95***

8/15 clusters were identified to contain cohorts of functional related genes (p<0.05; >2-fold enrichment) primarily represented by transcription factors (clustes 1/2/5/12) and genes associated with limb/skeletal morphogenesis (clusters 1/2/5/6). ‘Gene count’ represents the number of genes in the cluster with the specified biological process or molecular function, while ‘total # genes’ represents the total number of genes present on the microarray within the same category. Enrichment is fold over background. Genes corresponding to GO enriched categories highlighted in brown are plotted in Figure 5. BP biological process; MF molecular function.

In particular, the transcript corresponding to the novel gene *C130021I20Rik*, which was one of the most highly enriched transcript [classified among the top 100 genes at all developmental stages examined (E9.5 to E13.5)], was found to have a highly restricted expression pattern, primarily in the dorsal mesenchyme, highly similar to LIM homeodomain transcription factor 1 beta (*Lmx1b*) [9] (Figure 2C). Further examination of the genomic locus of this transcript, revealed that in the mouse, its transcription start site is physically located 682 base pairs upstream of *Lmx1B*, and is transcribed on the opposite strand (Figure 3A). Using nucleotide blast we compared the *Lmx1B* 1.2 kb mRNA (NM_010725) to the *C130021I20Rik* mRNA (AK147796) and found no significant sequence homology between the transcripts. We also did a detailed *in situ* analysis of these two genes from E10.5 to E12.5 (Figure 3B–C) and found them to have highly similar expression patterns in the limb. The close proximity of these genes, the lack of sequence homology shared by the transcripts and the divergent transcriptional direction of these two genes suggest that they are transcribed from a bidirectional promoter and are likely to share *cis*-regulatory elements that may be required for their shared limb-specific expression patterns. *LMX1B* mutations in humans cause an autosomal dominant inherited disease called nail-patella syndrome (NPS), which is characterized by abnormalities of the arms and legs as well as kidney disease and glaucoma [10], [11]. Expression of *Lmx1b* has been described as dorso-mesenchymal and this gene has been shown to be critical for specification of dorsal limb cell fates and consequently dorso-ventral patterning of limbs [12]. *C130021I20Rik* also appears to be restricted to the dorsal mesenchyme (Figure 3B), suggesting that this new transcript may functionally cooperate with *Lmx1b* to play a central role in fate determination and/or cell differentiation in the dorsal limb.

### Limits of Transcriptome Analysis: Missing Functional Genes with Low Limb Expression

To identify transcripts with elevated levels of expression in the limb we initially compared each limb sample to its corresponding whole embryo sample. In this comparison, genes that are highly expressed in the limb but at relatively low levels in all other parts of the embryo will emerge as ‘up-regulated’ in the limb. Even if a gene is expressed at high levels in other parts of the embryo, it will still be identified as ‘up-regulated’ in the limb as long as its overall RNA concentration (total RNA per sample volume) remains lower in the whole embryo as compared to the limb sample. In contrast, a gene that is ‘ubiquitously’ expressed will have similar RNA concentrations and hence will not be identified as ‘up-regulated in the limb. Thus, one potential limitation of this analysis stems from comparing the limb expression to the whole embryo expression; while powerful at extracting genes that are robustly expressed in the limb in relationship to other tissues, it falsely eliminates transcripts that are expressed in the limb at very low levels, or play important roles in non-limb tissues and are thus highly expressed in other parts of the embryo as well.

To estimate the rate of false negatives we examined how many genes known to cause limb defects are up-regulated in our comparisons. We extracted from the Mouse Genome Informatics Database (http://www.informatics.jax.org/) all the genes (674 genes) annotated with abnormal limb morphology (MP:0002109) as well as categories directly related to this one in the Phenotype Ontology hierarchy. Of these 674 genes, only 449 were represented on the array and 186 (41%) were significantly up-regulated in the limb in at least one comparison. Next we examined whether the genes that were not significantly up-regulated had different expression levels in the limb and in the whole embryo as compared with the genes up-regulated in the limb. We found that the genes that were not up-regulated in limb were expressed at significantly lower levels in the limb (P-value = 0.4×10^−13^, Wilcoxon rank sum test), but at approximately the same levels in the whole embryo. Furthermore, these genes were found to be expressed at lower levels in the limb as compared with all the genes on the array (P-value 2.9×10^−6^, Wilcoxon rank sum test), but not in the whole embryo. This analysis suggests that it is the particularly low level of expression of these genes in the limb rather than the differences in their average level of expression in the whole embryo that reduces our power to detect them in the context of this analysis (Figure S2).

We also examined 200 genes corresponding to the BMP, WNT, TGF-beta, hedgehog, FGF, and ROR signaling pathways, homeobox transcription factors along with other known signaling molecules and transcription factors linked to these pathways. We found 40% of these genes to be up-regulated in the limb in at least one comparison (82/200; Table S2). Twenty-nine percent (58) of these transcripts were down-regulated in the limb, compared to whole embryos, at least at one time point, and unchanged at all other time points, suggesting that a different part of the embryo had much greater expression of these transcripts than the expression observed in the limb. An additional 6 transcripts displayed a mixture of up-regulated and down-regulated incidences at specific time points, mostly in the *Hox* transcription factor category (4/6 genes). Most of the hedgehog signaling pathway genes were underrepresented among the up-regulated genes (*Gli2*; 1/7 was enriched in the limb), primarily because of their wide tissue distribution that masked their transcriptional enrichment in the limb.

Finally, we examined the expression values for genes transcribed at low, moderate and high levels in the limb. Genes on the arrays had (log2 transformed) expression values ranging from 2.4 to 14.6, with a median of 6.1. Using the average expression values for *Lrp5* and *Shh*, genes known to have important roles in the limb, but expressed at low levels (*Lrp5*; 4.2–5.6 expression value range) or in a highly restricted cluster of cells (*Shh*; 4.7–8.8 expression value range), we identified 19 genes with low expression values (<5) at all 5 developmental time points. This list included 4 genes known to have limb defects as heterozygous or homozygous null (*Tbx6*/*Tbx22*/*HoxB13*/*Lmx1a*). These analyses suggest that up to 60% of genes of limb genes expressed at very low levels may be missed by this method (Table S2; genes highlighted in yellow). However, additional biological validation will be needed to confirm the true rate of false negatives.

### Shared Expression Profiles Reveal Gene Specific Functions in Limb Development

**Figure 4:**
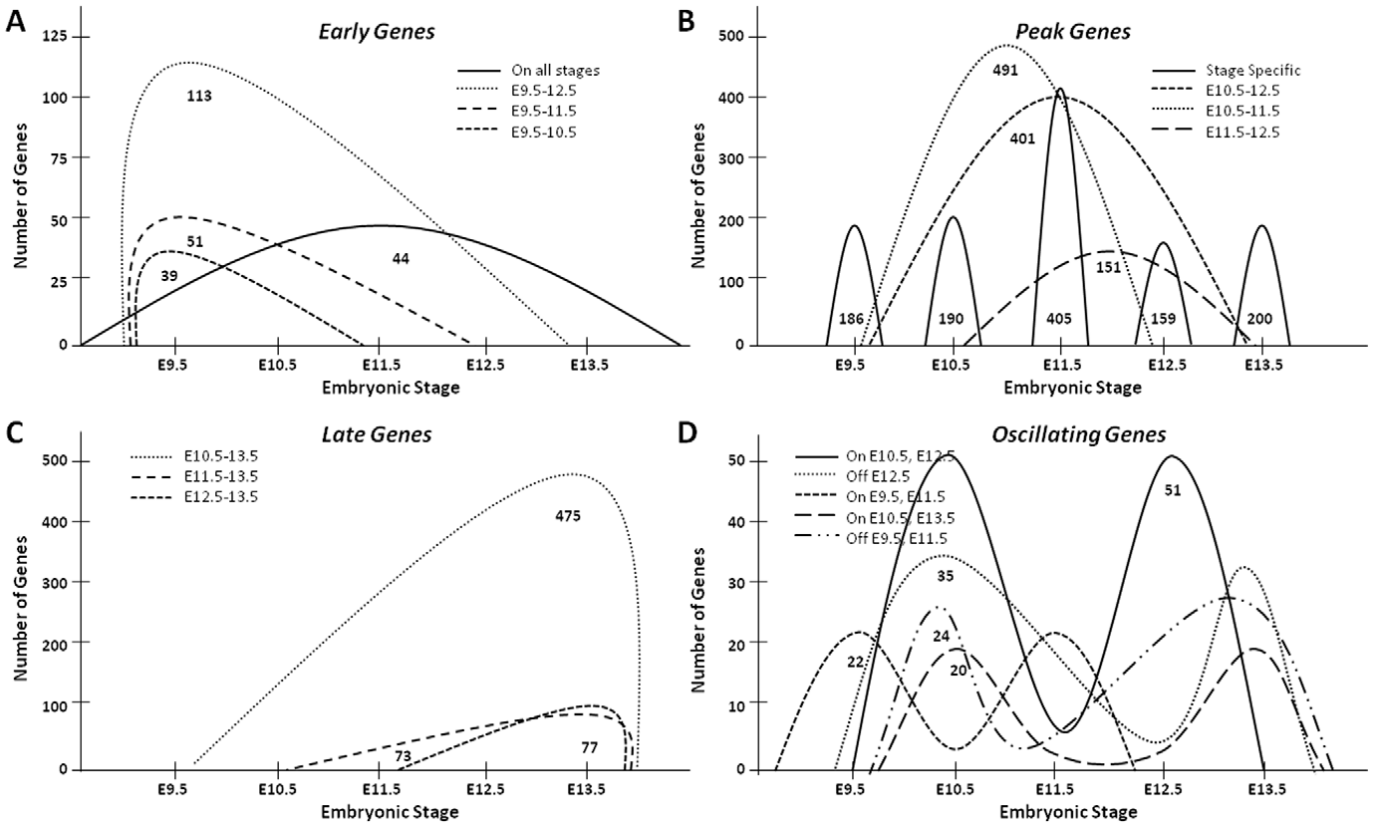
Temporal Expression Pattern Analysis in the Developing Forelimb. Clusters of co-transcribed genes were grouped into early genes (A), peak genes (B), late genes (C) and oscillating genes (D) based on their profiles. The number of genes corresponding to each curve are indicated below the trace.

Genes significantly up-regulated in the forelimb clustered into 31 groups according to their expression profiles. Twenty of these groups were comprised primarily of the up-regulated expression profiles of 3213 transcripts or 91% of the total genes found to be up-regulated in at least one pair-wise comparison with the corresponding whole embryo control. These 20 clusters were further grouped into four major categories: early genes, peak genes, late genes and oscillating genes (Figure 4A–D; genes corresponding to each cluster are listed in Table S3). The expression of 247 transcripts was elevated at E9.5 and at least one other time point, as part of clusters 1–4 comprising the ‘early gene’ category, where only 44 transcripts were enriched at all examined time points (Figure 4A).

A second category of genes, defined as ‘peak genes’, were distinctly up-regulated either at a single time point or within a narrow range of time points (Figure 4B). The largest cluster consisted of transcripts up-regulated at E10.5 and E11.5 (491). A smaller cluster encompassed genes enriched between E10.5 and E12.5 (401) and 151 transcripts were significantly up-regulated at E11.5 and E12.5. Genes that peaked at a single time point were classified as ‘stage-specific’ genes and are discussed in the next section. A third category of genes, termed ‘*late genes*’ turned on past E9.5 and their up-regulated expression persisted up to E13.5, and possibly beyond. A large number of transcripts (475) were found significantly up-regulated from E10.5 to E13.5, while smaller cohorts were enriched from E11.5 (73) or E12.5 (77) and beyond.

The last category of genes, ‘*oscillating genes*’ is represented by clusters of genes that display patterns of oscillating up-regulation in the limb, relative to whole embryo. A small number of genes (151) were distributed among these 5 clusters, with the smallest clusters representing genes that are up-regulated only at E9.5 and E11.5 (22), or at E10.5 and E13.5 (20). To determine if these genes truly have an oscillating behavior or whether their profiles are misleadingly derived because of fluctuating expression levels in the whole embryo we normalized the log2 expression value of each gene so that the mean and standard deviation across all the arrays are 0 and 1 respectively and plotted the limb and whole embryo expression in Figure S3. For most whole embryo samples we found the log2 expression to be negative, corresponding to low level of expression in the whole embryo. In most cases we found that, indeed, the limb expression oscillates, but the oscillating behavior was due either to variability in the limb expression only, or both in the limb and whole embryo (Figure S3). For example, in the case of genes that are “on” in the forelimb at E10.5 and E12.5 (51 genes), we actually have two effects at work: these genes are particularly highly expressed in the limb at these time points, but they are also slightly less expressed in the whole embryo as well, magnifying this effect. Similar observations were made for genes “on” in the fore-limb at E10.5 and E13.5: their expression was slightly decreased in the forelimb at E11.5 and E12.5, but also slightly increased in the whole embryo. Although the observed changes in expression are obviously not restricted to the limb, there is a limb-specific trend in their expression and this trend in the limbs is contrary to that in the whole embryo.

**Figure 5:**
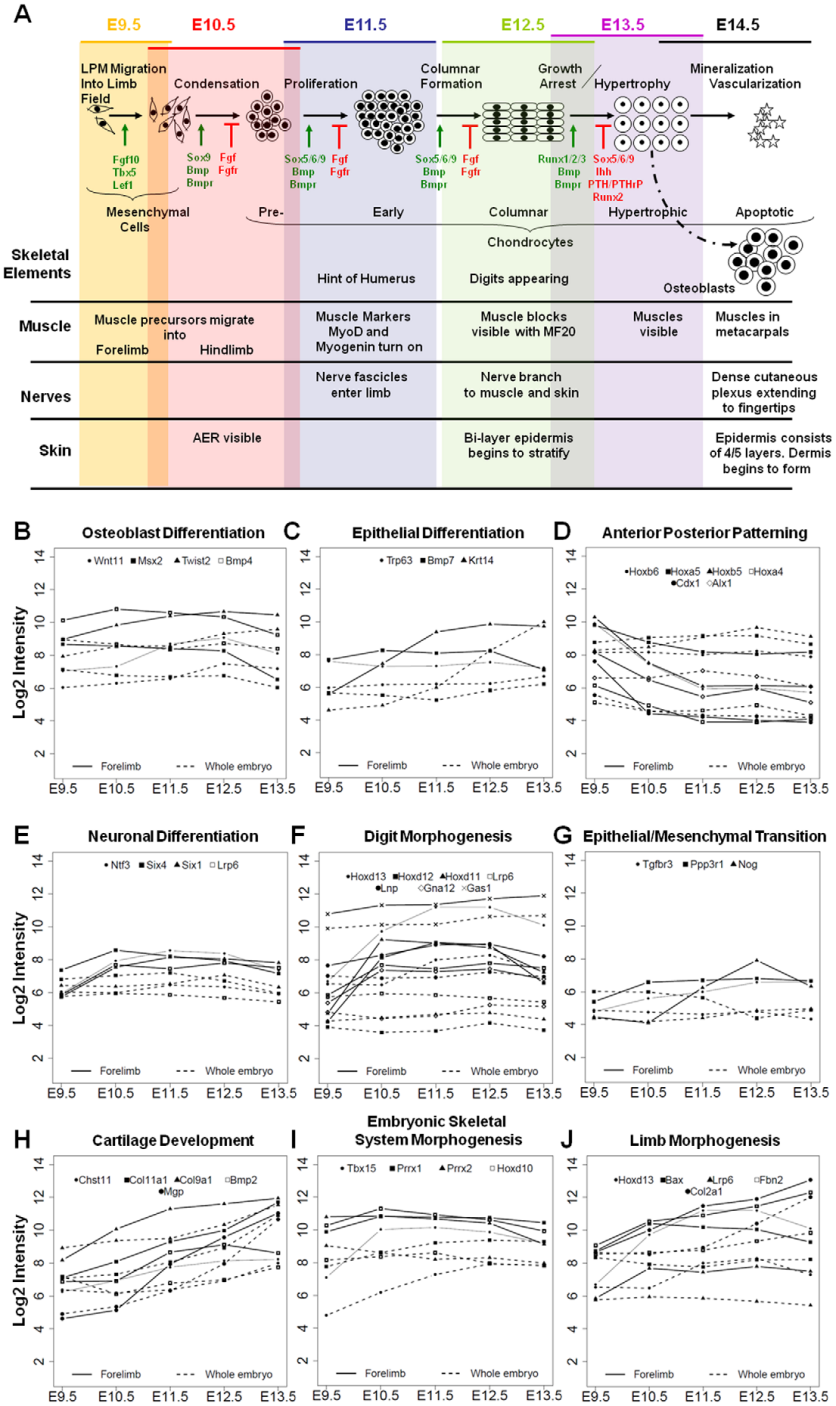
Differentiation Events Corresponding to Stages of Limb Development and Functional Category Enrichments that Describe some of these Events. Here we graphically depict the change in cell types and tissue formation as a function of developmental stage, highlighting key events during bone, muscle, nerve and skin formation (A). Some key molecules known to participate in these processes are also included along with the cell type morphologies for different stages of skeletal development. GO analysis identified several enrichment categories including osteoblast differentiation (B), epithelial differentiation (C), anterior-posterior patterning (D), neuronal differentiation (E), digit morphogenesis (F), epithelial to mesenchymal transition (G), cartilage development (H), embryonic skeletal system morphogenesis (I) and limb morphogenesis (J). For each plot log2 intensity is on the y axis, and developmental stages are on the x-axis. For each gene the solid trace depicts limb expression and the dashed trace depicts expression found in the whole embryo. Each gene corresponds to a different symbol along the traces.

One goal of using cluster analysis for such comprehensive expression datasets is to detect co-expressed genes, potentially revealing gene networks likely to regulate functionally distinct developmental processes such as chondrogenesis, osteoblastogenesis or myogenesis that progress concomitantly (Figure 5A). While the limb has long been an important model system for examining the molecular mechanisms of tissue patterning during development, its tissue heterogeneity, with many different cell types and signaling pathways intersecting to build complex multifunctional structures, poses a great challenge for the identification of functionally related genes. In Figure 5 we have outlined the events, developmental landmarks, and few molecular markers currently known for the formation of skeletal elements, muscle, nerves and skin between E9.5 and E14.5. To determine whether we can identify new gene candidates likely to contribute to one, or more of these pathways, we examined all clusters with more than 40 transcripts (15 clusters) for the presence of enriched functional categories (enrichment ≥2 fold with *p*-values adjusted for multiple testing ≤0.05) using the available Gene Ontology (GO) annotation for each transcript. ∼50% of the clusters examined (8/15) were found to be statistically enriched in genes that share a molecular function or a biological process (Table 2).

Clusters 1, 2, 5, and 6 were found to be enriched in transcripts involved in limb and skeletal morphogenesis, while clusters 1, 2, 5 and 12 were enriched in genes associated with transcriptional regulatory functions. The transcripts in cluster 1 (44 transcripts) were up-regulated in the limb at all examined time points; 45% of them had documented limb and/or skeletal defects (20/44) and 40% corresponded to transcription factors (18/44), a 12-fold enrichment for DNA binding transcription factor activity (Tables 2; S3).

We plotted the relative expression levels for the genes corresponding to osteoblast differentiation, epithelial differentiation, anterior-porterior patterning, neuronal differentiation, digit morphogenesis, epithelial-mesenchymal transition, cartilage development, embryonic skeletal morphogenesis, and limb morphogenesis enriched categories in Figure 5B–J and examined their expression dynamics vis-à-vis what is known about the morphological events during this developmental time course (Figure 5A). For most of these clusters we found a tight correlation between their expression pattern and the function they are known to participate in. For example anterior-posterior pattering is an early event, therefore most of the genes associated with this function had the highest expression level at E9.5, and continued to decline beyond this time point (Figure 5D). In contrast, digit morphogenesis occurs beyond E10.5, and most transcripts associated with this function were not expressed at E9.5, but were sharply up-regulated at E10.5 and beyond (Figure 5F). The most surprising observation was for genes associated with osteoblast differentiation (Figure 5B). Osteoblasts themselves are not ‘present’ in the limb until late E12.5, early E13.5, yet, these genes seemed to be consistently expressed at high levels during most time points, suggesting that these genes either participate in multiple independent events during limb morphogenesis, or that osteoblast specification and differentiation may initiate much earlier than anticipated and new cell-type specific markers may be needed to discriminate pre-osteoblasts from mature osteoblasts. While functional correlation of genes comprising these clusters can only be achieved through experimentation and validation, these new relationships can assist prioritization in future functional characterization of limb genes and conclusively link the genes with the biological process they mediate during musculoskeletal morphogenesis.

### Stage Specific Limb Development is Regulated by Redundant Mechanisms

With the aim of identifying the genes that determine limb patterning at particular developmental stages, we first established differences among cohorts of up-regulated genes between all pairs of successive time points. Overall, less than 27% of genes were differentially expressed between consecutive time points, with most changes ranging between 3–10% of the total 6216 differentially expressed transcripts were limb specific. We found the most dramatic up-regulation of gene expression to occur between E9.5 and E10.5 fore-limb comparisons, where ∼8 times more transcripts or 27% of total transcripts were more highly expressed at E10.5 forelimb than at E9.5. In the hindlimb, a similar transition was observed between E11.5 and E12.5 where the E11.5 hindlimb had 6 times more genes expressed than the E12.5 hindlimb. Since hindlimb development is delayed approximately by half a day in relationship to fore-limb development [13], we compared the overlap between E10.5 fore-limb specific genes (1675) and E11.5 hind-limb specific genes (1193). We found 607 transcripts or 50% of genes to be shared among these two cohorts, suggesting that E10.5 in forelimb and E11.5 in hindlimb may be proceeding through similar morphological processes and may represent key transitional time points during limb development, with the greatest number of up-regulated transcripts during the E9.5 to E13.5 developmental window.

**Figure 6:**
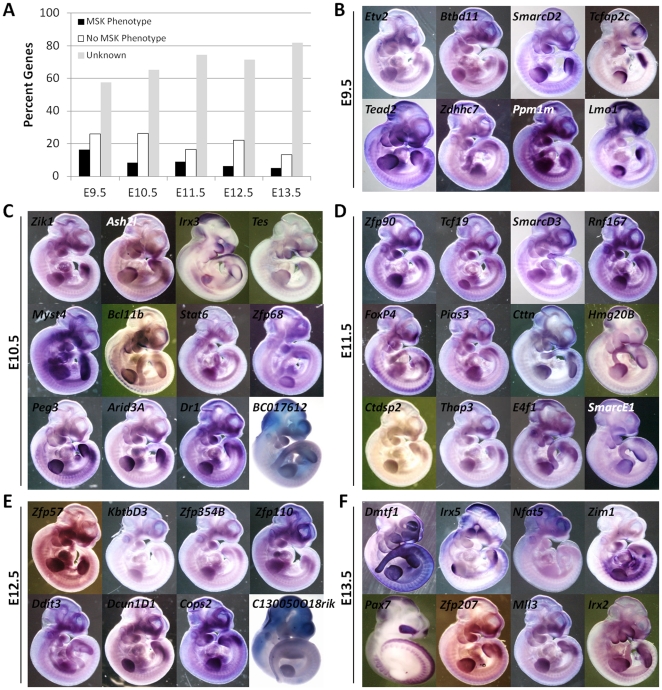
Analysis of Stage Specific Gene Expression. 1140 transcripts were found to be significantly up-regulated exclusively at one developmental time point. A large fraction of these stage specific genes were found to be novel (A), in particular at E13.5 only 5% of the transcripts had confirmed musculoskeletal knockout phenotypes and 6% of the genes have been validated by *in situ* hybridization. A survey of available *in situ* expression profiles for genes with peak expression levels at E9.5 (B), E10.5 (C), E11.5 (D), E12.5 (E) and E13.5 (F) revealed that they display a wide range of patterns, and include closely related members of gene families such as Irx, Smarc and zinc finger transcription factors.

Next, we turned to the analysis of strictly stage-specific genes, i.e., genes that are exclusively up-regulated at one time point (Figure 4B; stage specific genes). E11.5 had the largest number of *stage-specific* transcripts (405 or 7%), E12.5 has the least (159 or 3%), while all other time points had an average of 192 genes (3%). Twenty-three of these transcripts were also found among the top 100 overexpressed genes in the fore-limb, and are highlighted in Table S1 by 1 or 3 asterisks. We examined the known molecular function, description of available knock-out mouse phenotypes and the documented limb expression in whole mount mouse embryos for these 1121 genes (Table S4). E9.5 enriched transcripts had the largest number of confirmed musculoskeletal null phenotypes (30/184 or 16%), while E13.5 had the lowest (9/183 or 5%). The reverse relationship was observed for genes without any reported function in the limb, where 82% of the E13.5 enriched transcripts (150/183) had no available knockout, nor was the gene ever linked to any embryonic limb developmental function; in contrast 57% of the E9.5 enriched genes present in this category (106/184) (Figure 6A).

The *in situ* expression pattern survey of these 1121 stage specific genes revealed that with the exception of E9.5 and E10.5 genes where 18.5% and 11.5% were confirmed to be expressed in the limb, all other time points had less than 7% of genes with reported limb expression data. In Figure 6 we show the spatial expression pattern for representative stage specific genes from each time point (marked by an asterisk in Table S4). While a majority of these transcripts appear to be expressed throughout the limb bud (without differentiating between superficial ectodermal staining and mesenchymal deep staining), a large number of these genes also exhibit restricted and unique limb expression patterns. Interestingly, even transcripts where the available knockout mouse has not been reported to have a musculoskeletal defect show highly restricted limb expression patterns. Such genes include *Lmo1, Lmo3, Irx2* and *Irx5* transcription factors (Figure 6). While *Lmo1* and *Lmo3* knockouts have no obvious phenotypes, the *Lmo1/Lmo3* double knockout dies shortly after birth due to neural tube defects. In contrast *Lmo2* and *Lmo4* knockouts die embryonically before E10.5, suggesting that some or all of the 4 *Lmo* family members have redundant and overlapping functions, possibly including aspects of limb development [14].

Several other gene families were overrepresented among these stage specific enriched genes: 19 transcription factors belonged to the Kruppel associated box (KRAB-containing zinc finger) transcription factor family of transcriptional repressors (in descending order of expression: E9.5 enriched *Zfp771*, *Zfp775*; E10.5 enriched *Zfp763*, *Zfp418*, *Zfp68*; E11.5 enriched *Zfp90*, *Zfp783*, *Zfp13*, *Zfp637*; E12.5 enriched *Zfp57*, *Zfp354b*, *Zfp110*, *Zfp324*, *Zfp715*; and E13.5 enriched *Zfp760*, *Zfp826*, *Zfp280d*, *Zfp207*, *Zfp788*); 11 genes belonged to the *Tmem* family of transmembrane-like proteins (E9.5 enriched *Tmem59l*, *Tmem115*, *Tmem54*, *Tmem178*, *Tmem101*; E10.5 *Tmem177*, *Tmem2*, *Tmem120a* ; E11.5 *Tmem138*; E12.5 *Tmem208*, *Tmem11*); 4 genes are metallopeptidases as part of *Adam* and *Adamts* familes (E9.5 enriched *Adamts18* and *Adamts1*; E10.5 *Adam10*; E11.5 *Adamts7* and E13.5 *Adamts6*); and 3 genes belonged to the SWI/SNF related, matrix associated, actin dependent chromatin regulators or *Smarc* genes (E9.5 *SmarcD2*; E11.5 *SmarcD3* and *SmarcE1*).

Zinc finger proteins containing the KRAB motif represent the largest single family of transcription factors, estimated to make up ∼30% of all annotated zinc finger transcription factors in the human genome (290/799) [15], therefore it is no surprise that ∼2% of all the stage-specific enriched genes or 12% of the total transcription factors belong to this category. What is unexpected, however, is that only 2 of the 11 genes identified have been previously studied; *Zfp110* knockout has been described to be perinatal lethal by E12.5, and *Zfp826* has documented skeletal and craniofacial defects [16]. Among the 91 transcription factors in this category, 51 have available knockouts, 46 (90%) of which have been described to exhibit various musculoskeletal/limb defects or are perinatal lethal. The remaining 5 KOs are either normal or do not have any reported limb defects (Table S4). This high percentage of transcription factors with skeletal defects suggests that most of the other 40 novel transcription factors in this category are likely to participate in important events during limb morphogenesis, particularly with a likely role during the narrow developmental time point when their transcription is relatively up-regulated.

Disintegrin and metalloproteinases (ADAM) and ADAMs with thrombospondin motifs (ADAMTS) are two subfamilies of metalloproteinases closely related to the matrix metalloproteinases (MMPs). Some members of these subfamilies are associated with various physiological and pathological processes involved in embryonic development, angiogenesis, coagulation, and arthritis [17]. Nineteen distinct ADAMTS genes have been identified in humans, 4 (21%) of which are enriched in the limb. Their substrates include procollagen, hyalectans, decorin, fibromodulin and cartilage oligomeric matrix protein, hence based on their previously characterized roles these 4 *Adamts* genes enriched during limb development are likely to contribute to joint and cartilage formation. *Adamts1* knockout has a described adipose tissue defect, while the function of all the other 3 (*Adamts 6, 7* and *18*) have not yet been examined in knockout mice. ADAM10, the other metalloproteinase found to be up-regulated in the limb at E10.5, causes embryonic lethality by E9.5 due to failure of the cardiovascular and nervous system failures [18].

### Hindlimb Identity is likely achieved by Inhibition of Forelimb-Specific Genes

The analysis of differentially expressed genes is a powerful approach for elucidating the genetic mechanisms underlying the morphological and evolutionary diversity of serially homologous structures within the same organism (hand vs. foot) or among different species (hand vs. wing). Most genes known to be involved in limb patterning processes have been shown to confirm highly similar expression patterns in both the fore- and the hind-limb, however they dictate the formation of skeletal elements that results in distinctly unique structures such as hands and fingers in the forelimb and feet and toes in the hindlimb. Despite these dramatically different phenotypic skeletal patterning outputs, to date only a few genes have been determined to regulate limb-type identity. These include the forelimb-restricted *Tbx5* and the hindlimb-restricted *Tbx4* and *Pitx1* transcription factors [19], [20], [21], [22]. It has been hypothesized that additional key regulators of limb identity exist. This hypothesis is based on several observations: *Pitx1*, *Tbx4* and *Tbx5* are all transcription factors however their target genes are not known, nor are the upstream transcriptional regulators that confirm their limb specificity. In addition, gain- and loss-of-function mutations in these genes result in partial limb-type transformation phenotypes suggesting that additional key molecules participate in limb-type specificity.

**Figure 7:**
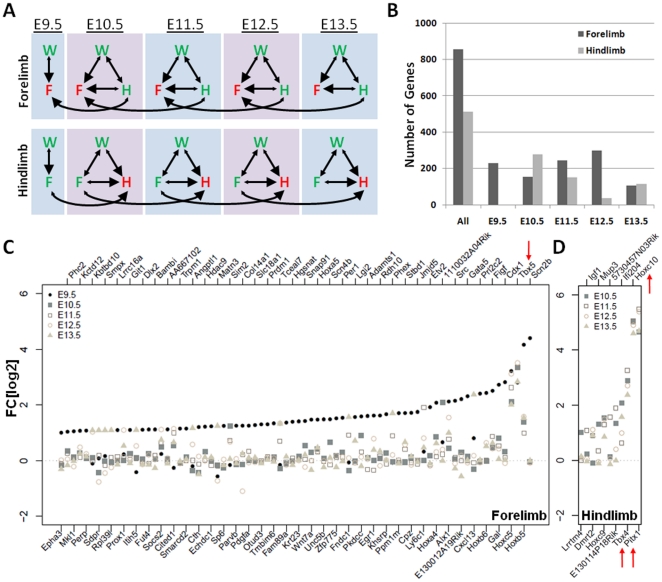
Identifying Genes that Contribute to Limb Identity. To identify genes specifically enriched in the forelimb or hindlimb, we adjusted for the developmental delay of hindlimbs by comparing forelimbs to same stage hindlimbs as well as to hindlimbs from a subsequent time point (A). More forelimb enriched genes were identified (B), and the genes with >2 fold up-regulation in the forelimb (C) or hindlimb (D) are displayed. Genes previously known to function as a limb-identity determinant are marked by red arrows (*Tbx5*; *Hoxc10*, *Tbx4*, *Pitx1*).

To identify new genes and formulate new hypotheses about initial patterning control and molecular pathways that dictate limb-specific identity we compared fore- and hind-limb expression at each time point (E10.5–E13.5, Figure 7A). Comprehensively, 855 transcripts were found to be up-regulated specifically in the fore- and 511 in the hind-limb, at least in one developmental time point. 230 transcripts were enriched in the forelimb at E9.5, 155 at E10.5, 245 at E11.5, 298 at E12.5 and 105 at E13.5. In the hindlimb, we observed 278 enriched transcripts at E10.5, 151 at E11.5, 36 at E12.5 and 114 at E13.5 (Figure 7B).

**Figure 8:**
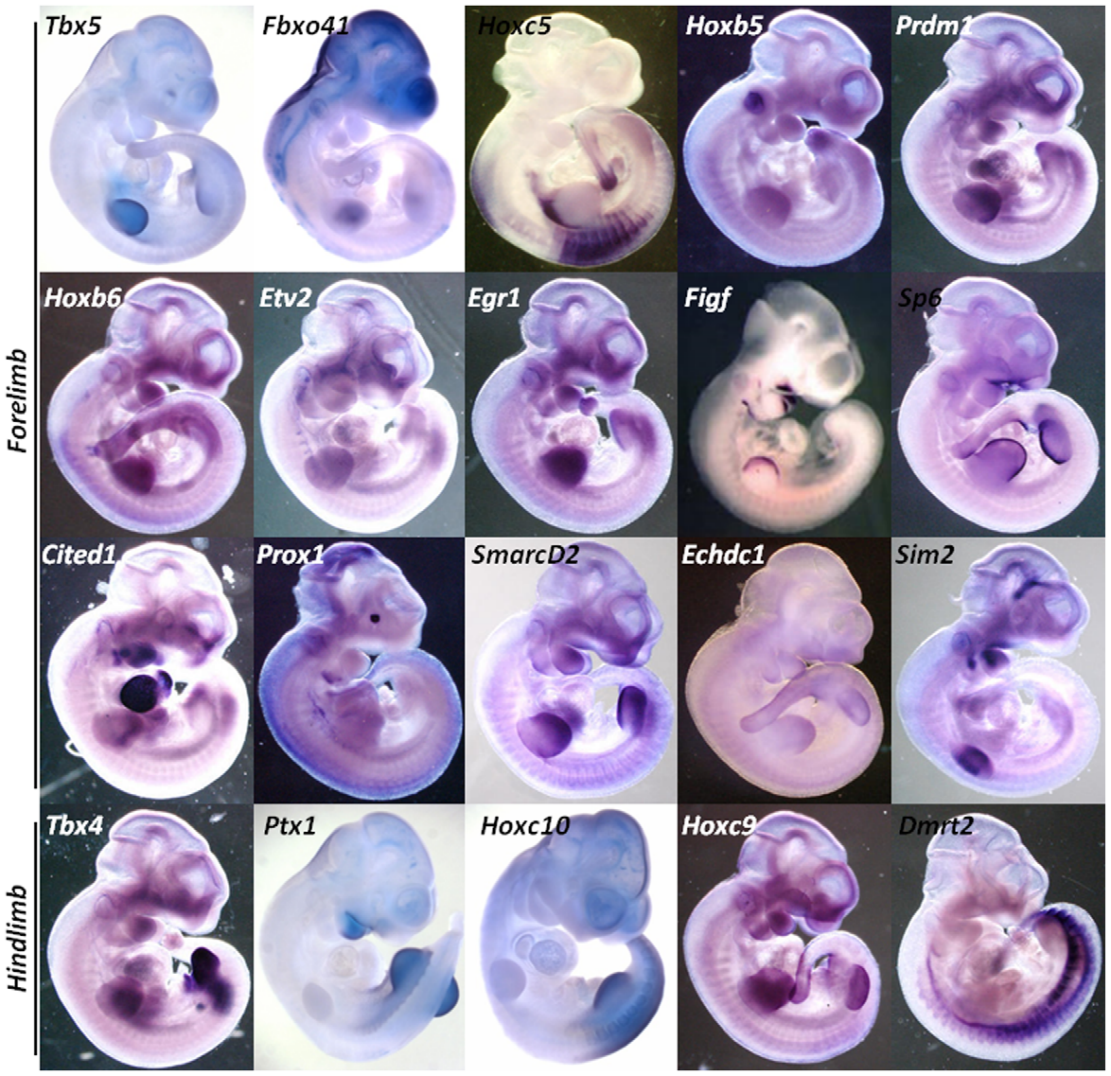
Expression Patterns of Fore- and Hind-limb Specific Genes. Qualitatively some genes identified as forelimb or hindlimb specific do not appear to be restricted to one limb such as *Sp6*, *SmarcD2* and *Hoxc10*, yet quantitatively these transcripts are >2 fold up-regulated in one limb in relationship to the other.

Figures 7C–D show genes up-regulated 2-fold or more in fore- (76) or hind-limb (11), relative to each other, in at least one time point. Furthermore, we examined the known molecular function, description of available knock-out mouse phenotypes and the documented limb expression pattern in whole mount mouse embryos for these genes and found 37% of them to have confirmed limb and skeletal phenotypes (fore- 29/76; hind-limb 6/11) in available knockout mice (Table S5). In addition, all genes that have been previously shown to be differentially expressed between fore- and hind-limbs, including *Pitx1*, *Tbx4*, *Tbx5*, and several *Hox* genes were also found to be differentially expressed at least at one sampled time point (Figure 7C–D; red arrows). The identification of these genes provided a positive control, and these differences were confirmed by whole mount *in situ* hybridization (Figure 8). Among the genes for which we could evaluate a protein function description based on GO classification confidence level and available experimental data, we found 25% (19/76) of fore- and 45% (5/11) of hind-limb genes to be known or putative transcription factors, suggesting that limb identity may be driven by a divergent transcriptional program, that uses different cohorts of transcription factors to instruct limb identity.

## Discussion

### Limb Morphogenesis is orchestrated by >35% of a Vertebrate Genome

This analysis provides a detailed description of the gene expression dynamics during murine limb development from limb bud initiation at E9.5 to a fully patterned autopod, at E13.5, and complements other efforts that attempt to study individual genes and pathways during limb morphogenesis through gene visualization (*in situs*) or targeted mutagenesis. Our analysis identified 3520 transcripts that are highly expressed in the limb and are likely to participate directly or indirectly in various aspects of limb patterning, growth and tissue function, dramatically increasing the repertoire of candidate genes that can be systematically studied in reverse genetic analysis to identify novel contributors to this intricate developmental process.

Since our analysis specifically focused on transcripts enriched in the limb >2-fold relative to whole embryo, we recognized that one potential limitation of our approach would be to falsely eliminate important limb genes that are expressed at very low levels in the limb, and do not meet these standards. To estimate the false negative rate, we used the average expression values to carry out a systematic survey of genes known to cause limb defects when mutated in knockout mice, yet are endogenously expressed at very levels in the limb. Our results estimate that up to 60% of these genes may be missed by this method suggesting that the list of genes in the limb may be much greater than the 3520 identified by the microarray experiment described herein. Nonetheless, using an estimate of 25000 total genes in the mouse genome, we can estimate that about ∼35% of a vertebrate genome participates in aspects related to limb patterning and morphogenesis.

### Identification of New Candidate Genes

In this manuscript, comprehensively, we have described ∼1500 genes that are highly expressed in the limb, and we provide detailed information about their known contribution to limb development including the molecular function, description of available knock-out mouse phenotypes and the documented limb expression in whole mount mouse embryos along with relevant citations. We find more than 30% of these genes to be novel or have not yet been examined in the context of limb development, and provide some new insights into expression dynamics and gene cohorts that may contribute to unique aspects of limb morphogenesis. Particularly we focused on describing a cohort of 137 novel transcripts which represent a useful resource of new limb candidates that can be further examined in the context of mutant mice. We also find a large number of transcription factors (E9.5: 27/184 or ∼15%; Table S4) to be specifically enriched during limb initiation, in contrast to all other time points examined (E10.5: 9.5%; E11.5: 5%; E12.5: 4.5%; 11%; Table S4), 30% of which are novel (9/27; Table S4, highlighted in grey). These genes are likely to be responsible for setting up cascades of gene activation or repression, therefore further analysis of these cohorts of genes will facilitate the dissection of gene regulatory networks underlying limb development through additional genomic and proteomic approaches. It also represents a starting point for interrogating parallel and overlapping gene networks that contribute to the specification, formation and growth of different tissues that comprise the developing limb and possibly be applicable to other organs in the mammalian body.

### Functional Redundancy during Limb Patterning

Similar to other published microarray reports, we too recognize that there is significant functional redundancy among paralogous and closely related genes in the limb. While one gene knockout may not reveal a limb or musculoskeletal phenotype, there is a high likelihood that closely related genes co-expressed in the limb may compensate for each other and be deemed as modifiers of limb development when analyzed in compound mutant allele configurations. This is the case for several transcription factors, such as *Lhx2*, *Lhx9* (the most highly up-regulated gene at E9.5), *Prrx1* and *Prrx2* which are all normal as single knockouts but *Lhx2/Lhx9* double knockouts display limb patterning and growth defects along AP and PD axis and *Prrx1/Prrx2* double knockouts exhibit postaxial polydactyly and bent zeugopods (Table S1; References S1). Similarly, genes for which the knockout phenotype is ‘normal’ or displays no limb/musculoskeletal abnormalities but the *in situ* expression reveals a remarkably strong and specialized pattern as in the case of *Wif1*, *Figf*, and *Pknox1* (to name a few from the 38 transcripts found to have no limb knockout phenotypes), there is a high likelihood that functionally related transcripts may be compensating for the loss of these genes.

### Genetic Pathways Responsible for Limb Identity

Recently it has been proposed that limb identity is achieved by default in the forelimb, whereas *Tbx4* propagates a unique transcriptional program in the hindlimb through the systematic repression of certain transcripts that may primarily function to drive forelimb morphogenesis [23]. Our results are consistent with this view since we observed ∼7.5-fold less genes up-regulated in the hindlimb and 45% of these hindlimb up-regulated genes are transcription factors (Table S5). Since it is generally recognized that the transcriptome can be used as a phenotypic character, and hence a potential source of defining limb identity, these findings suggest that these hindlimb up-regulated transcription factors are likely to play key roles in repressing the expression of forelimb specific genes in the hindlimb. *Hoxc10* was identified as the gene with the second highest expression level in the hindlimb, relative to forelimb, and its close relative *Hoxc9* was ranked 7^th^. Despite the fact that these two genes have displayed no hindlimb phenotypes in loss of function mice [24], [25], the dramatic up-regulation of these two genes in the hindlimb may suggest that these two homeobox transcription factors may have a yet not fully appreciated role in contributing to limb identity, in the mouse.

While it is still unclear what genetic components are required for specifying the identity of limbs to distinguish features unique to hands and feet, our data creates new opportunities to expand our current views, by comprehensively distinguishing genes that have restricted expression patterns, or are quantitatively distinct between fore- and hind-limbs. An alternative possibility to the *bauplan* hypothesis proposed where the forelimb represents the default genetic plan, and the hindlimb is primarily mediated by *Pitx1/Tbx4* is that limb identify represents the sum of both quantitative and qualitative shifts in expression patterns that ultimately give rise to bones that are different shapes and sizes, along with the accompanying muscle/vasculature/nerve architecture. While we only find 11 transcripts to be enriched more than >2 fold in the hindlimb we actually observe a much larger number of genes that are significantly more highly expressed in the hindlimb at E10.5 than in the forelimb (Figure 7B), but at less than a 2-fold threshold. Unfortunately, because the hindlimb has a slight developmental delay of almost half a day, it is very hard to determine what genes expressed in the E9.5 forelimb overlap with an equivalent developmental stage in the hindlimb, and we have yet to determine which of these transcripts are truly differentially expressed, or represent a slight developmental delay.

A recent report examining digit identity in chicken identified *Socs2* as a transcript highly expressed specifically in the third wing digit, but excluded in all other forelimb and hindlimb digits. This gene was also identified by our study as one of the most enriched forelimb specific gene, uniquely up-regulated in the E12.5 forelimb-hindlimb comparison, suggesting that this transcript may be involved in specifying forelimb digit identity, in mice and chickens [26]. The findings by Wang *et al* suggest that further functional characterization of the novel genes we found to be up-regulated in the forelimb are likely to emerge as contributors for morphological genetic programs. One other possibility that has not yet been explored and may account for morphological differences in limb and digit identity is gene repression *via* micro RNAs (miRNA). Recently, it has been shown that miRNAs can determine cell fate. In particular Yoo *et al* were able to change the fate of human fibroblasts into neurons through the overexpression of a particular set of miRNAs. It is therefore plausible that hindlimb identify may be driven by a miRNA down-regulation of forelimb specific genes. While this is still a hypothesis, future characterization of miRNA profiles during limb development will be able to confirm if regulatory RNAs play a significant role in limb morphogenesis.

### Candidate Genes for Limb Defects in Humans

Our work was primarily motivated by the disconnect between the number of syndromes accompanied by hand and digit malformation which exceeds the number of confirmed genes associated with these syndromes by more than 4 fold (84 genes confirmed to cause congenital limb defects/341 syndromes with hand/digit defects). The work herein offers the opportunity to shed light on the genetic basis for some of these well documented limb defects that sometimes occur with significant frequency. With the advancement of highly quantitative platforms for gene expression analysis that gives raw counts of mRNA, one could envision the generation of a limb-specific array representing a large percentage of transcripts enriched in the limb that can be tested on limb derived RNA from ENU derived mutant mice that display limb defects. Such a platform would expedite mapping of candidate genes to their physical location in the mouse genome. Alternatively, one can envision a genotyping platform that allows patients with congenital hand/foot defect to be tested for candidate mutations in these genes. In general finding transcriptional differences between healthy and diseased or malformed organs will ultimately represent a powerful approach to linking genotypes to phenotypes.

## Materials and Methods

### Animals

**A**pprovals for work conducted on the mice used in this study were granted by Lawrence Livermore National Laboratory Institutional Animal Care and Use Committee, under application no. 168. Animals were treated humanely; housing and experimental procedures followed the guidelines outlined in the National Institute of Health ‘Principles of Laboratory Care’.

### Tissue Collection and RNA Isolation

Wildtype mice (FVB) were used as the tissue source for the microarray analysis. Embryonic day 0.5 (E0.5) was established at noon the day a plug was identified. Pregnant females were euthanized by CO_2_ followed by cervical dislocation, and harvested embryos were dissected under a dissecting scope in ice cold RNase-free phosphate buffered saline (PBS). Embryos were staged according to Wanek at al. (1989) [27]. For each sample fore- or hind-limb buds were from one embryo only, from a total of 5 embryos. All analyzed embryos are experimental replicates and originate from one mouse plug. Total RNA was isolated using Qiagen Rnasy, per manufacturer's suggestions.

### QC and Microarray Hybridization

Microarray expression analysis was performed using Affymetrix® Mouse GeneChips 430 2 (430v2). Hybridization of biotin-labeled cRNA fragment to Mouse Genome 430 2.0 array, washing, staining with streptavidin-phycoerythrin (Molecular Probes), and signal-amplification were performed according to the manufacturer's instructions.

### Microarray Preprocessing and Identification of Differentially Expressed Genes

Fifty-two array data sets were analyzed to search for genes with expression levels significantly altered between different time points and between fore- and hind-limb. For each time point, up to 5 biological replicates were obtained from the same litter for each limb, and 2 from whole embryos. Each sample represents the total RNA collected for fore-, hind-limbs, and whole-embryo homogenates from single embryos, for up to 12 arrays per time point. Mouse Genome 430 2.0 arrays contain 45,101 probe sets associated with ∼20,000 genes. Probe sets were mapped to genes using information provided by the Jackson Laboratory (http://www.informatics.jax.org/). Compared to the whole embryo controls, we identified 6216 differentially expressed genes. All data is MIAME compliant and the raw data has been deposited in a MIAME compliant database (GSE30138).

All analyses were performed using R [28], BioConductor [29], and several customized Perl scripts. R packages “affy” [30] and “simpleaffy” [31] and “affyQCReport” [32] were employed to evaluate the quality of the arrays by means of images, histograms, box plots, degradation plots, and scatter plots. CEL files were scrutinized for spatial artifacts using Harshlight package (PMC1274260). Using the median value of a group of replicates as the reference value, we corrected a small blemish affecting the readings for one of the whole-embryo control samples at E12.5. Expression values were derived using the Robust Multichip Average (RMA) protocol [33] with default settings. All analyses were done at the so-called sequence level, i.e., data from probes representing the same gene were combined. We did not apply any unspecific filter on the expression values. Differentially expressed genes were identified using an empirical Bayes method implemented in the R package “Limma” [34]. P-values were corrected for multiple testing using a 5% false discovery rate [35]. The expression fold change (FC) of a gene was calculated as the ratio between its average normalized expression value in a given limb sample and in the time-matched control. Therefore, a fold change of 1 (log2 FC = 0) indicates no change, while a fold change of 2 (log2 FC = 1) equals a doubling in transcript abundance, and a fold change of 0.5 represents a reduction by half (log2 FC = −1).Genes with expression fold-changes of 2 or greater with adjusted p-value<0.001 were considered differentially expressed.

### Top Up-regulated Forelimb Genes

Genes that were differentially expressed (i.e., displayed a fold-change of 2 or greater, with an adjusted p-value<0.001) between fore-limb samples and time-matched controls were sorted by decreasing fold change values.

### Clustering Analysis

We sorted the 3215 genes up-regulated with respect to controls at least at one fore-limb developmental stage based on expression fold change similarity. For this purpose, at each developmental stage, genes were classified into either up-regulated or down-regulated/unchanged, and separated into the 31 non-overlapping clusters representing all possible combinations of expression patterns at 5 different forelimb developmental stages. For instance, cluster 1 (Figure 4A) comprises genes up-regulated in forelimb at time 9.5, and down-regulated/unchanged at all other considered time points.

### Functional analysis

Functional analysis was based on over-representation of GO terms (PMCID: PMC3013640). Statistical enrichment of GO terms was quantified by using the hypergeometric distribution statistics. We report categories with p-values adjusted for multiple testing using Benjamini and Hochberg's method [35] that are smaller than 0.05.

### Limb identity

Since hindlimb development is delayed by an approximate half a day, to account for transcriptional changed due to this forelimb/hindlimb developmental discrepancy, we defined forelimb specific genes as genes that are up-regulated in forelimbs with respect to time matched controls (i.e., displayed a fold-change of 2 or greater, with an adjusted p-value<0.001) at a particular developmental stage, and not up-regulated in hind-limbs within the same stage, as well as in the subsequent developmental stage. Similarly, we defined hind-limb specific genes as genes that are up-regulated in hindlimb at a given developmental stage with respect to time matched controls, and not up-regulated in forelimbs within the same stage, as well as in the preceding developmental stage.

### Whole Mount *In situ* Hybridization (WISH)

WISH was performed as previously described [36] with some modifications. Briefly, embryos were isolated from timed pregnancies of C57BL6 mice (Jackson Labs) and fixed overnight at 4°C in 4%PFA in PBS and stored in 100% Methanol at −20°C until ready to use. Embryos were rehydrated gradually into PBT, and endogenous peroxidase quenched with 6% H_2_O_2_ in PBT for one hour. Hybridization was performed with digoxigenin labeled antisense riboprobes for 16 hrs at 70°C. Post-incubation the embryos were washed in 50% formamide, 4X SSC, 1% SDS and 50% Formamide, 2XSSC for one hour each at 65°C. After blocking embryos (2% Roche Blocking agent in 1XMAB) for 1 hr, embryos were incubated overnight at 4°C in 1∶3000 anti-digoxigenin AP Fab fragment antibody. Color was developed with BM Purple. For probe info see *Material and Methods S1*.

## Supporting Information

Figure S1 **Known Genes Identified among the Top 100 Up-regulated Genes.** 279 genes were examined for their putative protein function, available *in situ* expression in the limb, and description of null mouse phenotype. Here are some *in situ* expression patterns for the genes known to function during limb development. *LacZ expression pattern of the knock-in allele.
(TIF)

Figure S2 **Whole Embryo and Limb Average Expression Values.** Whole embryo expression values are independent of whether a gene is up-regulated or not in the limb (P-value = 0.4×10−13, Wilcoxon rank sum test). Solid lines represent expression distribution for limb and dashed lines for whole embryo [all (green); up-regulated in limb (black); not up-regulated (red)].
(TIF)

Figure S3 **Whole Embryo and Limb Normalized Log2 Expression Values for Oscillating Genes.** The log2 expression values were normalized for each gene such that the mean and standard deviation across all arrays are 0 and 1, respectively. The height of the bar represents the average of this normalized expression value for each sample. Whole embryo expression is visualized by black bars and limb expression by red bars. Each cluster (A) On E10.5, E12.5; (B) On E10.5, E13.5; (C) On E9.5, E11.5; (D) Off E9.5, E11.5; (E) Off E12.5, has whole embryo values below 0 (except for E13.5 in panel C), suggesting that whole embryo expression is very low relative to limb expression which is dominantly above 0.
(TIF)

Table S1 **Top 100 upregulated genes.**
(PDF)

Table S2 **Expression values for genes known to function during limb development, or family members of signal transduction pathways known to contribute to limb patterning.**
(PDF)

Table S3 **Clusters of Differential Gene Expression.**
(PDF)

Table S4 **Stage-specific enriched genes.**
(PDF)

Table S5 **Forelimb and Hindlimb up-regulated transcripts.**
(XLSX)

Material and Methods S1 **In Situ Probe Information.**
(PDF)

References S1 **Supplemental references.**
(PDF)
