# Supplementary figures and images for: Global Gene Expression Analysis of Murine Limb Development
Source: PLoS One. 2011 Dec 9;6(12):e28358. doi: 10.1371/journal.pone.0028358 (PMC3235105; doi:10.1371/journal.pone.0028358)

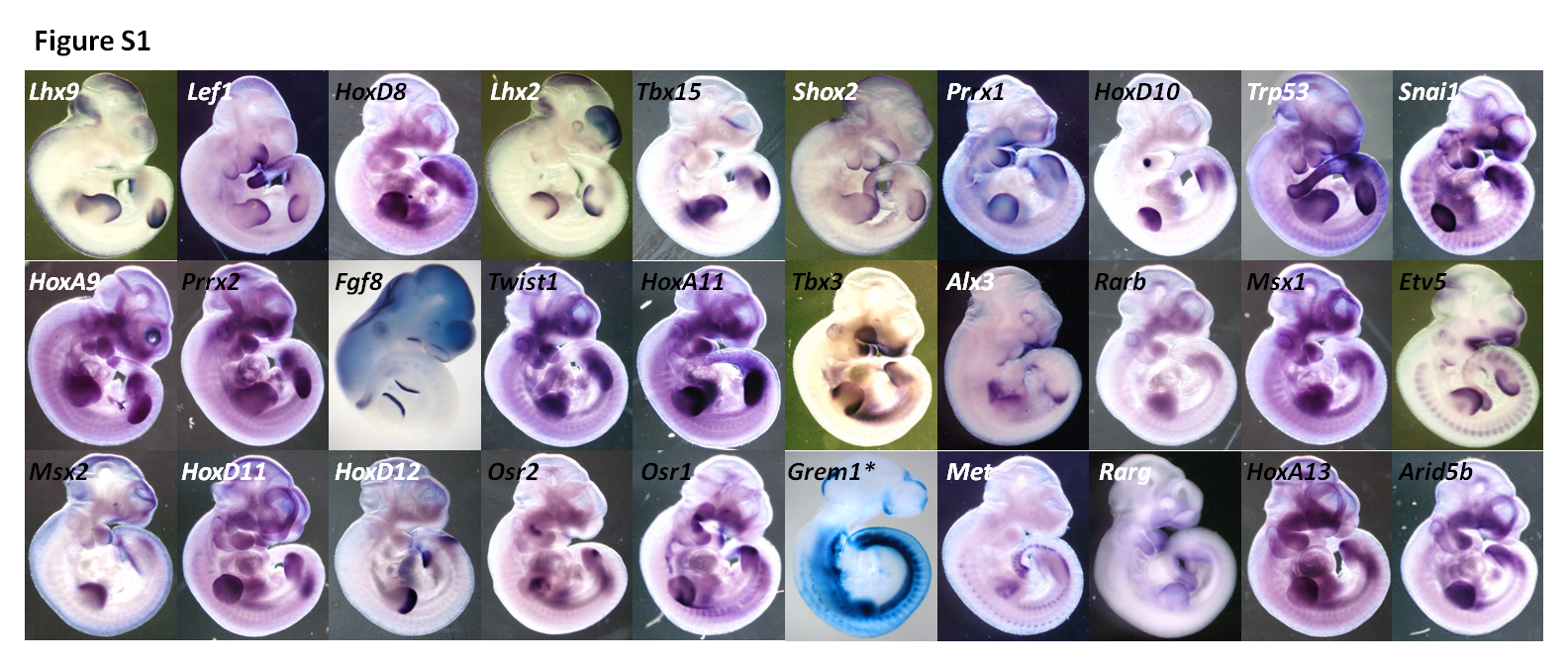

Supplement: Figure S1 — Known Genes Identified among the Top 100 Up-regulated Genes. 279 genes were examined for their putative protein function, available in situ expression in the limb, and description of null mouse phenotype. Here are some in situ expression patterns for the genes known to function during limb development. *LacZ expression pattern of the knock-in allele. (TIF) [file pone.0028358.s001.tif]

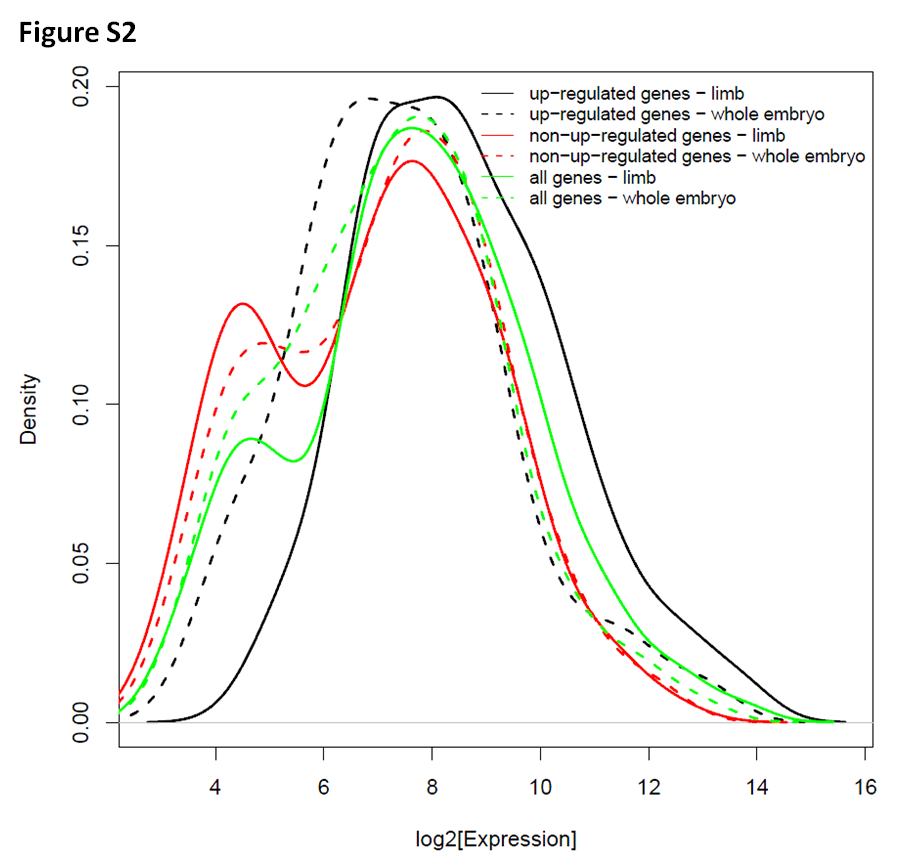

Supplement: Figure S2 — Whole Embryo and Limb Average Expression Values. Whole embryo expression values are independent of whether a gene is up-regulated or not in the limb (P-value = 0.4×10^−13^, Wilcoxon rank sum test). Solid lines represent expression distribution for limb and dashed lines for whole embryo [all (green); up-regulated in limb (black); not up-regulated (red)]. (TIF) [file pone.0028358.s002.tif]

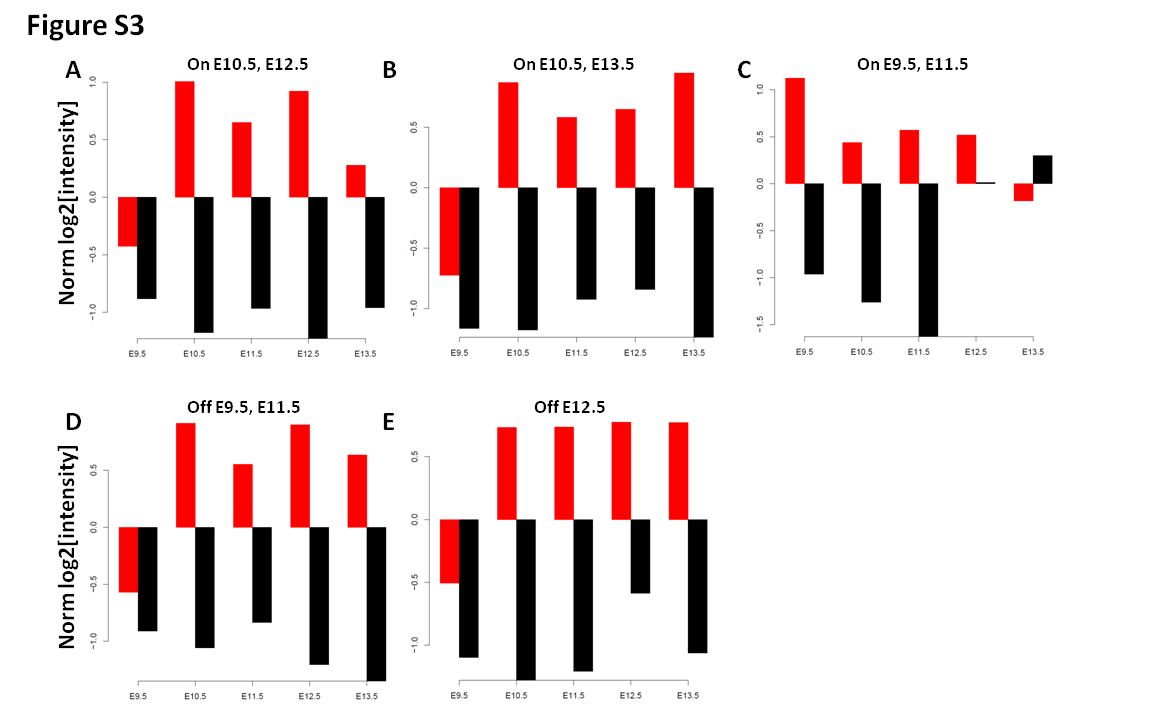

Supplement: Figure S3 — Whole Embryo and Limb Normalized Log2 Expression Values for Oscillating Genes. The log2 expression values were normalized for each gene such that the mean and standard deviation across all arrays are 0 and 1, respectively. The height of the bar represents the average of this normalized expression value for each sample. Whole embryo expression is visualized by black bars and limb expression by red bars. Each cluster (A) On E10.5, E12.5; (B) On E10.5, E13.5; (C) On E9.5, E11.5; (D) Off E9.5, E11.5; (E) Off E12.5, has whole embryo values below 0 (except for E13.5 in panel C), suggesting that whole embryo expression is very low relative to limb expression which is dominantly above 0. (TIF) [file pone.0028358.s003.tif]
