# Supplementary material for: Global Gene Expression Analysis of Murine Limb Development
Source: PLoS One. 2011 Dec 9;6(12):e28358. doi: 10.1371/journal.pone.0028358 (PMC3235105; doi:10.1371/journal.pone.0028358)
Supplement: Table S1 — Top 100 upregulated genes. (PDF) [file pone.0028358.s004.pdf]

Table S1. Top 100 upregulated genes. From each forelimb comparison (forelimb vs whole embryo) we examined the top 100 limb-enriched genes, corresponding to a total of 279 non-redundant transcripts. The putative function, expression pattern and KO phenotype of each transcript was examined in Pubmed, MGI (http://www.informatics.jax.org/phenotypes.shtml), and Visigene (http://genome.ucsc.edu/cgi-bin/hgVisiGene). For genes with KO mice available, the most descriptive reference is cited. Abbreviations: Musculoskeletal (MSK; genes highlighted in brown), Yes (Y), Normal (N), Not available (NA), embryonic lethal prior to limb formation (EL; highlighted in purple), anterior-posterior (AP), proximal-distal (PD), Knockout (KO), double knockout (dKO), embryonic day (E). *Gene also present in Table S4; **Gene also present in Table S5; ***Gene also present in Tables S4/5. ˣlimb *in situ* expression in Figure S1. ˜limb *in situ* expression detected in a species other than mouse. LogFC value that classifies a gene in the top 100 category is indicated in bold and italics, additional values are included for timepoints where the gene is significantly upregulated, but not among the top 100. Novel genes are highlighted in grey. Cited references are listed in References S1.

		E9.5 FL		E10.5 FL		E11.5 FL		E12.5 FL		E13.5 FL						
No.	Gene Symbol	logFC	Adj. p-val	logFC	Adj. p-val	logFC	Adj. p-val	logFC	Adj. p-val	logFC	Adj. p-val	Putative Function	KO phenotype	Ref	In situ	MSK Function
1	Lhx9ˣ	***3.75***	***3.90E-24***	2.78	1.5E-21	2.69	1E-20	1.89	3E-16			Transcription factor	Lhx2/Lhx9 dKOs patterning and growth dedects along AP and PD	[1]	Y	Y
2	Wif1	***3.70***	***2.30E-14***	***3.39***	***2.50E-15***	2.54	2.5E-11	1.47	2.8E-06			Wnt signaling modulator	no reported limb phenotype	[2]	Y	N
3	HoxD9	***3.69***	***1.70E-20***	2.59	2E-17	2.15	1.5E-14	1.37	1.6E-09			Transcription factor	forelimb patterning	[3-4]	Y	Y
4	Figf**	***3.27***	***2.70E-17***	1.20	2.9E-07							growth factor	no reported limb phenotype	[5]	Y	N
5	C130021I20Rik	***2.94***	***4.60E-22***	***3.95***	***2.00E-28***	***3.81***	***2.60E-27***	***3.35***	***3.10E-26***	***3.58***	***9.90E-28***	novel	unknown		NA	
6	Cdo1	***2.81***	***5.00E-20***	2.21	1.3E-18	1.41	3.2E-12					dioxigenase activity	unknown		NA	
7	Rspo4	***2.78***	***1.20E-14***	1.76	5.2E-11							wnt signaling ligand	unknown		NA	
8	Scn2b**	***2.72***	***8.00E-18***									sodium channel	no reported limb phenotype		NA	N
9	Bhlha9	***2.57***	***2.30E-15***	2.53	2.6E-17	1.63	3.2E-11					Transcription factor	unknown		NA	
10	Lef1ˣ	***2.57***	***6.00E-10***	***3.31***	***4.10E-15***	***2.95***	***3.10E-13***	2.24	2.4E-10	1.98	5.4E-09	Transcription factor	Wnt3a KO phenotype in Lef1/Tcf1 dKOs; arrested limb development at E9.5	[6]	Y	Y
11	Pfn1	***2.53***	***2.70E-04***	***4.66***	***6.90E-11***	***4.88***	***4.70E-11***	***2.53***	***2.50E-05***			Profilin1: actin binding protein	lethal, Col2pfn1 --progressive chondrodysplasia	[7]	NA	Y
12	E130012A19Rik***	***2.45***	***2.00E-04***									novel	unknown		NA	
13	Scn4b***	***2.44***	***8.00E-13***									sodium channel	unknown		NA	
14	HoxD8ˣ	***2.41***	***4.60E-15***	2.37	5.1E-17	2.23	8.8E-16	1.69	1.2E-12	2.16	6.9E-16	Transcription factor	axial skeletal patterning defects; thoracic and lumber defects	[8]	Y	Y
15	Mup1	***2.41***	***2.70E-04***	2.52	0.000013							lipocalin family member	unknown		NA	
16	Lhx2ˣ	***2.33***	***7.00E-15***	2.74	2.3E-19	2.15	1.5E-15					Transcription factor	Lhx2/Lhx9 dKOs patterning and growth dedects along AP and PD	[1]	Y	Y
17	Tbx15ˣ	***2.29***	***2.30E-15***	***3.85***	***4.70E-25***	***2.89***	***2.10E-20***	1.95	2.30E-15	1.37	6.2E-11	Transcription factor	reduction in bone size and shape	[9]	Y	Y
18	Bmp7	***2.21***	***3.00E-12***	2.75	3.20E-17	***2.89***	***1.50E-17***	2.42	1.60E-15			growth factor	Involved in chondrogenesis and osteoblast differentiation,	[10]	Y	Y
19	Shox2ˣ	***2.21***	***1.00E-16***	***3.69***	***2.90E-26***	2.71	2.40E-21	2.15	1.40E-18	1.85	1.4E-16	Transcription factor	chondrogenesis, short stature	[11]	Y	Y
20	Capn6	***2.19***	***5.50E-19***	***3.31***	***4.40E-27***	***3.00***	***3.00E-25***	2.42	1.10E-22	1.68	1.6E-17	Ca(2+)-dependent cysteine proteases	unknown		NA	
21	Cdx1***	***2.17***	***1.00E-11***									Transcription factor	skeletal defects	[12]	Y	Y
22	Cpz***	***2.17***	***3.40E-08***									carboxypepsidase	unknown		NA	
23	Pkdcc**	***2.16***	***6.20E-09***	1.67	4.7E-08	1.20	0.000034					kinase	delayed ossification; short limbs; cleft palate	[13-14]	NA	Y
24	Hoxc5**	***2.15***	***8.50E-07***					1.56	0.000011	1.55	0.000013	Transcription factor	skeletal defects; patterning of the rib cage	[15]	Y	Y
25	Tmem173	***2.15***	***1.20E-15***	1.04	7.6E-09							transmembrane protein	no reported limb phenotype	[16]	NA	N
26	Prrx1ˣ	***2.14***	***4.10E-22***	2.22	1.2E-24	1.57	3.1E-19	1.37	9.8E-18	1.16	1.4E-15	Transcription factor	prx1/2 dKOs limb abnormalities including postaxial polydactyly and	[17]	Y	Y
27	Hoxb5***	***2.14***	***2.30E-05***									Transcription factor	specifies the position of the limb	[18]	Y	Y
28	Crlf1	***2.14***	***4.90E-10***	1.93	8.4E-11	2.26	2.8E-12	1.34	3.7E-07			cytokine receptor	motorneuronal defects	[19]	NA	N
29	HoxD10ˣ	***2.11***	***3.60E-19***	***2.94***	***3.50E-26***	2.32	3.00E-22	***2.68***	***4.80E-25***	2.11	1.1E-21	Transcription factor	hindlimb defects, anterior shift in the position of patella	[20]	Y	Y
30	Trp53ˣ	***2.07***	***2.20E-04***	***3.25***	***2.60E-09***	***3.48***	***1.00E-09***					nuclear transport	abnormal osteoblast morphology, tumorigenesis	[21]	Y	Y
31	Lix1	***2.06***	***5.90E-09***	***3.82***	***7.30E-19***	2.08	1.40E-10	1.97	2.30E-10	2.02	1.3E-10	novel	unknown	[22]	Y˜	
32	Daam2	***2.05***	***3.10E-10***	1.99	7.6E-12	1.46	3.6E-08					actin binding	unknown	[23]	Y	
33	Snai1ˣ	***2.05***	***1.40E-05***	***2.82***	***5.70E-10***	***2.86***	***8.70E-10***	1.49	1.20E-04			Transcription factor	craniofacial; skeletal defect, embryonic lethal	[24]	Y	Y
34	Ifrd2	***2.04***	***8.30E-06***	2.35	1.8E-08	2.00	9.1E-07					novel	unknown		NA	
35	Wnt6	***2.04***	***3.30E-12***	1.05	5.6E-07							wnt signaling ligand	unknown	[2]	Y	
36	Cldn6	***2.02***	***2.10E-04***	1.92	0.000041							tight junction	KO has no obvious defects; normal	[25]	NA	N
37	Pdxp	***2.01***	***2.00E-05***	1.45	0.00018	1.50	0.00018					phosphatase	unknown		NA	
38	HoxA9ˣ	***2.00***	***1.60E-19***	1.32	9.1E-16					1.36	2.7E-16	Transcription factor	Forelimb and axial skeletal patterning defects	[3]	Y	Y
39	Prrx2ˣ	***1.99***	***3.50E-10***	2.24	1.1E-13	2.48	1.4E-14	2.10	6.7E-13	1.16	1.1E-06	Transcription factor	prx1/2 dKOs limb abnormalities including postaxial polydactyly and	[17]	Y	Y
40	Upf1	***1.98***	***7.20E-05***	2.27	4E-07	2.11	2.8E-06					mRNA decay	perinatal lethal by E7.5	[26]	NA	EL
41	Fgf8ˣ	***1.94***	***1.10E-05***	***4.18***	***1.00E-15***	2.07	2.40E-07	1.30	2.90E-04			growth factor/signaling	essential for limb outgrowth and patterning	[27]	Y	Y
42	Wnt7a***	***1.94***	***1.30E-11***									wnt signaling ligand	required for normal DV and AP patterning of the limb	[28]	Y	Y
43	Btbd11*	***1.94***	***7.20E-15***									novel	unknown		Y	

44	Twist1[x]	***1.93***	***1.20E-10***	1.60	2.1E-10	1.54	1.1E-09					Transcription factor	Saethre-Chotzen syndrome, limb and craniofacial defects	[29]	Y	Y
45	Epha3***	***1.93***	***1.30E-08***									receptor	muscle and nerve defects	[30]	NA	Y
46	Tshz2	***1.93***	***2.40E-14***	1.10	1.4E-09							Transcription factor	unknown	[31]	Y	
47	Snap91***	***1.92***	***2.60E-12***									novel	unknown		NA	
48	Parvb***	***1.92***	***5.50E-08***									novel	unknown		NA	
49	Pknox1	***1.91***	***1.10E-08***	2.40	3.5E-13	1.65	1.4E-08	1.83	5.1E-10	1.27	1.6E-06	Transcription factor	no reported limb phenotype	[32]	Y	N
50	Rspo2*	***1.89***	***1.10E-08***									signaling molecule	asymmetric limb malformations/truncation	[33]	Y	Y
51	Tcfap2c*	***1.88***	***2.30E-10***									Transcription factor	perinatal lethal by E9.5	[34]	Y	EL
52	Tbx2*	***1.87***	***7.00E-07***									Transcription factor	polydactyly, craniofacial defects	[35]	Y	Y
53	Asb4	***1.87***	***3.50E-10***	1.68	7.1E-11							signaling molecule	unknown		NA	
54	Ada	***1.86***	***3.80E-11***	1.17	5E-08	1.33	6E-09	1.44	3.5E-10			adenosine deaminase	perinatal lethal; rib cage defect	[36]	NA	Y
55	Smn1	***1.86***	***2.50E-05***	***4.16***	***1.80E-15***	***4.03***	***1.10E-14***	***2.65***	***5.20E-10***	1.46	0.000076	growth factor	muscle defect	[37]	NA	Y
56	Nnat	***1.85***	***8.70E-05***	***4.70***	***5.10E-16***	***4.40***	***9.50E-15***	1.83	7.5E-06			novel	unknown		NA	
57	Ntf5	***1.85***	***6.60E-08***	1.51	1.3E-07	1.20	0.000017					growth factor	neuronal defects, no reported limb phenotype	[38]	NA	N
58	Dnm1	***1.84***	***3.80E-09***	1.29	2.4E-07							cytoskeleton	nervous system, behaviour defects, no reporterd limb phenotype	[40]	NA	N
59	HoxA11[x]	***1.84***	***6.00E-12***	***3.39***	***1.80E-22***	***3.04***	***1.70E-20***	2.01	3.2E-15	1.81	6.8E-14	Transcription factor	Hoxa11/hoxd11 defects in radius and ulna formation	[39]	Y	Y
60	Pcgf1	***1.84***	***1.30E-05***	***3.34***	***2.40E-13***	***3.32***	***6.60E-13***	1.77	1.2E-06			Transcription factor	unknown		Y	
61	Mmp2	***1.82***	***3.70E-09***	1.60	1.3E-09	1.35	1.3E-07					extracellular matrix	cardiovascular, nervous system, muscle defects, no reported limb	[41]	NA	N
62	Gal***	***1.82***	***9.90E-06***									signaling molecule	nervous system defects, no reported limb phenotype	[42]	NA	N
63	HoxB6***	***1.81***	***9.00E-06***									Transcription factor	specifies the position of the limb	[18]	Y	Y
64	Krt5	***1.81***	***1.80E-11***	***2.98***	***2.60E-20***	2.38	8.6E-17					structural molecule	limbs lack epidermis	[43]	NA	Y
65	Bin1	***1.81***	***2.30E-11***	1.28	2.5E-09	1.42	4E-10					signaling molecule	muscle defect	[44]	NA	Y
66	Tbx3[x]	***1.80***	***8.00E-11***	2.08	1E-14	1.77	3E-12	1.87	2.3E-13	2.10	6.5E-15	Transcription factor	limb abnormalities	[45]	Y	Y
67	Lsm2	***1.80***	***1.10E-04***	2.75	1.4E-09	2.78	2.3E-09					novel	unknown		NA	
68	Emid2	***1.79***	***3.30E-07***	1.81	6.3E-09	1.60	1.8E-07					novel	unknown		NA	
69	Ctsk*	***1.79***	***3.00E-12***									peptidase	hypermineralization of bones	[46]	NA	Y
70	Xab2*	***1.78***	***2.00E-04***									DNA repair	perinetal lethal by E3.5	[47]	NA	EL
71	Alx3[x]	***1.78***	***3.40E-07***	1.19	0.00002	1.26	0.000013					Transcription factor	Alx3/Alx4 double mutant mice have limb and craniofacial defects	[48]	Y	Y
72	Sertad4*	***1.78***	***1.10E-13***									novel	unknown		NA	
73	Tbx5**	***1.77***	***2.90E-08***	2.45	6.3E-14	2.77	3.9E-15	***3.03***	***7.40E-17***	***2.56***	***1.30E-14***	Transcription factor	essential for forelimb initiation and patterning of the limb	[49]	Y	Y
74	1200009O22Rik	***1.77***	***2.00E-17***	1.11	3.1E-13							novel	unknown		NA	
75	Khsrp***	***1.76***	***0.0008***									mRNA splicing	unknown		NA	
76	Rarb[x]	***1.75***	***8.30E-09***	1.84	3.5E-11			1.66	4.9E-10	***2.26***	***7.70E-14***	growth factor/signaling	RAR double mutants have craniofacial and skeletal	[50]	Y	Y
77	Slc4a2	***1.72***	***1.40E-04***	1.92	1.7E-06	1.83	0.000007					anion transport	abnormal skeletal development	[51]	NA	Y
78	Msx1[x]	***1.70***	***2.30E-09***	1.96	5.9E-13	1.93	2.2E-12	1.93	8E-13	1.64	6.8E-11	Transcription factor	limb abnormalities	[52]	Y	Y
79	Perp***	***1.68***	***1.80E-09***									cell junction	immune defect; no reporter limb phenotype	[53]	NA	N
80	Lbx1	***1.68***	***1.40E-12***	1.98	6.9E-17	1.24	1.5E-10					Transcription factor	limb muscle specification	[54]	Y	Y
81	Lad1*	***1.67***	***2.10E-07***									novel	unknown		NA	
82	Etv5[x]	***1.66***	***4.00E-12***	2.37	4.9E-19	2.20	1.3E-17	1.37	8E-12	1.06	5.3E-09	Transcription factor	expansion of AP axis	[55]	Y	Y
83	Spry1*	***1.66***	***1.80E-07***									growth factor	none reported	[56]	Y	N
84	Dlk1	***1.65***	***6.50E-05***	1.23	0.00036	1.47	0.000049					Notch antagonist	rib cage defects	[57]	NA	Y
85	Msx2[x]	***1.64***	***3.10E-10***	1.78	3.1E-13	1.72	1.9E-12	1.51	3.2E-11			Transcription factor	msx1/msx2 dKOs severe limb defects	[58]	Y	Y
86	Map2k6	***1.63***	***1.00E-04***	2.77	7.9E-11	2.64	6.1E-10	1.32	0.00018			kinase	no reported limb phenotype		NA	N
87	Ptp4a3	***1.63***	***2.90E-04***			1.30	0.00086					phosphatase	unknown		NA	
88	Plekhj1	***1.63***	***3.10E-04***	***2.83***	***4.10E-10***	***2.88***	***5.80E-10***					novel	unknown		NA	
89	Asna1	***1.63***	***5.40E-04***	***3.14***	***1.00E-10***	***3.72***	***2.30E-12***	2.15	5.8E-07			ATP binding	perinatal lethal by E8.5	[59]	NA	EL
90	Pddc1	***1.62***	***1.30E-04***	2.47	2E-09	2.11	1.5E-07					novel	unknown		NA	
91	Pdgfa**	***1.61***	***6.80E-08***			1.26	5.6E-07					growth factor	embyonic lethal before E10.5; skeletal, muscle and vascular	[60]	Y	Y
92	Trp63	***1.61***	***1.50E-15***	1.11	8.8E-13	1.11	2.1E-12	1.33	4.1E-15			chromatin	kyphosis	[61]	NA	Y
93	Csk*	***1.61***	***2.60E-05***									kinase	perinatal lethaby by E10.5	[62]	Y	EL
94	Capg	***1.61***	***1.10E-06***	1.41	4.6E-07	1.64	3.9E-08					actin binding	immune defect; no reporter limb phenotype	[63]	NA	N
95	6230427J02Rik	***1.60***	***1.40E-11***	1.81	3.1E-15	1.61	2.8E-13	1.12	1.8E-09			novel	unknown		NA	
96	Prl2c2***	***1.60***	***4.30E-07***									growth	unknown		NA	
97	Col14a1***	***1.57***	***5.10E-08***									extracellular matrix	muscle, skeleton, tendon defects	[64]	NA	Y
98	Alx1***	***1.57***	***2.20E-05***									Transcription factor	craniofacial and skeletal defects--limb defects as double KO with Alx4	[65]	NA	Y
99	Atp11b*	***1.57***	***1.80E-04***									ATP binding	unknown		NA	
100	Tmem119	***1.57***	***2.40E-07***	1.39	8.1E-08	1.21	2.3E-06					transmembrane	unknown		NA	
101	AI506816			***5.43***	***9.10E-38***	***5.46***	***3.10E-37***	***5.05***	***1.80E-36***	***4.90***	***2.20E-36***	novel	unknown		NA	
102	Phf6			***5.29***	***4.70E-25***	***4.78***	***3.70E-23***	***4.22***	***7.20E-22***	***3.87***	***8.60E-21***	Transcription factor	unknown		NA	
103	Zfp146	1.11	0.00089	***4.85***	***6.00E-21***	***4.89***	***1.20E-20***	***5.29***	***2.30E-22***	***4.16***	***4.80E-19***	Transcription factor	unknown		Y	
104	Snurf			***4.79***	***5.20E-20***	***4.19***	***8.80E-18***	***3.09***	***4.10E-14***			nuclear protein	Prader-Willi Syndrome	[66]	NA	Y
105	HoxD11[x]			***4.74***	***1.20E-28***	***4.34***	***6.60E-27***	***3.95***	***3.10E-26***	***2.85***	***1.30E-21***	Transcription factor	appendicular skeletal defects	[67]	Y	Y
106	HoxD12[x]			***4.50***	***4.10E-19***	***5.40***	***2.70E-21***	***4.73***	***6.10E-20***	***2.85***	***4.60E-13***	Transcription factor	limb defects	[67]	Y	Y
107	Cap1			***4.49***	***1.20E-23***	***4.91***	***1.60E-24***	***4.34***	***2.30E-23***	***5.43***	***3.50E-27***	actin binding	unknown		NA	
108	Ubl7			***4.43***	***4.10E-19***	***4.33***	***2.10E-18***	***2.87***	***2.50E-13***	1.92	1.00E-08	ubiquitin like	unknown		NA	
109	Net1			***4.22***	***1.90E-21***	***4.63***	***1.70E-22***	***3.81***	***3.60E-20***	***3.30***	***3.00E-18***	growth factor	unknown		NA	
110	Pmaip1			***4.16***	***2.50E-13***	2.36	6.5E-07	1.41	0.00082			apoptosis	hematopoietic defect	[68]	NA	N

111	Osr2*			4.16	3.70E-19	3.86	1.00E-17	2.12	1.8E-10			Transcription factor	skeletal defects	[69]	Y	Y
112	Osr1*			4.08	6.00E-21	5.28	2.60E-24	4.20	1.50E-21	3.42	9.90E-19	Transcription factor	skeletal defects	[69]	Y	Y
113	Gorab			4.02	2.50E-21	3.86	2.20E-20	3.58	8.10E-20	2.99	2.20E-17	novel	unknown		NA	
114	Cox6b2			4.00	1.80E-20	3.98	5.10E-20	2.60	1.40E-14	1.18	3.7E-06	cytocrome C subunit	unknown		NA	
115	Cxcl14	1.26	0.000049	3.92	2.00E-19	4.04	1.80E-19	2.52	1.80E-13	1.63	1.8E-08	chemokine	no reported limb phenotype	[70]	NA	N
116	Grem1			3.83	1.90E-12	1.88	0.000028	3.05	7.40E-10	1.89	0.000015	growth factor	limb defects	[71]	Y	Y
117	BC056474			3.82	5.20E-14	4.00	3.30E-14	2.14	1.5E-07	1.27	0.00068	novel	unknown		NA	
118	Ptk7			3.78	2.50E-22	3.71	1.20E-21	2.82	3.20E-18	2.85	1.60E-18	tyrosine kinase	polydactyly	[72]	NA	Y
119	Cfl1			3.75	2.40E-13	3.99	9.60E-14	2.45	1.6E-08	2.24	1.1E-07	actin binding	perinetal lethality by E9.5	[73]	NA	EL
120	Scaper			3.72	4.00E-11	3.13	6.80E-09	3.92	9.00E-12	3.83	1.60E-11	novel	unknown		NA	
121	Met*			3.72	4.20E-20	3.22	1.00E-17	2.78	4.00E-16	1.78	1.6E-10	proto-oncogene	muscle and craniofactial defects	[74]	Y	Y
122	Ifitm2	1.32	0.00044	3.69	1.30E-15	4.33	2.10E-17	2.61	2.90E-11	1.76	3.1E-07	membrane bound	unknown		Y	
123	a			3.68	3.90E-20	2.76	8.9E-16	2.02	3.5E-12	2.08	1.4E-12	growth factor	no reported limb phenotype	[75]	NA	N
124	Csrp1			3.67	6.10E-26	4.12	3.70E-27	4.25	2.60E-28	3.74	6.10E-27	actin binding	unknown		NA	
125	Zbtb12			3.65	2.50E-17	4.29	3.20E-19	3.90	2.50E-18	2.99	9.80E-15	novel	unknown		NA	
126	Npm3			3.63	3.20E-24	3.77	2.40E-24	3.75	6.00E-25	2.46	7.60E-19	rRNA processing	unknown		NA	
127	Otub1			3.63	3.20E-17	3.55	1.60E-16	3.03	7.80E-15	1.87	4.7E-09	ubiquitin like	unknown		Y	
128	Slc25a24			3.62	1.60E-26	3.65	2.80E-26	3.34	1.20E-25	3.54	4.90E-27	membrane bound	unknown		NA	
129	Ccdc109b			3.61	2.30E-20	2.72	5.1E-16	1.76	5.8E-11			novel	unknown		NA	
130	Aes			3.61	4.00E-14	3.09	8.70E-12	1.58	0.000012			Transcription factor	limb/skeletal defects	[76]	NA	Y
131	Frg1			3.55	1.60E-08	4.67	3.80E-11					mRNA processing	unknown		NA	
132	Tubb2a-ps2			3.50	7.40E-17	4.10	1.20E-18	3.82	4.10E-18	2.39	5.90E-12	novel	unknown		NA	
133	Nolc1	1.23	3.2E-06	3.50	1.50E-20	3.31	2.20E-19	3.07	8.70E-19	2.17	4.2E-14	novel	unknown		NA	
134	Supt16h			3.48	9.60E-21	3.42	4.20E-20	2.72	2.40E-17	1.62	9.9E-11	DNA repair	unknown		NA	
135	Zfa			3.38	4.50E-14	3.24	4.00E-13	3.88	5.90E-16	2.48	2.20E-10	transcription factor	no reported limb phenotype	[77]	NA	N
136	Pdia4	1.02	0.0003	3.32	3.70E-18	3.23	2.10E-17	3.01	6.90E-17	2.80	5.50E-16	catalytic enzyme	unknown		NA	
137	Pi4kb			3.26	3.60E-23	3.74	8.30E-25	3.95	3.00E-26	3.95	8.50E-27	catalytic enzyme	unknown		NA	
138	Mab21l2			3.25	6.80E-09	2.52	2.5E-06					nuclear transport	perinetal lethality by E14.5/no reporter limb defects	[78]	Y	EL
139	Efna4	1.23	2.6E-09	3.23	2.20E-24	3.09	2.10E-23	2.73	4.00E-22	2.02	7.4E-18	membrane	unknown		NA	
140	Hdac8			3.18	5.70E-21	2.66	4.6E-18	2.66	1.50E-18	1.05	2.4E-07	chromatin	perinetal lethality/craniofacial	[79]	NA	Y
141	HoxD13			3.18	2.40E-17	3.23	3.30E-17	2.91	3.20E-16	2.83	6.80E-16	transcription factor	limb defects	[80]	Y	Y
142	Zfp2			3.17	2.30E-21	2.78	4E-19	1.88	3.3E-14	1.34	3.8E-10	transcription factor	unknown		NA	
143	Aass			3.17	1.70E-18	2.41	2.3E-14	2.07	7.8E-13			catalytic enzyme	unknown		NA	
144	Pecam1			3.17	2.70E-24	3.34	1.20E-24	3.11	2.70E-24	3.12	1.10E-24	cell adhesion	arthritis/joint inflammation	[81]	Y	Y
145	A730054J21Rik			3.15	2.70E-18	3.08	1.40E-17	2.88	4.40E-17	1.58	1.3E-09	novel	unknown		NA	
146	Rabl3	1.03	0.00013	3.13	4.70E-18	2.45	2.7E-14	1.98	4.7E-12	1.18	1.1E-06	GTPase	unknown		NA	
147	Calml4			3.11	7.00E-16	1.70	2.3E-08	1.60	4.2E-08			Ca-binding	unknown		NA	
148	Akt1s1			3.11	1.10E-20	3.83	2.30E-23	2.88	9.60E-20	1.54	2E-11	novel	unknown		NA	
149	Dusp7	1.49	0.000011	3.10	3.00E-15	3.17	3.90E-15	1.81	7.5E-09	1.99	7.3E-10	dephosphorylation	unknown		NA	
150	Ube2s			3.10	6.30E-26	3.57	2.50E-27	3.24	9.80E-27	2.59	9.30E-24	ubiquitin like	unknown		NA	
151	Hyls1			3.08	4.60E-18	2.79	2.7E-16	3.07	4.30E-18	2.60	7.50E-16	novel	unknown		NA	
152	Nrk			3.07	8.30E-10	2.76	2.2E-08	3.46	3.50E-11	3.64	8.50E-12	kinase	unknown		Y	
153	Tmem87a			3.07	4.70E-25	3.47	2.20E-26	3.93	5.30E-29	4.23	9.40E-31	membrane bound	unknown		NA	
154	Fibin			3.06	1.00E-16	1.71	3.5E-09	1.31	5.1E-07	1.32	4.4E-07	novel	unknown		NA	
155	Dynlrb1			3.05	9.80E-22	3.14	8.80E-22	1.89	4.3E-15	1.45	8.2E-12	cytoskeleton	unknown		NA	
156	D10627			3.02	6.00E-23	2.53	5.5E-20	3.17	6.40E-24	2.74	5.50E-22	novel	unknown		NA	
157	Sdccag3	1.20	2.6E-06	3.01	5.50E-19	2.97	2.00E-18	2.07	5.5E-14	1.64	4E-11	novel	unknown		NA	
158	Rarg*	1.56	1.8E-07	3.00	3.30E-17	2.89	2.80E-16	1.96	9.8E-12	1.47	1.6E-08	membrane	stunted growth, axial skeletal	[82]	Y	Y
159	Cbl			3.00	7.30E-07	2.08	0.00036	2.79	2.60E-06	3.37	6.10E-08	signaling molecule	hematopoietic defect	[83]	Y	N
160	Ptbp1			3.00	5.90E-17	3.30	6.30E-18	2.94	8.80E-17	2.68	1.40E-15	mRNA processing	unknown		NA	
161	1700066M21Rik			2.99	1.80E-15	2.55	5.5E-13	2.80	1.20E-14	1.61	3E-08	novel	unknown		NA	
162	8430426H19Rik			2.97	3.30E-17	3.04	4.20E-17	3.00	2.20E-17	2.54	3.50E-15	novel	unknown		NA	
163	Gna12			2.97	1.20E-20	2.72	4.5E-19	2.20	1.5E-16	1.66	7.7E-13	guanine binding	no phenotype	[84]	NA	N
164	Atp5j2			2.96	1.60E-18	3.31	1.00E-19	2.87	4.00E-18	2.21	1.2E-14	ATP binding	unknown		NA	
165	Kdm5a			2.95	2.20E-07	2.17	0.000078	3.14	5.70E-08	3.06	9.90E-08	chromatin	postnatal lethality; immune and hematopoietic defects		NA	N
166	Ranbp6			2.94	1.50E-16	2.38	2.4E-13	2.64	3.70E-15	2.39	7.20E-14	protein transport	unknown		NA	
167	Bcam			2.93	6.50E-20	2.78	8.7E-19	2.51	8.00E-18	2.44	1.30E-17	adhesion molecule	no reported limb phenotype	[85]	NA	N
168	Ephb4	1.14	0.00047	2.92	2.60E-14	3.30	1.60E-15	2.23	5.3E-11	2.09	2.6E-10	membrane/receptor	perinetal lethality	[86]	NA	EL
169	Rac3			2.91	3.50E-18	3.33	1.00E-19	2.84	6.40E-18	1.05	1.8E-06	ATP/GTP binding	no reported limb phenotype		NA	N
170	G6pd2			2.90	6.00E-23	2.24	7.5E-19	2.03	6.7E-18			catalytic enzyme	unknown		NA	
171	Gsc			2.88	5.20E-11	1.79	4.1E-06					transcription factor	skeletal and craniofacial defects	[87]	Y	Y
172	Hipk3			2.88	1.50E-13	3.09	4.30E-14	3.60	1.40E-16	2.60	2.20E-12	ATP binding	unknown		NA	
173	Fanci			2.88	1.20E-11	2.00	1.5E-07	2.30	3.3E-09			DNA repair/cell cycle	unknown		NA	
174	Tnrc18			2.87	7.30E-07	2.52	0.000013	2.93	4.70E-07	3.03	2.50E-07	novel	unknown		NA	
175	Pttg1			2.85	3.00E-26	2.87	6.10E-26	2.78	3.70E-26	2.01	1.5E-21	cell cycle/DNA repair	lethal E11.5	[88]	NA	EL
176	Mme			2.85	1.10E-16	3.58	1.60E-19	3.69	1.70E-20	3.34	3.20E-19	membrane bound	no reported limb phenotype	[89]	NA	N
177	Taf7			2.84	2.20E-23	2.85	4.50E-23	2.57	4.00E-22	2.42	1.90E-21	transcription factor	unknown		NA	
178	Usf1			2.82	3.30E-14	2.31	2.5E-11	2.19	4E-11	1.81	5.1E-09	transcription factor	nervous system defects, no reported limb phenotype		Y	N
179	Rab4a			2.81	2.20E-12	2.71	1.5E-11	1.52	3.6E-06			oncogene	unknown		NA	
180	Rab5c			2.81	5.50E-15	3.03	1.40E-15	2.80	5.60E-15	2.43	3.30E-13	oncogene	unknown		NA	
181	Arpc4	1.43	0.00071	2.79	1.50E-10	3.00	5.20E-11					actin binding	unknown		NA	
182	Kera					4.33	3.40E-17	3.35	3.70E-14	1.50	8.70E-06	extracellular matrix	eye defects	[90]	NA	N
183	Matn4					4.31	1.20E-18	3.73	4.50E-17	3.09	1.4E-14	extracellular matrix	unknown		NA	
184	HoxA13*			2.38	7E-08	4.02	2.20E-13	3.60	2.00E-12	2.13	6.7E-07	transcription factor	loss of the most anterior digit of all feet	[91]	Y	Y
185	Gdpd3			2.46	1.5E-14	3.72	7.50E-20	3.61	5.80E-20	2.69	6.80E-16	membrane	unknown		NA	
186	Gdf5					3.69	9.70E-17	3.09	8.40E-15	2.37	1.90E-11	growth factor	limb and joint defects	[92]	Y	Y
187	Myl1					3.59	3.70E-08	3.30	1.10E-07			myosin	muscle defect	[93]	Y	Y
188	Col1a1			2.47	2.2E-09	i	7.90E-13	2.18	4E-08	1.64	9.6E-06	collagen	impaired bone formation	[94]	Y	Y

189	Krt14	1.01	0.00035	2.51	2.6E-14	**3.40**	**5.00E-18**	1.61	4.2E-09			keratin	skin defect	[95]	NA	Y
190	Id1			2.63	2.4E-19	**3.31**	**3.00E-22**	**2.92**	**5.40E-21**	**2.36**	**5.00E-18**	transcription factor	vascular defects	[96]	Y	N
191	Atp5l			2.74	8E-22	**3.21**	**8.80E-24**	**2.81**	**2.10E-22**	**2.46**	**1.10E-20**	ATP metabolism	unknown		NA	
192	Fgf18			1.48	0.000075	**3.18**	**1.60E-11**	**2.52**	**2.70E-09**	1.21	0.00096	growth factor/signaling	osteoblast and chondrocyte defect	[97]	NA	Y
193	Ogn					**3.14**	**8.30E-15**	**2.74**	**1.60E-13**	2.07	3.6E-10	extracellular matrix	collagen fiber defects	[98]	NA	Y
194	Hadha			2.52	6.7E-20	**3.13**	**1.20E-22**	2.16	9.1E-18	1.38	6.9E-12	metabolism	liver defects	[99]	NA	N
195	Kcne3	1.40	2.3E-06	2.77	9.3E-16	**3.11**	**5.80E-17**	**2.94**	**1.30E-16**	2.08	3.7E-12	channel /membrane	interstinal transport	[100]	NA	N
196	Hist1h2ae			2.74	9.3E-18	**3.09**	**4.70E-19**	**2.71**	**1.10E-17**	**2.69**	**1.00E-17**	chromatin	unknown		NA	
197	Vps72			2.44	4.1E-07	**3.06**	**6.00E-09**	1.54	0.00051			transcription factor	unknown		Y	
198	Hoxa11as			2.58	1.4E-19	**2.99**	**2.70E-21**	2.45	5.7E-19	2.03	2E-16	antisense RNA	unknown		NA	
199	Hist1h2bp			2.57	1.5E-19	**2.98**	**2.80E-21**	**2.90**	**2.10E-21**	1.75	2E-14	chromatin	unknown		NA	
200	Kbtbd10**					**2.97**	**1.40E-10**	1.97	7.1E-07			novel	unknown		NA	
201	Rps24			2.30	1.5E-20	**2.95**	**9.10E-24**	1.70	2.3E-16	1.80	2.7E-17	ribosomal protein	unknown		NA	
202	Papss2					**2.92**	**5.30E-14**	**2.68**	**2.50E-13**			ATP binding	shorter limbs	[101]	NA	Y
203	Fam171a1	1.19	0.00072	2.64	4.3E-12	**2.91**	**6.60E-13**	1.92	1.3E-08	1.29	0.00003	novel	unknown		NA	
204	Pdk2			2.10	3.8E-10	**2.91**	**1.20E-13**	1.26	0.000014			ATP binding	unknown		NA	
205	Fxc1			2.67	3.3E-22	**2.90**	**4.80E-23**	2.39	9.4E-21	1.44	9.6E-14	mitochondrial	unknown		NA	
206	Ctse			1.63	6.1E-07	**2.86**	**1.50E-12**	1.50	2.9E-06			metabolism	skin defect	[102]	NA	N
207	Eif4a1			2.79	2.6E-24	**2.86**	**3.00E-24**	2.16	1.1E-20	2.11	1.4E-20	ATP binding	unknown		NA	
208	Snrpb2			2.62	4.7E-25	**2.84**	**8.50E-26**	**2.49**	**1.50E-24**	**2.25**	**2.40E-23**	novel	unknown		NA	
209	Maz			2.57	8.8E-13	**2.81**	**1.60E-13**	1.82	7E-09	1.29	7.1E-06	transcription factor	unknown		NA	
210	Uqcr10			2.51	6.2E-22	**2.80**	**3.30E-23**	2.46	8.4E-22	2.12	8.1E-20	membrane	unknown		NA	
211	Cdc23			2.78	2.4E-19	**2.80**	**4.60E-19**	2.26	1.6E-16	1.53	1.7E-11	cell cycle/DNA repair	unknown		NA	
212	Krt17					**2.80**	**1.90E-16**	2.41	7E-15			keratin	skin defect	[103]	NA	N
213	Mybpc1			1.45	3.1E-07	2.08	1.6E-10	**3.32**	**6.40E-17**	**3.26**	**8.40E-17**	cytoskeleton	unknown		NA	
214	Add3			2.41	6.5E-16	2.41	1.6E-15	**3.16**	**7.80E-20**	**3.07**	**1.50E-19**	cytoskeleton	cardiavascular	[104]	NA	N
215	Kctd12b			1.40	0.00013	1.78	5.2E-06	**3.04**	**1.50E-11**	**2.97**	**2.70E-11**	channel /membrane	unknown		NA	
216	Nog					1.84	2.5E-08	**3.02**	**1.90E-14**	1.36	5.6E-06	growth factor	skeletal defects	[105]	Y	Y
217	Aspn					1.19	0.0007	**2.87**	**1.40E-11**	1.77	1.2E-06	extracellular matrix	unknown		NA	
218	Arpc3			2.05	8.5E-10	2.27	1.3E-10	**2.86**	**7.90E-14**	1.29	0.000012	actin binding	perinatal lethal by E5.5	[106]	NA	EL
219	Laptm4a			2.70	6.4E-20	2.55	9.5E-19	**2.84**	**8.60E-21**	1.98	9E-16	membrane	unknown		NA	
220	Megf9			2.26	2.1E-08	2.36	1.6E-08	**2.83**	**8.00E-11**	**2.90**	**3.80E-11**	receptor	unknown		NA	
221	Loxl2			1.93	9E-10	2.50	2E-12	**2.79**	**2.70E-14**	2.04	1.8E-10	oxydase	unknown		NA	
222	Rnf25			2.31	1.5E-19	2.70	2.4E-21	**2.77**	**2.70E-22**	1.79	3.5E-16	ligase	unknown		Y	
223	Igfbp5							**2.73**	**4.00E-13**	**2.93**	**4.40E-14**	growth factor	no reported limb phenotype	[107]	NA	N
224	Sox8			2.21	3.9E-11	1.92	3.2E-09	**2.71**	**1.10E-13**	**2.49**	**1.20E-12**	transcription factor	abnormal bone morphology	[108]	NA	Y
225	Cdk14			2.42	4.1E-09	1.97	6.5E-07	**2.69**	**2.50E-10**	1.53	0.000032	kinase	unknown		NA	
226	Zfp458			2.35	4.3E-11	2.29	1.9E-10	**2.58**	**3.20E-12**	**2.37**	**3.00E-11**	transcription factor	unknown		NA	
227	Cytl1					1.09	0.00059	**2.56**	**2.10E-11**			growth	unknown		NA	
228	Tmed2			2.73	1.3E-18	2.75	2.5E-18	**2.54**	**1.00E-17**	2.22	6E-16	membrane	limb defects	[109]	NA	Y
229	Wdfy1			2.64	4E-28	2.48	1.1E-26	**2.54**	**1.00E-27**	2.08	3.6E-25	novel	unknown		NA	
230	BC005561			2.33	1.1E-10	1.87	4.3E-08	**2.54**	**9.60E-12**	**2.92**	**1.60E-13**	novel	unknown		NA	
231	Gm13251			1.58	3.2E-10	2.24	4E-14	**2.54**	**3.10E-16**	1.84	4.5E-12	novel	unknown		NA	
232	Gabrp			1.54	1.3E-09	2.65	7.3E-16	**2.53**	**1.00E-15**	**2.47**	**1.80E-15**	membrane	normal/no phenotype		NA	N
233	1200011I18Rik			2.32	2.3E-15	2.26	1.2E-14	**2.53**	**1.30E-16**	1.77	5.7E-12	novel	unknown		NA	
234	Casc5			2.39	9.7E-10	1.86	5.4E-07	**2.50**	**3.00E-10**	2.15	1.1E-08	novel	unknown		NA	
235	Gm3168			1.27	7.9E-07	2.04	2.5E-11	**2.50**	**3.00E-14**	**2.32**	**2.40E-13**	novel	unknown		NA	
236	Ppp3r1					1.06	1.2E-07	**2.47**	**2.80E-18**	1.76	1E-13	catalytic enzyme	perinetal lethal by E10.5/vascular defects	[110]	NA	EL
237	4931406C07Rik			2.45	5.8E-20	2.72	4.7E-21	**2.46**	**3.80E-20**	2.00	2.8E-17	novel	unknown		NA	
238	Dnm3os							1.55	0.000017	**4.81**	**3.30E-18**	novel	skeletal defects	[111]	NA	Y
239	AI503316							1.02	1.8E-07	**3.66**	**3.00E-24**	novel	unknown		NA	
240	Gas5*									**3.61**	**4.90E-19**	novel	unknown		NA	
241	2810043O03Rik*									**3.61**	**2.70E-13**	novel	unknown		NA	
242	Mirg			1.06	9.4E-08	1.49	6.1E-11	1.87	3.5E-14	**3.54**	**1.40E-23**	novel	unknown		NA	
243	6430537K16Rik*									**3.35**	**6.90E-15**	novel	unknown		NA	
244	BC023969			1.68	8.3E-09	1.37	1.1E-06	1.92	2.7E-10	**3.24**	**4.90E-17**	novel	unknown		NA	
245	Slc4a7							1.82	2.7E-06	**2.97**	**4.10E-11**	membrane	blindness/hearing	[112]	NA	N
246	Ogt			1.76	5.6E-17	1.49	2.3E-14	1.82	1.5E-17	**2.96**	**4.20E-25**	metabolism	perinatal lethal	[113]	NA	EL
247	Fcho2			1.92	2.1E-10	1.56	6E-08	2.28	1.8E-12	**2.86**	**1.80E-15**	novel	unknown		NA	
248	Mirhg1									**2.85**	**3.00E-15**	novel	unknown		NA	
249	1110006E14Rik*									**2.84**	**1.30E-17**	novel	unknown		NA	
250	2310003F16Rik*									**2.84**	**6.30E-15**	novel	unknown		NA	
251	D2Ertd173e*									**2.80**	**1.40E-08**	novel	unknown		NA	
252	2700023E23Rik							1.92	7.5E-17	**2.77**	**2.00E-22**	novel	unknown		NA	
253	2900010J23Rik			1.99	3.8E-21	2.16	6.4E-22	2.45	1.8E-24	**2.61**	**8.60E-26**	novel	unknown		NA	
254	Ccnl1					1.20	2E-15	1.33	3.1E-17	**2.60**	**1.20E-27**	kinase	unknown		NA	
255	2310067E19Rik*									**2.59**	**1.70E-10**	novel	unknown		NA	
256	Zfhx4			2.79	1.3E-20	1.99	1.7E-15	2.05	2.2E-16	**2.55**	**1.20E-19**	transcription factor	unknown		Y	
257	Crebzf			2.16	1E-15	2.26	5.8E-16	1.74	5.9E-13	**2.54**	**3.40E-18**	transcription factor	unknown		NA	
258	Sln							1.92	9.1E-08	**2.51**	**1.70E-10**	membrane	muscle defect	[114]	NA	Y
259	Dkk2					1.92	2.2E-08	1.66	2.4E-07	**2.48**	**1.60E-11**	Wnt antagonist	osteoblast defect	[115]	Y	Y
260	E030016H06Rik*									**2.48**	**6.70E-12**	novel	unknown		NA	
261	Fbn2			1.90	3.2E-11	2.09	5E-12	2.15	8.7E-13	**2.45**	**1.60E-14**	extracellular matrix	limb defects/syndactyly	[116]	NA	Y
262	Tbx18			2.69	2.6E-19	2.49	7.6E-18	2.24	7.7E-17	**2.41**	**5.20E-18**	transcription factor	muscle and skeletal defects	[117]	Y	Y
263	D5Ertd798e*									**2.40**	**1.70E-09**	novel	unknown		NA	
264	A430108E01Rik							1.22	4.4E-06	**2.40**	**4.10E-13**	novel	unknown		NA	
265	Ramp2			1.68	0.00014					**2.40**	**3.70E-07**	membrane	embryonic lethal/cardiovascular	[118]	NA	N
266	Ccdc39*									**2.39**	**3.50E-14**	novel	unknown		NA	
267	Sp4			2.22	5.5E-07	1.82	0.00003	2.27	3.3E-07	**2.39**	**1.20E-07**	transcription factor	cardiovascular/neonatal lethal	[119]	Y	N

268	Rtl1*							***2.36***	***4.60E-17***	novel	perinatal lethal E15.5-E19	[120]	NA	EL
269	Clk4	1.48	9.2E-11	1.28	7.3E-09	1.80	3.5E-13	***2.35***	***7.90E-17***	kinase	unknown		NA	
270	Fyttd1	1.94	2E-14	2.04	1.1E-14	2.41	1.8E-17	***2.34***	***3.50E-17***	novel	unknown		NA	
271	9630010G10Rik*							***2.34***	***1.20E-13***	novel	unknown		NA	
272	Rbm39*							***2.33***	***2.00E-22***	transcription factor	lethal	[121]	NA	EL
273	Arid5b*	2.15	0.000017	2.06	0.000054	2.22	9.3E-06	***2.28***	***6.30E-06***	transcription factor	lethal/skeletal defect	[122]	Y	Y
274	Ttc14*							***2.27***	***8.90E-18***	novel	unknown		NA	
275	Nsa2	1.97	4.1E-22	2.21	1.9E-23	1.97	2.8E-22	***2.27***	***7.80E-25***	novel	unknown		NA	
276	5830474E16Rik*							***2.27***	***6.10E-10***	novel	unknown		NA	
277	Zfp101			1.01	3.7E-09	1.33	1.1E-12	***2.26***	***3.70E-20***	transcription factor	unknown		Y	
278	Tgfbr3			1.38	1.1E-07	1.74	2.1E-10	***2.25***	***1.50E-13***	receptor	skeltal defects/lethal	[123]	NA	Y
279	Thoc2	1.18	0.000011	1.07	7.40E-05	1.64	1.50E-08	***2.25***	***5.10E-12***	novel	unknown		NA	
