# Supplementary material for: Global Gene Expression Analysis of Murine Limb Development
Source: PLoS One. 2011 Dec 9;6(12):e28358. doi: 10.1371/journal.pone.0028358 (PMC3235105; doi:10.1371/journal.pone.0028358)
Supplement: Table S2 — Expression values for genes known to function during limb development, or family members of signal transduction pathways known to contribute to limb patterning. (PDF) [file pone.0028358.s005.pdf]

Table S2. Expression values for genes known to function during limb development, or family members of signal transduction pathways known to contribute to limb patterning. For each time point, raw expression values for forelimb (FL) and whole embryo (W) for each available embryonic day (E) are given along with the log2FC and adjusted p-value. Log2FC values >1 correspong to >2 fold up-regulation (grey highlighted genes), log2FC values < -1 correspond to >2 fold down-regulation (green highlighted genes. Genes highlighted in grey are upregulated in at least one developmental time point, genes with raw expression values <5 at all develomental time points are highlighted in yellow. *gene that is identified as being upregulated yet has a raw expression value <5; **genes that are both up-regulated and down-regulated at specific time points; ***genes with very low raw expression values, yet known to cause limb defects.

No.	Gene Symbol	FL9.5	WH9.5	log2FC	adjusted P-value	FL10.5	WH10.5	log2FC	adjusted P-value	FL11.5	WH11.5	log2FC	adjusted P-value	FL12.5	WH12.5	log2FC	adjusted P-value	FL13.5	WH13.5	log2FC	adjusted P-value
TGF-beta signaling																					
1	Tgfbr2	6.64	6.38	0.26	> 0.001	7.46	6.63	0.83	> 0.001	8.374	7.061	1.313	1.50E-14	9.145	7.507	1.638	6.34E-18	9.719	8.636	1.083	1.79E-12
2	Tgfbr3	4.78	4.87	-0.09	> 0.001	5.59	4.76	0.83	> 0.001	5.988	4.621	1.367	1.07E-07	6.576	4.804	1.772	2.05E-10	6.596	4.333	2.263	1.47E-13
3	Tgfb1i1	7.96	8.75	-0.79	> 0.001	7.36	8.58	-1.22	4.78E-06	8.239	8.181	0.058	> 0.001	8.763	9.304	-0.541	> 0.001	9.128	9.585	-0.457	> 0.001
4	Tgfbi	8.90	8.68	0.22	> 0.001	9.14	9.29	-0.15	> 0.001	10.313	9.813	0.5	> 0.001	10.78	10.605	0.175	> 0.001	11.203	10.526	0.677	> 0.001
5	Tgfbr1	9.65	9.53	0.12	> 0.001	10.21	9.39	0.82	> 0.001	10.198	9.503	0.695	> 0.001	10.03	9.491	0.539	> 0.001	9.89	8.929	0.961	> 0.001
6	Tgfb1	5.69	5.59	0.11	> 0.001	6.52	4.996	1.52	3.58E-08	6.444	5.014	1.43	9.59E-08	6.39	5.925	0.465	> 0.001	6.253	6.886	-0.633	> 0.001
7	Tgfb3	6.30	5.77	0.52	> 0.001	7.53	6.36	1.16	5.12E-07	8.263	7.118	1.145	1.18E-06	8.795	8.568	0.227	> 0.001	9.324	9.431	-0.107	> 0.001
Hedgehog signaling																					
8	Gli1	5.08	6.89	-1.81	4.23E-09	6.69	6.55	0.14	> 0.001	6.666	6.317	0.349	> 0.001	6.602	6.52	0.082	> 0.001	7.28	6.993	0.287	> 0.001
9	Ptch2	7.60	8.47	-0.87	> 0.001	7.90	8.69	-0.80	> 0.001	7.72	8.575	-0.855	> 0.001	7.372	8.527	-1.155	1.98E-06	8.147	8.201	-0.054	> 0.001
10	Ihh	4.80	4.78	0.02	> 0.001	4.40	4.27	0.13	> 0.001	5.095	4.593	0.502	> 0.001	5.671	4.892	0.779	> 0.001	5.936	5.886	0.05	> 0.001
11	Ptch1	9.36	10.92	-1.56	5.73E-12	9.80	10.76	-0.96	> 0.001	9.671	10.616	-0.945	> 0.001	9.773	10.832	-1.059	3.03E-09	10.13	10.673	-0.543	> 0.001
12	Gli3	10.70	9.85	0.85	> 0.001	10.15	9.96	0.19	> 0.001	9.787	9.852	-0.065	> 0.001	10.026	9.892	0.134	> 0.001	10.109	9.487	0.622	> 0.001
13	Gli2	9.86	9.07	0.80	> 0.001	9.83	9.15	0.68	> 0.001	9.95	8.857	1.093	4.10E-10	10.124	8.967	1.157	1.46E-11	9.916	9.046	0.87	> 0.001
14	Shh	5.94	9.21	-3.27	2.73E-13	8.46	9.17	-0.71	> 0.001	5.533	9.081	-3.548	2.61E-16	4.741	8.374	-3.633	7.05E-17	4.866	7.476	-2.61	1.56E-12
FGF signaling																					
15	Fgf9	6.14	6.16	-0.02	> 0.001	6.70	5.85	0.85	> 0.001	7.47	6.361	1.109	1.47E-09	7.591	6.869	0.722	> 0.001	6.887	6.751	0.136	> 0.001
16	Fgfrl1	8.74	7.84	0.90	> 0.001	8.83	7.83	1.00	1.80E-07	9.052	7.984	1.068	6.68E-08	9.018	8.28	0.738	> 0.001	8.752	8.513	0.239	> 0.001
17	Fgf18	4.40	5.30	-0.90	> 0.001	5.94	4.37	1.56	7.48E-05	7.305	4.125	3.18	1.60E-11	7.784	5.267	2.517	2.68E-09	7.385	6.17	1.215	9.62E-04
18	Fgfr3	6.39	7.75	-1.36	3.76E-06	5.98	8.05	-2.07	5.92E-12	7.324	8.025	-0.701	> 0.001	7.602	7.959	-0.357	> 0.001	8.265	8.268	-0.003	> 0.001
19	Fgf10	5.38	4.79	0.59	> 0.001	6.29	4.47	1.82	1.04E-09	5.824	4.112	1.712	7.74E-09	5.728	4.311	1.417	2.55E-07	5.173	4.279	0.894	> 0.001
20	Fgf11	4.15	4.10	0.05	> 0.001	3.98	3.99	0.00	> 0.001	4.454	4.522	-0.068	> 0.001	4.554	5.013	-0.459	> 0.001	5.143	5.059	0.084	> 0.001
21	Fgfr2	9.22	8.73	0.50	> 0.001	9.15	8.68	0.47	> 0.001	9.539	8.858	0.681	> 0.001	9.897	9.419	0.478	> 0.001	9.861	9.235	0.626	> 0.001
22	Fgf7	4.39	4.77	-0.38	> 0.001	4.34	4.68	-0.34	> 0.001	5.544	4.791	0.753	> 0.001	6.94	5.407	1.533	1.96E-11	7.612	6.695	0.917	> 0.001
23	Fgf6	4.30	4.25	0.05	> 0.001	3.87	4.49	-0.62	> 0.001	4.187	4.399	-0.212	> 0.001	4.453	4.535	-0.082	> 0.001	5.402	5.281	0.121	> 0.001
24	Fgfbp1	4.65	4.80	-0.15	> 0.001	5.16	4.88	0.28	> 0.001	5.725	4.41	1.315	2.39E-04	5.503	4.843	0.66	> 0.001	6.951	6.507	0.444	> 0.001
25	Fgf17	3.84	4.88	-1.04	> 0.001	4.18	3.84	0.34	> 0.001	3.879	3.819	0.06	> 0.001	3.709	3.852	-0.143	> 0.001	3.6	3.579	0.021	> 0.001
26	Fgfr1op	9.89	9.94	-0.05	> 0.001	9.59	9.58	0.01	> 0.001	9.318	9.514	-0.196	> 0.001	9.162	9.182	-0.02	> 0.001	8.767	8.738	0.029	> 0.001
27	Fgfr4	4.79	5.05	-0.27	> 0.001	4.65	4.85	-0.20	> 0.001	5.3	4.712	0.588	> 0.001	5.566	5.194	0.372	> 0.001	6.276	5.762	0.514	> 0.001
28	Fgf8	7.72	6.08	1.63	1.06E-05	9.06	4.90	4.16	1.01E-15	6.797	4.75	2.047	2.42E-07	5.835	4.516	1.319	2.86E-04	4.248	4.306	-0.058	> 0.001
29	Fgf13	7.58	7.78	-0.20	> 0.001	7.38	7.71	-0.33	> 0.001	8.114	8.39	-0.276	> 0.001	8.262	9.394	-1.132	5.08E-08	7.948	9.782	-1.834	9.91E-14
30	Fgf5	4.93	4.80	0.13	> 0.001	4.93	4.97	-0.04	> 0.001	4.238	5.313	-1.075	4.32E-04	4.267	5.184	-0.917	> 0.001	4.047	4.434	-0.387	> 0.001
31	Fgfbp3	5.02	7.96	-2.93	2.87E-08	5.50	7.76	-2.26	9.40E-08	5.803	7.969	-2.166	5.51E-07	5.645	8.656	-3.011	1.34E-10	5.518	8.114	-2.596	3.34E-09
32	Fgf12	4.55	4.43	0.12	> 0.001	4.37	4.15	0.22	> 0.001	4.66	4.756	-0.096	> 0.001	6.043	5.567	0.476	> 0.001	5.857	6.035	-0.178	> 0.001
33	Fgfr1	9.37	8.38	0.98	6.22E-05	9.68	8.10	1.58	5.04E-08	9.598	8.2	1.398	8.04E-07	9.509	9.1	0.409	> 0.001	9.665	9.26	0.405	> 0.001
34	Fgf15	4.92	7.52	-2.60	4.65E-10	4.32	7.48	-3.16	1.58E-14	4.791	7.664	-2.873	7.63E-13	4.809	7.752	-2.943	4.68E-14	4.155	6.739	-2.584	5.15E-12
35	Fgfr1op2	8.53	8.20	0.33	> 0.001	9.30	8.09	1.21	8.76E-18	9.285	7.798	1.487	2.33E-20	9.248	8.035	1.213	4.36E-18	9.552	8.274	1.278	6.79E-19
ROR signaling																					
36	Ror2	7.88	7.37	0.51	> 0.001	7.77	7.34	0.43	> 0.001	7.54	7.105	0.435	> 0.001	7.507	7.093	0.414	> 0.001	7.157	6.768	0.389	> 0.001
37	Ror1	7.37	6.87	0.51	> 0.001	7.93	6.75	1.18	5.66E-10	7.735	6.985	0.75	> 0.001	7.939	6.788	1.151	1.50E-09	7.722	6.688	1.034	1.15E-08
Transcription factors																					
38	Lbx1	7.07	5.38	1.69	1.41E-12	7.82	5.82	2.00	6.95E-17	6.952	5.707	1.245	1.52E-10	6.363	5.948	0.415	> 0.001	6.111	5.845	0.266	> 0.001
39	Sall2	9.33	9.85	-0.53	> 0.001	8.49	9.59	-1.10	8.34E-07	8.61	9.619	-1.009	4.32E-06	8.372	9.826	-1.454	1.25E-09	8.044	9.267	-1.223	6.45E-08
40	Lmx1a***	4.51	4.94	-0.43	> 0.001	4.99	4.62	0.37	> 0.001	4.88	4.382	0.498	> 0.001	4.197	4.145	0.052	> 0.001	4.164	3.971	0.193	> 0.001
41	Sall1	7.48	7.87	-0.38	> 0.001	8.08	7.31	0.77	> 0.001	7.933	7.319	0.614	> 0.001	7.42	7.163	0.257	> 0.001	6.526	6.379	0.147	> 0.001
42	Msx1	9.58	8.01	1.57	2.32E-09	9.79	7.84	1.96	5.85E-13	9.398	7.469	1.929	2.17E-12	9.619	7.672	1.947	8.00E-13	8.565	6.927	1.638	6.75E-11
43	Sox18	9.21	8.25	0.96	1.82E-05	9.16	8.45	0.72	> 0.001	9.009	8.4	0.609	> 0.001	9.189	8.755	0.434	> 0.001	8.888	8.955	-0.067	> 0.001
44	Pax9	5.06	5.53	-0.46	> 0.001	4.75	5.81	-1.06	8.85E-08	6.204	6.009	0.195	> 0.001	6.359	6.217	0.142	> 0.001	5.499	5.688	-0.189	> 0.001
45	Pax7	4.00	4.34	-0.34	> 0.001	4.25	4.49	-0.24	> 0.001	5.193	4.24	0.953	> 0.001	5.807	4.865	0.942	> 0.001	5.85	4.553	1.297	9.74E-07
46	Msx3	5.42	7.32	-1.91	5.65E-07	4.89	7.61	-2.72	8.50E-13	4.879	7.235	-2.356	1.13E-10	4.758	7.555	-2.797	3.36E-13	5.095	5.481	-0.386	> 0.001
47	Meis1	7.34	7.40	-0.06	> 0.001	5.68	8.18	-2.50	6.11E-13	4.937	7.981	-3.044	4.95E-15	4.757	6.02	-1.263	5.32E-06	5.584	5.631	-0.047	> 0.001
48	Sox5	6.21	5.93	0.28	> 0.001	6.97	6.57	0.41	> 0.001	7.162	6.771	0.391	> 0.001	7.444	7.407	0.037	> 0.001	6.318	7.132	-0.814	> 0.001
49	Pitx3	5.70	5.79	-0.10	> 0.001	5.59	5.40	0.20	> 0.001	6.625	5.806	0.819	> 0.001	6.944	6.277	0.667	> 0.001	6.381	6.239	0.142	> 0.001
50	Sox6	7.45	6.97	0.48	> 0.001	7.04	7.12	-0.08	> 0.001	8.115	7.623	0.492	> 0.001	8.497	8.161	0.336	> 0.001	8.279	8.485	-0.206	> 0.001
51	Myf5	5.97	6.95	-0.98	> 0.001	7.78	7.18	0.60	> 0.001	7.885	7.504	0.381	> 0.001	7.845	7.129	0.716	> 0.001	8.511	8.086	0.425	> 0.001
52	Sox11	11.92	11.48	0.43	> 0.001	11.11	12.03	-0.92	> 0.001	10.945	12.158	-1.213	1.41E-10	10.852	12.851	-1.999	1.26E-17	10.653	12.441	-1.788	3.31E-16
53	Sall4	10.43	10.23	0.20	> 0.001	8.77	9.62	-0.86	> 0.001	6.623	8.202	-1.579	2.55E-07	5.035	7.064	-2.029	9.29E-11	4.394	6.148	-1.754	3.58E-09
54	Sp5	6.36	7.05	-0.69	> 0.001	6.68	6.52	0.15	> 0.001	5.618	5.811	-0.193	> 0.001	5.527	6.193	-0.666	> 0.001	6.272	6.2	0.072	> 0.001
55	Sp4	5.93	6.06	-0.13	> 0.001	7.98	5.78	2.19	5.48E-07	7.74	5.746	1.994	2.99E-05	7.789	5.505	2.284	3.33E-07	7.2	4.814	2.386	1.17E-07
56	Meis3	7.03	7.14	-0.11	> 0.001	6.14	6.38	-0.23	> 0.001	5.525	6.228	-0.703	> 0.001	5.832	7.388	-1.556	3.64E-04	5.745	6.988	-1.243	> 0.001
57	Sox10	5.84	6.89	-1.05	> 0.001	5.36	6.44	-1.08	9.69E-05	5.903	6.322	-0.419	> 0.001	6.27	7.045	-0.775	> 0.001	6.512	6.794	-0.282	> 0.001
58	Sp6	7.66	6.24	1.42	3.07E-10	6.14	5.59	0.55	> 0.001	5.68	5.42	0.26	> 0.001	5.252	5.641	-0.389	> 0.001	5.042	5.436	-0.394	> 0.001
59	Sox17	6.90	6.42	0.48	> 0.001	6.88	6.53	0.35	> 0.001	6.295	6.079	0.216	> 0.001	6.513	6.245	0.268	> 0.001	6.615	6.471	0.144	> 0.001
60	Runx2	4.46	4.07	0.38	> 0.001	6.20	4.19	2.01	1.07E-10	7.003	5.334	1.669	3.18E-08	7.189	6.45	0.739	> 0.001	7.397	7.033	0.364	> 0.001
61	Meox2	7.36	7.46	-0.10	> 0.001	9.27	7.71	1.56	6.98E-11	10.198	8.228	1.97	1.00E-13	9.965	8.239	1.726	2.32E-12	10.116	8.991	1.125	1.39E-07
62	Dlx1	6.27	6.78	-0.52	> 0.001	6.83	7.05	-0.22	> 0.001	6.678	7.438	-0.76	> 0.001	6.402	7.505	-1.103	4.79E-06	5.068	7.17	-2.102	8.26E-13
63	Sox9	7.99	8.07	-0.08	> 0.001	9.41	8.68	0.73	> 0.001	9.844	9.611	0.233	> 0.001	9.841	9.97	-0.129	> 0.001	9.438	9.482	-0.044	> 0.001
64	Meox1	8.82	9.48	-0.66	> 0.001	7.78	8.84	-1.06	8.78E-04	7.771	7.669	0.102	> 0.001	8.216	7.984	0.232	> 0.001	8.371	7.669	0.702	> 0.001
65	Pitx2	8.50	8.65	-0.16	> 0.001	9.61	8.82	0.80	> 0.001	9.908	9.032	0.876	> 0.001	9.661	9.586	0.075	> 0.001	9.003	9.128	-0.125	> 0.001
66	En1*	4.23	3.87	0.36	> 0.001	4.54	3.51	1.03	2.87E-04	4.592	3.084	1.508	3.61E-07	4.878	4.164	0.714	> 0.001	4.707	4.574	0.133	> 0.001
67	En2	3.68	6.77	-3.10	4.54E-21	3.46	6.20	-2.74	2.70E-21	3.339	5.79	-2.451	2.83E-19	3.319	6.24	-2.921	2.13E-22	3.362	5.67	-2.308	5.22E-19
68	Jag2	7.70	6.27	1.43	9.09E-09	7.02	6.27	0.75	> 0.001	6.903	6.446	0.457	> 0.001	6.866	6.99	-0.124	> 0.001	6.885	7.147	-0.262	> 0.001
69	Sp3	9.07	8.70	0.37	> 0.001	10.22	8.72	1.51	2.07E-10	10.34	9.09	1.25	4.05E-08	10.345	9.349	0.996	3.45E-07	10.526	9.428	1.098	3.19E-07
70	Sp8	8.78	7.95	0.83	> 0.001	7.23	7.60	-0.37	> 0.001	6.202	7.559	-1.357	1.40E-06	5.339	6.92	-1.581	5.93E-08	4.384	6.104	-1.72	4.47E-09
71	Sox2	3.62	10.95	-7.33	1.09E-10	3.57	10.85	-7.29	1.23E-12	4.126	10.57	-6.444	8.51E-11	4.582	10.806	-6.224	9.71E-11	4.316	9.8	-5.484	2.10E-09
72	Sall3	7.50	7.27	0.23	> 0.001	7.34	6.98	0.37	> 0.001	5.937	6.922	-0.985	> 0.001	5.284	6.861	-1.577	2.92E-09	5.076	6.159	-1.083	6.68E-06
73	Jag1**	6.13	7.41	-1.29	2.62E-06	7.83	6.59	1.24	3.30E-07	8.373	6.865	1.508	3.54E-09	7.979	7.055	0.924	> 0.001	8.301	7.408	0.893	> 0.001
74	Meis2	9.99	9.32	0.67	> 0.001	9.44	9.43	0.01	> 0.001	8.61	9.208	-0.598	> 0.001	8.655	9.325	-0.67	> 0.001	8.391	8.76	-0.369	> 0.001
75	Pax1	5.35	6.45	-1.10	> 0.001	6.16	6.65	-0.49	> 0.001	6.264	6.963	-0.699	> 0.001	5.506	8.055	-2.549	4.05E-11	5.495	7.946	-2.451	1.82E-10
76	Myf6	4.22	4.59	-0.38	> 0.001	3.80	5.55	-1.75	7.15E-07	3.907	4.731	-0.824	> 0.001	3.881	4.719	-0.838	> 0.001	5.06	5.439	-0.379	> 0.001

77	Dlx2	7.73	6.87	0.86	1.86E-04	6.80	6.87	-0.07	> 0.001	6.655	6.558	0.097	> 0.001	6.352	7.59	-1.238	6.45E-07	5.869	7.162	-1.293	1.61E-07
78	Sp1	10.25	10.13	0.12	> 0.001	10.50	10.16	0.34	> 0.001	10.414	9.872	0.542	> 0.001	10.602	9.875	0.727	> 0.001	10.898	9.976	0.922	> 0.001
79	Sp7	5.44	5.13	0.31	> 0.001	4.87	5.13	-0.26	> 0.001	5.16	5.21	-0.05	> 0.001	5.215	5.911	-0.696	> 0.001	5.935	6.668	-0.733	> 0.001
80	Pax3	7.27	8.09	-0.83	> 0.001	7.74	8.17	-0.43	> 0.001	5.496	7.904	-2.408	2.00E-14	5.044	7.467	-2.423	1.06E-14	5.004	6.318	-1.314	7.54E-08
81	Runx3	5.14	5.06	0.08	> 0.001	4.73	5.01	-0.28	> 0.001	5.411	5.093	0.318	> 0.001	5.822	5.579	0.243	> 0.001	6.01	5.481	0.529	> 0.001
82	Dlx5	6.86	6.99	-0.12	> 0.001	7.25	6.73	0.52	> 0.001	8.717	6.656	2.061	1.49E-11	8.705	7.398	1.307	4.13E-07	7.792	7.519	0.273	> 0.001
83	Myog	5.24	5.97	-0.72	> 0.001	6.26	7.03	-0.78	> 0.001	10.023	7.651	2.372	5.42E-04	10.668	9.475	1.193	> 0.001	11.304	10.153	1.151	> 0.001
84	Evx1	5.49	6.51	-1.03	> 0.001	6.44	5.67	0.77	> 0.001	6.285	5.649	0.636	> 0.001	5.393	5.827	-0.434	> 0.001	5.235	4.907	0.328	> 0.001
85	Sox13	8.33	7.99	0.34	> 0.001	7.51	7.26	0.25	> 0.001	6.703	6.813	-0.11	> 0.001	6.478	7.162	-0.684	> 0.001	6.749	6.999	-0.25	> 0.001
86	Sox21	4.28	6.90	-2.61	4.80E-09	4.13	6.80	-2.67	1.20E-11	4.281	6.501	-2.22	4.05E-09	4.548	6.807	-2.259	2.29E-09	4.352	5.627	-1.275	6.48E-05
87	Pax6	4.20	5.99	-1.79	3.31E-07	3.91	6.03	-2.12	1.05E-10	3.95	5.601	-1.651	8.15E-08	3.869	5.368	-1.499	3.17E-07	3.987	4.657	-0.67	> 0.001
88	Sox8	3.47	3.43	0.05	> 0.001	5.46	3.23	2.23	3.91E-11	5.338	3.368	1.97	3.18E-09	6.082	3.362	2.72	1.11E-13	6.245	3.755	2.49	1.17E-12
89	Sp2	7.95	7.45	0.50	> 0.001	7.38	6.99	0.40	> 0.001	7.33	6.765	0.565	> 0.001	6.975	7.653	-0.678	> 0.001	6.888	7.874	-0.986	> 0.001
90	Msx2	8.67	7.14	1.54	3.10E-10	8.57	6.77	1.79	3.09E-13	8.409	6.693	1.716	1.93E-12	8.28	6.766	1.514	3.19E-11	6.517	6.031	0.486	> 0.001
91	Sox4	9.57	8.98	0.60	> 0.001	10.42	9.36	1.07	1.82E-07	10.498	9.528	0.97	1.85E-06	10.563	10.52	0.043	> 0.001	10.536	10.303	0.233	> 0.001
92	Pitx1	5.64	6.98	-1.34	> 0.001	4.82	6.63	-1.81	1.52E-07	5.291	6.641	-1.35	3.97E-05	5.34	7.754	-2.414	6.19E-11	5.149	7.458	-2.309	1.84E-10
93	Lbx2	5.18	4.78	0.39	> 0.001	4.74	4.75	-0.02	> 0.001	4.588	4.799	-0.211	> 0.001	4.431	5.136	-0.705	> 0.001	4.675	4.611	0.064	> 0.001

Homeobox transcription factors

94	Hoxa2	6.81	7.27	-0.46	> 0.001	6.27	7.37	-1.10	1.52E-04	5.987	6.946	-0.959	> 0.001	5.835	6.983	-1.148	5.80E-05	5.823	5.966	-0.143	> 0.001
95	Hoxb8	6.26	5.90	0.36	> 0.001	5.82	5.88	-0.06	> 0.001	4.78	6.131	-1.351	9.08E-04	4.56	7.096	-2.536	1.29E-07	4.529	5.619	-1.09	> 0.001
96	Hoxa5**	9.82	8.76	1.06	9.56E-05	8.76	9.06	-0.29	> 0.001	8.18	9.16	-0.98	> 0.001	8.05	9.163	-1.113	1.31E-06	8.171	8.654	-0.483	> 0.001
97	Hoxc13	4.64	4.84	-0.20	> 0.001	5.32	5.01	0.31	> 0.001	6.088	5.435	0.653	> 0.001	5.865	6.308	-0.443	> 0.001	5.296	5.795	-0.499	> 0.001
98	Hoxd13	6.69	6.54	0.15	> 0.001	9.73	6.47	3.26	2.41E-17	11.206	7.988	3.218	3.32E-17	11.203	8.287	2.916	3.21E-16	10.108	7.294	2.814	6.78E-16
99	Hoxb5**	10.29	8.28	2.02	2.25E-05	7.52	8.48	-0.97	> 0.001	6.097	9.09	-2.993	3.98E-09	6.128	9.666	-3.538	6.33E-12	6.077	9.121	-3.044	3.58E-10
100	Hoxc6	7.71	6.86	0.86	> 0.001	7.64	6.87	0.78	> 0.001	7.603	6.67	0.933	> 0.001	7.548	7.132	0.416	> 0.001	6.824	6.42	0.404	> 0.001
101	Hoxc5	9.45	7.36	2.09	8.53E-07	8.34	7.69	0.65	> 0.001	8.695	7.699	0.996	> 0.001	9.217	7.609	1.608	1.15E-05	8.106	6.534	1.572	1.31E-05
102	Hoxc4	9.72	8.52	1.20	> 0.001	8.33	9.05	-0.72	> 0.001	8.21	8.881	-0.671	> 0.001	8.475	9.127	-0.652	> 0.001	8.31	8.271	0.039	> 0.001
103	Hoxb3	8.40	7.44	0.96	> 0.001	7.94	7.84	0.10	> 0.001	7.036	7.962	-0.926	> 0.001	6.825	8.207	-1.382	2.23E-05	7.13	7.766	-0.636	> 0.001
104	Hoxa4**	6.14	5.11	1.03	5.06E-04	4.92	4.57	0.35	> 0.001	3.92	4.618	-0.698	> 0.001	3.915	4.943	-1.028	8.47E-04	4.1	4.294	-0.194	> 0.001
105	Hoxb6**	9.91	8.15	1.76	9.00E-06	7.46	8.15	-0.69	> 0.001	5.931	8.009	-2.078	4.24E-08	5.985	8.236	-2.251	1.88E-09	5.721	7.889	-2.168	7.23E-09
106	Hoxa3	7.41	6.75	0.67	> 0.001	6.61	6.43	0.17	> 0.001	6.303	6.423	-0.12	> 0.001	6.215	6.896	-0.681	> 0.001	6.246	6.225	0.021	> 0.001
107	Hoxd10	10.27	8.16	2.11	3.63E-19	11.31	8.36	2.94	3.49E-26	10.923	8.604	2.319	3.02E-22	10.623	7.947	2.676	4.75E-25	9.932	7.819	2.113	1.09E-21
108	Hoxb1	8.28	8.48	-0.21	> 0.001	3.85	6.54	-2.69	6.74E-09	3.45	4.658	-1.208	> 0.001	3.455	3.848	-0.393	> 0.001	3.336	3.346	-0.01	> 0.001
109	Hoxa11	8.29	6.53	1.76	5.96E-12	10.26	6.88	3.38	1.78E-22	9.791	6.786	3.005	1.66E-20	9.393	7.369	2.024	3.24E-15	8.407	6.59	1.817	6.83E-14
110	Hoxc9	6.87	7.36	-0.49	> 0.001	6.45	7.22	-0.77	> 0.001	6.023	7.094	-1.071	4.51E-09	5.747	7.094	-1.347	1.03E-11	6.125	6.565	-0.44	> 0.001
111	Hoxd12	4.24	3.90	0.34	> 0.001	8.13	3.59	4.54	4.14E-19	9.056	3.68	5.376	2.72E-21	8.902	4.161	4.741	6.05E-20	6.589	3.732	2.857	4.55E-13
112	Hoxa10	8.75	7.77	0.98	> 0.001	10.41	8.03	2.38	1.19E-15	10.566	8.754	1.812	1.09E-11	10.363	9.097	1.266	8.11E-09	9.826	7.978	1.848	2.06E-12
113	Hoxd4	5.62	5.49	0.13	> 0.001	7.06	5.33	1.73	1.43E-08	5.616	5.153	0.463	> 0.001	5.719	4.899	0.82	> 0.001	5.629	4.695	0.934	> 0.001
114	Hoxa7	8.66	7.98	0.69	> 0.001	8.38	7.61	0.78	> 0.001	7.644	7.812	-0.168	> 0.001	7.555	7.651	-0.096	> 0.001	6.936	7.085	-0.149	> 0.001
115	Hoxb13***	4.59	4.17	0.42	> 0.001	4.52	5.03	-0.50	> 0.001	4.655	5.479	-0.824	> 0.001	4.319	4.965	-0.646	> 0.001	4.201	5.04	-0.839	> 0.001
116	Hoxd9	11.88	8.25	3.64	1.66E-20	11.20	8.61	2.59	2.03E-17	10.305	8.196	2.109	1.51E-14	9.802	8.476	1.326	1.63E-09	8.878	8.056	0.822	> 0.001
117	Hoxa11as	5.93	5.57	0.36	> 0.001	8.34	5.77	2.58	1.37E-19	8.892	5.904	2.988	2.72E-21	8.534	6.076	2.458	5.73E-19	7.7	5.659	2.041	2.03E-16
118	Hoxb4	7.06	6.49	0.57	> 0.001	6.60	6.28	0.32	> 0.001	6.064	6.239	-0.175	> 0.001	6.345	6.161	0.184	> 0.001	5.985	5.469	0.516	> 0.001
119	Hoxa9	11.24	9.25	1.99	1.65E-19	11.24	9.91	1.33	9.12E-16	10.416	9.8	0.616	> 0.001	10.258	9.65	0.608	> 0.001	10.147	8.788	1.359	2.74E-16
120	Hoxb9	5.89	5.56	0.33	> 0.001	4.81	4.94	-0.13	> 0.001	4.375	5.131	-0.756	> 0.001	4.669	5.501	-0.832	> 0.001	4.702	4.917	-0.215	> 0.001
121	Hoxa13	4.94	4.95	-0.02	> 0.001	7.25	4.68	2.57	7.05E-08	8.451	4.417	4.034	2.22E-13	8.319	4.763	3.556	1.98E-12	6.622	4.501	2.121	6.69E-07
122	Hoxd8	10.28	7.88	2.40	4.62E-15	10.13	7.75	2.38	5.07E-17	9.309	7.078	2.231	8.77E-16	9.417	7.782	1.635	1.24E-12	9.704	7.601	2.103	6.94E-16
123	Hoxd11	4.73	4.28	0.45	> 0.001	9.23	4.48	4.75	1.21E-28	9.024	4.692	4.332	6.59E-27	8.744	4.783	3.961	3.06E-26	7.233	4.39	2.843	1.34E-21
124	Hoxc10	3.97	8.46	-4.49	2.10E-12	5.21	7.61	-2.41	5.88E-08	5.266	8.281	-3.015	2.27E-09	4.71	9.321	-4.611	4.06E-16	3.903	8.006	-4.103	1.43E-14
125	Hoxa1	10.39	9.75	0.65	> 0.001	6.97	8.62	-1.64	7.23E-08	6.166	7.772	-1.606	5.34E-07	6.504	6.752	-0.248	> 0.001	6.112	6.631	-0.519	> 0.001
126	Hoxb2	7.99	7.81	0.17	> 0.001	7.74	7.44	0.29	> 0.001	7.19	7.211	-0.021	> 0.001	7.276	7.396	-0.12	> 0.001	7.078	6.697	0.381	> 0.001
127	Hoxd1	7.20	6.17	1.03	> 0.001	4.21	5.03	-0.82	> 0.001	3.886	4.722	-0.836	> 0.001	5.048	4.735	0.313	> 0.001	4.259	4.411	-0.152	> 0.001

BMP signaling

128	Grem2	4.77	5.08	-0.31	> 0.001	4.57	6.18	-1.61	7.32E-09	6.621	6.901	-0.28	> 0.001	7.263	6.727	0.536	> 0.001	6.868	6.82	0.048	> 0.001
129	Bambi	10.94	9.47	1.47	5.80E-14	9.65	8.98	0.68	> 0.001	9.826	8.724	1.102	1.07E-11	9.672	8.823	0.849	> 0.001	8.774	8.641	0.133	> 0.001
130	Bmp6	4.75	5.58	-0.83	> 0.001	4.76	6.04	-1.28	2.30E-08	5.258	6.052	-0.794	> 0.001	5.759	6.36	-0.601	> 0.001	5.606	7.178	-1.572	1.13E-10
131	Bmp4	10.13	8.96	1.17	9.09E-09	10.82	8.67	2.15	1.09E-17	10.597	8.361	2.236	7.70E-18	10.339	8.73	1.609	7.30E-14	9.239	8.404	0.835	> 0.001
132	Grem1	6.19	5.57	0.61	> 0.001	9.19	5.36	3.84	1.92E-12	7.536	5.401	2.135	2.81E-05	8.045	4.967	3.078	7.39E-10	7.071	5.183	1.888	1.55E-05
133	Nog	4.47	4.39	0.09	> 0.001	4.10	4.18	-0.08	> 0.001	6.249	4.405	1.844	2.53E-08	7.912	4.863	3.049	1.90E-14	6.329	4.959	1.37	5.56E-06
134	Bmp2	6.87	7.17	-0.31	> 0.001	6.91	6.12	0.79	> 0.001	8.665	6.784	1.881	1.21E-10	9.121	6.973	2.148	1.41E-12	8.614	7.753	0.861	> 0.001
135	Gdf3	4.94	5.20	-0.27	> 0.001	4.41	5.10	-0.69	> 0.001	4.368	5.318	-0.95	> 0.001	4.298	5.714	-1.416	3.05E-10	4.499	5.227	-0.728	> 0.001
136	Bmp1	7.29	6.57	0.72	> 0.001	7.87	6.68	1.19	2.25E-11	7.96	7.25	0.71	> 0.001	7.99	7.705	0.285	> 0.001	8.053	7.71	0.343	> 0.001
137	Bmp7	7.69	5.66	2.03	3.03E-12	8.27	5.52	2.75	3.17E-17	8.081	5.209	2.872	1.48E-17	8.231	5.814	2.417	1.57E-15	7.074	6.195	0.879	> 0.001
138	Gdf10	5.91	6.88	-0.97	> 0.001	6.35	7.05	-0.70	> 0.001	8.921	8.707	0.214	> 0.001	9.778	9.49	0.288	> 0.001	10.811	10.505	0.306	> 0.001
139	Bmp5	7.64	7.64	0.00	> 0.001	6.27	7.30	-1.03	1.70E-05	7.154	7.648	-0.494	> 0.001	7.795	8.105	-0.31	> 0.001	7.836	8.274	-0.438	> 0.001
140	Bmper	3.85	6.26	-2.42	1.56E-09	5.20	6.24	-1.04	1.55E-04	8.059	7.651	0.408	> 0.001	8.588	7.979	0.609	> 0.001	8.67	8.679	-0.009	> 0.001
141	Gdf2	5.75	6.06	-0.31	> 0.001	5.10	6.15	-1.04	1.12E-09	5.176	6.076	-0.9	> 0.001	4.963	6.36	-1.397	4.58E-13	5.55	6.936	-1.386	5.79E-13
142	Gdf5	4.82	4.53	0.29	> 0.001	5.88	4.84	1.04	> 0.001	8.573	4.881	3.692	9.72E-17	9.442	6.338	3.104	8.41E-15	8.72	6.347	2.373	1.88E-11
143	Bmpr1b	6.30	6.04	0.26	> 0.001	6.79	6.82	-0.03	> 0.001	7.523	7.181	0.342	> 0.001	7.65	7.231	0.419	> 0.001	7.078	6.653	0.425	> 0.001
144	Bmpr2	9.17	9.22	-0.05	> 0.001	9.49	9.59	-0.10	> 0.001	9.853	10.102	-0.249	> 0.001	9.85	10.305	-0.455	> 0.001	10.077	10.312	-0.235	> 0.001

WNT signaling

145	Wnt1	4.38	6.22	-1.85	3.76E-05	4.01	5.41	-1.40	8.86E-06	3.949	5.025	-1.076	7.39E-04	3.929	5.09	-1.161	1.77E-04	4.165	4.285	-0.12	> 0.001
146	Lrp5	4.18	4.02	0.16	> 0.001	5.54	3.69	1.85	1.30E-11	5.539	3.6	1.939	4.38E-12	5.329	4.155	1.174	4.54E-07	4.993	4.141	0.852	> 0.001
147	Dkk2	5.79	5.60	0.19	> 0.001	6.50	5.96	0.54	> 0.001	8.421	6.535	1.886	2.20E-08	9.407	7.82	1.587	2.44E-07	10.069	7.589	2.48	1.62E-11
148	Tcf7l2	6.88	6.73	0.15	> 0.001	8.10	7.23	0.87	> 0.001	8.206	7.463	0.743	> 0.001	8.227	8.215	0.012	> 0.001	8.556	8.368	0.188	> 0.001
149	Wnt11	7.05	6.03	1.02	5.77E-08	7.31	6.29	1.02	2.76E-09	8.687	6.573	2.114	3.26E-18	9.062	7.482	1.58	1.13E-14	8.122	7.19	0.932	> 0.001
150	Dkk3	5.61	6.40	-0.79	> 0.001	5.97	6.45	-0.49	> 0.001	6.756	6.637	0.119	> 0.001	7.424	7.305	0.119	> 0.001	7.849	8.064	-0.215	> 0.001
151	Fzd9	5.40	6.17	-0.77	> 0.001	4.95	5.82	-0.86	> 0.001	6.403	5.995	0.408	> 0.001	6.749	6.854	-0.105	> 0.001	7.466	7.735	-0.269	> 0.001
152	Lrp4	6.44	6.01	0.43	> 0.001	7.04	5.26	1.78	3.35E-12	6.688	5.368	1.32	6.69E-09	6.868	5.507	1.361	9.85E-10	6.957	6.07	0.887	> 0.001
153	Tcf25	8.61	8.27	0.34	> 0.001	8.81	8.24	0.57	> 0.001	9.059	8.239	0.82	> 0.001	9.135	8.964	0.171	> 0.001	9.409	8.997	0.412	> 0.001
154	Fzd4	7.72	8.06	-0.34	> 0.001	6.69	8.17	-1.48	2.54E-04	7.005	7.829	-0.824	> 0.001	7.816	8.126	-0.31	> 0.001	8.2	8.232	-0.032	> 0.001
155	Fzd6	7.69	7.03	0.66	> 0.001	7.80	7.27	0.54	> 0.001	7.82	7.297	0.523	> 0.001	8.052	7.309	0.743	> 0.001	8.336	7.775	0.561	> 0.001
156	Wnt6	8.77	6.86	1.92	3.32E-12	7.15	6.11	1.05	5.60E-07	6.961	6.201	0.76	> 0.001	6.886	6.631	0.255	> 0.001	6.556	6.646	-0.09	> 0.001
157	Wnt3a	5.11	6.36	-1.24	1.23E-06	4.55	5.86	-1.30	3.89E-09	4.967	5.66	-0.693	> 0.001	5.143	5.72	-0.577	> 0.001	5.135	5.342	-0.207	> 0.001
158	Wnt7b	7.10	7.10	0.00	> 0.001	7.07	7.48	-0.41	> 0.001	7.449	7.974	-0.525	> 0.001	7.547	8.198	-0.651	> 0.001	7.279	7.838	-0.559	> 0.001
159	Sost	4.97	4.69	0.28	> 0.001	6.64	4.96	1.68	8.97E-12	5.443	4.459	0.984	> 0.001	4.41	4.58	-0.17	> 0.001	3.865	4.118	-0.253	> 0.001
160	Wnt9a	4.46	5.14	-0.67	> 0.001	4.83	5.56	-0.73	> 0.001	5.492	5.681	-0.189	> 0.001	5.974	5.932	0.042	> 0.001	5.913	5.952	-0.039	> 0.001
161	Fzd10	7.10	7.16	-0.06	> 0.001	7.94	7.14	0.80	> 0.001	7.675	7.275	0.4	> 0.001	7.4	6.922	0.478	> 0.001	7.205	6.231	0.974	> 0.001
162	Wnt16	4.29	4.82	-0.53	> 0.001	4.99	4.44	0.56	> 0.001	4.555	4.301	0.254	> 0.001	4.586	4.253	0.333	> 0.001	4.737	3.969	0.768	> 0.001
163	Tcf3	9.84	8.92	0.92	1.44E-04	10.23	8.29	1.94	8.37E-10	10.135	8.091	2.044	3.10E-10	9.936	9.159	0.777	> 0.001	9.161	8.726	0.435	> 0.001
164	Lrp8	6.72	6.62	0.11	> 0.001	6.64	6.72	-0.07	> 0.001	6.13	6.882	-0.752	> 0.001	5.936	7.037	-1.101	3.58E-10	5.721	7.163	-1.442	1.37E-13
165	Wnt5b	7.12	6.61	0.51	> 0.001	6.78	6.45	0.34	> 0.001	6.556	5.972	0.584	> 0.001	6.647	6.631	0.016	> 0.001	5.884	5.909	-0.025	> 0.001

166	Wnt5a	8.98	8.49	0.49	> 0.001	9.68	8.23	1.45	4.37E-11	10.327	8.627	1.7	1.13E-12	10.202	8.993	1.209	1.23E-09	9.974	9.007	0.967	> 0.001
167	Fzd5	5.53	6.99	-1.46	1.66E-09	4.76	6.78	-2.01	1.50E-15	5.53	6.94	-1.41	1.77E-10	5.821	7.576	-1.755	1.68E-13	5.877	8.068	-2.191	8.55E-17
168	Fzd1	9.69	8.68	1.01	8.12E-11	9.56	9.00	0.56	> 0.001	9.783	9.408	0.375	> 0.001	9.686	9.971	-0.285	> 0.001	9.453	9.713	-0.26	> 0.001
169	Lrp6**	5.86	5.76	0.10	> 0.001	7.69	5.95	1.74	8.09E-07	7.444	5.864	1.58	1.12E-05	7.801	5.676	2.125	6.97E-09	7.504	5.433	2.071	2.15E-08
170	Wnt4	8.21	7.90	0.31	> 0.001	7.59	7.39	0.20	> 0.001	8.079	7.251	0.828	> 0.001	8.458	8.212	0.246	> 0.001	8.399	8.162	0.237	> 0.001
171	Lrp10	8.69	7.90	0.79	1.63E-04	8.24	7.73	0.51	> 0.001	8.364	7.383	0.981	1.71E-05	8.314	8.416	-0.102	> 0.001	8.694	9.111	-0.417	> 0.001
172	Lrp1	7.81	7.41	0.41	> 0.001	7.75	7.53	0.22	> 0.001	8.135	7.798	0.337	> 0.001	8.128	8.086	0.042	> 0.001	8.954	8.652	0.302	> 0.001
173	Tcf21	4.40	6.16	-1.77	> 0.001	4.26	6.24	-1.98	5.20E-04	3.736	6.358	-2.622	9.00E-05	3.679	7.052	-3.373	9.31E-07	3.467	7.946	-4.479	2.05E-09
174	Lef1	7.81	5.47	2.33	6.01E-10	8.00	4.69	3.31	4.10E-15	7.636	4.695	2.941	3.15E-13	7.648	5.49	2.158	2.39E-10	6.854	4.87	1.984	5.42E-09
175	Tcf15	8.47	8.68	-0.21	> 0.001	9.14	8.61	0.53	> 0.001	7.878	8.086	-0.208	> 0.001	7.838	8.342	-0.504	> 0.001	7.81	7.61	0.2	> 0.001
176	Lrp12	8.27	7.81	0.45	> 0.001	9.15	8.04	1.11	9.67E-13	9.319	8.439	0.88	> 0.001	9.311	8.471	0.84	> 0.001	8.942	8.161	0.781	> 0.001
177	Lrp2	4.14	6.50	-2.36	1.10E-18	3.27	5.32	-2.05	8.33E-19	3.254	4.767	-1.513	3.13E-14	3.338	4.263	-0.925	> 0.001	3.163	3.622	-0.459	> 0.001
178	Lrp11	7.16	7.02	0.14	> 0.001	5.51	6.81	-1.30	1.64E-07	4.816	7.148	-2.332	1.86E-13	5.203	7.166	-1.963	3.06E-11	4.692	7.524	-2.832	1.50E-16
179	Tcf7	6.76	5.83	0.93	1.34E-04	7.08	5.20	1.88	2.16E-10	6.115	4.939	1.176	6.97E-06	5.58	5.551	0.029	> 0.001	5.485	5.147	0.338	> 0.001
180	Tcf4	9.64	8.97	0.67	> 0.001	10.42	9.25	1.18	6.18E-11	10.918	9.515	1.403	1.47E-12	10.979	10.291	0.688	> 0.001	11.165	10.517	0.648	> 0.001
181	Fzd2	11.00	10.40	0.60	> 0.001	10.89	10.23	0.66	> 0.001	10.958	10.354	0.604	> 0.001	11.032	11.251	-0.219	> 0.001	10.917	10.903	0.014	> 0.001
182	Wnt7a	8.99	7.09	1.90	1.29E-11	7.09	7.29	-0.20	> 0.001	6.688	7.268	-0.58	> 0.001	6.703	7.431	-0.728	> 0.001	5.806	6.89	-1.084	2.12E-07
183	Tcf19	7.65	7.46	0.19	> 0.001	8.39	7.72	0.67	> 0.001	8.788	7.658	1.13	6.00E-11	8.836	7.985	0.851	> 0.001	8.751	8.536	0.215	> 0.001
184	Dkk4	4.61	4.33	0.28	> 0.001	4.50	4.18	0.32	> 0.001	4.296	4.325	-0.029	> 0.001	4.252	4.301	-0.049	> 0.001	5.542	4.827	0.715	> 0.001
185	Dkk1	8.01	7.43	0.58	> 0.001	8.47	7.33	1.14	8.81E-09	7.529	7.105	0.424	> 0.001	7.158	7.078	0.08	> 0.001	7.03	6.874	0.156	> 0.001
186	Tcf12	11.10	10.54	0.56	> 0.001	11.93	10.48	1.45	2.51E-17	11.787	10.536	1.251	6.43E-15	11.768	10.775	0.993	8.18E-13	11.651	10.393	1.258	1.59E-15
187	Fzd7	9.64	9.54	0.10	> 0.001	9.07	8.99	0.08	> 0.001	9.054	8.535	0.519	> 0.001	9.032	8.612	0.42	> 0.001	8.887	8.24	0.647	> 0.001
188	Fzd3	8.45	8.87	-0.42	> 0.001	8.65	8.86	-0.21	> 0.001	8.287	8.714	-0.427	> 0.001	8.215	8.3	-0.085	> 0.001	7.611	7.74	-0.129	> 0.001
189	Wnt2	8.62	7.71	0.91	> 0.001	5.77	7.58	-1.81	2.30E-07	5.901	6.927	-1.026	> 0.001	6.473	6.835	-0.362	> 0.001	6.091	6.846	-0.755	> 0.001

T-box genes

190	Tbx3	7.88	6.08	1.80	8.03E-11	8.37	6.28	2.09	1.02E-14	8.099	6.323	1.776	2.96E-12	7.983	6.151	1.832	2.29E-13	7.767	5.665	2.102	6.49E-15
191	Tbx15	7.09	4.77	2.32	2.28E-15	10.02	6.18	3.84	4.68E-25	10.157	7.266	2.891	2.11E-20	9.863	7.92	1.943	2.31E-15	9.184	7.818	1.366	6.16E-11
192	Tbx5	8.21	6.43	1.78	2.89E-08	9.11	6.65	2.46	6.27E-14	9.597	6.74	2.857	3.91E-15	9.764	6.73	3.034	7.45E-17	8.983	6.423	2.56	1.34E-14
193	Tbx19	4.73	4.94	-0.21	> 0.001	4.19	5.11	-0.91	> 0.001	4.267	4.716	-0.449	> 0.001	4.025	4.857	-0.832	> 0.001	4.337	4.347	-0.01	> 0.001
194	Tbx2	7.71	6.12	1.59	6.98E-07	7.98	7.07	0.91	> 0.001	7.994	7.442	0.552	> 0.001	7.525	8.163	-0.638	> 0.001	6.853	7.48	-0.627	> 0.001
195	Tbx4	4.57	6.41	-1.83	2.08E-12	6.93	6.66	0.27	> 0.001	6.975	7.114	-0.139	> 0.001	7.058	7.29	-0.232	> 0.001	6.161	6.829	-0.668	> 0.001
196	Tbx6***	4.20	5.57	-1.37	3.62E-04	3.99	4.38	-0.39	> 0.001	4.007	4.126	-0.119	> 0.001	3.966	4.023	-0.057	> 0.001	4.075	4.21	-0.135	> 0.001
197	Tbx22***	3.44	3.65	-0.20	> 0.001	3.60	3.79	-0.19	> 0.001	4.396	4.594	-0.198	> 0.001	3.705	3.997	-0.292	> 0.001	3.572	3.828	-0.256	> 0.001
198	Tbx20	4.42	6.91	-2.49	7.55E-08	3.42	6.33	-2.91	3.41E-11	3.432	6.162	-2.73	1.21E-09	3.291	6.714	-3.423	2.37E-11	3.437	6.257	-2.82	1.06E-10
199	Tbx1	5.15	7.10	-1.95	8.05E-07	5.67	6.86	-1.19	1.12E-05	6.823	6.388	0.435	> 0.001	7.063	6.569	0.494	> 0.001	6.716	6.311	0.405	> 0.001
200	Tbx18	8.13	7.20	0.93	> 0.001	10.15	7.44	2.71	2.60E-19	10.678	8.187	2.491	7.64E-18	10.567	8.336	2.231	7.73E-17	10.284	7.869	2.415	5.22E-18
