# Supplementary material for: Global Gene Expression Analysis of Murine Limb Development
Source: PLoS One. 2011 Dec 9;6(12):e28358. doi: 10.1371/journal.pone.0028358 (PMC3235105; doi:10.1371/journal.pone.0028358)
Supplement: Table S3 — Clusters of Differential Gene Expression. (PDF) [file pone.0028358.s006.pdf]

Table S3. Clusters of Differential Gene Expression. 20 major clusters were identified that grouped into the 4 major categories depicted in Figure 4. All transcripts from each cluster as listed in this table, and the Log2FC values.

Up Early-down late

	E9	E10	E11	E12	E13
**Cluster 1 (44 genes)**					
Hoxd8	2.4	2.4	2.2	1.7	2.2
Rabl3	1	3.1	2.4	2	1.2
Mmp14	1.3	1.6	2.3	1.1	1.8
Tbx15	2.3	3.8	2.9	1.9	1.4
Lef1	2.6	3.3	3	2.2	2
Pknox1	1.9	2.4	1.7	1.8	1.3
Fam120a	1.1	1.6	1.2	1.1	1
Efna4	1.2	3.2	3.1	2.7	2
Tbx5	1.8	2.4	2.8	3	2.6
Hoxa11	1.8	3.4	3	2	1.8
Pdia4	1	3.3	3.2	3	2.8
Dbn1	1.1	2	2.1	1.1	1.1
Hoxd10	2.1	2.9	2.3	2.7	2.1
Wasf2	1.1	2.2	2.3	1.7	1.3
Etv5	1.7	2.4	2.2	1.4	1.1
Zfp146	1.1	4.9	4.9	5.3	4.2
S1pr3	1.5	2.7	2.3	1.7	1.5
Prrx2	2	2.2	2.5	2.1	1.2
Rarg	1.6	3	2.9	2	1.5
Socs2	1.2	2.2	2.1	1.9	1.3
Sdccag3	1.2	3	3	2.1	1.6
Tshz1	1.5	1.1	1.7	1.7	1.6
Gpc3	1.4	2.7	2.6	1.7	2
Fam171a1	1.2	2.6	2.9	1.9	1.3
Msx1	1.7	2	1.9	1.9	1.6
Ephb4	1.1	2.9	3.3	2.2	2.1
Zkscan5	1.3	2.2	2.4	1.3	1
Tbx3	1.8	2.1	1.8	1.9	2.1
Parp8	1.2	1.9	1.4	1.5	1.2
Lix1	2.1	3.8	2.1	2	2
Smn1	1.9	4.2	4	2.7	1.5
Hand2	1.4	2.4	2.7	1.9	1.5
Cxcl14	1.3	3.9	4	2.5	1.6
Fmnl3	1.1	2.3	2.2	1.7	1.5
Prrx1	2.1	2.2	1.6	1.4	1.2
C130021I20Rik	2.9	4	3.8	3.4	3.6
Shox2	2.2	3.7	2.7	2.2	1.9
Ifitm2	1.3	3.7	4.3	2.6	1.8
Dusp7	1.5	3.1	3.2	1.8	2
Nolc1	1.2	3.5	3.3	3.1	2.2
Capn6	2.2	3.3	3	2.4	1.7
Kcne3	1.4	2.8	3.1	2.9	2.1
Sema5a	1.6	1.8	1.6	1.2	1.1
Sema3a	1.5	1.6	1.4	1.6	1.3
**Cluster 2 (51 genes)**					
Shc1	1.1	2.2	2.3	1.2	0
Ap2s1	1	2.6	2.5	1.9	0
Phlda3	1.1	1.5	1.7	1.2	0
Krt14	1	2.5	3.4	1.6	0
Mpzl2	1.5	1.5	1.5	1.2	0
Vasn	1.1	1.3	1.5	1	0
Trp63	1.6	1.1	1.1	1.3	0
Drg2	1	1.5	1.9	1.2	0
Sigmar1	1.3	1.7	1.8	1.2	0
Smad6	1.1	1.5	1.9	1.2	0
Aip	1.2	1.6	1.9	1.2	0
Bmp7	2.2	2.7	2.9	2.4	0
Pcgf1	1.8	3.3	3.3	1.8	0
Aspscr1	1	1.8	1.8	1.2	0
Spr	1.2	2.6	2.5	1.8	0
Stra6	1.2	2	2.4	1.4	0
Dhx8	1.2	1.8	1.8	1.1	0
Lhx9	3.8	2.8	2.7	1.9	0
Gpr182	1	1.4	1.7	1	0
Asna1	1.6	3.1	3.7	2.1	0
Hoxd9	3.7	2.6	2.2	1.4	0
Crlf1	2.1	1.9	2.3	1.3	0
Ssbp4	1.2	2.5	2.4	1.4	0
Msx2	1.6	1.8	1.7	1.5	0
Twist2	1.1	1.3	1.8	1.3	0
Echdc2	1	1.3	1.5	1.2	0
Nab2	1.4	1.6	2.1	1	0
Fam176b	1.2	1.8	1.8	1.4	0
Serpinf1	1.4	1.6	1.7	1.2	0
Pdlim4	1.1	1.7	2	1.3	0
Map2k6	1.6	2.8	2.6	1.3	0
Emp1	1.1	1.4	1.6	1.2	0
Ets2	1.5	1.5	1.3	1	0
Fgf8	1.9	4.2	2.1	1.3	0
Pcolce2	1.2	1.1	1.7	1.5	0
Wnt11	1	1	2.1	1.6	0
Ifitm3	1.1	2.1	2.3	2	0
Snai1	2	2.8	2.9	1.5	0
B4galt4	1.2	1.5	1.6	1.1	0
Cpxm1	1	1.5	1.8	1.3	0
Pfn1	2.5	4.7	4.9	2.5	0
Emilin1	1.4	1.8	1.6	1	0
6230427J02Rik	1.6	1.8	1.6	1.1	0
Ednra	1.1	1.8	1.3	1.1	0
Wif1	3.7	3.4	2.5	1.5	0
D17Wsu92e	1.5	2.7	2.3	1.2	0
Prdx6	1	2.1	1.9	1.3	0
Nnat	1.9	4.7	4.4	1.8	0
Ada	1.9	1.2	1.3	1.4	0
Ltbr	1.6	2.4	2.3	1	0
Bmp4	1.2	2.2	2.2	1.6	0
**Cluster 3 (113 genes)**					
Kctd12	1.3	1.4	1.4	0	0
Sh3bp1	1.4	1.2	1.5	0	0
Gtf3c1	1	1	1.3	0	0
Usp5	1.2	1.2	1.3	0	0
4632415K11Rik	1.2	1.3	1.2	0	0

Up late-down early

	E9	E10	E11	E12	E13
**Cluster 6 (475 genes)**					
Tes3-ps	0	1.2	1.1	1.1	1.2
Atp5e	0	1.6	1.7	1.6	1.3
Golt1b	0	1.9	1.4	1.7	1.1
Ext1	0	1.2	1.6	1.7	1.3
Med27	0	2.4	2.3	2.1	1.5
Six4	0	1.5	1	1.2	1.2
Epb4.1l2	0	1.6	1.7	1.5	1.4
Akt1s1	0	3.1	3.8	2.9	1.5
4933407H18Rik	0	1.5	1.6	1.7	1.7
Trpm7	0	1.2	1.2	1.3	1.6
Srpk2	0	1.2	1.2	1.4	1.4
Smek1	0	1.7	1.3	1.5	1.4
Agtr2	0	2.4	2.7	2	1.5
Slc25a24	0	3.6	3.7	3.3	3.5
A430107O13Rik	0	1.5	1.2	1.6	1.4
Btbd3	0	1.3	1	1.5	1.3
Dynlrb1	0	3.1	3.1	1.9	1.5
Rpl37	0	2.3	2.7	1.8	1.4
Il6st	0	1.1	1.3	1.5	1.5
2610018G03Rik	0	1.4	1.9	2.1	1.8
Sfxn4	0	1.5	1.3	1.6	1.2
Cdc26	0	1.2	1.5	1.3	1.1
Tgif1	0	1.6	1.6	1.3	1.3
Tmem26	0	1.7	1.5	1.3	1.4
Lmnb1	0	2.1	2	2.1	1.5
Fcho2	0	1.9	1.6	2.3	2.9
1110057K04Rik	0	1.5	1.2	1.4	1
Zfp825	0	1.7	1.6	1.4	1.3
Tmem184c	0	1.1	1.1	1.2	1.4
Eml2	0	1.8	1.9	1.4	1.1
Sntb2	0	1.4	1	1.5	1.2
Chd4	0	2.5	2.2	1.6	1.6
Cdc23	0	2.8	2.8	2.3	1.5
Exosc10	0	1.5	1.8	1.2	1
Fam176a	0	1.8	2.5	2.1	1.2
Shroom2	0	1.6	1.6	1.4	1.6
9530018H14Rik	0	1.9	2.4	2.3	1.4
8430426H19Rik	0	3	3	3	2.5
Cdh5	0	1.4	1.5	1.4	1
Sep15	0	1.3	1.6	1.2	1.1
Mdfi	0	2.5	2.7	2.4	1.5
Cacna1h	0	1.2	1.4	1.1	1.1
Gna12	0	3	2.7	2.2	1.7
Fv1	0	1.3	1.5	2.5	1
Zdhhc20	0	1.4	1.6	1.4	1.7
Sept10	0	2.7	2.4	2.2	1.4
Tmsb15b1-Tmsb1!	0	1.9	2.3	2.1	1.4
Sostdc1	0	1.1	2.5	2	1
5730437N04Rik	0	1.7	1.9	1.8	1.6
Rev1	0	1.3	1.3	1.7	1.2
Mrpl15	0	1.9	2.2	1.7	1.3
Inppl1	0	1.4	1.3	1.4	1.6
Rpl18	0	1.9	2.2	1.9	1.4
Ikzf5	0	1.4	1.3	1.2	1
Xrn1	0	1.8	1.7	1.9	2.1
Sugt1	0	1.1	1.3	1	1.2
Fibin	0	3.1	1.7	1.3	1.3
Naa10	0	1.6	1.8	1.5	1.1
Net1	0	4.2	4.6	3.8	3.3
Rnf4	0	1.1	1.4	1.5	1.2
Ndufb2	0	1.5	1.7	1.6	1.3
Gpr125	0	1.2	1.5	1.5	1.5
Arhgef2	0	2	2.1	1.5	1.1
Plekhh2	0	1.2	1.7	1.7	1.8
Ctnnal1	0	1.1	1.5	1.4	1.1
a	0	3.7	2.8	2	2.1
Ttc30b	0	2.3	2	2	1.6
Tmem87a	0	3.1	3.5	3.9	4.2
Phf6	0	5.3	4.8	4.2	3.9
Wdr35	0	1.6	1.5	1.9	1.3
Rabac1	0	2.3	2.6	2	1.3
Nasp	0	1.5	1.7	1.3	1.2
Ercc6l	0	1.5	1.8	2	1.2
Sptlc1	0	1.4	1.1	1.1	1.2
Zfp51	0	1.1	1.4	1.6	1.6
Mgat2	0	1.1	1.1	1.2	1.1
3110009E18Rik	0	1	1.5	1.4	1.5
Txndc5	0	1.7	1.7	1.6	1.4
Mkx	0	1.5	1.7	2.2	1.5
1810074P20Rik	0	2.2	2.5	2.2	2.1
Sp3	0	1.5	1.2	1.1	1.1
Ndufa7	0	1.9	2.2	1.8	1.5
Cox16	0	1	1.7	1.4	1.1
Plxdc2	0	1.3	1.2	1.3	1
Lrp6	0	1.7	1.5	2.2	2.1
Gdpd3	0	2.5	3.7	3.6	2.7
Arid5b	0	2.1	2.1	2.2	2.3
Cradd	0	1.7	2.1	1.9	1.1
Zfp458	0	2.4	2.3	2.6	2.4
2610005L07Rik	0	1.7	2.1	2.1	2
Usp8	0	1.6	1.4	1.8	1.7
Rpl36a	0	1.3	1.8	1.4	1.4
A730054J21Rik	0	3.2	3.1	2.9	1.6
Laptm4a	0	2.7	2.6	2.8	2
Ift20	0	2.2	2.5	2.3	1.7
Lnp	0	1.4	2	1.7	1.2
Mark3	0	1.2	1.3	1.1	1.2
Cant1	0	2	2.2	2.1	1.3
4632434I11Rik	0	2	1.8	2.3	1.7
Ostc	0	1.5	1.5	1.4	1.2
Rps24	0	2.3	2.9	1.7	1.8
Cox6b2	0	4	4	2.6	1.2
Fam70a	0	1.8	1.5	1.4	1.3
Rpl32	0	2.4	2.6	2.1	1.6

Peak at 11

	E9	E10	E11	E12	E13
**Cluster 10 (401 genes)**					
AA987161	0	1.3	1.4	2	0
Dapk3	0	2.4	2.5	1.6	0
4931414P19Rik	0	1.7	1.2	1.2	0
Arl16	0	1.3	1.5	1.7	0
Stambp	0	1.3	1.7	1.3	0
Cdh3	0	1.2	1.9	1.4	0
Hspb1	0	1.5	1.5	1.4	0
Serinc2	0	1.5	2.6	1.3	0
Helq	0	1.6	1.4	1.2	0
Lrp5	0	1.8	1.9	1.2	0
Spata7	0	1.3	1.6	1.4	0
Ubxn8	0	1.5	1.7	1.1	0
Sf3b5	0	1.1	1.4	1.5	0
Gm5617	0	1.1	1.4	1.2	0
Cep68	0	1.8	1.7	1.5	0
Cyp4f16	0	1.5	1.6	1.1	0
1700048O20Rik	0	1.2	1.2	1.8	0
Ccdc89	0	1.4	1.2	1.3	0
Mdm2	0	1	1.4	1	0
Pigb	0	1.5	1.3	1.6	0
Cldn10a	0	1.6	1.3	1.2	0
Pcdhb7	0	1.1	1.8	1.5	0
Fkbp7	0	1.1	1.4	1.3	0
Ptcd1	0	1.2	1.4	1.3	0
2610029G23Rik	0	1.5	1.4	1.2	0
Ddit4	0	2.1	2.6	1.4	0
Fmc1	0	1.5	1.8	1.6	0
1700052K11Rik	0	1.3	1.6	1.4	0
Cd99	0	1.8	2.3	1.5	0
Narf	0	1.4	1.2	1	0
Artn	0	2.2	2.4	2	0
Tm9sf4	0	1.1	1.3	1	0
Taf1c	0	1.1	1.5	1.3	0
Zfp882	0	1.5	1.6	1.5	0
Lig3	0	1.5	1.5	1.3	0
Tie1	0	1.8	1.5	1.4	0
Sfrs2ip	0	1.2	1.1	1.1	0
Calml4	0	3.1	1.7	1.6	0
Gm7444	0	1.6	1.5	1.5	0
Timp3	0	1.3	1.1	1.1	0
Rsrc1	0	1.7	1.3	1	0
Med12	0	1.5	1.7	1	0
Mrpl49	0	1.3	1.3	1.2	0
1110059G10Rik	0	1.3	1.3	1.1	0
Angptl2	0	2.1	2.3	1.8	0
Ube2c	0	1.6	1.9	1.2	0
Zfp647	0	1.1	1.1	1.1	0
Casp8	0	1.7	1.9	1.3	0
Mtmr2	0	1.3	1.4	1.1	0
Cdc42bpa	0	1.1	1	1.3	0
Qsox1	0	1.1	1.3	1.1	0
Gsg2	0	1.3	1.5	1.2	0
Gem	0	1.3	1.4	1.1	0
Rfc3	0	1.3	1.5	1.3	0
AI413582	0	1.2	1.7	1.4	0
Brp44l	0	1.3	1.5	1.3	0
Erf	0	1.9	2.4	1.5	0
Bckdhb	0	1.1	1.4	1.6	0
Hspa13	0	1.2	1.1	1.3	0
Nek4	0	1.5	1.6	1.3	0
Tmem30b	0	1.8	1.9	1.6	0
Eif2ak2	0	2.3	2.5	2	0
Aass	0	3.2	2.4	2.1	0
Scand1	0	1.8	2.3	1.4	0
Flywch2	0	1.9	2.2	1.6	0
Rnf10	0	1.6	1.4	1	0
5530601H04Rik	0	1.2	1.2	1.2	0
Zc3h14	0	1.2	1.1	1.1	0
Usp3	0	1.3	1.2	1.3	0
Myb	0	2.4	2.1	1.5	0
Scyl2	0	1.6	2	2	0
Acox3	0	1	1.4	1.2	0
Rpl12	0	1.1	1.5	1.2	0
Parva	0	1.4	1.5	1.1	0
Bcat2	0	2.5	2.4	1.8	0
Mertk	0	2.3	1.7	1.4	0
Ndufa4	0	1.1	1.4	1.2	0
Tbc1d10a	0	1.3	1.5	1.1	0
2510006D16Rik	0	1.3	1.2	1.1	0
Prkacb	0	1.3	1.2	1.1	0
Polr2e	0	1.1	1.2	1.4	0
Cdk2ap2	0	1.8	1.8	1.8	0
Gbas	0	1.6	1.8	1.1	0
Sfn	0	2.2	2.1	1.5	0
Zfp74	0	1.2	1.2	1.5	0
Mrpl30	0	1.5	1.6	1.4	0
Tubgcp2	0	1.5	1.9	1.2	0
Pgbd1	0	1.4	1.6	1.1	0
Sfmbt1	0	1.3	1.3	1.4	0
2010007H12Rik	0	2.2	2.2	1.2	0
Atn1	0	1.5	2.1	1.2	0
Rab4a	0	2.8	2.7	1.5	0
Alkbh6	0	1.2	1.4	1.3	0
Rbm4b	0	1.7	1.6	1.3	0
Pmaip1	0	4.2	2.4	1.4	0
Lrp4	0	1.8	1.4	1.4	0
Zfp706	0	1	1.1	1.1	0
Sirt6	0	1.6	1.8	1.1	0
Cep135	0	2	1.6	1.6	0
Zcchc4	0	1.4	1.6	1.3	0
Rnf14	0	1.2	1.7	1.4	0
Mbd2	0	1.5	1.6	1.2	0
Zfp574	0	1.6	1.7	1.6	0
Fcf1	0	1	1.5	1.3	0

On 10-11

	E9	E10	E11	E12	E13
**Cluster 12 (491 genes)**					
Eno1	0	1.3	1.8	0	0
Taf8	0	1.1	1.3	0	0
Top3a	0	1.1	1.4	0	0
Cuta	0	1.4	1.5	0	0
Rnaset2a	0	1	1.1	0	0
Ufd1l	0	1.1	1.1	0	0
Ccdc23	0	1.1	1.5	0	0
Snx2	0	1	1.2	0	0
Atxn2l	0	1.3	1.2	0	0
D430042O09Rik	0	1.4	1.5	0	0
Rpl41	0	1.1	1.4	0	0
Vps33b	0	1.2	1.6	0	0
Usp15	0	1.2	1	0	0
Tecr	0	1.2	1.4	0	0
Unc119	0	1	1.1	0	0
Smtn	0	1.5	1.4	0	0
2310047B19Rik	0	1.3	1	0	0
Dhx38	0	1.3	1.3	0	0
Rrp1	0	1	1.2	0	0
1110032A03Rik	0	1	1	0	0
Macc1	0	1.1	1.3	0	0
Uqcr11	0	1.4	1.5	0	0
Rfc1	0	1.1	1.2	0	0
Rasip1	0	1.3	1.3	0	0
BC027344	0	2.1	1.7	0	0
D10Wsu102e	0	1.1	1.2	0	0
E130309D02Rik	0	1.1	1.2	0	0
Snd1	0	1.4	1.6	0	0
Ccdc85b	0	1.6	1.8	0	0
Rasgef1b	0	1.7	1.9	0	-2
Atp5sl	0	1.2	1.3	0	0
Traip	0	1.1	1.1	0	0
Pltp	0	1.3	1.6	0	0
Hsbp1	0	1.2	1.2	0	0
Safb2	0	1.6	1.4	0	0
1700106N22Rik	0	1.2	1.1	0	0
Tomm40	0	1.4	1.7	0	0
Vps25	0	1.4	1.5	0	0
Psmd4	0	1.2	1.5	0	0
Wdr1	0	1.2	1.4	0	0
Puf60	0	1.1	1.2	0	0
Dnajc14	0	1.1	1.1	0	0
Fbrsl1	0	1.5	1.2	0	0
Tpmt	0	1.5	1.4	0	0
Comt1	0	1.1	1.1	0	0
Cd59a	0	1.3	1.5	0	0
Fkbp8	0	1.5	1.9	0	0
Fas	0	1.7	1.5	0	0
Deaf1	0	1.9	1.6	0	0
Islr	0	1.1	2.2	0	0
Lypla1	0	1.2	1.3	0	0
Mtr	0	1.2	1	0	0
Trp53rk	0	1.1	1.1	0	0
Cdc37	0	1.2	1.3	0	0
Leng1	0	1.5	1.9	0	0
Nt5dc2	0	1.2	1.2	0	0
Cdc42bpg	0	1	1.2	0	0
Scyl1	0	1	1.1	0	0
Calb2	0	1.2	1.2	0	-1
Rassf9	0	1.7	1.3	0	0
Polrmt	0	1	1.2	0	0
Vac14	0	1.6	1.6	0	0
Pim1	0	1.4	1.2	0	0
2810021B07Rik	0	1.1	1.3	0	0
Pbx2	0	1.6	1.7	0	0
Zfp658	0	1.2	1.4	0	0
Map3k3	0	1.1	1	0	0
Mtvr2	0	1.1	1	0	0
Runx1t1	0	1.6	1.5	0	0
Fxr2	0	1	1.1	0	0
Col23a1	0	1.1	1.1	0	0
Zfp688	0	1.2	1.2	0	0
Pgls	0	1.2	1.2	0	0
Hba-x	0	1	1.6	0	0
Rhov	0	1.1	1.2	0	0
4930471M23Rik	0	1.1	1.4	0	0
Copz2	0	1.3	1.8	0	0
Dcxr	0	1.4	1	0	0
Incenp	0	1	1.1	0	0
Mbd1	0	1.2	1.2	0	0
BC017158	0	1.1	1.7	0	0
Chac2	0	1.5	1.3	0	-1
Ethe1	0	1.3	1.1	0	0
Ap2a2	0	1.2	1.1	0	0
Sim2	0	1.8	1.3	0	0
Gm3716	0	1.4	1.1	0	0
Uba52	0	1	1.5	0	0
Dctn1	0	1.6	1.7	0	0
Agpat4	0	1	1.6	0	0
Pkig	0	1.1	1.3	0	0
Plxna1	0	1.5	1.3	0	0
Lsm6	0	1.1	1.1	0	0
AI837181	0	1.1	1.4	0	0
Col1a2	0	1.9	1.5	0	0
Gprc5b	0	1.3	1.4	0	0
Ttyh2	0	1.2	1.2	0	0
Kctd1	0	1.1	1.1	0	0
Jund	0	2	1.9	0	0
Camk1	0	2	2.4	0	0
Actn4	0	1.1	1.3	0	0
Gtf3c6	0	1.2	1.3	0	0
Zfp395	0	1.7	2.1	0	0
Bin3	0	1.1	1.3	0	0
Sept2	0	1	1.2	0	0

On 11-12

	E9	E10	E11	E12	E13
**Cluster 14 (153 genes)**					
Fam101a	-1	-1	1.5	1.2	0
Stxbp6	0	0	2	1.9	0
Rwdd3	0	0	1.4	1	0
Cryab	-1	0	1	1.2	0
Col17a1	0	0	2.3	2.1	0
Csgalnact1	0	0	1	1.4	0
Atpaf2	0	0	1.2	1.1	0
A930041H05Rik	0	0	1.3	1.1	0
AU020094	0	0	1.8	1.1	0
Myl1	0	0	3.6	3.3	0
Mtrr	0	0	1.1	1.6	0
Chrna1	0	0	1.7	1.2	0
Acot13	0	0	1	1.3	0
C1qtnf7	0	0	1.5	1.5	0
Ndufc2	0	0	1.2	1.3	0
Srd5a1	0	0	1.3	1.3	0
Ngrn	0	0	1.2	1	0
1810030N24Rik	0	0	1	1.2	0
Kdelc2	0	0	1.5	1.5	0
Brdt	0	0	1.5	1.5	0
Lgals3	0	0	1.4	1.4	0
2410017P09Rik	0	0	1.1	1	0
Papss2	0	0	2.9	2.7	0
Mgp	0	0	1.6	1.6	0
Prdxdd1	0	0	1.5	1.1	0
Gpx7	0	0	1	1.1	0
Rgnef	0	0	1.2	1.1	0
Cadm2	0	0	1.2	1.1	0
Ccdc109a	0	0	1.2	1.1	0
Spag7	0	0	1.4	1	0
Zfp709	0	0	1.2	1.3	0
Car12	0	0	1.2	1.5	0
5730455P16Rik	0	0	1.1	1.2	0
Il17b	0	0	1.2	1.3	0
Cops4	0	0	1	1	0
Arl5a	0	0	1.1	1.1	0
1110007C09Rik	0	0	1.6	1.3	0
Zswim7	0	0	1.7	1.4	0
Phlda2	-1	0	1.6	1.5	0
Gm70	0	0	1.2	1.3	0
Mcts1	0	0	1.1	1.1	0
Cytl1	0	0	1.1	2.6	0
Lum	-2	0	1.6	1.4	0
Galntl4	0	0	1.3	1.3	0
Creb3l4	0	0	1.1	1.2	0
Tatdn3	0	0	1.1	1.2	0
Eif2c2	0	0	1.3	1.1	0
Zfp52	0	0	1	1.6	0
Pofut1	0	0	1.1	1.3	0
Ifitm1	-2	0	2.1	1.7	0
Sav1	0	0	1.3	1.2	0
Snap23	0	0	1.3	1.1	0
Ndufs4	0	0	1.2	1.1	0
B9d1	0	0	1.4	1.4	0
4930547N16Rik	0	0	1	1.1	0
Has2	0	0	1.6	1.6	0
Msc	0	0	1.3	1.2	0
Ncrna00081	0	0	1	1.2	0
2410006H16Rik	0	0	1.2	1.1	0
Myl9	-2	0	2	1.3	0
Mia1	0	0	2.6	2.4	0
Lox	0	0	1.2	1.6	0
Col11a1	0	0	1.2	1	0
Tekt2	0	0	1.5	1.8	0
Casq2	0	0	1.3	1.4	0
Papola	0	0	1.1	1.3	0
1810063B07Rik	0	0	1.1	1	0
Mr1	0	0	1.7	1.7	0
Cxx1c	0	0	1	1	0
Smoc2	0	0	2	1.2	0
Crabp2	0	0	1.2	1	0
Pdgfd	0	0	1.3	1.6	0
Anxa8	0	0	1	1.3	0
Kbtbd10	0	0	3	2	0
Il17d	0	0	1.3	1.1	0
Chrng	0	0	1.5	1.5	0
5033414D02Rik	0	0	1	1.1	0
Ngfr	0	0	1	1.2	0
Tnni2	0	0	1.7	2	0
Zfp472	0	0	1.1	1.2	0
BC013529	0	0	1.2	1	0
Trim23	0	0	1	1.1	0
4930430F08Rik	0	0	1.1	1.2	0
Col9a2	0	0	2.4	1.8	0
N6amt1	0	0	1.4	1.6	0
Kdelr3	0	0	1.2	1.4	0
Camk2d	0	0	1.3	1.5	0
Mrps34	0	0	1.2	1.1	0
Eapp	0	0	1.3	1.1	0
Bmp2	0	0	1.9	2.2	0
Pigy	0	0	1	1.2	0
Col9a1	0	0	1.8	1.3	0
Ism1	0	0	1.2	1.2	0
Nfatc1	0	0	1.1	1.5	0
Matn1	0	0	1.6	1.7	0
Snap29	0	0	1.3	1.2	0
Spats2l	0	0	1.2	1	0
Peli2	0	0	1.2	1.2	0
Nans	0	0	1.1	1.1	0
Pthlh	0	0	1	1.8	0
Erg	0	0	1.6	1.7	0
2310016M24Rik	0	0	1.1	1.3	0
Gli2	0	0	1.1	1.2	0
Nfkbia	0	0	1.1	1.2	0

Bin1	1.8	1.3	1.4	0	0
Bhlha9	2.6	2.5	1.6	0	0
Nfil3	1.3	1.7	1.1	0	0
Tcof1	1.1	1.2	1.4	0	0
Fbln1	1.3	1.7	1.8	0	0
Dlk1	1.7	1.2	1.5	0	0
Kctd15	1.3	1.3	1.2	0	0
Aldoa	1.1	1.5	1.5	0	0
Spry4	1	1.7	1	0	0
2700081O15Rik	1.3	1.6	1.2	0	0
Igf2	1	2	2	0	0
Man1b1	1.2	1.5	1.6	0	0
Megf6	1.1	1.4	1.6	0	0
Akt1	1.3	1.2	1.2	0	0
Ascc1	1.4	1.7	1.8	0	0
Arpc4	1.4	2.8	3	0	0
Lsm2	1.8	2.7	2.8	0	0
Capg	1.6	1.4	1.6	0	0
Fgfr1	1.3	1.6	1.5	0	0
Daam2	2.1	2	1.5	0	0
Hgf	1.5	2.4	1.1	0	0
Ntf5	1.8	1.5	1.2	0	0
Ctdsp1	1.3	1.1	1.2	0	0
Ubap2	1.1	1	1.1	0	0
Farp2	1.3	1.3	1.1	0	0
Slc44a2	1.3	1.3	1.2	0	0
Dlg5	1.5	1.2	1.3	0	0
Mbd3	1.2	1.2	1.3	0	0
Lhx2	2.3	2.7	2.1	0	0
Emid2	1.8	1.8	1.6	0	0
Pddc1	1.6	2.5	2.1	0	0
Pofut2	1.5	1.8	2	0	0
Mybbp1a	1.1	1.2	1.4	0	0
2310016E02Rik	1.3	1.8	1.8	0	0
Ldb1	1.2	1.8	1.9	0	0
Cd276	1.4	2.2	1.9	0	0
Apex2	1.1	1.3	1	0	0
Ddx56	1.1	1.3	1.4	0	0
Cog1	1.1	1.3	1.3	0	0
Dusp6	1	1.4	1.2	0	0
Mknk2	1.1	1.4	1.4	0	0
Trp53	2.1	3.2	3.5	0	0
Tcf7	1.1	1.8	1.2	0	0
Atoh8	1.2	1.1	1.7	0	0
Khnyn	1.2	1.6	1.7	0	0
Med24	1	1.2	1.2	0	0
Traf4	1.1	1.7	1.7	0	0
Tmem109	1.2	1	1.6	0	0
Tmem98	1.4	1.8	1.6	0	0
Nme4	1.1	1.1	1.2	0	0
Sars	1	1.3	1.1	0	0
Ifrd2	2	2.3	2	0	0
Itpkb	1.2	1.4	1	0	0
Mdk	1.4	1.7	2.2	0	0
Fzr1	1.1	1.8	2.1	0	0
Ctsb	1.3	1.3	1.5	0	0
Cdo1	2.8	2.2	1.4	0	0
Ehmt2	1.1	1.5	1.5	0	0
Slc25a10	1.2	1.6	1.7	0	0
Dchs1	1.4	1.4	1.5	0	0
Lpcat3	1.2	1.1	1.2	0	0
Cc2d1a	1.2	1.4	1.4	0	0
Tcf3	1.2	2	2.1	0	0
Krt5	1.8	3	2.4	0	0
Shmt2	1.3	1.3	1.3	0	0
Ilkap	1.1	1.5	1.5	0	0
Dbnl	1	1.1	1.3	0	0
Sdc1	1.1	1.1	1.4	0	0
Pdxp	2	1.5	1.5	0	0
Mmp2	1.8	1.6	1.4	0	0
Itgb5	1.2	1.4	1.2	0	0
Pcid2	1.2	1.7	1.7	0	0
Tmem119	1.6	1.4	1.2	0	0
Slc4a2	1.7	1.9	1.8	0	0
Leprel2	1.3	1.9	2.1	0	0
Zswim1	1.1	1.1	1.2	0	0
Twist1	1.9	1.6	1.5	0	0
Rbm19	1.5	1.3	1.1	0	0
Gatad2a	1.3	1.3	1.3	0	0
Psmb7	1.5	1.7	1.9	0	0
Grasp	1.2	1.1	2.4	0	0
Abcb8	1	1.2	1.8	0	0
Igfbp3	1.3	1.1	1	0	0
Phc2	1.5	1.3	1.3	0	0
Shf	1.3	1.4	1.6	0	0
Agap3	1.2	1.3	1.1	0	0
Isyna1	1.4	1.3	1.2	0	-1
Gpsm3	1.3	1.2	1.1	0	0
Selenbp2	1.4	1.1	1.6	0	-1
Mapk6	1.5	2.7	2.4	0	0
Hspbp1	1.2	1.2	1.4	0	0
Pkdcc	2.2	1.7	1.2	0	0
Tsen54	1.3	1.5	1.6	0	0
Dennd1a	1.3	1.3	1.5	0	0
Def6	1.3	1.4	1.4	0	0
Ptn	1.1	1.6	1.1	0	0
Alx3	1.8	1.2	1.3	0	0
Upf1	2	2.3	2.1	0	0
Shank3	1.5	1.4	1.5	0	0
Notum	1.5	1.4	1.1	0	0
Limd2	1.1	1.5	1.7	0	0
Csnk1g2	1.1	1.9	1.8	0	0
Lrrc49	1.2	2.1	1.7	0	0
Arf4	1.2	1.9	2.2	0	0
Plekhj1	1.6	2.8	2.9	0	0
Pelp1	1.1	1.2	1.5	0	0
Lace1	1.1	1.3	1.1	0	0
Lbx1	1.7	2	1.2	0	0

Nnt	0	1.4	1.6	1.5	1.3
Atp2c1	0	2.3	2.1	2.3	1.4
Tnpo2	0	2.2	2.1	1.6	1.3
Bud31	0	1.8	2	1.5	1.1
Ift74	0	1.1	1.6	1.4	1.2
Rbl2	0	2.1	2.1	1.5	1.1
Rbm6	0	1.8	1.6	1.5	1.3
Stag1	0	1.5	1.5	1.6	1.6
Prmt1	0	1.8	2.3	2.2	1.3
Cdk14	0	2.4	2	2.7	1.5
Syne2	0	2	2	1.7	1.4
Gm9735	0	2.6	2.1	1.9	1.1
Ppwd1	0	1.9	1.8	1.7	1.1
Col2a1	0	1.4	2.5	1.5	1
Selm	0	2.6	2.7	2.1	1.1
Casc5	0	2.4	1.9	2.5	2.2
Hoxd13	0	3.2	3.2	2.9	2.8
Ptk7	0	3.8	3.7	2.8	2.8
Ankhd1	0	1.5	1.7	2	2.1
Zfp62	0	1.8	1.5	1.6	1.7
Xrn2	0	1.6	1.7	1.7	1.7
Mum1l1	0	1.1	1.2	1.6	1.7
Srp19	0	1.4	1.6	1.7	1.5
Polh	0	1.8	1.8	2	1.5
Adrbk2	0	2	1.5	2	2.2
Gnpnat1	0	1.6	1.7	1.8	1.3
Prpf39	0	1.3	1.1	1.3	2
Nid2	0	2	2.1	1.9	2.1
Zbtb8os	0	1.7	2.1	1.6	1.2
1810014B01Rik	0	1.8	1.8	1.7	1.9
Cldn4	0	1.8	1.3	2.1	1.4
Zdhhc15	0	1.6	1.8	2	1.4
Fyttd1	0	1.9	2	2.4	2.3
Mme	0	2.8	3.6	3.7	3.3
Zfp260	0	1.3	1.4	1.3	1.6
Anapc10	0	1.3	1.3	1.8	1.3
Rps15	0	2.3	2.6	1.8	1.3
A230046K03Rik	0	1.5	1.2	1.7	1.9
Dis3l	0	1.1	1.3	1.4	1
Scx	0	1.4	1.9	1.7	1.1
Scaper	0	3.7	3.1	3.9	3.8
Bzw1	0	1.2	1.4	1.3	1.2
Otub1	0	3.6	3.6	3	1.9
Mycl1	0	2.5	2.8	2.2	1
Gm5595	0	2.3	2	2	1.4
Ranbp6	0	2.9	2.4	2.6	2.4
Raet1a	0	1.6	2.4	1.9	1.7
Axl	0	1.7	1.8	1.3	1.6
Senp7	0	1.1	1	1.1	1.3
Atp5l	0	2.7	3.2	2.8	2.5
2810030E01Rik	0	1.6	1.5	2.3	1.2
Fut11	0	1.9	1.5	1.9	1.4
Uso1	0	1.2	1.2	1.6	1.5
Homer1	0	2.4	1.7	2	1.6
Dyrk1a	0	2.1	1.9	2.1	1.7
Mirg	0	1.1	1.5	1.9	3.5
Ttc26	0	1.3	1.2	1.2	1.2
Tmed2	0	2.7	2.7	2.5	2.2
Ndufa6	0	1.3	2.2	1.6	1.2
AI506816	0	5.4	5.5	5.1	4.9
Zkscan3	0	1.5	1.6	1.8	2
Mrps16	0	1.5	1.8	1.6	1.1
Vps35	0	1.2	1.3	1.5	1.3
Ube2w	0	1.1	1	1.1	1.1
Lrig3	0	2.1	2.1	2.1	2.1
Eif4a1	0	2.8	2.9	2.2	2.1
Prpf40a	0	1.8	1.5	1.6	1.7
Spa17	0	2.4	2	1.6	1.3
Mmp16	0	1.8	1.4	1.9	1.7
Itga5	0	1.8	2.3	1.9	1.8
Ccdc53	0	1.9	2.2	1.9	1.3
Pecam1	0	3.2	3.3	3.1	3.1
Cpd	0	1.4	1.2	1.4	1.1
2410002F23Rik	0	2	2.4	1.3	1.5
Qrich1	0	1.5	1.6	1.3	1.2
1110018J18Rik	0	2.1	2.2	2.4	1.2
2310045N01Rik	0	2	1.8	1.8	1.1
D6Wsu116e	0	1.8	1.8	1.6	1.5
Igf2as	0	1.5	1.1	1.5	1.1
Commd2	0	1.2	1.6	1.5	1.3
Rpp21	0	1.9	2.3	2.1	1.4
Grem1	0	3.8	1.9	3.1	1.9
Pdgfra	0	1.7	1.8	1.5	1.5
Frk	0	1.2	1.4	1.7	1.4
Poc1b	0	2.3	1.8	1.6	1.3
Adat2	0	1.4	1.7	2.1	1.8
Zc3h13	0	1.5	1.3	1.1	1.3
Megf9	0	2.3	2.4	2.8	2.9
Col12a1	0	1.2	1.1	1.4	1.4
Gulp1	0	1.4	1.7	1.4	1.6
Gabpb2	0	1.5	1.3	1.8	1.6
Ccdc77	0	2.2	2.1	2	1
St3gal4	0	2.6	2.8	2.3	2.1
Tceb2	0	2.4	2.6	1.9	1.3
Ing3	0	1.5	1.4	1.7	1.2
Rps27	0	1.4	2.1	1.4	1.1
Zfp53	0	1.3	1.4	1.6	1.4
Ctcf	0	1.2	1.2	1.3	1.2
Itga9	0	1.3	1.9	1.8	1.6
Bcam	0	2.9	2.8	2.5	2.4
Atp10d	0	2	1.7	2.4	2
Cap1	0	4.5	4.9	4.3	5.4
Nop58	0	1.8	1.9	1.7	1.4
Cacna1g	0	1.9	2.2	1.2	1.2
Hoxd11	0	4.7	4.3	4	2.8
Fli1	0	2.1	1.8	1.4	1.3
BC005561	0	2.3	1.9	2.5	2.9
Gabpb1	0	1.7	1.8	1.9	1.2
Acn9	0	1.2	1.7	1.9	1
1700066M21Rik	0	3	2.5	2.8	1.6
Cdc27	0	1.4	1.5	1.4	1.1

Sepw1	0	1.7	2.2	1	0
Rfxank	0	1	1.1	1.1	0
Smpdl3b	0	1.4	1.5	1.7	0
Stk4	0	1.5	1.2	1.3	0
Acer3	0	1	1.1	1.5	0
Abca1	0	1	1	1.2	0
Psmd8	0	1.7	1.7	1.3	0
Ccdc109b	0	3.6	2.7	1.8	-1
Dnajc15	0	1.4	1.7	1.6	0
Ctr9	0	1.6	1.8	1.3	0
Zfp295	0	1.3	1.5	1.3	0
Rhoc	0	1.8	2.1	1.5	0
Gpatch2	0	1.3	1.2	1.1	0
Ctse	0	1.6	2.9	1.5	-1
Timm8b	0	1.1	1.3	1.2	0
Mrpl32	0	1.1	1.4	1.6	0
Chrdl1	0	1.2	2	1.9	0
Pdpk1	0	1.5	1.2	1.5	0
Akr7a5	0	1.4	1.8	1.3	0
Arpp19	0	1	1.1	1.1	0
Spop	0	1.3	1.8	1.1	0
Anp32a	0	1.9	1.7	1.1	0
Chtf8	0	1.3	1.4	1.3	0
Scyl3	0	1.2	1.1	1.2	0
Aldh4a1	0	1.4	1.8	1.1	0
A2ld1	0	1.3	1.3	1.3	0
Fbxl19	0	2.7	2.7	1.8	0
Fus	0	1.9	2.1	1.1	0
Ugcg	0	1.4	1.1	1	0
Ciz1	0	1.6	1.8	1.1	0
Zfp35	0	1.5	1.3	1.4	0
Mipol1	0	1.4	1.2	1.3	0
Snurf	0	4.8	4.2	3.1	0
Stat2	0	1.1	1.3	1.2	0
Zfp287	0	1.9	1.6	1.8	0
Wdr85	0	1.3	1.5	1.1	0
Anxa6	0	1.5	2.3	1.1	0
Cab39l	0	1.2	1.5	1.3	0
Robld3	0	1.3	1.1	1.3	0
Ncapg	0	1.2	1.1	1.5	0
Lin9	0	1.1	1.2	1.2	0
C330006A16Rik	0	1.8	1.6	1	0
Cited1	0	1.7	2.1	2.1	0
Dcps	0	1.1	1.1	1.1	0
Lgals1	0	2.1	2.2	1.3	0
Fxyd3	0	1.9	2.1	1.8	0
Plekhf2	0	1.4	1.6	1.7	0
Alg12	0	1.7	1.4	1.3	0
Zfp558	0	2	2	2.1	0
Ranbp1	0	1.1	1.1	1.2	0
Lrrc68	0	1.9	1.6	1.2	0
Birc5	0	1.5	1.8	1.8	0
Fam171a2	0	2	2.2	1.7	0
Vti1b	0	1.1	1.3	1.2	0
Dsg2	0	1.2	1.5	1.6	0
Hmox1	0	1.6	1.6	1.7	0
Mdm1	0	1.3	1.3	1.3	0
2810428I15Rik	0	1.8	1.8	1.8	0
Aes	0	3.6	3.1	1.6	0
Nipsnap1	0	1.5	1.3	1.2	0
D3Ertd751e	0	1.9	2.1	1.5	0
Fkbp2	0	1.2	1.1	1.2	0
Mrpl27	0	1.1	1.6	1.3	0
Arl15	0	1.4	1.6	1.2	0
A030009H04Rik	0	1.9	1.5	1	0
Fam36a	0	1.1	1.3	1.2	0
Chchd3	0	1.5	1.7	1.5	0
Raly	0	1.5	1.9	1.2	0
Ola1	0	1.2	1.6	1.6	0
Mgst1	0	1.4	1.9	1.9	0
Rps26	0	1.3	1.7	1.2	0
Nqo1	0	2.1	1.9	1.1	0
Ercc8	0	1.4	1.3	1.3	0
Snx9	0	1.1	1	1.2	0
Zfp846	0	1.2	1.1	1.2	0
Tcea1	0	2	2	1.3	0
Rad1	0	1.2	1.5	1	0
Zfp316	0	1.1	1.5	1.2	0
Snrpc	0	1.3	1.6	1.1	0
Rps8	0	1.3	1.6	1	0
Tubgcp6	0	1.6	1.3	1.2	0
Sept8	0	1	1.2	1	0
Clpb	0	1.5	1.4	1.1	0
Olfml1	0	1.9	2.1	1.9	0
Taf12	0	1.6	1.6	1.6	0
Tfpt	0	1.7	1.8	1.1	0
Prkch	0	1.7	1.3	1.1	0
Bid	0	1.3	1.3	1.1	0
Erh	0	1.2	1.5	1	0
Cdc42ep5	0	1.9	2.4	1.5	0
Snapc2	0	1.9	2.2	1.1	0
Exoc6b	0	1.7	1.9	1.3	0
Nkd2	0	1.6	1.6	1	0
Mpst	0	2.3	2.6	1.8	0
2810408A11Rik	0	1.7	1.9	1.3	0
Chmp2a	0	1.6	1.8	1.1	0
Jhdm1d	0	2.3	1.5	1.6	0
Arhgef3	0	1.6	2.2	1.7	0
Fanci	0	2.9	2	2.3	0
Stip1	0	1.5	1.7	1.1	0
Aqp1	0	1	1.1	1.5	0
Lin37	0	2.2	1.8	1.3	0
Gstm2	0	1.6	1.9	1.5	0
1810027O10Rik	0	1.1	1.6	1.3	0
2400009B08Rik	0	1.3	1.6	1.7	0
Zfp428	0	1.7	2	1.1	0
Drap1	0	2.4	2.4	1.4	0
Fam149a	0	1.3	1.3	1.1	0
Rpe	0	1.4	1.5	1.6	0
Mast2	0	1.4	1.3	1.1	0
Cdk19	0	1.4	1.2	1.5	0

Ctxn1	0	1.7	2.3	0	0
Mgrn1	0	1.1	1.4	0	0
Rab4b	0	1.1	1.4	0	0
Cbx2	0	1.2	1.4	0	0
Galnt10	0	1	1.2	0	0
Eif2ak1	0	1.1	1.4	0	0
Tspan15	0	1.1	1.1	0	0
Bcas3	0	1.2	1.6	0	0
Cenpv	0	1.3	1.2	0	0
Plscr3	0	1	1.2	0	0
1110008P14Rik	0	1.2	1.5	0	0
Kat2a	0	1.2	1.3	0	0
Nrtn	0	1.1	1.6	0	0
Gsc	0	2.9	1.8	0	0
Frg1	0	3.5	4.7	0	0
Stk35	0	2.1	1.7	0	0
Csnk1d	0	1.1	1.2	0	0
Tbcc	0	1.1	1	0	0
Lnx2	0	1.4	1.1	0	0
Ube2d1	0	2.5	2.5	0	0
Vasp	0	1.1	1.4	0	0
Gspt1	0	1	1.1	0	0
Ppdpf	0	1.4	1.6	0	0
Hsd11b2	0	1.8	1.7	0	0
Arf5	0	1.4	1.6	0	-2
Ptms	0	1.7	1.9	0	0
Arrb2	0	1.2	1.3	0	0
Ndufa2	0	1	1.4	0	0
Ube2m	0	1.7	2	0	0
Zfp142	0	1	1	0	0
Slc37a3	0	1.2	1.2	0	0
Pex5	0	1.5	1.2	0	0
Gnb1	0	1.1	1.3	0	0
Fam117a	0	1.2	1.9	0	0
Ring1	0	1.3	1.3	0	0
Josd2	0	1.6	2.1	0	0
BC030867	0	1.3	1	0	0
Cacnb3	0	1.1	1.1	0	0
2310014D11Rik	0	1.2	1.3	0	0
Fam193b	0	1.3	1.4	0	0
Mark2	0	1.8	1.6	0	0
9030624J02Rik	0	1.1	1.2	0	0
1500010J02Rik	0	1.1	1	0	0
Praf2	0	1.3	1.3	0	0
Dnmt3a	0	1.5	1.1	0	0
Dhrs11	0	1.8	1.7	0	-3
Rps6kb2	0	1.1	1.2	0	0
Lipt2	0	1.1	1.2	0	0
Hnrnpd	0	1.7	1.9	0	0
Gja3	0	1.2	1.5	0	0
Dkc1	0	1.1	1.3	0	0
Sumf2	0	1.3	1.4	0	0
1110007A13Rik	0	1.3	1.2	0	0
Ssh1	0	1.3	1.4	0	0
Nedd8	0	1.5	1.5	0	0
Pctp	0	1.3	1.3	0	0
Fem1a	0	1.3	1.1	0	0
Calcoco1	0	1.9	2	0	0
Ppp1r14b	0	1.2	1.6	0	0
AI480653	0	1	1.1	0	0
Bcl9	0	1	1.2	0	0
Brap	0	1.1	1	0	0
Cd82	0	1.3	1.8	0	0
Pcx	0	1.2	1.2	0	0
Nudc	0	1.1	1.1	0	0
4930452B06Rik	0	1	1.2	0	0
Cox6a1	0	1	1.3	0	0
Sept9	0	1.1	1.3	0	0
Fgfrl1	0	1	1.1	0	0
Ilf2	0	1.2	1.1	0	0
Srprb	0	1.2	1.1	0	0
Sertad3	0	1	1.2	0	0
Hes1	0	1	1.5	0	0
Zfp362	0	1.1	1.5	0	0
Tom1	0	1	1.7	0	0
Hba-a1	0	1	1.8	0	0
Atf6b	0	1.2	1.9	0	0
Rhob	0	1.3	1.5	0	0
En1	0	1	1.6	0	0
Gcn1l1	0	1.1	1	0	0
Prpf8	0	1.2	1.3	0	0
Sdf4	0	1.1	1.2	0	0
1600002H07Rik	0	1.1	1.3	0	0
Lancl2	0	1.1	1.1	0	0
Tmem106c	0	1.3	1.5	0	0
Sept6	0	1.5	1.6	0	0
Rara	0	1.3	1.3	0	0
Wipi1	0	1.4	1.3	0	0
Ip6k2	0	1.1	1.6	0	0
Srfbp1	0	1	1.1	0	0
Cxxc1	0	1.1	1.5	0	0
Zfp335	0	1.4	1.5	0	0
Alg2	0	1.5	1.8	0	0
Mrps10	0	1	1.1	0	0
Etv4	0	1.9	1.5	0	0
H2afv	0	1.1	1.3	0	0
Adck4	0	1.4	1.2	0	0
Atf5	0	1.1	1.4	0	0
Zfp423	0	1.8	2.1	0	0
Rpusd2	0	1.7	1.1	0	0
Skiv2l	0	1.9	1.8	0	0
Abhd12	0	1.2	1.1	0	0
BC068281	0	1.3	1	0	0
Zfp358	0	1.5	1.5	0	0
Kat5	0	1.4	1.6	0	0
Samd4b	0	1.1	1.6	0	0
Pcdhb22	0	2.3	1.6	0	0
Mrp63	0	1.1	1.2	0	0
Myo1e	0	1.1	1.2	0	0
Pvrl4	0	1.1	1.1	0	0
Zfyve19	0	1.1	1.2	0	0

Chrac1	0	0	1.6	1.4	0
Flnb	0	0	1.6	1.2	0
Krt17	0	0	2.8	2.4	0
Kbtbd5	0	0	1	1.2	0
0610037L13Rik	0	0	1	1	0
Scin	0	0	1.2	1.3	0
Sap18	0	0	1	1.3	0
Chst11	0	0	1.4	1.3	0
Alg11	0	0	1.1	1.3	0
8430434A19Rik	0	0	1.6	1.8	0
Tnk2	0	0	1.1	1.2	0
Manbal	0	0	1.4	1.3	0
Cib2	0	0	1.5	1.3	0
Cdkn3	0	0	1.2	1.1	0
Esrp1	0	0	1	1.4	0
Tor1aip1	0	0	1.1	1.3	0
Rps13	0	0	1.4	1.2	0
Prss35	0	0	1.4	1.5	0
Arsj	0	0	1.6	1.6	0
Pqlc3	0	0	1.4	1	0
5730409N16Rik	0	0	1.2	1	0
Cpa3	0	0	1.6	2.4	0
Enpp1	0	0	1	1.1	0
Adamts5	0	0	1.6	1.4	0
Myod1	0	0	2.3	1.4	0
Mrpl43	0	0	1.3	1.1	0
Apip	0	0	1.1	1.3	0
Mrps18c	0	0	1.1	1	0
Zfp566	0	0	1	1.1	0
Mrs2	0	0	1.1	1.3	0
Mettl10	0	0	1.2	1.2	0
Ppih	0	0	1.2	1.2	0
Ppt1	0	0	1.1	1	0
Tmem38a	0	0	1	1	0
Tox	0	0	1.6	1	0
Lsm3	0	0	1.4	1	0
Uqcrh	0	0	1.1	1	0
Dlx5	0	0	2.1	1.3	0
Glrx	-1	0	1.2	1.2	0
Cdh15	0	0	1.3	1.1	0
U2af1	0	0	1.6	1.1	0
Lmod3	0	0	1.4	1.5	0
Hfe2	0	-1	1.5	1.2	0
Lipe	0	0	1	1	0
2310039H08Rik	0	0	1.1	1.4	0
Kcne1l	0	0	1.9	1.1	0
P4ha3	0	0	1.7	1.1	0
Chst2	1.4	0	1.6	1.5	0
Prdm1	1.4	0	1	1	0

Cluster 15 (159 genes)

Ap3m1	0	0	0	1.1	0
Calml3	0	0	0	1	0
Pygo1	0	0	0	1.1	0
Ifit2	0	0	0	1	0
Tnnt3	0	0	0	1.4	0
Hps5	0	0	0	1	0
Hiatl1	0	0	0	1.2	0
Bod1l	0	0	0	1	0
Trim29	0	0	0	1	0
Zfp715	0	0	0	1.1	0
1810063B05Rik	0	0	0	1.2	0
Pik3c2a	0	0	0	1.1	0
Zfp354b	0	0	0	1	0
Ociad1	0	0	0	1.3	0
Filip1l	0	0	0	1.3	0
Ttc32	0	0	0	1.5	0
Fbxo30	0	0	0	1	0
Txndc12	0	0	0	1.1	0
Car14	0	0	0	1	0
Dcun1d1	0	0	0	1.1	0
Mgat3	0	0	0	1.3	0
Osbpl3	0	0	0	1.4	0
C130050O18Rik	0	0	0	1	0
4933421E11Rik	0	0	0	1.1	0
Zmym1	0	0	0	1.1	0
Nebl	-1	0	0	1.4	0
Itga8	0	0	0	1.2	0
Hspb11	0	0	0	1.2	0
Apobec2	-2	-2	0	1.4	0
Epyc	0	0	0	1.6	0
Pcdhb11	0	0	0	1.1	0
Btaf1	0	0	0	1	0
Ddb2	0	0	0	1.2	0
Wapal	0	0	0	1	0
Sstr1	0	0	0	1	0
Ntn4	0	0	0	1.1	0
Musk	0	0	0	1.4	0
Srd5a2	0	0	0	1.4	0
2700097O09Rik	0	0	0	1.2	0
Cnksr3	0	0	0	1.2	0
Zfp110	0	0	0	1.2	0
Fam13a	0	0	0	1	0
Srbd1	0	0	0	1.1	0
Pno1	0	0	0	1	0
Tm2d3	0	0	0	1.2	0
Mcts2	0	0	0	1	0
A530047J11Rik	0	0	0	1.2	0
Col11a2	0	0	0	1.1	0
Rpf1	0	0	0	1.1	0
Zfp324	0	0	0	1.3	0
Klf10	0	0	0	1.1	0
Jak1	0	0	0	1.1	0
Pdhx	0	0	0	1.3	0
Ammecr1	0	0	0	1.1	0
Edn3	0	0	0	1	0
Zfp57	0	0	0	1	0
Lrrc51	0	0	0	1	0
Cav3	0	0	0	1	0
Agl	0	0	0	1.2	0
Pde7b	0	0	0	1.1	0

Cluster 4 (39 genes)

Gjb3	1.6	1.4	0	0	0
Tmem173	2.1	1	0	0	0
Dcaf6	1	1.7	0	0	0
Gm11772	1	1	0	0	0
Fbxw8	1.5	1.4	0	0	0
BC005624	1	1.1	0	0	0
Tshz2	1.9	1.1	0	0	0
Sept5	1.6	1.3	0	0	0
Dact3	1.1	1.6	0	0	0
Tmc6	1.4	1.1	0	0	-1
Mup1	2.4	2.5	0	0	0
Asb4	1.9	1.7	0	0	0
Phactr3	1.2	1.6	0	-2	-2
1190002H23Rik	1.2	1.4	0	0	-1
Psmc5	1.1	1.2	0	0	0
Mogat2	1.4	1.5	0	0	-1
Scube3	1.2	1.2	0	0	0
1200009O22Rik	1.8	1.1	0	0	0
Stambpl1	1.4	1.1	0	0	0
Ripk3	1.1	1.3	0	0	0
Pcolce	1.5	1	0	0	0
Pdgfrb	1	1.2	0	0	0
Lhfp	1.4	1.2	0	0	0
Ormdl3	1.1	1.1	0	0	0
Myst3	1.2	1.3	0	0	0
Cyp27a1	1.5	1.2	0	0	0
Rspo4	2.8	1.8	0	0	0
Dnm1	1.8	1.3	0	0	0
Dusp2	1.3	1	0	0	0
Kank4	1.2	1.2	0	0	0
Ppfibp2	1.1	1.1	0	0	0
Por	1.2	1.2	0	0	0
Rnf19b	1	1.2	0	0	0
Gpc1	1.2	1.3	0	0	0
Optn	1.4	1.5	0	0	-1
Wnt6	2	1	0	0	0
Cldn6	2	1.9	0	0	0
Usp29	1.1	1.4	0	0	0
Figf	3.3	1.2	0	0	0

Cluster 5 (186 genes)

Antxr1	1	0	0	0	0
Ppif	1.1	0	0	0	0
Tmem59l	1.3	0	0	0	0
Bach2	1.4	0	0	-1	-1
Crmp1	1.6	0	-3	-4	-4
Mkl1	1.3	0	0	0	0
Casd1	1.1	0	0	0	0
Lad1	1.7	0	0	0	0
Tcfap2c	1.9	0	0	0	0
Tulp2	1.1	0	0	0	0
Col14a1	1.6	0	0	0	0
Il17rd	1	0	0	0	0
Tmem178	1.4	0	0	0	-1
Cyth2	1.2	0	0	0	0
Myc	1.2	0	0	0	0
Cpz	2.2	0	0	-2	-2
AA667102	1.1	0	0	0	0
Fdx1	1	0	0	0	0
Dusp9	1.1	0	0	-1	-2
Pycr1	1	0	0	0	0
Ptpn9	1.1	0	0	0	0
Dpep1	1	0	0	0	0
Ssbp3	1.2	0	0	0	0
Cdc42ep1	1	0	0	0	0
Smarcd2	1.2	0	0	0	0
S1pr2	1.1	0	0	0	0
Rap1gds1	1.2	0	0	0	0
Gal	1.8	0	0	0	-2
Parp16	1.1	0	0	0	0
C1qtnf2	1	0	0	0	0
Zfp775	1.1	0	-1	-2	-2
Tbc1d7	1.1	0	0	0	0
Chst8	1.2	0	0	0	-1
Mfsd10	1.2	0	0	0	0
Zyx	1.2	0	0	0	0
Pik3r2	1.1	0	0	0	0
Otud3	1.3	0	0	0	0
Snap91	1.9	0	-2	-2	-2
Adamts1	1	0	0	0	0
Larp1	1	0	0	0	0
Hoxa4	1.3	0	0	-1	0
Blvra	1.1	0	0	0	-1
Nploc4	1.1	0	0	0	0
Fam110a	1.3	0	0	0	0
Zfp771	1	0	0	0	0
Xkr5	1.1	0	0	0	0
Apcdd1	1.2	0	0	0	0
Ppm1m	1	0	0	-1	-1
Csk	1.6	0	0	0	0
Fcer1g	1.2	0	0	0	0
Ptges2	1.4	0	0	0	0
Btbd11	1.9	0	0	0	0
Prdm16	1.2	0	0	0	0
Tmem54	1.3	0	0	0	0
Plxnb2	1.1	0	0	0	0
Enox1	1.1	0	0	0	0
Ddx41	1.2	0	0	0	0
Tns1	1.1	0	0	0	0
Farp1	1.1	0	0	0	0
Ppp5c	1.2	0	0	0	0
Xab2	1.8	0	0	0	0
E130012A19Rik	2.4	0	0	0	0
Zgpat	1	0	0	0	0
Khsrp	1.8	0	0	0	0
Trmt1	1	0	0	0	0
Git1	1.3	0	0	0	0
Rrn3	1	0	0	0	0
Rbpms	1.2	0	0	0	0
Per1	1.3	0	0	-1	-1

Crebzf	0	2.2	2.3	1.7	2.5
Dus4l	0	2.6	2.4	2.1	1.1
Angptl1	0	1.8	1.9	2.1	1.8
C630043F03Rik	0	1.7	1.6	1.8	1.2
Kdm5a	0	3	2.2	3.1	3.1
Maz	0	2.6	2.8	1.8	1.3
Sox8	0	2.2	1.9	2.7	2.5
Gm3168	0	1.3	2	2.5	2.3
Rnf144a	0	1.1	1.2	1.6	1.5
Cfl1	0	3.7	4	2.4	2.2
D10627	0	3	2.5	3.2	2.7
Usf1	0	2.8	2.3	2.2	1.8
Pi4kb	0	3.3	3.7	3.9	4
Zswim6	0	2	1.3	1.7	1.4
Zfa	0	3.4	3.2	3.9	2.5
Tsga14	0	1.7	1.7	2	1.9
Rac3	0	2.9	3.3	2.8	1.1
Aff2	0	2.2	1.9	1.1	1
Chchd5	0	1.8	1.9	1.7	1
4931406C07Rik	0	2.5	2.7	2.5	2
Klra18	0	2.4	2.3	1.5	1.6
Ube4a	0	2.2	1.9	1.7	1.2
Top2b	0	1.3	1.4	1.1	1.3
Hadha	0	2.5	3.1	2.2	1.4
Fxc1	0	2.7	2.9	2.4	1.4
Deb1	0	1.7	1.8	2.1	1.4
Fdps	0	2.2	2	2	1.3
Mmgt2	0	2.3	1.9	2.3	1.7
Ints8	0	1.9	1.8	2.2	1.9
Cox8a	0	2.3	2.4	2.1	1.5
Zc3h15	0	1.4	1.3	1.1	1.3
Ccdc66	0	1.2	1.7	1.4	1.8
Tfb2m	0	1.2	1.2	1.4	1.3
Tmod3	0	1.6	1.4	1.7	1.3
Nutf2	0	2	2.1	1.4	1.2
Syncrip	0	1.4	1.3	1.2	1.2
Yme1l1	0	1.4	1.8	1.4	1.3
Sh2b3	0	1.4	1.2	1.4	1
Taf7	0	2.8	2.8	2.6	2.4
Xiap	0	1.1	1.3	1.4	1.4
Atp5j2	0	3	3.3	2.9	2.2
Tcf12	0	1.5	1.3	1	1.3
Csrp1	0	3.7	4.1	4.3	3.7
Atp5g2	0	1.8	2.1	1.6	1.6
Zfp612	0	2	1.9	1.9	2.1
Snrpd2	0	1.7	2.1	1.6	1.2
Extl3	0	1.3	1.9	1.9	1.4
Xrcc6bp1	0	1.9	2	2.1	1.3
Loxl2	0	1.9	2.5	2.8	2
Zfp160	0	1.1	1.2	1.6	2
Kif11	0	1.8	1.5	1.9	1
Mybpc1	0	1.5	2.1	3.3	3.3
Arpc3	0	2	2.3	2.9	1.3
Setd5	0	1.5	1.5	1.6	1.8
Lims1	0	1.4	1.1	1.6	1.4
Wdfy1	0	2.6	2.5	2.5	2.1
Arih1	0	1.4	1.2	1.5	1.2
Heatr6	0	1.4	1.3	1.4	1.1
Zfhx4	0	2.8	2	2.1	2.6
Plekhg2	0	1.2	1.5	1.3	1.2
Add3	0	2.4	2.4	3.2	3.1
Rasa1	0	1.1	1.1	1.4	1.6
Nradd	0	1.8	2	1.6	1.1
Cdk5rap2	0	1.5	1.7	1.6	1
Emcn	0	1.4	1.2	1.8	1.4
Nup160	0	1.1	1.1	1.2	1.1
Ifi202b	0	1.5	1.8	1.3	1.1
Ube2s	0	3.1	3.6	3.2	2.6
Glmn	0	1.3	1.4	1.4	1.3
Rsl1d1	0	1.6	1.7	1.5	1.4
Scarf2	0	1.7	2	1.6	1.4
Hoxa11as	0	2.6	3	2.4	2
Tnrc18	0	2.9	2.5	2.9	3
Hoxd12	0	4.5	5.4	4.7	2.9
Bzw2	0	1.7	1.6	1.4	1.2
Rian	0	1.8	1.7	2.1	2
Rlf	0	1.9	1.8	1.9	1.4
Ttc28	0	1.4	1.5	1.3	1.1
Reps1	0	1.3	1.6	1.4	1.2
Hyls1	0	3.1	2.8	3.1	2.6
Cenpi	0	1.2	1.2	1.2	1.1
0910001L09Rik	0	2.1	2.3	2	1.9
Stk11ip	0	1.9	2	1.4	1.3
Zfp64	0	1.1	1.7	1.6	1.2
Itga4	0	1	1.4	1.9	1.3
Col1a1	0	2.5	3.4	2.2	1.6
Cox6c	0	1.1	1.5	1.2	1.1
Stag3	0	1.5	1.8	1.8	2.2
Magoh	0	1.3	1.9	1.7	1.6
Snrpd3	0	1.7	1.5	1.9	1.1
Thbs1	0	1	1.5	1.2	1.4
Ergic1	0	1.8	2	1.8	1.2
Tra2b	0	1.7	1.6	1.3	1.4
Hoxa13	0	2.4	4	3.6	2.1
Thap1	0	1.7	2	1.8	1.4
Ogt	0	1.8	1.5	1.8	3
Pla2g4b	0	1.2	1.3	1.4	1.5
Med29	0	2.5	2.4	2.1	1.1
Meox2	0	1.5	2	1.7	1.1
Snrnp27	0	2	2.4	2	1.5
Fmr1	0	1.3	1.4	1.9	1.5
Snrpa	0	1.6	2.2	1.6	1.1
Cst3	0	1.8	2.1	1.6	1
Wdr70	0	1.4	1.6	1.8	1.2
Tmco6	0	1.8	1.8	1.5	1.2
Mrpl14	0	1.7	2	2.1	1.4
Npm3	0	3.6	3.8	3.7	2.5
Zfp868	0	1.5	1.8	1.5	1.6
Epha4	0	1.7	1.1	1.3	1.1
Rest	0	1.4	1.3	1.2	1.5
Elf2	0	2.1	1.8	1.8	1.3

Rps10	0	1.4	1.9	1.1	0
Ifnar1	0	1.1	1.2	1.1	0
Mrpl34	0	1.8	1.5	1	0
Gemin7	0	1.5	1.5	1.2	0
Edf1	0	2.3	2.3	1.7	0
Fgf10	0	1.8	1.7	1.4	0
L3mbtl3	0	1.6	1.2	1.3	0
Med30	0	1.4	1.6	1.3	0
2700062C07Rik	0	1.3	1.2	1.6	0
Tst	0	1.7	1.8	1.2	0
Rnase4	0	1.4	1.2	1.4	0
Fancm	0	1.8	1.5	1.7	0
Mpi	0	1.3	1.2	1.1	0
Mthfs	0	1.7	2.1	1.8	0
Haus2	0	1.6	1.7	1.2	0
Ube2l3	0	1.4	1.4	1.1	0
Mier1	0	1.6	1.6	1.7	0
Rab23	0	1.1	1	1.4	0
Nfkbil1	0	2	1.6	1.4	0
Nsmce4a	0	1.2	1.6	1.1	0
Fbxw2	0	1.5	1.5	1.3	0
Rasa2	0	1.1	1.1	1.4	0
Ndufs8	0	1.6	1.7	1.5	0
Sdf2l1	0	1.8	1.1	2	0
Shoc2	0	2.7	2.3	1.2	0
Ubxn2b	0	1.2	1.1	1.7	0
Pop7	0	1.2	1.3	1	0
Xpo5	0	1	1	1	0
Ppil2	0	1.5	1.7	1.1	0
Hspa12b	0	1.4	1.5	1	0
Sh3bgrl3	0	1.4	1.4	1.3	0
Ppig	0	1.5	1.4	1.2	0
Sgpl1	0	1.4	1.4	1.3	0
Ppfia1	0	1.4	1	1.1	0
Tbccd1	0	1.2	1.1	1.1	0
Malt1	0	1.7	1.9	1.8	0
Tceal8	0	1	1.3	1.1	0
Mms19	0	1.5	1.3	1.1	0
Lsmd1	0	1.1	1.6	1.3	0
Zfp7	0	1.1	1.2	1.3	0
Bbs9	0	1.2	1.5	1.2	0
Timm13	0	1.3	1.4	1.2	0
Acad11	0	1.5	1.4	1	0
Whsc1l1	0	1.3	1.1	1.3	0
Nudt2	0	1.6	2	1.5	0
Ccdc71	0	1.5	1.6	1.9	0
Zfyve1	0	1.6	1.6	1.6	0
Nol11	0	1.1	1.4	1.2	0
St3gal3	0	1.2	1.5	1	0
Gpt2	0	1.6	1.5	1.3	0
Pdk2	0	2.1	2.9	1.3	0
Raver1	0	1.9	1.9	1.2	0
Lars2	0	1.4	1	1.3	0
Elmod3	0	1.6	2	1.3	0
Gpr124	0	2.1	2	2	0
1810014F10Rik	0	1.7	2	1.4	0
Nfya	0	2.2	2.2	1.3	0
A930005H10Rik	0	1.2	1.3	1.5	0
Nek1	0	1.5	1.4	1.4	0
S100a6	0	1.5	2.1	1.3	0
Anxa4	0	1.4	1.6	1.4	0
Gjb6	0	2	1.6	1.6	0
Fau	0	1.5	1.8	1.4	0
Pibf1	0	1.3	1.5	1.3	0
Nupl2	0	1.1	1.2	1	0
Osr2	0	4.2	3.9	2.1	0
Wnt5a	0	1.4	1.7	1.3	0
Emd	0	1.3	1.3	1.2	0
Loxl1	0	1.2	2.3	1.1	0
G6pd2	0	2.9	2.2	2	0
Ganc	0	1.1	1	1	0
Atp5g1	0	1.3	1.6	1.4	0
Lysmd2	0	1.2	1.5	1.2	0
Slfn9	0	1.5	1.4	1.7	0
Psap	0	1.3	1.4	1.2	0
Lxn	0	1.9	1.6	1.1	0
Gm71	0	2.2	2.1	2.2	0
Bnip1	0	1.8	2.1	1.7	0
Ppp1r8	0	2.1	2	1.3	0
Cox6b1	0	1.5	1.6	1.6	0
Cenph	0	1.2	1.2	1	0
4930529C04Rik	0	2.3	1.9	2	0
Efna1	0	2	2.3	1.8	0
Hemk1	0	1.5	1.4	1.1	0
0610011F06Rik	0	1.2	1.3	1.1	0
Krtap16-7	0	1.5	1.5	1.3	0
4732418C07Rik	0	1.2	1.1	1.3	0
Itprip	0	1.2	1.5	1.1	0
Gchfr	0	1.9	2	1.6	0
Cav1	0	1.1	1.1	1.2	0
Eri3	0	1.9	2.1	1	0
2310009B15Rik	0	1.6	1.7	1.1	0
1500035H01Rik	0	1.6	1.5	1.4	0
4930428B01Rik	0	1.1	1	1.2	0
Atp1a1	0	1.6	1.7	1.4	0
Tmem126b	0	1.4	1.5	1.6	0
Ints9	0	1	1.4	1.1	0
Fam54a	0	1.7	1.6	1.4	0
Fam134c	0	1.6	1.2	1.1	0
E130303B06Rik	0	1.1	1.1	1	0
2900010M23Rik	0	1.2	1.3	1	0
Tomm7	0	1.2	1.2	1.1	0
Depdc7	0	1.8	2.1	2.1	0
Banf1	0	1.9	1.8	1.5	0
Ddx49	0	1.6	1.8	1.5	0
Imp3	0	1	1.3	1.2	0
Wbp5	0	1.3	1.6	1.3	0
Agk	0	1.2	1.1	1.1	0
2700078K21Rik	0	1.6	1.2	1.2	0
Tmem121	0	1.3	1.2	1	0
Trappc2l	0	1.5	1.3	1.5	0

Cdkal1	0	1.1	1.2	0	0
Sh2b2	0	1.2	1.3	0	0
Skp1a	0	1.2	1.3	0	0
Polr3c	0	1.2	1.4	0	0
Kdelr2	0	1.2	1.5	0	0
Slc25a13	0	1.2	1.2	0	0
Gnb2	0	1.6	1.9	0	0
Cnot7	0	1.1	1.1	0	0
Tnfrsf14	0	1.6	1.1	0	0
Dnajb3	0	1	1.1	0	0
Alms1	0	1.2	1	0	0
Pon3	0	1.2	1.4	0	0
Taf10	0	1.4	1.8	0	0
Ubl5	0	1.2	1.2	0	0
Btrc	0	1.6	1.7	0	0
Tacstd2	0	1.8	1.2	0	0
Zfp385a	0	1.2	1.2	0	0
Tcfap4	0	1.6	1.6	0	0
Krt15	0	1	1.5	0	0
Rtkn	0	1.5	1.6	0	0
Obfc1	0	1.1	1.3	0	0
Ipo9	0	1.1	1.1	0	0
Otor	0	1.1	1.7	0	0
Dync1li1	0	1	1.5	0	0
Mbtps1	0	1	1.3	0	0
Dhtkd1	0	1.2	1.2	0	0
Papss1	0	1.6	1.5	0	0
Mab21l1	0	1.9	1.6	0	0
Hnrnpa1	0	1.5	1.3	0	0
9330133O14Rik	0	1.2	1.8	0	0
Sf3b2	0	1.1	1.2	0	0
1110014N23Rik	0	1.1	1.3	0	0
Tmem132c	0	1.1	1.1	0	0
Siah1b	0	1	1.1	0	0
Kdelr1	0	1.3	1.6	0	0
Nrd1	0	1.5	1.4	0	0
Sec61b	0	1.1	1.3	0	0
Dhrs4	0	1	1.1	0	0
Acot11	0	1.1	1	0	0
Nt5c3l	0	1.2	1.7	0	0
Rbm38	0	1.3	1.4	0	-1
Vcam1	0	1.9	1.1	0	0
Vamp8	0	1	1.2	0	0
Arhgef1	0	1.2	1.5	0	0
Ppp4c	0	1.3	1.3	0	0
Zfp646	0	1.9	1.8	0	0
Pmm2	0	1.6	1.6	0	0
Pcnxl3	0	1.1	1.5	0	0
Ncor2	0	1.1	1.2	0	0
Irf2bp1	0	1.7	2	0	0
Rps9	0	1.2	1.5	0	0
Cir1	0	1.6	1.6	0	0
Tsc22d3	0	1	1.1	0	0
Mad1l1	0	1.5	1.5	0	0
Dci	0	1.1	1.1	0	0
Lamc1	0	1.3	1.4	0	0
Mpdu1	0	1	1.2	0	0
2810013P06Rik	0	1.2	1	0	0
Spag5	0	1.1	1.1	0	0
Rab36	0	1.1	1.2	0	0
Sart3	0	1.1	1	0	0
Atg2a	0	1.3	1.2	0	0
Cand1	0	1	1.1	0	0
Gstm5	0	1.2	1.3	0	0
Pccb	0	1.2	1.3	0	0
Clybl	0	1.9	1.3	0	0
Fam110b	0	1.1	1.4	0	0
H2afx	0	1.1	1.4	0	0
Pip4k2c	0	1.3	1.3	0	0
Phax	0	1.1	1.1	0	0
Nubp1	0	1.2	1.5	0	0
Terf2ip	0	1.1	1.1	0	0
Nynrin	0	1.9	2	0	0
Sdhc	0	1.8	2.1	0	0
Slc16a13	0	1.4	1.9	0	0
Spint2	0	1.2	1.5	0	0
Mgst3	0	1.8	2	0	0
9130604C24Rik	0	1.1	1	0	0
Irf2bp2	0	1.5	1.6	0	0
Sepn1	0	1.7	2.1	0	0
Phf12	0	1.6	1.3	0	0
Rhoa	0	1.1	1.2	0	0
Ndufb9	0	1.2	1.4	0	0
Scaf1	0	2.1	2.1	0	0
Tmem176a	0	1.2	1	0	0
Gm5506	0	1.2	1.6	0	0
Mrpl20	0	1.1	1.5	0	0
St3gal2	0	1.1	1.1	0	0
Hdac4	0	1.3	1.1	0	0
Sulf2	0	1	1.1	0	0
Ier2	0	1	1.1	0	0
Acy1	0	1.3	1.2	0	0
Tgfb3	0	1.2	1.1	0	0
Bcl2l12	0	1.1	1.3	0	0
Ppp1r15a	0	1.7	2.1	0	-1
Sh3gl1	0	1.2	1.4	0	0
Rnf123	0	1.5	1.4	0	0
Pm20d1	0	2.1	1.6	0	0
Fam101b	0	1	1.1	0	0
Mrpl12	0	1.3	1.2	0	0
Trib1	0	1.2	1.5	0	0
Mks1	0	1.1	1.3	0	0
Mllt1	0	1.6	1.4	0	0
Fam98b	0	1.2	1	0	0
Oaf	0	1.1	1.4	0	0
Csnk2b	0	1.5	1.4	0	0
Thop1	0	2	2.1	0	0
Usp11	0	1.6	2.2	0	0
Dctn2	0	1.2	1.4	0	0
Cdk16	0	1.3	1.5	0	0
Evc	0	1.2	1.5	0	0

Kbtbd3	0	0	0	1.4	0
Rapsn	0	0	0	1.1	0
Ttk	0	0	0	1.1	0
Prox1	0	0	0	1.1	0
Sytl2	0	-1	0	1	0
Ptgfr	0	0	0	1.1	0
Nup35	0	0	0	1.1	0
Il10rb	0	0	0	1.1	0
Hpgds	0	0	0	1	0
Slc26a7	0	0	0	1.5	0
1700012D01Rik	0	0	0	1.2	0
Hs3st3a1	0	0	0	1	0
Soat1	0	0	0	1.2	0
Myeov2	0	0	0	1.1	0
Mettl4	0	0	0	1.4	0
BC011248	0	0	0	1	0
Pdlim5	0	0	0	1.1	0
H2-L	0	0	0	1.1	0
Sycp3	0	0	0	1.2	0
Pde6d	0	0	0	1.2	0
Gnpda2	0	0	0	1.1	0
Ddit3	0	0	0	1.1	0
2610307P16Rik	0	0	0	1.2	0
Tbc1d23	0	0	0	1	0
Prr5l	0	0	0	1.1	0
Fgf7	0	0	0	1.5	0
4933411K20Rik	0	0	0	1	0
Herc1	0	0	0	1.2	0
Ccdc58	0	0	0	1.4	0
Mobkl1b	0	0	0	1.1	0
Bbs5	0	0	0	1.4	0
Ccdc127	0	0	0	1.3	0
Pcdh17	0	0	0	1.1	0
Rab28	0	0	0	1	0
Exph5	0	0	0	1.2	0
Cdc14b	0	0	0	1	0
Slc14a1	0	0	0	1	0
Wars2	0	0	0	1.1	0
Mfap3l	0	0	0	1.1	0
Orc1l	0	0	0	1.2	0
Ccdc18	0	0	0	1	0
Bivm	0	0	0	1.1	0
Med7	0	0	0	1.1	0
Hspa1b	0	0	0	1.7	0
L1td1	0	0	0	1.1	0
Tmem11	0	0	0	1	0
BC006779	0	0	0	1.1	0
Trpc1	0	0	0	1.5	0
Ncapd3	0	0	0	1	0
Ankrd29	0	0	0	1.6	0
Ube2v2	0	0	0	1	0
Med8	0	0	0	1	0
Spin4	0	0	0	1.1	0
Trip4	0	0	0	1.2	0
Snx13	0	0	0	1.3	0
Rabgef1	0	0	0	1.1	0
Ghr	0	0	0	1.3	0
Aimp2	0	0	0	1	0
Mrpl53	0	0	0	1.1	0
Dach1	0	0	0	1	0
Slc25a31	0	0	0	1.3	0
Alkbh7	0	0	0	1.1	0
Rev3l	0	0	0	1.2	0
Myot	0	0	0	1.2	0
Gnb1l	0	0	0	1	0
Rarres2	0	0	0	1.1	0
Agpat6	0	0	0	1	0
D330028D13Rik	0	0	0	1	0
Dguok	0	0	0	1	0
Kdm1b	0	0	0	1.1	0
Pigh	0	0	0	1.1	0
Nudt12	0	0	0	1.1	0
2610036L11Rik	0	0	0	1.1	0
Pde3a	0	0	0	1.1	0
3110035C09Rik	0	0	0	1.1	0
Rsu1	0	0	0	1	0
Txnip	0	0	0	1.1	0
Manf	0	0	0	1.1	0
Scrg1	0	-1	0	1.8	0
Setdb1	0	0	0	1.1	0
1810029B16Rik	0	0	0	1.2	0
Ankle2	0	0	0	1	0
Nat2	0	0	0	1	0
Slc35b4	0	0	0	1.1	0
A2m	0	0	0	1.2	0
Dusp14	0	0	0	1.3	0
Atl2	0	0	0	1.3	0
Tmem208	0	0	0	1.1	0
Hapln1	0	0	0	1.3	0
Ccdc56	0	0	0	1	0
Comp	0	0	0	1.2	0
C79407	0	0	0	1.2	0
Plk4	0	0	0	1.1	0
Ift80	0	0	0	1.3	0
Ap3b1	0	0	0	1	0
Gm106	0	0	0	1.3	0
9230114K14Rik	0	0	0	1.1	0
Fam82a1	0	0	0	1.2	0
Cops2	0	0	0	1	0

On E10, E12

Cluster 16 (51 genes)

	E9	E10	E11	E12	E13
Limd1	0	1.6	0	1	0
Ptpn18	0	1.1	0	1.2	0
Txndc11	0	1.1	0	1.4	0
Lztfl1	0	1.2	0	1.1	0
Zfp109	0	1.1	0	1.2	0
Zfp820	0	1.1	0	1.3	0
Id2	0	1.1	0	1.1	0
Jmy	0	1.2	0	1.4	0

Agpat2	1.1	0	0	0	0
Cth	1.2	0	0	0	-2
Yrdc	1.2	0	0	0	0
BB303372	1.1	0	0	0	-1
Maff	1.1	0	0	0	0
Fiz1	1.2	0	0	0	0
Jmjd5	1.3	0	0	0	-1
Scn2b	2.7	-2	-1	-2	-1
Il1rap	1.2	0	0	0	0
Trpm1	1.3	0	0	0	0
Edar	1.3	0	0	0	0
Prl2c2	1.6	0	0	0	0
Zdhhc7	1	0	0	-1	-1
Hoxb6	1.8	0	-2	-2	-2
Abcf2	1.1	0	0	0	0
Rspo2	1.9	0	-1	-1	0
Stbd1	1	0	0	0	-1
Serpine2	1.1	0	0	0	0
Kdm2b	1.2	0	0	0	0
Dmd	1.4	0	0	0	0
Hoxb5	2.1	0	-3	-4	-3
Src	1.5	0	0	-2	-2
Parvb	1.9	0	0	-1	-2
Cygb	1.1	0	0	0	0
Bcl7b	1	0	0	0	-1
Marcksl1	1	0	0	0	0
Crym	1.4	-2	-1	-1	-1
Ctsk	1.8	0	0	0	-1
Unc5b	1.2	0	0	-1	0
Fto	1	0	0	0	0
Plk1	1	0	0	0	0
Wars	1.2	0	0	0	0
Hdac9	1.2	0	0	0	0
Snord123	1.1	0	0	0	0
Cdx1	2.2	0	0	0	0
Mrps27	1	0	0	0	0
Dlx2	1	0	0	-1	-1
Kti12	1.1	0	0	0	0
Pqlc1	1	0	0	0	-1
Wbscr16	1.3	0	0	0	-2
Rnf150	1.4	0	0	0	0
Echdc1	1.2	0	0	0	0
Crispld1	1	0	0	0	0
Adprhl2	1	0	0	0	0
Mbd6	1.2	0	0	0	0
Tshz3	1	0	0	0	0
C030046I01Rik	1.6	0	0	-2	-2
Jag2	1.5	0	0	0	0
Tmem115	1.1	0	0	0	0
Sertad4	1.8	0	0	0	0
Ctu1	1.1	0	0	0	0
Tmem101	1.2	0	0	0	-1
Atp11b	1.6	0	0	0	0
Slc35a4	1.3	0	0	0	0
Igfbp4	1.3	0	0	0	0
Kcnk2	1	0	0	0	0
Sox18	1.2	0	0	0	0
Cdc42ep4	1	0	0	0	0
Gata5	1.2	0	0	0	0
Dgat2	1.2	0	0	0	-2
Lmo1	1.5	0	0	-2	-2
Ush1c	1.2	0	0	0	0
Alx1	1.6	0	-2	0	0
B630019K06Rik	1.2	0	0	0	-1
Sp6	1.4	0	0	0	0
1110029L17Rik	1.3	0	0	0	0
Mast3	1	0	0	-1	-1
Arfgap2	1.1	0	0	0	0
Hgsnat	1.1	0	0	0	0
Fam89a	1.6	0	0	0	-1
Krt23	1.3	0	0	0	0
Pth1r	1.1	0	0	0	-1
Fam102a	1.2	0	0	0	0
Ppm1g	1.4	0	0	0	0
Adamts18	1.2	0	0	0	0
Egr1	1.4	0	0	0	0
Homer3	1	0	0	0	0
Mpzl3	1.2	0	0	0	0
Fzd1	1	0	0	0	0
Eef2	1	0	0	0	0
Olfm1	1	0	0	0	-1
Actr1a	1	0	0	0	0
Mvd	1.1	0	0	0	0
Etv2	1.4	0	0	0	0
Abhd8	1.3	0	0	0	-2
Epha3	1.9	0	0	0	0
Bat1a	1.2	0	0	0	0
Fut4	1.1	0	0	0	0
Lrrc16a	1.1	0	0	0	0
Cd79b	1.1	0	0	0	0
Hoxa5	1.1	0	0	-1	0
Kctd10	1.1	0	0	0	0
Ruvbl2	1.1	0	0	0	0
Wnt7a	1.9	0	0	0	-1
Cd248	1.1	0	0	0	0
Tbl3	1.3	0	0	0	0
Slc18a1	1.1	0	0	0	0
Asns	1.1	0	0	-1	-2
Poll	1	0	0	0	-1
Ralgds	1.4	0	0	0	0
Tead2	1.2	0	0	0	0
Scn4b	2.4	0	0	0	0
Spry1	1.7	0	0	0	0
Ctbp2	1.1	0	0	0	0
Cldn7	1.2	0	0	-1	-3
Crtap	1.1	0	0	0	0
Dhrs3	1.1	0	0	0	0
Lgi2	1.2	0	0	0	0
Tmbim6	1.1	0	0	-1	-1
Gpr4	1	0	0	0	0
Sipa1	1.3	0	0	0	0

Ggcx	0	1.6	1.7	1.7	1.4
Hist2h3c1	0	1.3	1.6	1.8	1.2
Gabrp	0	1.5	2.6	2.5	2.5
Dpp8	0	1.6	1.5	1.4	1
Kctd12b	0	1.4	1.8	3	3
Ntf3	0	1.9	2.1	2	1.4
A530054K11Rik	0	1.9	2	1.5	1.6
Rprd1a	0	1.4	1.6	2	1.3
Bloc1s1	0	2.2	2.7	1.9	1.3
Id1	0	2.6	3.3	2.9	2.4
Tbc1d15	0	1.2	1.4	1.6	1
2900010J23Rik	0	2	2.2	2.5	2.6
Zfp862	0	1.7	1.9	1.9	1.4
2810055F11Rik	0	1.9	2.2	1.9	1.1
Pcm1	0	2	1.8	1.2	1.2
Plekha5	0	1	1	1.6	1.3
1200011I18Rik	0	2.3	2.3	2.5	1.8
Bbx	0	1.1	1	1.3	1.5
Gas1	0	1.2	1.2	1.1	1.2
Cbl	0	3	2.1	2.8	3.4
Dnm1l	0	1.1	1.1	1.3	1.1
Clk4	0	1.5	1.3	1.8	2.3
Efcab7	0	2	2.1	2.1	2.1
Rpl37a	0	2.4	2.8	2.2	1.6
Hist1h2ae	0	2.7	3.1	2.7	2.7
Sympk	0	1.9	2.6	2.1	1.5
Epm2aip1	0	1.4	1.3	1.6	1.7
Tcfcp2	0	1.4	1.3	1.3	1
Ccdc75	0	1.8	1.5	1.7	1.5
Eya4	0	2.1	2.1	2.4	2.2
Rpl17	0	2	2.2	1.7	1.5
Naa15	0	1.1	1.1	1.1	1.3
Magt1	0	1.8	1.5	2.5	2.2
Hipk3	0	2.9	3.1	3.6	2.6
Mrps11	0	1.8	2	1.9	1.5
Tbx18	0	2.7	2.5	2.2	2.4
6330416L07Rik	0	1.3	1.2	1.7	1.5
BC003267	0	1.5	1.4	1.7	1.3
Fbn2	0	1.9	2.1	2.1	2.4
Lsm12	0	1.8	1.6	1.4	1.1
Gsta4	0	1.4	1.6	1.4	1
Fam92a	0	1.2	1.5	1.6	1.6
Szt2	0	1.5	1.6	1.3	1
Hddc2	0	2.1	2.6	2.2	2
Aspm	0	1.5	1.1	1.3	1.1
Hnrnpl	0	2.7	2.8	1.5	1.7
Crkl	0	1.4	1.3	1.3	1
Supt16h	0	3.5	3.4	2.7	1.6
Rnf25	0	2.3	2.7	2.8	1.8
Twsg1	0	1.7	1.3	1.2	1.1
Pus10	0	1.6	1.3	1.2	1.6
Stk3	0	1.9	2.2	1.6	1.3
Gm9751	0	2.5	1.8	1.9	1.3
Prnd	0	2.8	2.7	2.1	1.5
Rpl26	0	1.2	1.9	1.3	1.1
Tctex1d2	0	2.1	2.2	2	1.8
Tubb2a-ps2	0	3.5	4.1	3.8	2.4
Hdac8	0	3.2	2.7	2.7	1
Commd6	0	2.1	2.4	2.3	1.2
Tex15	0	1.1	1.5	1.3	1
Nsa2	0	2	2.2	2	2.3
Cand2	0	2	2.2	1.6	1.5
Gpc2	0	2.7	2.4	1.1	1.4
Ap2b1	0	1.7	2	1.7	1.2
Cep350	0	1.6	1.5	2.1	1.6
Met	0	3.7	3.2	2.8	1.8
Bat2	0	2.5	2.4	1.4	1.7
Prcc	0	2.6	2.2	1.7	1.1
Tbl1x	0	2.1	2.4	1.5	1.6
Ing4	0	1.3	1.2	1.1	1
1110058L19Rik	0	1.6	2.4	1.8	1.4
Itgav	0	1.9	1.4	1.3	1
Lrig2	0	2.1	2.5	2.4	1.7
Ssb	0	1.1	1.3	1.1	1.3
Abhd1	0	1.2	1.3	1.1	1.3
Med21	0	1.5	2	2	1.5
Pum1	0	1.5	1.2	1.2	1.2
Pign	0	1.7	1.7	1.8	1.2
Snrpb2	0	2.6	2.8	2.5	2.3
Psmd7	0	1.5	1.4	1.1	1
1700081L11Rik	0	1.2	1.3	1.3	1.3
Ubl7	0	4.4	4.3	2.9	1.9
Gpbp1l1	0	1.8	1.7	1.4	1.1
Tnpo3	0	1.7	1.6	1.2	1.1
BC049807	0	1.1	1.1	1.4	1.4
0610009D07Rik	0	1.5	2	1.7	1.6
Pkn2	0	1.1	1.1	1.4	1.2
Gm13251	0	1.6	2.2	2.5	1.8
Spry2	0	1.5	1.7	1.4	1.4
Nedd4	0	1.8	1.9	1.4	1.6
Hoxa10	0	2.4	1.8	1.3	1.8
Ap4s1	0	1.8	1.9	1.9	1.4
Psma4	0	1.5	1.7	1.4	1.2
AI256396	0	2	1.7	1.6	1.4
Fam48a	0	1.1	1.3	1.2	1.4
Srcap	0	1.4	1.1	1.3	1.3
Tmsb15a	0	1.1	1.4	1.6	1.1
Zbtb12	0	3.6	4.3	3.9	3
BC023969	0	1.7	1.4	1.9	3.2
Arhgap10	0	2.5	2.4	1.6	1.2
Vcan	0	1	1.2	1	1.2
Usp9x	0	1.6	1.4	1.8	1.7
Elavl1	0	1.6	1.8	1.3	1.3
Sp4	0	2.2	1.8	2.3	2.4
Cdh11	0	1.6	1.9	1.3	1.3
Gorab	0	4	3.9	3.6	3
Zfp748	0	1.2	1.1	1.7	1.4
Ralgapa1	0	1.7	1.8	2.1	1.9
Rbm12b	0	1.5	1.3	1.5	1.7
Zfp187	0	1.9	1.5	2.2	1.9
Nrk	0	3.1	2.8	3.5	3.6

Cox7a2	0	1.1	1.5	1.3	0
Chst10	0	1.9	1.7	1.1	0
Zfp58	0	1.6	1.5	1.2	0
Trmt112	0	1.4	1.6	1.1	0
Rp2h	0	1.5	1.5	1.6	0
Bmyc	0	1.8	2.1	1.3	0
Hebp1	0	2	2.5	1.7	0
Prr5	0	2	2	1.9	0
Limk1	0	1.2	1.6	1.2	0
Cd97	0	1.5	1.2	1.1	0
5730528L13Rik	0	1.6	1.6	1	0
Taf1b	0	1.3	1.2	1.2	0
Cdhr4	0	1.3	1.3	1	0
Mfap4	0	2.2	2.1	1.8	0
Rhod	0	1.8	1.8	1.6	0
Polr2j	0	1.2	1.4	1.3	0
1810030O07Rik	0	1.1	1.3	1.2	0
Ppp1r11	0	1.8	1.6	1.4	0
Slc17a5	0	1.4	1.7	1.5	0
Crlf2	0	1.2	1.4	1	0
Gm10762	0	1.1	1.4	1.3	0
Gps2	0	2.2	2.5	1.4	0
Tmem9	0	1.8	2.4	1.4	0
Arpc1b	0	1.1	1.4	1.2	0
2010012O05Rik	0	1.4	1.6	1.4	0
Abcc10	0	1.4	1.4	1.1	0
Slc25a17	0	2.1	1.8	1.2	0
Bloc1s2	0	1.5	1.6	1.5	0
Dvl2	0	1.7	2.2	1.2	0
Xpa	0	1.2	1.4	1.2	0
Exo1	0	1.3	1.2	1.5	0
Mrpl52	0	1.4	1.6	1.1	0
D10Jhu81e	0	1.8	2.2	2	0
Dgkz	0	1.3	1.5	1.4	0
Tprkb	0	1.2	1.5	1.3	0
Pcp2	0	1.3	1.4	1.1	0
Ssr1	0	1.2	1.3	1	0
A430033K04Rik	0	1.5	1.4	1.6	0
Decr2	0	1.5	1.7	1.1	0
Cdk13	0	1.3	1.2	1.2	0
Cldn23	0	1	1.1	1.3	0
Pfdn4	0	1.5	1.5	1.3	0
Cd302	0	1.2	1.6	1.3	0
Stra13	0	2	2.3	1.6	0
Armc2	0	1.6	1.5	1.1	0
0610012G03Rik	0	1.1	1.3	1.1	0
Clic1	0	1.2	1.4	1.3	0
Brd2	0	1.4	1.5	1.1	0
Smc1a	0	1.4	1.4	1.1	0
Clip2	0	1.5	1.6	1.5	0
Nol9	0	1.2	1.3	1.1	0
Mylip	0	1.3	1.4	1.6	0
Clasp2	0	1.6	1.3	1.3	0
Pdap1	0	1.7	2.1	1.7	0
Pcdhb17	0	2.1	1.7	1.5	0
Rab18	0	1.3	1.5	1.3	0
Vpreb1	0	1.4	1	1	0
Tob1	0	1.2	1.5	1.4	0
Vps72	0	2.4	3.1	1.5	0
Snrpd1	0	1.3	1.5	1.3	0
Ifngr2	0	1.1	1.4	1.1	0
Znhit1	0	1.1	1.3	1	0
Ntn3	0	1.5	1.8	1.2	0
Pomp	0	1.1	1.4	1	0
Txlna	0	1.1	1.2	1.1	0
Ncstn	0	2.2	2.3	1.4	0
Pmf1	0	2.3	2.6	1.3	0
Kif2c	0	1.1	1.3	1.1	0
Gbe1	0	1.4	1.1	1	0
Psme1	0	1.6	1.3	1.2	0
Rabep2	0	1.9	2.2	1.6	0
Pof1b	0	1.2	1	1.6	0
Med11	0	1.1	1.4	1.3	0
Pdxdc1	0	1.2	1.4	1.1	0
Rpl36	0	1.1	1.5	1.1	0

Cluster 11 (405 genes)

Acan	0	0	1.5	0	0
Rpl35a	0	0	1.1	0	0
Mapk7	0	0	1.1	0	0
Lrrc4b	0	0	1.3	0	0
2610036D13Rik	0	0	1	0	0
6030426L16Rik	0	0	1.4	0	0
Zhx2	0	0	1.2	0	0
Fance	0	0	1	0	0
1110036O03Rik	0	0	1.4	0	0
Eif2b5	0	0	1.1	0	0
Gtf2ird1	0	0	1.1	0	0
Mrpl13	0	0	1	0	0
Rpl35	0	0	1.2	0	0
Plcd3	0	0	1	0	0
Tnfrsf22	0	0	1.1	0	0
Snrpg	0	0	1.5	0	0
Med17	0	0	1.1	0	0
Rpl21	0	0	1.1	0	0
Mrps26	0	0	1	0	0
Eif4ebp1	0	0	1.2	0	0
Tkt	0	0	1.1	0	0
Gabbr1	0	0	1	0	0
Hn1l	0	0	1	0	0
D630008O14Rik	0	0	1.4	0	0
Nol12	0	0	1.1	0	0
Rpl36al	0	0	1.1	0	0
Slc48a1	0	0	1	0	-1
Rbm22	0	0	1	0	0
Setd1b	0	0	1.4	0	0
Cbln4	0	0	1.4	0	0
Vps29	0	0	1.1	0	0
C1qc	0	0	1.2	0	-1
Rps21	0	0	1	0	0
Mad2l2	0	0	1.3	0	0

Prps1	0	1.3	1.1	0	0
Pnkd	0	1.1	1.2	0	0
Chmp1a	0	1.1	1	0	0
Ogfr	0	1.2	1.5	0	0
Anapc11	0	1.1	1	0	-1
Mfap2	0	1.6	1.7	0	0
Pgap3	0	1	1.3	0	0
Ucp2	0	1.2	1.5	0	-1
Fam108a	0	1	1.2	0	0
Serpinh1	0	1.1	1.5	0	0
Dysf	0	1.9	1.4	0	0
Plekho1	0	1.2	1.2	0	0
Napa	0	1.1	1.4	0	0
Thoc4	0	1.1	1.2	0	0
Tet2	0	1.3	1.4	0	0
Smc2	0	1	1	0	0
Cbx5	0	1.3	1.1	0	0
Lmo4	0	1.1	1.2	0	0
S100a11	0	2.3	2.7	0	0
Vps24	0	1.1	1.1	0	0
Tcf4	0	1.2	1.4	0	0
Rab1b	0	1.4	1.7	0	0
Erbb2	0	1.1	1.6	0	0
Cep76	0	1.3	1.2	0	0
Mta2	0	1	1	0	0
Cadm4	-1	1.7	2.1	0	0
Wibg	0	1.3	1.2	0	0
Atp6v1f	0	1.3	1.6	0	0
2510039O18Rik	0	1.3	1.5	0	0
Ypel1	0	1.2	1.3	0	0
Ndor1	0	1.3	1.3	0	0
Bcl11a	0	1.6	1.1	0	0
Ccni	0	1.3	1.1	0	0
Mpv17l2	0	1.7	1.5	0	0
2010317E24Rik	0	1.3	1.5	0	0
Tmsb10	0	1.7	2.2	0	0
Mmp11	0	1.4	1.6	0	0
Aaas	0	1.3	1.8	0	0
Prkar1a	0	1.3	1.3	0	0
Gpi1	0	1.4	1.4	0	0
4932415G12Rik	0	1.2	1	0	0
Frzb	0	1.8	1.5	0	0
Elmo3	0	1.1	1.2	0	0
Rag1	0	1.1	1.1	0	0
Pcgf2	0	1.8	1.6	0	0
Rab3il1	0	1.1	1.1	0	0
Dcakd	0	2.2	2.4	0	0
Efs	0	1.2	1.6	0	0
Asb13	0	1	1.2	0	0
Mab21l2	0	3.2	2.5	0	0
Cdkn2d	0	1.3	1	0	0
Pafah1b3	0	1.2	1.1	0	0
Nfix	0	1	2.1	0	0
Fam64a	0	1.1	1.3	0	0
Pcyt1a	0	1.3	1	0	0
Gjb2	0	2.3	1.7	0	0
Tmlhe	0	1.2	1.1	0	0
Sepx1	0	1.6	1.6	0	0
Zfp397	0	1.7	1.5	0	0
Mlf2	0	1.7	1.7	0	0
Clcn2	0	1.1	1.2	0	0
Chn1	0	1.4	1.1	0	0
1110054M08Rik	0	1.2	1.3	0	0
Acat3	0	1.4	1.4	0	0
Cnih2	0	1.4	1.2	0	-2
Cables2	0	1.2	1.1	0	0
Eri1	0	1	1	0	0
Grcc10	0	1.3	1.1	0	0
Gtf2f2	0	1.4	1.2	0	0
Map2k3	0	1.3	1.1	0	0
2210419D22Rik	0	1.8	1	0	0
Enpp5	0	1.5	1.6	0	0
Hmgb1	0	1.3	1.5	0	0
Ttc15	0	1.1	1.2	0	0
Ambra1	0	1.4	1.2	0	0
Sdccag8	0	1.5	1.4	0	0
Nle1	0	1.1	1.8	0	0
Nat6	0	1.1	1.1	0	0
Pcdhb20	0	1.5	1.4	0	0
C1qtnf4	0	1.4	2	0	0
Tomm22	0	1.2	1.5	0	0
Ndufaf3	0	1.5	1.4	0	0
Polr3gl	0	1.3	2	0	0
Stx6	0	1.4	1.5	0	0
Coq4	0	1.1	1.4	0	0
D1Bwg0212e	0	1.1	1.3	0	0
Poc1a	0	1.2	1.1	0	0
Thada	0	1.3	1.2	0	0
Dhx35	0	1.2	1.2	0	0
Rchy1	0	1.1	1.2	0	0
Casc4	0	1	1.6	0	0
Eif3c	0	1	1.1	0	0
Lrfn3	0	1.3	1.4	0	0
Cirbp	0	1.2	1.4	0	0
Slc1a4	0	1.6	1.1	0	0
Sf3a2	0	1.2	1.2	0	0
2700089E24Rik	0	1.2	1.1	0	0
Uhrf1	0	1.3	1.4	0	0
Bak1	0	1.7	2	0	0
Sh3bp5	0	1.3	1.4	0	0
Trim28	0	1.1	1.5	0	0
Sdc4	0	1.5	1.4	0	0
Mrps22	0	1	1.2	0	0
Elk3	0	1.2	1.2	0	0
Gabarap	0	1.7	1.4	0	0
Nosip	0	1.5	1.6	0	0
Col25a1	0	1.4	1.3	0	0
Eftud2	0	1	1.4	0	0
Grik5	0	1.3	1.3	0	0
Ier5l	0	1.5	1.6	0	0
Sox4	0	1.1	1	0	0

Atox1	0	1.1	0	1	0
Syce2	0	1.2	0	1	0
Stat1	0	1.1	0	1.3	0
Ccdc59	0	1.6	0	1.3	0
Wdyhv1	0	1.2	0	1.5	0
Arhgap18	0	1.5	0	1.1	0
AI956758	0	1.2	0	1	0
2810408I11Rik	0	1.2	0	1.1	0
Fbxo46	0	1.1	0	1.1	0
Rdh11	0	1	0	1	0
C1galt1	0	1.5	0	1.2	0
Map4k5	0	1.2	0	1	0
Mapk8ip1	0	1.2	0	1.4	0
Xbp1	0	1.2	0	1.6	0
Hira	0	1.2	0	1	0
Ankrd26	0	1.1	0	1.4	0
Chd1	0	1.2	0	1	0
Efna5	0	1	0	1.2	0
Pphln1	0	1.2	0	1.2	0
2700078E11Rik	0	1.3	0	1.1	0
Pi4k2b	0	1	0	1.3	0
Sqrdl	0	1.2	0	1.2	0
Sgms1	0	1.2	0	1.2	0
Zfp60	0	1.2	0	1.1	0
Hyal1	0	1.2	0	1.3	0
Rad52	0	1.1	0	1.2	0
Stag2	0	1.1	0	1.1	0
Ddx46	0	1.1	0	1.1	0
Ccdc117	0	1.5	0	1.6	0
Uggt2	0	1.1	0	1	0
Aff3	0	1.8	0	1.5	0
Usp37	0	1.2	0	1.1	0
Rnf6	0	1.1	0	1.3	0
Nr2c1	0	1.1	0	1.3	0
Atg16l2	0	1.3	0	1.1	0
Cpne3	0	1.2	0	1.1	0
Irf6	0	1.8	0	1	0
Mier3	0	1	0	1	0
Ttc37	0	1.4	0	1.9	0
Rsc1a1	0	1	0	1	0
Polr3f	0	1.1	0	1.1	0
Slc10a7	0	1.1	0	1.5	0
4732471D19Rik	0	1	0	1	0

Off 12

	E9	E10	E11	E12	E13
**Cluster 17 (35 genes)**					
Plxnd1	1.2	1.5	1.6	0	1.3
2610301G19Rik	1.2	2.6	2.2	0	1.2
Peg10	1.2	2.6	1.7	0	1
9430022A06Rik	1.1	1.6	1.5	0	1
Pdgfc	1.2	1.5	1.3	0	1.1
Ranbp9	0	1.3	1.1	0	1.3
Pacs2	0	1	1.3	0	1.2
A230072C01Rik	0	1.3	1.8	0	1.1
Csde1	0	1.1	1.3	0	1
Hsd17b7	0	1.1	1.1	0	1.2
Ankrd11	0	1.1	1	0	1.1
Hdac3	0	1.4	1.4	0	1
Arid1b	0	1.7	1.3	0	1.2
Itgb1	0	1.6	1.2	0	1.4
Sfrs2	0	1.3	1.4	0	1.1
Rnd3	0	1.5	1.2	0	1.1
Sik1	0	1.7	1.1	0	1.4
Grhl3	0	1.8	1.5	0	1.2
Arhgap32	0	1.2	1.1	0	1.3
Polr2k	0	1.3	1.3	0	1.4
Crnkl1	0	1.1	1	0	1.2
AU019823	0	1.6	1.4	0	1
Gnai2	0	1.7	1.5	0	1.1
Ube2q2	0	1.4	1.2	0	1.3
Acly	0	1.9	1.4	0	1.1
Arhgef10	0	1.4	1.4	0	1.2
Cep57	0	1.2	1.3	0	1.1
Igf2bp2	0	1	1	0	1.1
Cdk2	0	0	1	0	1.1
Ubtd2	0	0	1.2	0	1.4
Irs2	0	0	1.1	0	1.3
Foxp1	0	0	1.2	0	1.1
Gas2	0	0	1.2	0	1.2
Smtnl2	0	0	1.2	0	1
Ebf3	0	0	1.2	0	1.2

On E9 & E11

	E9	E10	E11	E12	E13
**Cluster 18 (22 genes)**					
Mpped2	1.2	0	1.2	0	0
Wtip	1.1	0	1.1	0	0
Ccnd3	1.4	0	1.3	0	0
Eefsec	1.3	0	1.5	0	0
Lrp10	1	0	1	0	0
Dok4	1.5	0	1	0	0
Evl	1	0	1.1	0	0
Lmf1	1.3	0	1.1	0	0
Nr1h2	1.2	0	1.1	0	0
Acpp	1.2	0	1	0	0
Eid1	1.2	0	1.3	0	0
Zfp36l2	1.1	0	1.1	0	0
Ptp4a3	1.6	0	1.3	0	-2
Wdr34	1.1	0	1	0	-1
Fscn1	1.2	0	1	0	0
Dcbld1	1	0	1.1	0	0
BC046404	1	0	1	0	0
Scamp4	1.1	0	1.1	0	0
Tarbp2	1.3	0	1	0	0
Pdgfa	1.6	0	1.3	0	0
Bambi	1.5	0	1.1	0	0
Kremen1	1.1	0	1.2	0	0

Tbx2	1.9	0	0	0	0
Ddx18	1.1	0	0	0	0
Gxylt2	1.1	0	0	0	0
Cmtm3	1.3	0	0	0	0
Selenbp1	1.4	0	0	0	-2
Perp	1.7	0	0	0	0

Enah	0	1.6	1.4	1.5	1.7
Zfp81	0	1.7	1.7	1.7	1
Naca	0	1.8	2.4	1.5	1.2
Atic	0	2.6	2.6	2.2	1.6
Pttg1	0	2.9	2.9	2.8	2
Pdia3	0	2.5	2.2	2.3	2.1
Wls	0	1.3	1.6	1.6	1.9
Hmgb2	0	1.9	2.3	1.7	1.5
Zfp2	0	3.2	2.8	1.9	1.3
Six1	0	1.2	1.6	1.1	1.5
P4ha1	0	1.5	1.1	1.7	1.3
Pik3r1	0	1.3	1.3	1.6	1.3
Pcdhb21	0	2.2	1.6	2	1.1
Bclaf1	0	1	1.2	1.2	1.4
Tmed7	0	1.2	1.2	1.4	1
Osr1	0	4.1	5.3	4.2	3.4
Fgfr1op2	0	1.2	1.5	1.2	1.3
Fhl3	0	1.2	1.3	1.4	1.7
Thoc2	0	1.2	1.1	1.6	2.2
Gyltl1b	0	1.1	1.7	1.5	1.3
Slc15a2	0	1.9	2	1.4	1.8
2810474O19Rik	0	1.7	1.6	1.7	1.4
Epha1	0	1.8	1.7	1.5	1
Gm11968	0	1	2	1.2	1.1
Bax	0	2.5	2.4	1.9	1.1
Ntan1	0	2	2.1	1.7	1.3
Fgf18	0	1.5	3.2	2.5	1.2
Zfp677	0	1.2	1.3	1.4	1.5
Rab5c	0	2.8	3	2.8	2.4
Acads	0	2.2	2.2	1.4	1.2
BC056474	0	3.8	4	2.1	1.3
E130311K13Rik	0	1	1.7	1.5	1.1
Ptbp1	0	3	3.3	2.9	2.7
Spopl	0	1.1	1.3	1.9	1.4
2010107E04Rik	0	1.7	1.8	1.7	1.2
Uqcr10	0	2.5	2.8	2.5	2.1
Tsen15	0	1.1	1.4	1.6	1.4
Hist1h2bp	0	2.6	3	2.9	1.8

Cluster 7 (73 genes)

Mtap1b	0	0	1.2	1.7	2.1
Ltbp1	0	0	1.6	1	1.6
Tomm5	0	0	1.2	1.2	1.3
Foxc1	-2	0	1.3	1.4	1.7
Ogn	0	0	3.1	2.7	2.1
Arpp21	0	0	1.9	2.1	1.3
2310015B20Rik	0	0	1.9	2	1.7
2610301B20Rik	0	0	1.1	1.5	1.6
Zadh2	0	0	1.3	1.8	1.5
Mylpf	0	0	2.1	2.3	1.5
Snhg1	0	0	1.6	2.1	1.8
Gdf5	0	0	3.7	3.1	2.4
Cry1	0	0	1.4	1.4	1
Zfp101	0	0	1	1.3	2.3
C530028O21Rik	0	0	1.2	1.4	1.1
Olfml2b	0	0	1.3	1.2	1
Epb4.1l3	0	0	1.3	1.3	1.5
Emp2	0	0	1.3	1.5	1.1
Aspn	0	0	1.2	2.9	1.8
Ccnl1	0	0	1.2	1.3	2.6
Wwp2	0	0	1.8	1.2	1
Ugp2	0	0	1.1	1.1	1.2
1190002N15Rik	0	0	1.2	1.4	1.3
Tgfbr3	0	0	1.4	1.7	2.3
Hs6st2	0	0	1.1	1.8	1.7
Nox4	0	0	1.3	1.1	1.1
Commd1	0	0	1.4	1.4	1.1
Fam103a1	0	0	1.3	1.2	1.1
Mybph	0	0	1.5	1.5	1.3
4930533K18Rik	0	0	1.2	1.6	1.3
Tgfbr2	0	0	1.3	1.6	1.1
Tmtc2	0	0	1.1	1.9	1.7
Sncaip	0	0	1.2	1.1	1.1
Uspl1	0	0	1.1	1.3	1
Mafb	0	0	1.3	1.1	1.1
Fndc1	0	0	1.2	2.2	1.3
Arhgef9	0	0	1.2	1.4	1.4
Egfr	0	0	1.2	1.3	1.1
Zfml	0	0	1.1	1.2	1.7
BC016423	0	0	1.4	1.4	1.4
Phex	0	0	1.3	2	1.9
Kera	0	0	4.3	3.7	3.1
1810035L17Rik	0	0	1.1	1.1	1.2
Nog	0	0	1.8	3	1.4
BC002059	0	0	1.3	1.5	1.8
Ngf	0	0	1.7	1.8	1
Zdhhc1	0	0	1.4	1.1	1.2
Nfia	0	0	1.3	1.2	1.3
Id3	0	0	1.4	1.3	1
BE687857	-1	0	1.8	2.1	1.2
Dleu2	0	0	1.1	2	2.1
Gpr27	0	0	1.3	1.8	1.1
Matn4	0	0	4.3	3.3	1.5
Ppp3r1	0	0	1.1	2.5	1.8
Tbc1d8	0	0	1.2	1.3	1.3
Slc16a4	0	0	1.6	1.6	1.2
Rgs16	0	0	1.4	1.4	1
1810022K09Rik	0	0	1.3	1.1	1.5
2310002L09Rik	0	0	1.3	1.7	1.2
Lgr5	0	0	1.1	1.2	1.1
Cdh6	0	0	1.4	1.5	1.8
Mustn1	0	0	1.4	2	1.4
Mef2a	0	0	1.3	1.4	1.8
Dkk2	0	0	1.9	1.7	2.5
Acbd3	0	0	1	1.3	1.2
Bnc2	0	0	1.2	1.1	1.4
Zfp867	0	0	1.6	1.3	1.3
Chchd1	0	0	1.4	1.3	1.3
2310001H12Rik	0	0	1	1.4	1.1
Scfd1	0	0	1.2	1.2	1.1
Neb	0	0	2	1.8	1.5

Btbd2	0	0	1.3	0	0
Prune	0	0	1	0	0
Rhobtb1	0	0	1.2	0	0
Rps19	0	0	1.2	0	0
Kcnmb4	0	0	1.7	0	0
Myo1c	0	0	1	0	0
Fam63a	0	0	1	0	0
Rpl19	0	0	1.4	0	0
Rps12	0	0	1.4	0	0
Tk2	0	0	1.3	0	0
Zfp783	0	0	1.1	0	0
Hdac11	0	0	1.1	0	0
1810049H13Rik	0	0	1.1	0	0
Rsrc2	0	0	1.1	0	0
2610528E23Rik	0	0	1	0	0
Lrrc61	0	0	1.1	0	0
Sult5a1	0	0	1.6	0	0
Ap2a1	0	0	1.1	0	0
Lepre1	0	0	1.3	0	0
Lamb3	0	0	1.2	0	0
Strn4	0	0	1	0	0
Rps25	0	0	1.2	0	0
Btd	0	0	1.1	0	0
Rps5	0	0	1	0	0
Smarce1	0	0	1	0	0
Camk1g	0	0	1.2	0	0
Urm1	0	0	1.1	0	0
Pir	0	0	1.1	0	0
Ctdsp2	0	0	1.2	0	0
Wdr59	0	0	1.1	0	0
Dgcr6	0	0	1.1	0	0
Ehd3	0	0	1	0	-1
Rpl10a	0	0	1	0	0
Rsl24d1	0	0	1.3	0	0
Hsp90ab1	0	0	1.1	0	0
Gas2l1	0	0	1.1	0	0
Crls1	0	0	1.1	0	0
Rab40c	0	0	1.1	0	0
Gtpbp3	0	0	1.1	0	0
Egfl6	0	0	1.6	0	0
3110056O03Rik	0	0	1.2	0	0
Rps4y2	0	0	1.1	0	0
Bai2	0	0	1.7	0	0
Thap3	0	0	1.3	0	0
Rpl11	0	0	1	0	0
Cpt1a	0	0	1.1	0	0
Elof1	0	0	1.1	0	0
Zfp90	0	0	1.2	0	0
Fbxw5	0	0	1.1	0	0
E4f1	0	0	1.1	0	0
Pcdhb19	0	0	1.1	0	0
Rps27l	0	0	1.3	0	0
Fam195b	0	0	1	0	0
BC037039	0	0	1	0	0
Txndc9	0	0	1.1	0	0
Plvap	0	0	1.1	0	0
Iscu	0	0	1	0	0
Trappc9	0	0	1.1	0	0
Nhp2l1	0	0	1.1	0	0
2310014H01Rik	0	0	1	0	0
Adamts7	0	0	1.2	0	0
Ccdc104	0	0	1.3	0	0
Fntb	0	0	1.4	0	0
Ncl	0	0	1.2	0	0
0610009O20Rik	0	0	1.2	0	0
Zranb1	0	0	1	0	0
Lipa	0	0	1.1	0	0
Lactb2	0	0	1.1	0	0
Dgcr14	0	0	1.1	0	0
Cyb5	0	0	1.1	0	0
Rnf167	0	0	1.2	0	0
Col9a3	0	0	2.3	0	0
1110004F10Rik	0	0	1	0	0
Mapk13	0	0	1	0	0
Dusp23	0	0	1	0	0
Rpl27a	0	0	1.3	0	0
Ckap4	0	0	1.3	0	0
Mif	0	0	1.1	0	0
Rcvrn	0	0	1	0	0
Pias3	0	0	1	0	0
Neil1	0	0	1	0	0
Stt3a	0	0	1	0	0
Hexim2	0	0	1.3	0	0
Sesn2	0	0	1	0	0
Hmg20b	0	0	1.9	0	0
Rpl7a	0	0	1	0	0
Glyr1	0	0	1	0	0
1810009N02Rik	0	0	1	0	0
Gm166	0	0	1	0	0
Cad	0	0	1.1	0	0
Mccc2	0	0	1.1	0	0
Hic1	0	0	2	0	0
Mterfd2	0	0	1	0	0
Man2a2	0	0	1.2	0	0
Ndufa11	0	0	1.1	0	0
Rac1	0	0	1	0	0
S100a13	0	0	1.4	0	0
2010002M09Rik	0	0	1.2	0	0
Wdr6	0	0	1.2	0	0
Eif3d	0	0	1.3	0	0
Kri1	0	0	1.2	0	0
Col6a2	0	0	1.7	0	0
Ube2e3	0	0	1.1	0	0
Txndc17	0	0	1	0	0
Xylt2	0	0	1.6	0	0
Ubc	0	0	1.2	0	0
Ntm	0	0	1.1	0	0
C1qa	0	0	1.3	0	0
Tsen34	0	0	1.2	0	0
Osbpl5	0	0	1.2	0	0
Pef1	0	0	1.3	0	0

Alad	0	1.1	1.2	0	-1
Arnt	0	1.1	1	0	0
Dcun1d4	0	1.2	1.2	0	0
Pld6	0	1.3	1.4	0	0
Inpp5e	0	1	1.1	0	0
Ppme1	0	1.3	1	0	0
Efnb1	0	1.6	1.4	0	0
Ube2i	0	1.1	1.2	0	0
Lmbr1l	0	1.2	1.3	0	0
Hsp90ab1	0	1	1.2	0	0
Tbc1d13	0	1.5	1.9	0	0
Rhog	0	1.3	1.6	0	0
Men1	0	1	1.3	0	0
U2af2	0	1.1	1.6	0	0
Jag1	-1	1.2	1.5	0	0
Efna3	0	1.5	1.2	0	0
4833420G17Rik	0	1	1.1	0	0
Ube2n	0	1.9	1.9	0	0
Tsc2	0	1.6	1.3	0	0
Igsf9	0	1.1	1.8	0	0
Dhps	0	1.4	1.6	0	0
Sirt2	0	1	1.2	0	0
Reep5	0	1.3	1.4	0	0
Kif26b	0	1.7	1.5	0	0
4930422N03Rik	0	1.1	1.5	0	0
Emg1	0	1.2	1.5	0	0
Btf3l4	0	1	1.1	0	0
Srebf2	0	1.5	1.4	0	0
Arhgdib	0	1.1	1.1	0	0
Aldh18a1	0	1.7	1.9	0	0
Unc45a	0	1.2	1.1	0	0
Atp13a1	0	1.1	1.3	0	0
Echs1	0	1.1	1.3	0	0
Ahcy	0	1	1.2	0	0
Rftn2	0	1.4	1.2	0	0
Bri3	0	1	1.6	0	0
Auts2	0	1.6	1.2	0	0
Ncoa4	0	1.1	1.3	0	0
Scpep1	0	1	1.4	0	0
Pla2g12a	0	1.8	1.3	0	0
Cib1	0	1.5	1.3	0	0
Runx2	0	2	1.6	0	0
Ttc1	0	1.1	1	0	0
Pfdn1	0	1.5	1.8	0	0
Tgfb1	0	1.5	1.5	0	0
Ino80b	0	1.6	1.5	0	0
Podxl2	0	1.9	1.6	0	0
Adi1	0	1.2	1.3	0	0
AV099323	0	1.2	1.5	0	0
2610306M01Rik	0	1.4	1.4	0	0
Triobp	0	1.1	1.5	0	0
Myh9	0	1.3	1.4	0	0
Igsf3	0	1.4	1.3	0	0
Rasl11a	0	1.5	1.2	0	0

Cluster 13 (190 genes)

Gm13032	0	1.1	0	0	0
Ttpa	0	1	0	0	0
Hrc	0	1	0	0	0
Nmnat1	0	1.2	0	0	0
Hhat	0	1.1	0	0	0
Cars	0	1.1	0	0	0
Tmem177	0	1	0	0	0
Pcdhb3	0	1	0	0	0
Pik3r3	0	1.4	0	0	0
Galnt2	0	1	0	0	0
Zfat	0	1.1	0	0	0
Dkk1	0	1.1	0	0	0
Rdh12	0	1	0	0	0
Kif3b	0	1.1	0	0	0
Neu1	0	1.1	0	0	0
Stat6	0	1.4	0	0	0
Lsg1	0	1.1	0	0	0
Zik1	0	1.2	0	0	0
Bmp1	0	1.2	0	0	0
Cast	0	1	0	0	0
Bcl7c	0	1.2	0	0	0
Dpyd	0	1.2	0	0	0
Mtus1	0	1.5	0	0	0
Ncaph2	0	1	0	0	0
Elmo2	0	1.1	0	0	0
Fam46a	0	1.5	0	0	0
Eif5b	0	1	0	0	0
Ogdh	0	1	0	0	0
Peg3	0	1	0	0	0
Ash2l	0	1.2	0	0	0
Gimap4	0	1.1	0	0	0
Gabarapl2	0	1.2	0	0	0
Irx3	0	1	0	0	0
Padi3	0	1.7	0	0	0
Rqcd1	0	1.1	0	0	0
Exosc2	0	1	0	0	0
BC017612	0	1.4	0	0	0
Scube1	0	1.3	0	0	0
Senp1	0	1.1	0	0	0
Coro1a	0	1.1	0	0	-2
Trappc3	0	1.2	0	0	0
Tm9sf2	0	1.1	0	0	0
Mrps18a	0	1.1	0	0	0
Ccng2	0	1	0	0	0
Adam10	0	1.1	0	0	0
Vav2	0	1.2	0	0	0
Tmem120a	0	1.2	0	0	0
Epb4.9	0	1.1	0	-1	-3
Wdr5b	0	1	0	0	0
Mov10	0	1.1	0	0	0
Rgl3	0	1.2	0	0	0
Peli1	0	1.1	0	0	0
Myst4	0	1.1	0	0	0
Lbh	0	1.1	0	0	0
Dock10	0	1.7	0	0	0

On 10&13

	E9	E10	E11	E12	E13
**Cluster 19 (20 genes)**					
Anapc1	0	1	0	0	1
Spag1	0	1.2	0	0	1.2
Pja2	0	1	0	0	1
Gpc6	0	1.3	0	0	1.3
Dhcr24	0	1.8	0	0	1.7
Pcmtd1	0	1	0	0	1.3
Senp6	0	1.1	0	0	1.4
Rbm9	0	1.1	0	0	1.1
Suz12	0	1.2	0	0	1.1
Ramp2	0	1.7	0	0	2.4
Eml4	0	1.3	0	0	1.2
Kdm5b	0	1	0	0	1.1
4933439C10Rik	0	1.3	0	0	1.4
D3Wsu106e	0	1.1	0	0	1.7
Tnfaip6	0	1.3	0	0	1
Huwe1	0	1.1	0	0	1.1
Zfp644	0	1	0	0	1.2
Ubn1	0	1.1	0	0	1.1
Rhobtb3	0	1	0	0	1.2
Zfp445	0	1.5	0	0	1.2

Off E9 & E11

	E9	E10	E11	E12	E13
**Cluster 20 (24 genes)**					
Map3k7	0	1.2	0	1.4	1.3
Dnajc3	0	1.2	0	1.7	1.1
Ccdc132	0	1.3	0	1.3	1
Arid4b	0	1.2	0	1.3	1
Sfrs13a	0	1.2	0	1.2	1.3
Gatad2b	0	1.5	0	1.6	2
Gja5	0	1.1	0	2.1	1.3
Fam165b	0	1	0	1.6	1.1
Frmd6	0	1.4	0	1.4	1.1
Mtmr4	0	1.1	0	1.2	1.3
Prex2	0	1.2	0	1.2	1.4
AI314180	0	1	0	1.1	1
7630402I04Rik	0	1.8	0	1.5	1.6
Lpgat1	0	1.3	0	1.3	1.2
Zfp516	0	1.4	0	1.3	1.5
Zfp191	0	1.2	0	1.1	1.3
Klhl4	0	1.3	0	1.2	1.2
Zfp157	0	1.1	0	1.1	1.1
Sfrs12	0	1	0	1.2	1.4
Zfp40	0	1.2	0	1.3	1.8
Sdc2	0	1.1	0	1.4	1.2
Ror1	0	1.2	0	1.1	1
Zc3h11a	0	1	0	1	1.3
Rgl1	0	1.5	0	1.1	1

Unique patterns

	E9	E10	E11	E12	E13
**(4 genes)**					
Hoxc5	2.1	0	0	1.6	1.5
Rdh10	1.2	0	0	0	1.1
Fam198b	1.3	1.2	0	1.2	0
Hoxa9	2	1.3	0	0	1.4

Anpep	0	0	1.4	1.4	1.1
Sema3d	0	0	1.9	2.1	1.4

Cluster 8 (77 genes)

Lrch3	0	0	0	1	1
Igfbp5	-1	0	0	2.7	2.9
Smyd1	0	0	0	1.5	1.5
Tmem47	0	0	0	1.4	1.2
Papolg	0	0	0	1.2	1
Gnptab	0	0	0	1	1.2
Ccl21b	0	0	0	1.8	1.6
Serpinb7	0	0	0	1.4	1.3
Rif1	0	0	0	1.2	1.7
Hivep2	0	0	0	1.6	1.8
Fam73a	0	0	0	1.1	1.5
Hspb3	0	-1	0	1.4	1.3
Gpx8	0	0	0	1.1	1
Ctdspl2	0	0	0	1.5	1.4
A430108E01Rik	0	0	0	1.2	2.4
Heatr5a	0	0	0	1.5	1.2
AI503316	0	0	0	1	3.7
Rhoq	0	0	0	1.3	1.1
Airn	0	0	0	1.1	1.5
D4Wsu53e	0	0	0	2	1.6
B930025B16Rik	0	0	-1	1	2.2
Angpt1	0	0	0	1.2	1
Myom2	0	0	0	1.8	2.1
Ttn	-2	-3	0	2	2.1
Zfp354c	0	0	0	1.1	1.2
Evi5	0	0	0	1.3	1.1
Igf1	0	0	0	1.2	1.5
Hspb2	0	-1	0	1.6	1.3
Arhgap20	0	0	0	1.3	1
Lepr	0	0	0	1.2	1.9
Slc30a4	0	0	0	1.1	1
Adam12	0	0	0	1.3	1.3
Esyt2	0	0	0	1.2	1.1
Stac3	0	0	0	1.3	1.2
Dnm3os	0	0	0	1.5	4.8
Flrt2	0	0	0	1.2	2
5230400M03Rik	0	0	0	1.1	1.8
March7	0	0	0	1	1.4
Dgcr8	0	0	0	1.2	1.1
Cenpe	0	0	0	1.2	1.1
Enpp2	0	0	0	1.2	1.3
Prr16	0	0	0	1.3	2
Nt5e	0	0	0	1.4	1.5
2810403A07Rik	0	0	0	1.2	1.7
Xpo1	0	0	0	1.1	1.3
Pcdhb16	0	0	0	1	1.2
6430704M03Rik	0	0	0	1.1	1.2
Pnmal2	0	0	0	2	1.8
Atrnl1	0	0	0	1.1	1.1
Nespas	0	0	0	1.9	2.2
Ddx26b	0	0	0	1.2	1.9
Tbc1d4	0	0	0	1.1	1.1
Igf2r	0	0	0	1.1	1.3
Prdm5	0	0	0	1.2	1.6
Zfp758	0	0	0	1.8	1.1
Ppp1r3c	0	0	0	1.9	1.7
Hip1	0	0	0	1.1	1.4
Pds5a	0	0	0	2.2	1.9
Far1	0	0	0	1.1	1.5
Cep192	0	0	0	1.2	1.3
Narg2	0	0	0	1.3	1.4
2700023E23Rik	0	0	0	1.9	2.8
Gpr153	0	0	0	1.4	1.1
Slc4a7	0	0	0	1.8	3
Smpx	-4	-3	0	1.7	1.5
A930038C07Rik	0	0	0	1.1	1.3
Taf15	-1	0	0	1.9	2.1
Rpl39l	0	0	0	1.6	1.6
5730408K05Rik	0	0	0	1.1	1.2
Tmx3	0	0	0	1.4	1.2
Gpr64	0	0	0	1.5	1.5
Tceal7	0	0	0	1.9	1.4
1110059M19Rik	0	0	0	1.9	1.6
Zfp185	0	0	0	1.4	2.1
Iqgap1	0	0	0	1.2	1
Sln	0	0	0	1.9	2.5
Matn3	0	0	0	1.9	1.2

Cluster 9 (200 genes)

2210403K04Rik	0	0	0	0	1.1
6030400A10Rik	0	0	0	0	1.1
4833423F13Rik	0	0	0	0	1.1
1110006E14Rik	0	0	0	0	2.8
Usp28	0	0	0	0	1.1
Guf1	0	0	0	0	1.2
Dzip3	0	0	0	0	1.2
Sdpr	-1	0	0	0	1.2
Sltm	0	0	0	0	1.1
Rhoj	0	0	0	0	1.3
Meg3	0	0	-1	0	1.7
Ppargc1b	0	0	0	0	1.1
9630010G10Rik	0	0	-2	0	2.3
Cep110	0	0	0	0	1.1
Gas5	0	0	0	0	3.6
D630030B22Rik	0	0	0	0	1.1
Scai	0	0	0	0	1.3
Caprin2	0	0	0	0	1.3
5830418K08Rik	0	-1	-1	0	1.8
6330417A16Rik	0	0	0	0	1.4
Ccdc39	0	0	0	0	2.4
Ccdc52	0	0	0	0	1
Ankrd16	0	0	0	0	1.8
Lime1	0	0	0	0	1
Hcfc2	0	0	0	0	1
6030451C04Rik	0	0	0	0	2.1
Ptprv	0	0	0	0	1.2
D430033H22Rik	0	0	0	0	2.1
Gng13	0	0	1	0	0
Mest	0	0	1	0	0
Sirpa	0	0	1	0	0
Iqcb1	0	0	1.1	0	0
Casp3	0	0	1.1	0	0
Nudt22	0	0	1.1	0	0
Tpcn1	0	0	1.1	0	0
0610007C21Rik	0	0	1	0	0
Rab11fip2	0	0	1.1	0	0
Trp53i13	0	0	1.2	0	0
Ube2o	0	0	1	0	-1
Nr2c2ap	0	0	1.1	0	0
Lsm1	0	0	1	0	0
Rplp0	0	0	1.2	0	0
Nfkbib	0	0	1.1	0	0
Hps4	0	0	1.1	0	0
Cwc25	0	0	1.3	0	0
H2afy	0	0	1	0	0
Gclm	0	0	1.3	0	0
Smarcd3	0	0	1.2	0	0
Fgf9	0	0	1.1	0	0
Prkab1	0	0	1.3	0	0
Nop2	0	0	1.2	0	0
Mrps23	0	0	1.1	0	0
Clcf1	0	0	1.4	0	0
1110021L09Rik	0	0	1	0	0
Sap30l	0	0	1.3	0	0
Pgrmc1	0	0	1.1	0	0
Rps18	0	0	1.5	0	0
Timm17a	0	0	1	0	0
Rfesd	0	0	1.2	0	0
5430416N02Rik	0	0	1.1	0	0
Ncf4	0	0	1.6	0	0
Sarnp	0	0	1.2	0	0
Avpi1	0	0	1.4	0	0
Mrpl40	0	0	1.1	0	0
Rad51l1	0	0	1	0	0
Sec31a	0	0	1.2	0	0
Sfrp2	0	0	1.7	0	0
Sssca1	0	0	1.2	0	0
Rplp2	0	0	1.1	0	0
Cenpb	0	0	1.1	0	0
Arhgap17	0	0	1.2	0	0
Prelid1	0	0	1.1	0	0
Rpl7l1	0	0	1	0	0
Nsmce1	0	0	1.4	0	0
Tradd	0	0	1.1	0	0
Mrpl41	0	0	1.1	0	0
Pdlim2	0	0	1.1	0	0
Fgfbp1	0	0	1.3	0	0
Tle3	0	0	1.2	0	0
Jdp2	0	0	1.4	0	0
Hook2	0	0	1.1	0	0
Fnip2	0	0	1.1	0	0
Col8a1	0	0	1.6	0	0
Gprc5c	0	0	1	0	0
Gstm7	0	0	1.3	0	0
Rps14	0	0	1.1	0	0
Npm1	0	0	1	0	0
Sertad1	0	0	1.2	0	0
Hdhd3	0	0	1.1	0	0
Mrpl17	0	0	1	0	0
Phactr2	0	0	1.1	0	0
Rpl13a	0	0	1.1	0	0
Rfk	0	0	1.1	0	0
Mxd4	0	0	1	0	0
Psmd9	0	0	1	0	0
H1f0	0	0	1.4	0	0
S100a10	0	0	1	0	0
S1pr1	0	0	1	0	0
9430038I01Rik	0	0	1.1	0	0
Morf4l1	0	0	1	0	0
Ttc5	0	0	1	0	0
H3f3a	0	0	1	0	0
Psma7	0	0	1.1	0	0
Cpped1	0	0	1.1	0	0
Tesk1	0	0	1.1	0	0
Smo	0	0	1.2	0	0
2310008H09Rik	0	0	1.2	0	0
Adrm1	0	0	1	0	0
Nbl1	0	0	1.5	0	0
Anapc2	0	0	1.1	0	0
Zfp637	0	0	1	0	0
Slc43a1	0	0	1	0	0
Rpl29	0	0	1.3	0	0
Foxp4	0	0	1.1	0	0
2700060E02Rik	0	0	1	0	0
BC002163	0	0	1.1	0	0
Cited4	0	0	1	-2	-3
Lypd6b	0	0	1.7	0	0
Hspb6	0	0	1.3	0	0
Slc2a10	0	0	1.2	0	0
Lfng	0	0	1	0	0
Atxn7l3	0	0	1.1	0	0
Cul9	0	0	1.1	0	0
H2-D1	0	0	1	0	0
Ubb	0	0	1	0	0
Plod1	0	0	1.3	0	0
Mea1	0	0	1.1	0	0
Nucb1	0	0	1.1	0	0
Hsd3b6	0	0	1.3	-1	-1
Amacr	0	0	1.1	0	0
Dbp	0	0	1.2	0	0
Tulp1	0	0	1.1	0	0
Bend5	0	0	1	0	0
Chd3	0	0	1	0	0
Refbp2	0	0	1	0	0
Zcchc3	0	0	1.1	0	0
Sat1	0	0	1.1	0	0
Tcp11l2	0	0	1.5	0	0
Inadl	0	0	1.2	0	0
Nek3	0	1	0	0	0
Pafah1b2	0	1.1	0	0	0
Tes	0	1	0	0	0
Arhgap28	0	1.4	0	0	0
Capns1	0	1.1	0	0	0
Ifi204	0	1.1	0	0	0
Srp72	0	1.2	0	0	0
Wipi2	0	1	0	0	0
Glrb	0	1.1	0	0	0
Pcyt1b	0	1.2	0	0	0
Slc29a4	0	1.2	0	0	-1
Gfm2	0	1.3	0	0	0
Cul3	0	1.1	0	0	0
Hsd17b12	0	1.2	0	0	0
Bcl11b	0	1.4	0	0	0
Gm9918	0	1	0	0	0
Tyro3	0	1.1	0	0	0
Crhbp	0	1.1	0	0	0
Shb	0	1.1	0	0	0
Pbx1	0	1.4	0	0	0
Cul5	0	1.1	0	0	0
Nln	0	1.1	0	0	0
Junb	0	1.1	0	0	0
Grk5	0	1.6	0	0	0
4930455C21Rik	0	1.4	0	0	0
Gm9897	0	1.1	0	0	0
Slitrk6	0	1.3	0	0	0
Nckap5l	0	1.1	0	0	0
Lrp12	0	1.1	0	0	0
Lcp1	0	1.1	0	0	0
Arid3a	0	1.6	0	0	0
Cbr2	0	2.8	0	0	0
Piga	0	1.2	0	0	0
Mesdc1	0	1	0	0	0
Sap130	0	1.2	0	0	0
Gypa	0	1.2	0	-3	-5
Cd40	0	1.4	0	0	0
Ergic2	0	1	0	0	0
Becn1	0	1.2	0	0	0
Npc1	0	1.1	0	0	0
Rabl5	0	1.2	0	0	0
Mup10	0	1.4	0	0	0
Copg2	0	1	0	0	0
Cdkn2aip	0	1	0	0	0
Ryr1	0	1.1	0	0	0
Atf7ip2	0	1	0	0	0
Fam129c	0	1.4	0	0	0
2810019C22Rik	0	1	0	0	0
Ppp2r5b	0	1.1	0	0	0
Phka1	0	1.1	0	0	0
Arhgap24	0	1.1	0	0	0
5830417I10Rik	0	1.1	0	0	0
Ptrf	0	1.1	0	0	0
Gpcpd1	0	1	0	0	0
Zic2	0	1.6	0	0	0
E2f4	0	1.1	0	0	0
Mfsd9	0	1	0	0	0
Asb9	0	1.1	0	0	0
Cd8b1	0	1.2	0	0	0
Lpar1	0	1.2	0	0	0
Zfp763	0	1.2	0	0	0
Phkg2	0	1	0	0	0
Zfp418	0	1.1	0	0	0
Tmem2	0	1	0	0	0
Fchsd2	0	1	0	0	0
Arl6ip5	0	1	0	0	0
4930534B04Rik	0	1.1	0	0	0
Apoc1	0	1.1	0	0	-1
2610002D18Rik	0	1.1	0	0	0
Pde4dip	0	1.1	0	0	0
Vps39	0	1.2	0	0	0
2410131K14Rik	0	1.1	0	0	0
Ccnt1	0	1.4	0	0	0
Kpna3	0	1	0	0	0
Fancd2	0	1	0	0	0
Bdh1	0	1	0	0	-1
Mras	0	1	0	0	0
Zfp68	0	1.1	0	0	0
Sost	0	1.7	0	0	0
A030001D20Rik	0	1.1	0	0	0
1110032A04Rik	0	1.9	0	0	0
Myo10	0	1.3	0	0	0
Map3k11	0	1.1	0	0	0
Ube2a	0	1.1	0	0	0
Agpat1	0	1.2	0	0	0
Kdm4b	0	1.1	0	0	0
Samd14	0	1.2	0	0	0
Sacm1l	0	1.1	0	0	0
1110020G09Rik	0	1.1	0	0	0
Galnt7	0	1.1	0	0	0
Banp	0	1.3	0	0	0
Micall2	0	1.1	0	0	-2
Slit3	0	1.2	0	0	0
Hoxd4	0	1.7	0	0	0
Lrrc57	0	1.1	0	0	0
Gng2	0	1.1	0	0	0
Serpine1	0	1	0	0	0
Stard3nl	0	1.1	0	0	0
Luzp1	0	1.1	0	0	0
Fis1	0	1.2	0	0	0
2410024N18Rik	0	1.2	0	0	0
Stau1	0	1.1	0	0	0
Naa40	0	1.1	0	0	0
Hsf2	0	1	0	0	0
Hax1	0	1	0	0	0
Atf2	0	1.1	0	0	0
Itpkc	0	1.1	0	0	0
Zbtb5	0	1.1	0	0	0
Fblim1	0	1	0	0	0
2310067B10Rik	0	1	0	0	0
Gorasp2	0	1.1	0	0	0

Prdm2	0	0	0	0	1
2810055G20Rik	0	0	0	0	1.4
9530086O07Rik	0	0	0	0	1.6
Dse	0	0	0	0	1.3
C230037E05Rik	0	0	0	0	1.7
1700012D14Rik	0	0	-1	0	1
D2Ertd173e	0	0	0	0	2.8
Nktr	0	0	0	0	1.4
9430047G12Rik	0	0	0	0	1.6
Chodl	0	-2	-2	0	1.3
Gls	0	0	0	0	1.2
Ccnt2	0	0	0	0	1.4
Ptcd3	0	0	0	0	1
D19Ertd409e	0	-2	-2	0	1.4
Luc7l3	0	0	0	0	1
D5Ertd505e	0	0	0	0	1.1
Ttc14	0	0	0	0	2.3
C130057M05Rik	-1	-1	-1	0	1.5
Cxcl13	0	-1	0	0	1.7
AA415038	0	0	0	0	1.3
Rgag4	0	0	0	0	1.1
AI428301	0	0	0	0	1.3
Dep1	0	0	0	0	1.3
A130004G07Rik	0	0	0	0	1
Kdm3a	0	0	0	0	1
4632427E13Rik	-1	-2	-3	0	1.6
Malat1	0	0	0	0	2.2
5830415B17Rik	0	0	0	0	1.1
C80068	0	0	0	0	1.1
D5Ertd798e	0	0	0	0	2.4
Golga1	0	0	0	0	1
5830458C19Rik	0	-1	-1	0	1.5
Scml2	0	0	0	0	1.1
2310067E19Rik	0	0	0	0	2.6
C80142	0	0	0	0	2.1
Irx5	0	0	0	0	1
Chrnb1	0	0	0	0	1.2
D030041H20Rik	0	0	0	0	1.3
Mtmr1	0	0	0	0	1.3
6720462K09Rik	0	-1	-2	0	1.4
AI605517	0	0	0	0	1.3
BC022960	0	0	0	0	1.2
9930017N22Rik	0	0	0	0	1.7
Irx2	-1	0	0	0	1.2
5830474E16Rik	0	0	0	0	2.3
Rtl1	0	0	0	0	2.4
2610319H10Rik	0	-1	-2	0	1.7
BB211804	0	0	0	0	1.3
Ebf2	-1	0	0	0	1.1
Zbed6	0	0	0	0	2.2
2610311E24Rik	0	0	0	0	1.8
Top2a	-1	-1	-1	0	1.5
D130084N16Rik	0	0	0	0	2
Med13l	0	0	0	0	1.1
Atp11a	0	0	0	0	1
Eif4a2	0	0	0	0	1.1
6430537K16Rik	0	0	0	0	3.4
2810405F17Rik	0	0	0	0	1.2
Wsb1	0	0	0	-1	1.1
Srrm2	0	0	0	0	1.4
2410042D21Rik	0	0	0	0	1.1
4930422I07Rik	0	0	0	0	1.1
5830407P18Rik	0	0	0	0	1.8
Nfat5	0	0	0	0	1.4
Robo2	0	0	-1	0	1.4
Moxd1	0	0	0	0	1.1
AU041975	0	0	0	0	1.1
D330040H18Rik	0	0	0	0	2.2
4930518I15Rik	0	0	0	0	1
2310003F16Rik	0	0	0	0	2.8
Gm2590	0	0	-2	0	1.9
Phf20l1	0	0	0	0	1.1
Serinc4	0	0	0	0	1
2610042L04Rik	0	0	0	0	1.4
Mll3	0	0	0	0	1.2
Rbm39	0	0	0	0	2.3
Zim1	0	0	0	0	1.1
Pax7	0	0	0	0	1.3
Fndc3c1	0	0	0	0	1.5
9430076C15Rik	0	0	0	0	1.1
5430406J06Rik	0	0	0	0	1.9
2810043O03Rik	0	0	0	0	3.6
D5Wsu152e	0	-2	-2	0	1.7
B230117O15Rik	-1	0	0	0	1.1
Krit1	0	0	0	0	1.3
Rnf122	0	-1	-1	0	1.4
E030016H06Rik	0	0	0	0	2.5
Zfp826	0	0	0	0	1.9
Gm9159	0	0	0	0	1.3
Gm15241	0	0	0	0	2
Tslp	0	0	0	0	1.2
C77673	0	0	-1	0	1.6
Ms10w	0	-2	-2	0	1.8
5031426D15Rik	0	0	0	0	1.5
9530029O12Rik	0	0	-2	0	1.6
Egln3	0	0	0	0	1.1
Mdm4	0	0	0	0	1.1
Tpsab1	0	0	0	0	1.1
Fbn1	0	0	0	0	1.2
Ythdf3	0	0	0	0	1.3
B830007D08Rik	0	-2	-3	0	1.1
Cox8b	0	0	0	0	1.2
Sobp	0	0	0	0	1.2
Hmcn1	0	0	0	0	1.3
Spata1	0	0	0	0	1.2
Zmynd11	0	0	0	0	1
AU042873	0	-1	-1	0	1.4
AI131651	0	0	0	0	1.4
Wipf1	0	0	0	0	1.2
Fam82b	0	0	0	0	1
D030002E05Rik	0	0	0	0	1.5
Tspan18	0	0	1.4	0	0
Tab2	0	0	1	0	0
Ddx24	0	0	1.3	0	0
Mettl5	0	0	1.1	0	0
AI414330	0	0	1.2	0	0
Rpl34	0	0	1	0	0
Snx21	0	0	1.2	0	0
Psmb1	0	0	1.1	0	0
Nek6	0	0	1.4	0	0
Polr2d	0	0	1	0	0
Mrto4	0	0	1	0	0
Gm10244	0	0	1.3	0	-2
Cyb5r3	0	0	1.1	0	-1
Nup188	0	0	1.1	0	0
Tcf19	0	0	1.1	0	0
C1qb	0	0	1.2	0	-1
Plec	0	0	1.2	0	0
Ltbp3	0	0	1	0	0
Palmd	0	0	1.1	0	0
Fbxo17	0	0	1.5	0	0
Rpl38	0	0	1.4	0	0
Rassf7	0	0	1	0	0
Exosc4	0	0	1.1	0	0
Itfg3	0	0	1	0	0
Wiz	0	0	1.1	0	0
Rad23a	0	0	1.1	0	-1
Rpl23a	0	0	1.2	0	0
Mettl1	0	0	1.4	0	0
Ndufv2	0	0	1.1	0	0
Ttc17	0	0	1.2	0	0
Riok2	0	0	1.1	0	0
Gorasp1	0	0	1.1	0	0
Gatc	0	0	1.1	0	0
Frmpd1	0	0	1	0	0
Nxnl2	0	0	1	0	0
Rpl13	0	0	1.6	0	0
Rps17	0	0	1.5	0	0
Calhm2	0	0	1.3	0	0
Myog	0	0	2.4	0	0
Caps2	0	0	1	0	0
Atp5d	0	0	1	0	0
Ptma	0	0	1.2	0	0
Armcx6	0	0	1.3	0	0
Gm16517	0	0	1	0	-1
Supt7l	0	0	1.1	0	0
Sirt5	0	0	1	0	0
Pdcd5	0	0	1.1	0	0
Atp2a1	0	0	1.3	0	0
Rgs19	0	0	1.1	0	0
Slc25a39	0	0	1.3	0	0
Fxyd5	0	0	1	0	0
Bbs4	0	0	1.1	0	0
Srebf1	0	0	1.2	0	0
Fadd	0	0	1.2	0	0
Nenf	0	0	1.2	0	0
Ptov1	0	0	1.2	0	0
Arhgef17	0	0	1	0	0
Gngt2	0	0	1.1	0	0
Rrp7a	0	0	1.1	0	0
Ddt	0	0	1	0	0
Lect1	0	0	1.6	0	0
Mapk8ip3	0	0	1.2	0	0
Gapdh	0	0	1.1	0	0
2400001E08Rik	0	0	1	0	0
AI894139	0	0	1	0	0
2310016C08Rik	0	0	1.1	0	0
1300018J18Rik	0	0	1.1	0	0
Spata6	0	0	1.3	0	0
Gm7325	0	0	1.8	0	0
Gadd45gip1	0	0	1.1	0	0
Gipc1	0	0	1.4	0	0
Ciapin1	0	0	1.1	0	0
Ppp2r1a	0	0	1.6	0	0
1500001M20Rik	0	0	1	0	0
Col6a1	0	0	1.4	0	0
Btg1	0	0	1.1	0	0
Cstb	0	0	1.1	0	0
4933400F03Rik	0	0	1.4	0	0
Zfp13	0	0	1	0	0
Fasn	0	0	1.1	0	0
Ephb6	0	0	1.2	0	0
Hist1h1e	0	0	1.1	0	0
Mrps35	0	0	1.1	0	0
Taf9	0	0	1.1	0	0
Rpl23	0	0	1.4	0	0
Creg1	0	0	1.1	0	-1
Nat15	0	0	1.1	0	0
Cnbp	0	0	1.1	0	0
Rpl7	0	0	1.3	0	0
Scara3	0	0	1.6	0	0
Mre11a	0	0	1.1	0	0
Glce	0	0	1	0	0
Ly6c1	0	0	1.8	0	0
Capn1	0	0	1.1	0	0
Mrpl54	0	0	1	0	0
Mtx1	0	0	1	0	0
Ppia	0	0	1	0	0
Man2c1	0	0	1.1	0	0
Ssu72	0	0	1.2	0	0
Sorbs3	0	0	1.3	0	0
Gm5914	0	0	1.2	0	0
Akap1	0	0	1.1	0	0
Efcab2	0	0	1.1	0	0
Ypel3	0	0	1.3	0	0
Comtd1	0	0	1	0	0
Pabpc4	0	0	1.1	0	0
H13	0	0	1	0	0
Sgk1	0	0	1.1	0	0
Rpl24	0	0	1.2	0	0
Zbtb8a	0	0	1.3	0	0
Rfc4	0	0	1	0	0
Nudcd3	0	1.3	0	0	0
Bre	0	1	0	0	0
Obfc2b	0	1.3	0	0	0
Cbln1	0	1.3	0	0	-2
3010026O09Rik	0	1	0	0	0
Msl2	0	1.3	0	0	0
Acadl	0	1	0	0	0
Slc25a30	0	1.3	0	0	0
Sae1	0	1	0	0	0
Zfyve26	0	1	0	0	0
Dlg4	0	1.1	0	0	0
Mxi1	0	1.2	0	0	0
Maneal	0	1	0	0	0
Wdr33	0	1	0	0	0
Zbtb45	0	1.2	0	0	0
Ulk4	0	1.3	0	0	0
Sh3d19	0	1.3	0	0	0
Txndc16	0	1	0	0	0
Vrk2	0	1	0	0	0
Clasp1	0	1.2	0	0	0
Dr1	0	1.1	0	0	0
AI464131	0	1.1	0	0	0
Senp2	0	1.2	0	0	0
Gpx2	0	1.8	0	0	0

AW549877	0	0	0	0	1.6
Dmtf1	0	0	0	0	1
Jmjd1c	0	0	-1	0	1
C530014P21Rik	0	0	-2	0	1.9
Rbm25	0	0	0	0	1.6
4432414F05Rik	0	0	0	0	1.6
Fbxl3	0	0	0	0	1.2
D130023J23Rik	0	-1	-2	0	1.2
4932431P20Rik	0	0	0	0	1
6430537I21Rik	0	0	0	0	1.1
Clk1	0	0	0	0	1.3
Gm7890	0	0	0	0	1.7
1700094D03Rik	0	0	0	0	1.2
Zc3h7a	0	0	0	0	1
Zfp788	0	0	0	0	1.2
D530037H12Rik	0	-1	-2	0	1.2
4833414E09Rik	0	0	0	0	1.5
A630026N12Rik	0	0	0	0	1.1
Zcchc7	0	0	0	0	1.3
A630033H20Rik	0	0	0	0	1.3
Smek2	0	0	0	0	1.1
6430590A07Rik	0	0	0	0	1.8
Rbm5	0	0	0	0	1.3
Adamts6	0	0	0	0	1.9
Itih5	0	0	0	0	1
Pvrl3	0	0	0	0	1.4
C130075A20Rik	0	0	-2	0	1.4
Fubp1	0	0	0	0	1.4
AI451458	0	0	-1	0	1.4
E330013P04Rik	0	0	0	0	1.8
Lphn2	0	0	0	0	1
Boc	0	0	0	0	1.1
Rbbp4	0	0	-2	0	2.1
Sfrs18	0	0	0	0	1.8
Zfp760	0	0	0	0	1.2
6720420G18Rik	0	0	-1	0	1.1
Ccnl2	0	0	0	0	1.5
Akap9	0	0	0	0	1.2
9630030I15Rik	0	0	0	0	1.7
9930031P18Rik	0	0	-2	0	1.7
Scara5	0	0	0	0	1.2
2610011E03Rik	0	0	0	0	1.4
Rbm26	0	0	0	0	1.3
Eif2ak4	0	0	0	0	1
2700099C18Rik	0	0	0	0	1
BB046190	0	0	0	0	1.2
Rnpc3	0	0	0	0	1.1
AU018552	0	0	0	0	1.1
Gcfc1	0	0	0	0	1.8
Zfp280d	0	0	0	0	1.4
Dock4	0	0	0	0	1.1
Mpp5	0	0	0	0	1
Ankrd2	0	0	0	0	1.2
9430047L24Rik	0	0	0	0	1.8
Zfp207	0	0	0	0	1
Smc6	0	0	0	0	1
Mirhg1	-1	-1	-2	0	2.9
4732423E21Rik	0	0	-2	0	1.7
2010111I01Rik	0	0	0	0	1.1
9430085L16Rik	0	0	0	0	1.2
Abi3bp	0	0	0	0	1.3

Efemp2	0	0	1.2	0	0
E430018J23Rik	0	0	1.1	0	0
Cnn2	0	0	1	0	0
Pabpc1	0	0	1.1	0	0
Sf3b4	0	0	1.3	0	0
Dapk2	0	0	1.2	0	0
Vash2	0	0	1	0	0
Epb4.1	0	0	1.1	0	0
Fam162a	0	0	1.1	0	0
Vegfb	0	0	1.3	0	0
Dcaf8	0	0	1.1	0	0
Isg20l2	0	0	1.1	0	0
Gpx1	0	0	1	0	-1
Rps29	0	0	1.2	0	0
Gm5577	0	0	1.5	0	0
Tmem138	0	0	1.1	0	0
Cttn	0	0	1.1	0	0
Ahsp	0	0	1.1	-2	-4
Efha1	0	0	1.3	0	0
Ltbp4	0	0	1.6	0	0
Il11ra1	0	0	1.2	0	0
Arhgap1	0	0	1.1	0	0
Plcb3	0	0	1.1	0	0
Tcfe3	0	0	1.1	0	0
Dnajc19	0	0	1	0	0
5730559C18Rik	0	0	1.2	0	0
Sfrs13b	0	0	1.8	0	0
Tpst2	0	0	1.1	0	0
Cyth1	0	0	1.3	0	0
St8sia6	0	0	1	0	0
Ctnnb1	0	0	1.1	0	0
Mmab	0	0	1.1	0	0
Itfg2	0	0	1.4	0	0
Dazap1	0	0	1.5	0	0
Chmp6	0	0	1.1	0	0
Rpl22	0	0	1.3	0	0
Dullard	0	0	1.2	0	0
Tpm1	0	0	1.5	0	0
