# Supplementary material for: Global Gene Expression Analysis of Murine Limb Development
Source: PLoS One. 2011 Dec 9;6(12):e28358. doi: 10.1371/journal.pone.0028358 (PMC3235105; doi:10.1371/journal.pone.0028358)
Supplement: Table S4 — Stage-specific enriched genes. (PDF) [file pone.0028358.s007.pdf]

Table S4. Stage-specific enriched genes. The putative function, expression pattern and KO phenotype of each transcript was examined in Pubmed, MGI (http://www.informatics.jax.org/phenotypes.shtml), and Visigene (http://genome.ucsc.edu/cgi-bin/hgVisiGene). For genes with KO mice available, the most descriptive reference is cited (see References S1). Abbreviations: Musculoskeletal (MSK), Yes (Y), Normal (N), Not available (NA), embryonic lethal prior to limb formation (EL), anterior-posterior (AP), proximal-distal (PD), Knockout (KO), double knockout (dKO), embryonic day (E). Novel genes are highlighted in grey and genes with confirmed MSKs are in brown. All other genes have normal or no MSK KO phenotypes. **in situs* available in Figure 6. References cited are included in References S1.

		E9.5 FL						
**No.**	**Gene Symbol**	**logFC**	**Adj. p-val**	**Putative Function**	**KO phenotype**	**Ref**	**In situ**	**MSK Function**
1	Ruvbl2	1.07	2.07E-04	novel	unknown		NA	
2	Ctbp2	1.08	5.22E-06	Transcription factor	perinatal lethal by E10.5	[141]	NA	
3	Eef2	1.00	1.50E-05	Translation factor	unknown		NA	
4	Igfbp4	1.31	2.28E-05	growth factor	impaired growth	[142]	Y	N
5	Mrps27	1.03	3.90E-04	mitochondrial gene	unknown		NA	
6	Cd79b	1.06	3.35E-06	antigen	immune defect; no reporter limb phenotype	[143]	NA	N
7	Fzd1	1.02	8.12E-11	wnt signaling	no phenotype	[144]	NA	N
8	Prdm16	1.25	9.59E-07	ion binding	craniofacial/ossification defect	[145]	Y	Y
9	Larp1	1.01	9.07E-05	novel	unknown		NA	
10	Ralgds	1.37	1.17E-07	enzyme regulation	malignancy	[146]	NA	N
11	Tshz3	1.05	5.90E-09	transcription factor	kidney, smooth muscle defects	[147]	NA	N
12	Nploc4	1.09	4.81E-06	novel	unknown		NA	
13	Yrdc	1.18	3.11E-05	novel	unknown		NA	
14	Lgi2	1.25	6.45E-08	novel	unknown		NA	
15	Khsrp	1.76	8.04E-04	mRNA processing	unknown		NA	
16	Xkr5	1.09	1.41E-04	novel	unknown		NA	
17	Etv2*	1.38	2.87E-05	Transcription factor	perinatal lethal by E10.5, abnormal heart and vascular	[148]	Y	EL
18	Actr1a	1.02	3.14E-04	cytoskeleton	unknown		NA	
19	Sp6	1.43	3.07E-10	Transcription factor	syndactyly/oligodactyly	[149]	Y	Y
20	Tmem59l	1.27	9.33E-10	novel	unknown		NA	
21	Dhrs3	1.12	7.16E-06	metabolism	unknown		NA	
22	Tmem115	1.07	3.23E-05	transmembrane protein	unknown		NA	
23	Egr1	1.38	1.50E-05	Transcription factor	low bone mass	[150]	Y	Y
24	Ppif	1.11	1.85E-06	metabolism	nervous system defects, no reported limb phenotype	[151]	NA	N
25	Gpr4	1.05	1.22E-07	transmembrane protein	cardiovascular defects, no reported limb phenotype	[152]	NA	N
26	Ptpn9	1.09	2.06E-07	phosphatase	abnormal bone development	[153]	NA	Y
27	Bat1a	1.16	4.81E-05	novel	unknown		NA	
28	Arfgap2	1.15	3.57E-05	novel	unknown		NA	
29	Zfp771	1.04	1.52E-04	Transcription factor	unknown		NA	
30	E130012A19Rik	2.45	1.98E-04	novel	unknown		NA	
31	Plxnb2	1.12	2.71E-05	transmembrane protein	perinatal lethal by E9.5	[154]	NA	EL
32	Btbd11*	1.94	7.20E-15	novel	unknown		Y	
33	Pycr1	1.03	1.70E-04	novel	unknown		NA	
34	Cmtm3	1.28	6.05E-06	novel	unknown		NA	
35	Hdac9	1.20	1.29E-07	chromatin	cardiac hypotrophy	[155]	NA	N
36	Ssbp3	1.24	6.33E-07	Transcription factor	skeletal defects	[156]	NA	Y
37	Tbc1d7	1.14	6.56E-05	novel	unknown		NA	
38	Otud3	1.29	6.80E-07	novel	unknown		NA	
39	Epha3	1.93	1.33E-08	receptor	cardiovascular defects, no reported limb phenotype	[157]	NA	N
40	Hgsnat	1.07	5.93E-13	novel	unknown		NA	

41	Fcer1g	1.22	2.57E-08	receptor	limb and skeletal defects in complex phenotype	[158]	NA	Y
42	Ddx41	1.18	2.85E-04	novel	unknown		NA	
43	Antxr1	1.01	3.86E-09	receptor	craniofacial/skeletal defects	[159]	NA	Y
44	Csk	1.61	2.58E-05	signal transduction	perinatal lethal by E10.5	[160]	Y	EL
45	Cyth2	1.17	6.62E-06	novel	unknown		NA	
46	Fiz1	1.19	1.68E-04	Transcription factor	unknown		NA	
47	Prl2c2	1.60	4.28E-07	novel	unknown		NA	
48	Crtap	1.07	9.91E-06	cartilage associated	skeletal/limb defects	[161]	NA	Y
49	Perp	1.68	1.76E-09	apoptosis	no reported limb phenotype	[162]	NA	N
50	C1qtnf2	1.05	2.50E-05	novel	unknown		NA	
51	Cd248	1.14	3.59E-04	antigen	no reported limb phenotype	[163]	NA	N
52	1110029L17Rik	1.26	1.14E-04	novel	unknown		NA	
53	Il17rd	1.02	5.08E-07	receptor	nervous system	[164]	Y	N
54	Marcksl1	1.04	9.08E-04	unknown	postnatal lethality; neural defects	[165]	NA	N
55	Atp11b	1.57	1.81E-04	ATP binding	unknown		NA	
56	Zgpat	1.04	1.33E-05	transcription factor	unknown		NA	
57	Rnf150	1.44	1.28E-11	novel	unknown		NA	
58	Zyx	1.24	8.42E-04	cytoskeleton	no reported limb phenotype	[166]	NA	N
59	Casd1	1.09	1.84E-12	novel	unknown		NA	
60	Tmem54	1.26	1.95E-07	transmembrane protein	unknown		NA	
61	Smarcd2*	1.24	5.46E-08	chromatin	unknown		Y	
62	Serpine2	1.08	1.08E-08	enzyme regulation	nervous system defects, no reported limb phenotype	[167]	Y	N
63	Sertad4	1.78	1.06E-13	novel	unknown		NA	
64	Slc18a1	1.08	5.61E-10	transmembrane protein	normal		NA	N
65	Wars	1.17	5.94E-05	novel	unknown		NA	
66	Pik3r2	1.14	2.95E-04	metabolism	metabolic defect	[168]	NA	N
67	AA667102	1.09	1.20E-05	novel	unknown		NA	
68	Agpat2	1.12	5.10E-05	enzyme regulation	postnatal lethal/metabolism	[169]	NA	N
69	Ppp5c	1.19	1.87E-05	enzyme regulation	normal	[170]	NA	N
70	Krt23	1.30	4.88E-06	cytoskeleton	unknown		NA	
71	Scn4b	2.44	7.99E-13	sodium channel	unknown		NA	
72	Myc	1.17	6.94E-05	apoptosis	perinatal lethal by E10.5/growth size	[171]	NA	EL
73	Trmt1	1.01	1.93E-04	novel	unknown		NA	
74	Cdx1	2.17	1.03E-11	Transcription factor	skeletal defects	[172]	Y	Y
75	Tbl3	1.29	0.000396	novel	unknown		NA	
76	Kdm2b	1.17	1.09E-09	novel	unknown		NA	
77	Homer3	1.03	1.20E-04	GTP binding	no reported limb phenotype	[173]	NA	N
78	Enox1	1.13	8.15E-06	novel	unknown		NA	
79	Sipa1	1.30	2.01E-05	GTPase	immune defect; no reporter limb phenotype	[174]	NA	N
80	Tcfap2c*	1.88	2.30E-10	Transcription factor	perinatal lethal by E10.5	[175]	Y	EL
81	Apcdd1	1.19	6.39E-11	wnt signaling	unknown		Y	
82	Ctu1	1.06	3.04E-06	novel	unknown		NA	
83	Fut4	1.09	4.65E-08	metabolism	immune defects, no reported limb phenotype	[176]	NA	N
84	Fam110a	1.31	9.97E-04	novel	unknown		NA	
85	Kcnk2	1.04	1.27E-06	potassium channel	nervous system defects, no reported limb phenotype	[177]	NA	N
86	Snord123	1.06	7.77E-08	novel	unknown		NA	
87	Maff	1.08	1.53E-07	DNA binding	normal	[178]	Y	N
88	S1pr2	1.12	1.12E-06	novel	nervous system defects, no reported limb	[179]	NA	N
89	Adamts18	1.20	6.60E-06	extracellular matrix	unknown		NA	
90	Mfsd10	1.24	6.92E-04	novel	unknown		NA	
91	Tead2*	1.18	3.46E-04	Transcription factor	Tead1/2 dKO embryonic lethal by E9.5	[180]	Y	EL
92	Il1rap	1.15	4.17E-12	receptor accessory	normal	[181]	NA	N
93	Mbd6	1.19	6.14E-04	novel	unknown		NA	

94	Col14a1	1.57	5.07E-08	extracellular matrix	abnormal muscle and tendons	[182]	NA	Y
95	Gata5	1.16	1.41E-06	Transcription factor	abnormal reproduction	[183]	NA	N
96	Gxylt2	1.06	1.60E-05	novel	unknown		NA	
97	Tbx2	1.87	6.98E-07	transcription factor	polydactyly	[184]	Y	Y
98	Parp16	1.07	1.13E-08	novel	unknown		NA	
99	Kti12	1.05	4.91E-06	novel	unknown		NA	
100	Echdc1	1.21	1.29E-04	novel	unknown		NA	
101	Fto	1.02	8.09E-06	metabolism	obese	[185]	NA	N
102	Kctd10	1.07	1.62E-07	potassium channel	unknown		NA	
103	Cygb	1.12	4.51E-05	novel	unknown		NA	
104	Git1	1.34	0.000656	phosphorylation	respiratory	[186]	NA	N
105	Adprhl2	1.02	4.62E-05	novel	unknown		NA	
106	Ppm1g	1.40	0.000824	catalytic enzyme	lethal by weaning		NA	N
107	Rap1gds1	1.22	0.000112	novel	unknown		NA	
108	Fam102a	1.15	4.03E-07	novel	unknown		NA	
109	Rbpms	1.17	2.19E-05	RNA binding	unknown		NA	
110	Ptges2	1.38	2.12E-06	metabolism	normal	[187]	NA	N
111	Farp1	1.06	1.63E-06	novel	unknown		NA	
112	Tulp2	1.10	2.80E-05	novel	unknown		NA	
113	Cdc42ep1	1.00	0.000306	novel	unknown		NA	
114	Sox18	1.17	1.82E-05	Transcription factor	perinatal lethal by E14.5; skin/coat/nails defect	[188]	Y	
115	Xab2	1.78	0.000199	DNA repair	perinatal lethal by E3.5	[189]	NA	EL
116	Lad1	1.67	2.06E-07	novel	unknown		NA	
117	Slc35a4	1.26	5.37E-07	novel	unknown		NA	
118	Mpzl3	1.23	1.40E-06	tyrosine kinase	abnormal muscle and bones	[190]	NA	Y
119	Lrrc16a	1.08	8.53E-07	novel	unknown		NA	
120	Tns1	1.12	1.40E-05	actin binding	kidney/cardiovascular defects	[191]	NA	N
121	Plk1	1.03	8.20E-05	cell cycle/DNA repair	perinatal lethal by E3.5	[192]	NA	EL
122	Mvd	1.06	0.000229	novel	unknown		NA	
123	Abcf2	1.13	3.92E-06	ATP binding	unknown		NA	
124	Jag2	1.51	9.09E-09	growth factor	craniofacial/skeletal defects	[193]	Y	Y
125	Rrn3	1.04	3.39E-06	cell cycle/DNA repair	perinatal lethal by E9.5	[194]	NA	EL
126	Ddx18	1.08	5.50E-05	ATP binding	unknown		NA	
127	Fdx1	1.00	2.36E-08	ion transport	unknown		NA	
128	Edar	1.28	9.31E-09	Transcription factor	skin, coat, nail, skeletal defects	[195]	Y	Y
129	Cdc42ep4	1.00	3.93E-05	novel	unknown		NA	
130	Adamts1	1.01	2.64E-07	extracellular matrix	adipose tissue defect	[196]	Y	N
131	Mkl1	1.28	4.45E-05	actin binding	cardiovascular defects; perinatal lethal	[197]	NA	
132	Dpep1	1.02	0.000228	enzyme regulation	normal	[198]	NA	N
133	Dmd	1.43	1.93E-11	actin binding	muscle defects	[199]	NA	Y
134	Spry1	1.66	1.76E-07	growth factor	renal defects	[200]	Y	N
135	Crispld1	1.02	2.89E-09	novel	unknown		NA	
136	Trpm1	1.32	9.02E-11	ion transport	eye defects	[201]	NA	N
137	Ush1c	1.23	1.33E-05	catalytic enzyme	hearing/vision defects	[202]	NA	N
138	Poll	1.03	0.00056	catalytic enzyme	craniofacial/skeletal defects	[203]	NA	Y
139	BB303372	1.11	0.000552	novel	unknown		NA	
140	Chst8	1.16	0.000168	metabolism	reproductive/metabolic defects	[204]	NA	N
141	Abhd8	1.31	3.15E-05	metabolism	unknown		NA	
142	Bcl7b	1.00	3.23E-05	unknown	unknown		NA	
143	B630019K06Rik	1.17	1.58E-05	unknown	unknown		NA	
144	Tmem178	1.36	1.70E-11	novel	unknown		NA	
145	Pqlc1	1.04	8.56E-12	novel	unknown		NA	
146	Selenbp1	1.44	1.74E-05	novel	unknown		NA	
147	Wbscr16	1.31	2.20E-05	novel	unknown		NA	
148	Gal	1.82	9.91E-06	metabolism	neurological/reproductive defects	[205]	NA	N

149	Tmem101	1.18	1.01E-05	novel	unknown		NA	
150	Cth	1.17	2.28E-08	catalytic enzyme	muscle/cardiovascular defects	[206]	NA	Y
151	Stbd1	1.02	7.77E-08	novel	unknown		NA	
152	Wnt7a	1.94	1.29E-11	wnt signaling	limb defects	[207]	Y	Y
153	Blvra	1.08	1.62E-11	novel	unknown		NA	
154	Jmjd5	1.29	2.03E-08	novel	unknown		NA	
155	Pth1r	1.08	3.12E-05	receptor	skeletal/limb defects	[208]	Y	Y
156	Olfm1	1.01	1.74E-07	ion transport	nervous system and reproductive defects	[209]	Y	N
157	Ctsk	1.79	3.03E-12	hydrolysis	skeletal/limb defects	[210]	NA	Y
158	Dgat2	1.17	1.74E-06	membrane	skin, coat, nail, skeletal defects	[211]	NA	Y
159	Fam89a	1.56	5.38E-09	novel	unknown		NA	
160	Hoxa4	1.29	0.000506	Transcription factor	skeletal/limb defects	[212]	Y	Y
161	Hoxa5	1.07	9.56E-05	Transcription factor	skeletal/limb defects	[213]	Y	Y
162	Unc5b	1.21	1.60E-06	apoptosis	Perinatal lethal by E10	[214]	NA	EL
163	Cldn7	1.22	4.66E-05	cell junction	renal defects	[215]	NA	N
164	Parvb	1.92	5.46E-08	cell junction	normal	[216]	NA	N
165	Tmbim6	1.05	0.000145	membrane protein	metabolism/nervous system defects	[217]	NA	N
166	Zdhhc7*	1.04	3.02E-05	Transcription factor	unknown		Y	
167	Src	1.52	0.000772	ATP binding	abnormal limbs	[218]	NA	Y
168	Bach2	1.36	3.30E-13	Transcription factor	B-cell defect	[219]	Y	N
169	Lmo1*	1.48	1.32E-05	ion binding	normal	[220]	Y	N
170	Asns	1.14	7.02E-15	catalytic enzyme	unknown		NA	
171	Per1	1.30	8.20E-06	transcription factor	osteoblast defect	[221]	Y	Y
172	Mast3	1.01	3.39E-05	novel	unknown		NA	
173	Dusp9	1.13	1.76E-05	phosphatase	Perinatal lethal by E11.5	[222]	NA	EL
174	Cpz	2.17	3.38E-08	novel	unknown		NA	
175	Dlx2	1.01	0.000186	Transcription factor	craniofacial/skeletal defects	[223]	Y	Y
176	C030046I01Rik	1.55	0.000233	novel	unknown		NA	
177	Ppm1m*	1.02	1.17E-06	phosphotase	unknown		Y	
178	Hoxb5	2.14	2.25E-05	transcription factor	skeletal defects	[224]	Y	Y
179	Hoxb6	1.81	9.00E-06	Transcription factor	skeletal defects	[224]	Y	Y
180	Snap91	1.92	2.55E-12	novel	unknown		NA	
181	Crmp1	1.56	1.38E-08	metabolism	nervous system defects, no reported limb phenotype	[225]	NA	N
182	Zfp775	1.11	2.13E-05	Transcription factor	unknown		NA	
183	Alx1	1.57	2.19E-05	Transcription factor	skeletal/limb defects	[226]	NA	Y
184	Rspo2	1.89	1.13E-08	extracellular matrix	skeletal/limb defects	[227]	NA	Y

		E10.5 FL						
No.	Gene Symbol	logFC	Adj. p-val	Putative Function	KO phenotype	Ref	In situ	MSK Function
1	Wdr33	1.02	2.60E-12	novel	unknown		NA	
2	Mup10	1.39	0.000272	novel	unknown		NA	
3	Lsg1	1.1	7.33E-10	novel	unknown		NA	
4	4930534B04Rik	1.13	2.46E-18	novel	unknown		NA	
5	Trappc3	1.17	5.82E-13	novel	unknown		NA	
6	Gpx2	1.79	6.42E-11	metabolism	no phenotype	[228]	NA	N
7	Grk5	1.58	1.04E-16	kinase	metabolic/neural/respiratory/muscle defects	[229]	NA	N
8	Nln	1.06	2.70E-14	novel	unknown		NA	
9	Sh3d19	1.3	6.23E-07	novel	unknown		NA	
10	Capns1	1.1	3.27E-06	Ca-binding	perinatal lethal by E11.5; cardiovascular	[230]	NA	
11	Nudcd3	1.27	6.86E-11	novel	unknown		NA	
12	Ppp2r5b	1.05	9.55E-08	novel	unknown		NA	
13	Arl6ip5	1.02	4.07E-09	novel	unknown		NA	
14	Dpyd	1.17	3.82E-09	novel	unknown		NA	

15	Zik1*	1.18	7.74E-11	novel	unknown		Y	
16	Vps39	1.18	2.15E-13	novel	unknown		NA	
17	Copg2	1.04	1.10E-07	novel	unknown		NA	
18	Galnt7	1.09	1.00E-09	novel	unknown		NA	
19	AI464131	1.13	1.43E-07	novel	unknown		NA	
20	Luzp1	1.11	1.32E-08	novel	neural tube defects	[231]	NA	N
21	Dock10	1.65	1.73E-11	novel	unknown		NA	
22	Arhgap28	1.37	1.83E-11	novel	unknown		NA	
23	Atf7ip2	1.01	1.44E-09	transcription factor	unknown		NA	
24	Ccng2	1.01	2.37E-10	novel	unknown		NA	
25	Mtus1	1.53	7.05E-10	novel	unknown		NA	
26	Hhat	1.1	5.85E-09	transferase activity	limb defects	[232]	NA	Y
27	BC017612*	1.42	1.21E-09	novel	unknown		NA	
28	Hax1	1.03	1.87E-11	signaling molecule	nervous/immune system defects	[233]	NA	N
29	Lrp12	1.11	9.67E-13	novel	unknown		NA	
30	Mov10	1.08	8.90E-13	novel	unknown		NA	
31	2410024N18Rik	1.25	6.58E-10	novel	unknown		NA	
32	Maneal	1.01	0.000206	novel	unknown		NA	
33	Gpcpd1	1.03	1.58E-10	novel	unknown		NA	
34	Pbx1	1.41	1.19E-06	transcription factor	cardiovascular/limb defects	[234]	Y	Y
35	Ryr1	1.05	0.000128	ion transport	abnormal muscle and skeleton	[235]	NA	Y
36	Rabl5	1.16	1.42E-12	novel	unknown		NA	
37	Nmnat1	1.17	2.10E-09	novel	unknown		NA	
38	Lrrc57	1.05	1.70E-11	novel	unknown		NA	
39	Gabarapl2	1.15	1.26E-17	novel	unknown		NA	
40	A030001D20Rik	1.11	9.54E-09	novel	unknown		NA	
41	Clasp1	1.24	7.34E-09	novel	unknown		NA	
42	Ulk4	1.26	2.75E-12	novel	unknown		NA	
43	Bcl7c	1.24	4.80E-08	novel	unknown		NA	
44	Ogdh	1.03	6.14E-07	novel	unknown		NA	
45	Naa40	1.13	2.84E-08	novel	unknown		NA	
46	Tmem177	1.04	3.18E-11	novel	unknown		NA	
47	Tm9sf2	1.1	2.02E-14	novel	unknown		NA	
48	Vav2	1.23	1.21E-10	apoptosis	immune/vision defects	[236]	NA	N
49	Fam46a	1.47	8.87E-06	novel	unknown		NA	
50	Sap130	1.17	6.25E-15	novel	unknown		NA	
51	Peli1	1.07	8.39E-11	b-cell factor	immune system defect	[237]	NA	N
52	Ash2l*	1.15	1.64E-13	chromatin factor	perinatal lethal by E8.5	[238]	Y	EL
53	Slit3	1.2	1.67E-09	signaling molecule	growth/muscle/liver/lung defects	[239]	NA	N
54	5830417I10Rik	1.13	3.00E-07	novel	unknown		NA	
55	Gm9897	1.05	2.97E-09	novel	unknown		NA	
56	Neu1	1.11	6.09E-12	hydrolase	muscle/skeletal defects	[240]	NA	Y
57	Elmo2	1.1	6.59E-09	novel	unknown		NA	
58	Hsd17b12	1.18	3.98E-17	metabolism	perinatal lethal by E8.5	[241]	NA	EL
59	Mesdc1	1.04	1.03E-10	novel	unknown		NA	
60	Kpna3	1.03	3.35E-11	novel	unknown		NA	
61	Gorasp2	1.11	1.38E-08	novel	unknown		NA	
62	Hsf2	1.02	6.00E-05	transcription factor	reproductive/nervous/cardiovascular defects	[242]	NA	N
63	Pafah1b2	1.1	1.49E-13	metabolism	reproductive system defects	[243]	NA	N
64	Fchsd2	1.02	1.17E-12	novel	unknown		NA	
65	E2f4	1.05	6.26E-05	transcription factor	immune/growth size defects	[244]	NA	N
66	Irx3*	1.04	2.94E-05	transcription factor	unknown		Y	
67	Myo10	1.29	1.38E-13	myosin	unknown		NA	
68	Cd40	1.39	1.81E-12	antigen	immune defect	[245]	NA	N
69	Vrk2	1.04	1.45E-09	novel	unknown		NA	
70	Rqcd1	1.14	3.89E-09	novel	unknown		NA	

71	Lpar1	1.25	7.52E-10	receptor	cardiovascular/nervous system defects	[246]	NA	N
72	Pde4dip	1.13	1.00E-09	novel	unknown		NA	
73	Kif3b	1.12	8.95E-15	kinesin	growth/nervous/cardiovascular system defects	[247]	NA	N
74	Sae1	1.01	1.37E-10	novel	unknown		NA	
75	3010026O09Rik	1.02	5.88E-08	novel	unknown		NA	
76	Scube1	1.27	7.54E-08	signaling molecule	craniofacial/nervous system defects	[248]	NA	N
77	Cars	1.13	2.30E-12	novel	unknown		NA	
78	Hrc	1.01	1.78E-07	Ca-binding	cardiovascular/muscle defects	[249]	NA	Y
79	Slc25a30	1.3	2.76E-10	novel	unknown		NA	
80	Tes*	1.01	2.67E-08	novel	digestive/tumorigenesis	[250]	Y	N
81	Zfp763	1.2	2.41E-12	transcription factor	unknown		NA	
82	Zfyve26	1	1.78E-09	novel	unknown		NA	
83	Cbr2	2.78	3.62E-19	novel	unknown		NA	
84	Glrb	1.06	3.20E-06	receptor	skeletal defects	[251]	NA	Y
85	Msl2	1.26	4.60E-12	novel	unknown		NA	
86	Zbtb45	1.23	0.000712	novel	unknown		NA	
87	Fancd2	1.01	1.85E-10	DNA repair	growth size/tumorigenesis	[252]	NA	N
88	Phka1	1.07	4.34E-11	kinase/metabolism	metabolic defect	[253]	NA	N
89	Arhgap24	1.13	2.48E-13	novel	unknown		NA	
90	Rdh12	1.01	1.92E-10	metabolism	vision/nervous system defects	[254]	NA	N
91	Ergic2	1.01	1.94E-09	novel	unknown		NA	
92	Gm13032	1.08	1.23E-06	novel	unknown		NA	
93	Crhbp	1.09	0.000125	metabolism	behavior/growth size defects	[255]	NA	N
94	Cd8b1	1.16	2.58E-06	antigen	immune/hematopoietic defect	[256]	NA	N
95	Fam129c	1.37	2.04E-13	novel	unknown		NA	
96	Asb9	1.12	9.10E-07	novel	unknown		NA	
97	Ube2a	1.11	7.66E-18	DNA repair	infertility	[257]	NA	N
98	Ttpa	1.01	3.88E-08	novel	cardiovascular	[258]	NA	N
99	Nek3	1.02	1.41E-11	novel	unknown		NA	
100	Kdm4b	1.09	6.86E-05	novel	unknown		NA	
101	Mfsd9	1.01	1.72E-06	novel	unknown		NA	
102	Lcp1	1.05	6.13E-11	cytoskeleton	immune system defect	[259]	Y	N
103	2810019C22Rik	1.01	1.46E-05	novel	unknown		NA	
104	1110032A04Rik	1.93	2.78E-12	novel	unknown		NA	
105	Npc1	1.09	3.01E-14	metabolism	nervous/immune system defects	[260]	NA	N
106	Bre	1.03	7.69E-10	novel	unknown		NA	
107	Padi3	1.65	1.10E-08	novel	unknown		NA	
108	Banp	1.26	1.00E-06	novel	unknown		NA	
109	Gm9918	1.05	4.54E-08	novel	unknown		NA	
110	Lbh	1.08	9.52E-09	novel	unknown		NA	
111	Zfat	1.11	3.77E-10	transcription factor	perinatal lethal by E9	[261]	NA	EL
112	Ccnt1	1.37	3.76E-16	novel	unknown		NA	
113	Tmem2	1.02	8.64E-07	membrane	unknown		NA	
114	Zic2	1.60	2.42E-07	transcription factor	limb/skeletal defect	[262]	Y	Y
115	Exosc2	1.01	9.21E-12	novel	unknown		NA	
116	Atf2	1.14	1.55E-09	transcription factor	limb/skeletal defect	[263]	Y	Y
117	Slitrk6	1.33	1.90E-07	novel	nervous/hearing defects	[264]	NA	N
118	Samd14	1.20	2.69E-08	novel	unknown		NA	
119	Gfm2	1.32	1.50E-15	novel	unknown		NA	
120	Pcdhb3	1.04	6.70E-06	novel	unknown		NA	
121	Myst4*	1.07	5.68E-12	metabolism	craniofacial/nervous system defects	[265]	Y	N
122	Zfp418	1.07	1.04E-07	transcription factor	unknown		NA	
123	Junb	1.10	7.84E-06	oncogene	perinatal lethal by E8.5/osteopenia	[266]	NA	Y
124	Cdkn2aip	1.04	8.25E-09	novel	unknown		NA	
125	Senp2	1.19	6.84E-13	metabolism	perinatal lethal by E13.5/cardiovascular	[267]	NA	EL
126	Gimap4	1.12	3.05E-12	GTPase	immune system defect	[268]	NA	N

127	Eif5b	1.01	5.19E-09	translation factor	unknown		NA	
128	Cast	1.01	1.09E-13	enzymatic activity	neuronal defect	[269]	NA	N
129	Nckap5l	1.13	1.94E-05	novel	unknown		NA	
130	Ifi204	1.07	5.19E-04	novel	unknown		NA	
131	Phkg2	1.01	1.68E-07	novel	unknown		NA	
132	Galnt2	1.05	3.07E-07	novel	unknown		NA	
133	Stau1	1.12	2.70E-18	RNA binding	nervous system defect	[270]	NA	N
134	Srp72	1.19	1.07E-11	novel	unknown		NA	
135	Sacm1l	1.08	5.76E-11	metabolism	unknown		NA	
136	4930455C21Rik	1.41	2.39E-10	novel	unknown		NA	
137	Rgl3	1.16	1.35E-06	novel	unknown		NA	
138	Bcl11b*	1.37	1.33E-11	novel	skin/coat/nails/immune system defect	[271]	Y	N
139	Pcyt1b	1.19	4.11E-06	enzymatic activity	tumorigenesis/endocrine defect	[272]	NA	N
140	Stat6*	1.38	9.90E-11	transcription factor	immune system/skin/coat/nail defect	[273]	Y	N
141	Adam10	1.06	4.76E-12	metalloproteinase	nervous/craniofacial/cardiovascular defects	[274]	NA	N
142	Map3k11	1.08	1.50E-05	kinase	skin/coat/nails defect	[275]	NA	N
143	Tmem120a	1.17	1.03E-07	novel	unknown		NA	
144	Shb	1.10	3.65E-08	apoptosis	cardiovascular/limb defects	[276]	NA	Y
145	Dlg4	1.15	1.46E-09	novel	behavior/neurological/growth size defects	[277]	NA	N
146	Senp1	1.06	7.82E-09	enzymatic activity	cardiovascular/immune system defects	[278]	NA	N
147	Cul3	1.08	1.11E-18	enzymatic activity	perinatal lethal by E7.5	[279]	NA	EL
148	Pik3r3	1.41	3.60E-14	novel	unknown		NA	
149	Mrps18a	1.12	2.60E-12	novel	unknown		NA	
150	Serpine1	1.02	9.04E-07	extracellular matrix	cardiovascular/renal/metabolic/tumorigenesis	[280]	NA	N
151	Mras	1.01	1.53E-06	GTPase	normal	[281]	NA	N
152	Becn1	1.18	5.22E-16	autophagy	homozygous lethal; heterozygous tumorigenesis/immune defect	[282]	NA	EL
153	Agpat1	1.19	1.34E-14	novel	unknown		NA	
154	Tyro3	1.10	5.37E-09	kinase	skin/coat/nail/joint inflamation	[283]	NA	Y
155	Ncaph2	1.02	1.78E-06	novel	immune system defect	[284]	NA	N
156	2310067B10Rik	1.03	5.01E-04	novel	unknown		NA	
157	Piga	1.18	2.93E-08	biosynthesis	craniofacial/cartilage/skin/coat/nail defects	[285]	NA	Y
158	Gng2	1.06	2.58E-12	novel	unknown		NA	
159	Mxi1	1.20	8.30E-18	transcription factor	tumorigenesis/endocrine defect	[286]	Y	N
160	Zfp68*	1.08	6.34E-13	transcription factor	unknown		Y	
161	Acadl	1.05	7.67E-13	metabolism	cardiovascular/hepatic/urinary defects	[287]	NA	N
162	2610002D18Rik	1.13	4.14E-11	novel	unknown		NA	
163	Hoxd4	1.72	1.43E-08	transcription factor	craniofacial/skeletal defects	[288]	Y	Y
164	Obfc2b	1.27	8.73E-05	DNA repair	unknown		NA	
165	Ptrf	1.13	9.96E-07	metabolism	metabolism/behavior/growth size defects	[289]	Y	N
166	2410131K14Rik	1.12	1.38E-06	novel	unknown		NA	
167	Wdr5b	1.00	1.75E-09	novel	unknown		NA	
168	Cul5	1.07	3.12E-07	novel	unknown		NA	
169	Itpkc	1.05	1.61E-10	metabolism	normal	[290]	NA	N
170	Stard3nl	1.08	8.04E-08	novel	unknown		NA	
171	Bmp1	1.20	2.25E-11	growth factor	craniofacial/skeletal defects	[291]	NA	Y
172	Zbtb5	1.14	6.71E-05	transcription factor	unknown		Y	
173	Peg3*	1.02	1.20E-11	apoptosis	growth size/neurological	[292]	Y	N
174	Fis1	1.22	1.77E-06	apoptosis	unknown		NA	
175	Wipi2	1.05	2.02E-09	novel	unknown		NA	
176	1110020G09Rik	1.11	6.86E-13	novel	unknown		NA	
177	Txndc16	1.02	5.96E-10	novel	unknown		NA	
178	Sost	1.68	8.97E-12	Wnt antagonist	high bone mass	[293]	Y	Y
179	Dkk1	1.13	8.81E-09	Wnt antagonist	unknown	[294]	Y	Y
180	Fblim1	1.01	1.05E-10	cell adhesion	unknown		Y	
181	Arid3a*	1.55	1.74E-11	transcription factor	unknown		Y	

182	Dr1*	1.07	5.04E-08	transcription factor	unknown		Y	
183	Cbln1	1.30	2.99E-08	cell junction	nervous system defect	[295]	NA	N
184	Apoc1	1.12	3.82E-05	apolipoprotein	homeostasis/liver defect	[296]	NA	N
185	Slc29a4	1.24	2.02E-04	novel	unknown		NA	
186	Micall2	1.11	8.29E-05	novel	unknown		NA	
187	Coro1a	1.11	2.80E-07	actin binding	immune system defect	[297]	NA	N
188	Bdh1	1.04	1.17E-12	novel	unknown		NA	
189	Gypa	1.25	2.81E-05	cytoskeleton	hematopoietic defect	[298]	NA	N
190	Epb4.9	1.09	2.91E-08	cytoskeleton	hematopoietic defect	[299]	NA	N

		E11.5 FL						
No.	Gene Symbol	logFC	Adj. p-val	Putative Function	KO phenotype	Ref	In situ	MSK Function
1	Sorbs3	1.35	1.06E-16	cell adhesion	normal	[300]	Y	N
2	Ssu72	1.24	4.33E-14	novel	unknown		NA	
3	Zfp90*	1.17	9.37E-15	transcription factor	unknown		Y	
4	Ppia	1.04	3.57E-16	novel	inflammation/immune system defects	[301]	NA	N
5	Rplp2	1.09	4.40E-13	novel	unknown		NA	
6	2610036D13Rik	1.02	4.08E-08	novel	unknown		NA	
7	Nek6	1.43	1.07E-14	novel	unknown		NA	
8	Neil1	1.04	5.90E-08	DNA repair	inflammation/tumorigenesis/metabolism	[302]	NA	N
9	Vash2	1.01	5.28E-11	growth factor	cardiovascular defects	[303]		N
10	Nup188	1.10	1.77E-05	novel	unknown		NA	
11	Stt3a	1.05	3.27E-15	novel	unknown		NA	
12	Rbm22	1.01	2.50E-10	novel	unknown		NA	
13	Sgk1	1.11	6.50E-09	apoptosis	renal/urinary system defect	[304]	NA	N
14	Lect1	1.62	8.68E-04	extracellular matrix	skeletal defects/HBM	[305]	NA	Y
15	Cstb	1.14	1.82E-14	metabolism	nervous/muscle physiology defects	[306]	NA	
16	Glyr1	1.01	1.13E-15	novel	unknown		NA	
17	Zfp783	1.10	8.33E-08	novel	unknown		NA	
18	Rpl24	1.21	3.80E-22	novel	perinatal lethal by E9.5/limb defects hets	[307]	NA	Y
19	1110036O03Rik	1.40	7.32E-10	novel	unknown		NA	
20	Tk2	1.27	2.91E-07	metabolism	metabolism/cardiovascular/muscle defects	[308]	NA	Y
21	Rfc4	1.01	1.43E-12	novel	unknown		NA	
22	Pir	1.11	1.06E-08	ion binding	embryonic lethal by E9.5		NA	EL
23	Npm1	1.02	5.89E-18	novel	perinatal lethal by E12.5/abnormal liver	[309]	NA	N
24	Gclm	1.32	1.51E-12	metabolism	metabolism defect	[310]	NA	N
25	Mapk7	1.07	6.19E-04	kinase	perinatal lethal by E10.5/skin/nail/coat/muscle defects	[311]	NA	Y
26	Xylt2	1.65	1.25E-10	metabolism	liver/renal/metabolic defects	[312]	NA	N
27	Sesn2	1.03	6.30E-09	cell cycle	normal/increased apoptosis	[313]	NA	N
28	Gas2l1	1.09	2.56E-07	novel	unknown		NA	
29	Cpt1a	1.06	1.75E-08	membrane	perinatal lethal by E10.5	[314]	NA	EL
30	Mrps26	1.03	3.18E-09	novel	unknown		NA	
31	Col9a3	2.35	2.30E-10	extracellular matrix	unknown		NA	
32	Sat1	1.12	2.07E-12	metabolism	abnormal liver and energy metabolism	[315]	NA	N
33	2400001E08Rik	1.04	3.52E-05	novel	unknown		NA	
34	BC037039	1.03	9.57E-08	novel	unknown		NA	
35	Rps19	1.19	6.20E-20	novel	abnormal skin pigmentation		NA	Y
36	Itfg2	1.37	1.03E-04	novel	unknown		NA	
37	Tsen34	1.19	1.53E-09	novel	unknown		NA	
38	Ddt	1.02	1.41E-11	novel	unknown		NA	
39	H2afy	1.00	5.89E-06	chromatin	normal	[316]	NA	N
40	Tpcn1	1.11	2.84E-05	Ca binding	unknown		NA	
41	Efha1	1.26	1.34E-17	novel	unknown		NA	

42	Plcd3	1.01	7.94E-09	novel	unknown		NA	
43	Fnip2	1.06	4.13E-06	novel	unknown		NA	
44	Tcf19*	1.14	6.00E-11	transcription factor	unknown		Y	
45	Mrpl13	1.01	4.03E-16	novel	unknown		NA	
46	Smarcd3*	1.21	3.68E-07	chromatin	unknown		Y	
47	Morf4l1	1.01	1.92E-18	chromatin	cardiovascular/respiratory/skin/coat/nails/met abolic defects	[317]	NA	N
48	Sf3b4	1.30	2.03E-04	novel	unknown		NA	
49	Rps17	1.52	5.07E-21	novel	unknown		NA	
50	Riok2	1.13	8.15E-10	novel	unknown		NA	
51	Fadd	1.24	1.76E-11	novel	perinatal lethal by E11.5 /cardiovascular/muscle defects	[318]	NA	Y
52	Dullard	1.17	8.41E-04	novel	unknown		NA	
53	Lepre1	1.34	7.60E-10	novel	skeletal/limb/muscle defects	[319]	NA	Y
54	Scara3	1.56	1.26E-12	novel	unknown		NA	
55	Lrrc4b	1.26	4.73E-09	novel	unknown		NA	
56	Gprc5c	1.03	3.09E-10	novel	unknown		NA	
57	Vps29	1.09	1.70E-14	novel	unknown		NA	
58	Ppp2r1a	1.55	1.95E-04	novel	unknown		NA	
59	Nudt22	1.14	8.76E-11	novel	unknown		NA	
60	Nop2	1.16	3.85E-08	novel	unknown		NA	
61	Elof1	1.11	3.22E-06	novel	unknown		NA	
62	Plod1	1.30	3.95E-06	novel	cardiovascular/muscle/skin/coat/nail defects	[320]	NA	Y
63	Bai2	1.71	2.62E-07	novel	normal		NA	N
64	Cyb5	1.07	7.90E-15	electron transport	homeostasis	[321]	NA	N
65	Fbxo17	1.50	9.94E-16	novel	unknown		NA	
66	Rsrc2	1.11	1.59E-15	novel	unknown		NA	
67	Hook2	1.11	3.48E-05	novel	unknown		NA	
68	6030426L16Rik	1.36	6.22E-07	novel	unknown		NA	
69	Refbp2	1.05	9.48E-07	novel	unknown		NA	
70	Zfp13	1.01	3.04E-08	transcription factor	unknown		NA	
71	Rnf167*	1.22	6.87E-07	novel	unknown		Y	
72	Rpl10a	1.02	1.68E-14	novel	unknown		NA	
73	Ap2a1	1.12	1.91E-07	novel	unknown		NA	
74	Capn1	1.08	2.14E-09	Ca binding	metabolism/hematopoietic defect	[322]	NA	N
75	Plvap	1.09	8.63E-05	novel	unknown		NA	
76	2700060E02Rik	1.02	6.66E-15	novel	unknown		NA	
77	Egfl6	1.56	1.28E-05	cell adhesion	unknown		Y	
78	Foxp4*	1.06	6.23E-05	novel	perinatal lethal by E12.5 cardiovascular/digestive/respiratory defects	[323]	Y	N
79	Rpl29	1.33	7.43E-18	ribosomal	skeletal defects	[324]	NA	Y
80	E430018J23Rik	1.14	1.81E-15	novel	unknown		NA	
81	Pias3*	1.05	1.15E-06	transcription factor	unknown		Y	
82	Gatc	1.06	7.06E-11	novel	unknown		NA	
83	Mea1	1.10	2.92E-08	novel	unknown		NA	
84	Dazap1	1.46	5.95E-06	novel	unknown		NA	
85	Myo1c	1.02	6.02E-08	actin binding	hearing/nervous system defects	[325]	NA	N
86	Polr2d	1.05	8.56E-18	novel	unknown		NA	
87	Mrpl54	1.02	2.30E-06	novel	unknown		NA	
88	Tkt	1.08	1.94E-08	metabolism	perinatal lethal before morula/hets are smaller	[326]	NA	EL
89	Cpped1	1.11	8.84E-12	novel	unknown		NA	
90	Txndc9	1.07	4.67E-14	novel	unknown		NA	
91	Atxn7l3	1.06	3.31E-05	novel	unknown		NA	
92	Clcf1	1.40	4.10E-11	novel	unknown		NA	
93	Iscu	1.04	3.20E-11	novel	unknown		NA	
94	Nsmce1	1.40	2.02E-13	novel	unknown		NA	

95	9430038I01Rik	1.12	6.10E-12	novel	unknown		NA	
96	Hps4	1.07	5.10E-07	novel	abnormal pigmentation/renal/ vision/cardiovascular defects	[327]	NA	N
97	Hdhd3	1.07	3.33E-10	novel	unknown		NA	
98	Sec31a	1.20	1.60E-05	novel	unknown		NA	
99	Ctnnb1	1.11	1.93E-14	cell adhesion	skeletal/limb defects		NA	Y
100	Rsl24d1	1.28	5.66E-09	novel	unknown		NA	
101	Ptov1	1.21	1.69E-04	novel	unknown		NA	
102	Spata6	1.30	6.40E-17	novel	unknown		NA	
103	Gm7325	1.81	1.79E-05	novel	unknown		NA	
104	Pabpc1	1.13	9.72E-16	novel	unknown		NA	
105	Cyth1	1.32	3.77E-13	novel	unknown		NA	
106	Chmp6	1.14	4.72E-11	novel	unknown		NA	
107	Tesk1	1.08	8.88E-04	novel	unknown		NA	
108	Mmab	1.08	2.91E-12	novel	unknown		NA	
109	Rps12	1.39	1.81E-20	novel	unknown		NA	
110	S100a13	1.39	1.38E-13	novel	unknown		NA	
111	Prkab1	1.31	9.30E-13	metabolism	nervous/vision/growth defects	[328]	NA	N
112	Ttc17	1.24	9.65E-12	novel	unknown		NA	
113	Rpl7	1.25	2.40E-18	novel	unknown		NA	
114	Glce	1.02	5.98E-11	metabolism	polydactyly/craniofacial/skeletal defects	[329]	NA	Y
115	Sap30l	1.33	6.38E-11	novel	unknown		NA	
116	Mrpl41	1.13	2.65E-12	novel	unknown		NA	
117	Rfk	1.13	1.07E-15	metabolism	perinatal lethal by E7.5	[330]	NA	EL
118	Hsp90b1	1.06	3.59E-11	novel	perinatal lethal by E9.5	[331]	NA	EL
119	Rhobtb1	1.16	2.30E-13	novel	unknown		NA	
120	Rplp0	1.18	1.63E-13	novel	unknown		NA	
121	Setd1b	1.39	1.71E-04	novel	unknown		NA	
122	Dbp	1.19	1.10E-11	transcription factor	behaviour defects	[332]	NA	N
123	Sfrp2	1.65	1.61E-09	growth factor	limb/skeletal defects	[333]	NA	Y
124	Gm166	1.02	8.87E-09	novel	unknown		NA	
125	2310008H09Rik	1.20	1.85E-13	novel	unknown		NA	
126	Hdac11	1.05	2.28E-04	novel	unknown		NA	
127	Med17	1.05	6.65E-18	novel	unknown		NA	
128	Fgfbp1	1.28	2.39E-04	novel	unknown		NA	
129	St8sia6	1.02	1.58E-07	novel	unknown		NA	
130	H2-D1	1.05	3.03E-06	novel	immune defect	[334]	NA	N
131	Gtpbp3	1.11	1.44E-10	novel	unknown		NA	
132	Rpl36al	1.11	1.48E-17	novel	unknown		NA	
133	Mettl1	1.38	4.62E-08	novel	unknown		NA	
134	Pdcd5	1.15	4.92E-18	novel	unknown		NA	
135	Mterfd2	1.03	3.64E-12	novel	unknown		NA	
136	Trappc9	1.06	3.71E-07	novel	unknown		NA	
137	H1f0	1.39	2.89E-14	chromatin	normal	[335]	NA	N
138	Lrrc61	1.08	1.18E-05	novel	unknown		NA	
139	Plec	1.18	1.80E-04	cytoskeleton	cardiovascular/skin/coat/nail/muscle defect	[336]	NA	Y
140	Pgrmc1	1.13	5.84E-18	novel	unknown		NA	
141	Caps2	1.01	1.33E-05	novel	unknown		NA	
142	Urm1	1.10	7.07E-08	novel	unknown		NA	
143	Rpl22	1.28	2.41E-19	ribosomal	hematopoietic/immune defect	[337]	NA	N
144	Gm5914	1.21	9.99E-10	novel	unknown		NA	
145	H3f3a	1.04	2.66E-14	chromatin	behavior/growth/reproductive defects	[338]	NA	N
146	Cnn2	1.03	2.63E-06	actin binding	hematopoietic/immune defect	[339]	NA	N
147	Bend5	1.02	1.65E-13	novel	unknown		NA	
148	Rps4y2	1.10	1.15E-07	novel	unknown		NA	
149	Rab11fip2	1.14	6.46E-12	novel	unknown		NA	

150	Hic1	1.97	1.08E-13	transcription factor	craniofacial/limb/skeletal defects	[340]	Y	Y
151	Slc2a10	1.25	2.64E-11	transport	cardiovascular/immune/renal/respiratory defects	[341]	NA	N
152	Rps25	1.21	8.75E-20	novel	unknown		NA	
153	Adrm1	1.04	2.27E-05	novel	unknown		NA	
154	Comtd1	1.03	2.47E-09	novel	unknown		NA	
155	Myog	2.36	5.42E-04	transcription factor	muscle/skeletal defects	[342]	Y	Y
156	Tab2	1.01	2.36E-15	novel	perinatal lethal by E12.5 cardiovascular/skin/coat/nail/liver defects	[343]	NA	EL
157	BC002163	1.13	1.06E-14	novel	unknown		NA	
158	Gtf2ird1	1.05	1.23E-10	transcription factor	craniofacial/skin/coat/nails/cardiovascular	[344]	Y	Y
159	Isg20l2	1.14	2.01E-06	novel	unknown		NA	
160	Rpl27a	1.25	7.42E-17	novel	unknown		NA	
161	5430416N02Rik	1.09	1.55E-10	novel	unknown		NA	
162	Eif2b5	1.09	1.09E-10	novel	unknown		NA	
163	Lsm1	1.01	1.65E-14	novel	unknown		NA	
164	Nucb1	1.11	1.69E-05	novel	unknown		NA	
165	Itfg3	1.04	2.48E-07	novel	unknown		NA	
166	Hist1h1e	1.13	6.98E-07	chromatin	limb/skeletal defects	[345]	NA	Y
167	Rrp7a	1.09	1.75E-08	novel	unknown		NA	
168	Arhgap1	1.06	2.62E-07	metabolism	skeletal/muscle defects	[346]	NA	Y
169	Ubb	1.03	1.29E-13	metabolism	cardiovascular/growth size defect	[347]	NA	N
170	Mrpl40	1.07	1.76E-10	novel	unknown		NA	
171	Btd	1.11	4.40E-06	novel	unknown		NA	
172	Taf9	1.07	9.32E-19	novel	unknown		NA	
173	Rpl38	1.40	5.62E-20	novel	unknown		NA	
174	Rfesd	1.18	1.99E-09	novel	unknown		NA	
175	Armcx6	1.32	6.23E-14	novel	unknown		NA	
176	Dnajc19	1.01	1.09E-10	novel	unknown		NA	
177	Tpm1	1.46	1.19E-11	actin binding	perinatal lethal by E11.5	[348]	NA	EL
178	Gabbr1	1.01	4.04E-07	receptor	perinatal lethal	[349]	NA	EL
179	H13	1.04	6.25E-07	novel	unknown		NA	
180	Cttn*	1.06	5.55E-09	novel	perinatal lethal by 2 cell stage	[350]	Y	EL
181	Fxyd5	1.02	2.05E-08	novel	unknown		NA	
182	Tcp11l2	1.47	9.12E-11	novel	unknown		NA	
183	Tspan18	1.37	2.44E-11	novel	unknown		NA	
184	Ndufv2	1.08	6.42E-17	novel	unknown		NA	
185	Phactr2	1.07	2.57E-10	novel	unknown		NA	
186	Gorasp1	1.07	3.81E-08	novel	unknown		NA	
187	Sssca1	1.17	2.85E-11	novel	unknown		NA	
188	Pef1	1.32	1.35E-08	novel	unknown		NA	
189	Rps18	1.49	1.90E-13	ribosomal	normal	[351]	NA	N
190	Rpl11	1.03	2.05E-13	novel	unknown		NA	
191	1810049H13Rik	1.06	8.42E-11	novel	unknown		NA	
192	Hspb6	1.30	3.13E-09	novel	unknown		NA	
193	Eif3d	1.30	1.49E-09	novel	unknown		NA	
194	Prelid1	1.08	1.07E-06	novel	unknown		NA	
195	Ntm	1.11	2.82E-10	novel	unknown		NA	
196	Hmg20b*	1.86	3.33E-05	novel	unknown		Y	
197	Rpl19	1.42	4.07E-19	novel	unknown		NA	
198	Cul9	1.07	5.91E-08	cell cycle	normal	[352]	NA	N
199	Supt7l	1.08	1.95E-09	novel	unknown		NA	
200	Plcb3	1.11	1.41E-08	phospholipase	skin/coat/nail/immune/metabolic defects	[353]	NA	N
201	Mrto4	1.02	1.25E-08	novel	unknown		N/A	
202	C1qa	1.35	2.51E-09	extracellular protein	immune defect	[354]	Y	N
203	Atp5d	1.05	9.14E-07	novel	unknown		NA	

204	Fam195b	1.01	2.61E-09	novel	unknown		NA	
205	Rpl13a	1.07	2.02E-14	novel	unknown		NA	
206	Akap1	1.08	2.16E-09	membrane protein	reduced female fertility	[355]	NA	N
207	Strn4	1.02	9.85E-05	novel	unknown		NA	
208	Chd3	1.04	3.56E-06	chromatin	unknown		NA	
209	Nr2c2ap	1.14	3.69E-05	novel	unknown		NA	
210	Ube2e3	1.05	5.69E-10	novel	unknown		NA	
211	Hn1l	1.05	3.04E-08	novel	unknown		NA	
212	Adamts7	1.16	5.88E-06	novel	unknown		NA	
213	1810009N02Rik	1.01	4.27E-08	novel	unknown		NA	
214	Pdlim2	1.09	2.65E-06	actin cytoskeleton	immune defect	[356]	NA	N
215	Col6a1	1.36	2.17E-05	cell adhesion	muscle defect	[357]	NA	Y
216	2610528E23Rik	1.00	2.47E-10	novel	unknown		NA	
217	Rad51l1	1.02	2.53E-09	DNA repair	Perinatal lethal by E5.5	[358]	NA	EL
218	Fgf9	1.10	1.47E-09	novel	skeletal defects	[359]	Y	Y
219	Nxnl2	1.05	1.04E-08	novel	unknown		NA	
220	Sertad1	1.18	4.61E-12	novel	impaired glucose metabolism	[360]	Y	N
221	Atp2a1	1.31	1.73E-06	ATP binding	muscle defect	[361]	NA	Y
222	Nat15	1.07	8.12E-08	metabolism	unknown		NA	
223	Fasn	1.05	1.43E-06	novel	unknown		NA	
224	Dusp23	1.04	5.66E-07	novel	unknown		NA	
225	Frmpd1	1.03	2.00E-06	novel	unknown		NA	
226	Ncl	1.25	2.20E-14	novel	unknown		NA	
227	Acan	1.52	4.48E-05	cell adhesion	skeletal and limb defects	[362]	NA	Y
228	Rps14	1.08	5.96E-18	novel	unknown		NA	
229	Sirpa	1.02	1.28E-10	actin binding	growth/hematopoietic/immune defect	[363]	NA	N
230	Btg1	1.06	3.91E-11	novel	unknown		NA	
231	Mest	1.04	4.98E-11	novel	perinatal lethal/hets growth retardation	[364]	Y	EL
232	Gstm7	1.29	1.21E-12	novel	unknown		NA	
233	3110056O03Rik	1.20	2.17E-07	novel	unknown		NA	
234	Arhgef17	1.02	8.32E-11	novel	unknown		NA	
235	Pcdhb19	1.13	4.14E-06	cell adhesion	unknown		NA	
236	Tnfrsf22	1.05	1.76E-14	novel	unknown		NA	
237	Ctdsp2*	1.18	2.49E-11	novel	unknown		Y	
238	Mad2l2	1.35	1.29E-06	novel	unknown		NA	
239	Snrpg	1.50	3.04E-18	RNA splicing	unknown		NA	
240	Rpl34	1.04	1.62E-15	ribosomal	unknown		NA	
241	Dgcr14	1.06	2.69E-08	novel	congenital heart disease	[365]	NA	N
242	Fam63a	1.01	7.21E-09	novel	unknown		NA	
243	Dcaf8	1.14	4.86E-08	novel	unknown		NA	
244	Cwc25	1.29	3.13E-06	novel	unknown		NA	
245	Gngt2	1.11	5.69E-06	novel	unknown		NA	
246	D630008O14Rik	1.41	3.66E-10	novel	unknown		NA	
247	Ubc	1.22	5.74E-17	novel	perinatal lethal by E12.5	[366]	NA	EL
248	1300018J18Rik	1.08	6.50E-10	novel	unknown		NA	
249	2010002M09Rik	1.18	4.51E-07	novel	unknown		NA	
250	Exosc4	1.05	1.28E-11	novel	unknown		NA	
251	Calhm2	1.29	9.13E-10	novel	unknown		NA	
252	Rpl13	1.60	1.26E-20	novel	unknown		NA	
253	Palmd	1.11	7.01E-05	novel	unknown		NA	
254	Col8a1	1.58	7.02E-09	extracellular protein	vision/eye defects	[367]	NA	N
255	Slc25a39	1.30	3.45E-07	membrane protein	unknown		NA	
256	Lipa	1.05	1.08E-07	hydrolase activity	various metabolic defects	[368]	NA	N
257	Nenf	1.23	2.34E-09	novel	unknown		NA	
258	Ttc5	1.02	1.79E-12	novel	unknown		NA	
259	Cbln4	1.42	2.84E-08	novel	unknown		NA	

260	Ephb6	1.20	1.70E-08	signaling molecule	skeletal defects	[369]	NA	Y
261	Mrps23	1.10	7.89E-15	novel	unknown		NA	
262	Rps5	1.02	6.43E-16	ribosomal	unknown		NA	
263	Zbtb8a	1.32	1.33E-08	transcription factor	unknown		NA	
264	Thap3*	1.31	2.07E-11	novel	unknown		Y	
265	Zfp637	1.03	2.85E-10	transcription factor	unknown		NA	
266	Zcchc3	1.06	1.74E-06	novel	unknown		NA	
267	Man2a2	1.16	9.55E-10	metabolism	infertility/metabolic defects	[370]	NA	N
268	Col6a2	1.65	3.60E-10	extracellular protein	unknown		NA	
269	Anapc2	1.09	2.34E-07	cell cycle	skeletal defects	[371]	NA	Y
270	Gapdh	1.15	1.72E-07	metabolism	perinatal lethal shortly after implantation	[372]	NA	EL
271	Cenpb	1.07	3.59E-05	chromatin	reproductive/metabolic defects	[373]	NA	N
272	Rpl7a	1.04	8.56E-18	ribosomal	unknown		NA	
273	AI894139	1.03	7.85E-11	novel	unknown		NA	
274	5730559C18Rik	1.20	4.56E-09	novel	unknown		NA	
275	Fntb	1.38	7.63E-04	metabolism	perinatal lethal by E11.5	[374]	NA	EL
276	Avpi1	1.38	1.71E-14	novel	unknown		NA	
277	Tulp1	1.06	2.71E-09	cell junction	vision/eye defects	[375]	NA	N
278	Lfng	1.04	4.19E-06	metabolism	limb/skeletal defects		Y	Y
279	Rassf7	1.04	2.02E-08	novel	unknown		NA	
280	Mrpl17	1.03	1.54E-11	mitochondrial	unknown		NA	
281	Sarnp	1.19	3.87E-17	novel	unknown		NA	
282	Sult5a1	1.62	6.97E-14	metabolism	unknown		NA	
283	Rab40c	1.14	3.61E-09	novel	unknown		NA	
284	Il11ra1	1.22	8.24E-10	cytokine	perinatal lethal by E12	[376]	NA	EL
285	Camk1g	1.21	8.65E-11	ATP binding	nervous system defect	[377]	NA	N
286	Rpl23	1.40	6.20E-21	ribosomal	unknown		NA	
287	AI414330	1.22	1.27E-06	novel	unknown		NA	
288	Wiz	1.07	5.89E-04	transcription factor	unknown		NA	
289	Psma7	1.06	2.15E-18	novel	unknown		NA	
290	Srebf1	1.25	2.37E-08	transcription factor	lipid metabolism/liver defects	[378]	Y	N
291	Mccc2	1.11	8.91E-13	ATP binding	unknown		NA	
292	Arhgap17	1.25	7.67E-08	novel	unknown		NA	
293	Mapk13	1.03	6.65E-06	kinase activity	metabolic defects	[379]	NA	N
294	Nfkbib	1.14	2.82E-05	transcription factor	skeletal defects/arthritis	[380]	NA	Y
295	Rps27l	1.26	3.59E-16	DNA repair	unknown		NA	
296	Mrps35	1.09	5.33E-14	novel	unknown		NA	
297	Gadd45gip1	1.12	2.18E-07	cell cycle	perinatal lethal by E6.5	[381]	NA	EL
298	Tpst2	1.06	2.13E-08	novel	metabolic defects	[382]	NA	N
299	Ckap4	1.25	1.44E-06	novel	unknown		NA	
300	E4f1*	1.12	1.96E-11	transcription factor	perinatal lethal by E5.5	[383]	Y	EL
301	Ypel3	1.32	7.39E-08	novel	unknown		NA	
302	Tcfe3	1.12	5.38E-05	transcription factor	osteopetrosis in compound phenotype	[384]	Y	Y
303	Smo	1.20	4.35E-10	growth factor	limb/skeletal defects	[385]	NA	Y
304	Pabpc4	1.07	2.34E-05	novel	unknown		NA	
305	0610007C21Rik	1.04	1.34E-10	novel	unknown		NA	
306	Zhx2	1.24	2.84E-08	transcription factor	unknown		NA	
307	Lactb2	1.11	2.41E-12	metabolism	metabolic defects	[386]	NA	N
308	Rpl23a	1.20	7.62E-19	novel	unknown		NA	
309	Mtx1	1.03	1.17E-10	novel	perinatal lethal	[387]	NA	EL
310	Epb4.1	1.12	1.74E-06	actin binding	hematopoietic/immune defect	[388]	NA	N
311	Iqcb1	1.13	6.40E-17	novel	unknown		NA	
312	Kcnmb4	1.69	9.95E-16	Ca binding	nervous system defect	[389]	NA	N
313	Smarce1*	1.03	2.39E-10	chromatin	unknown		Y	
314	Mettl5	1.10	3.05E-10	methylation	unknown		NA	
315	Ltbp4	1.56	1.20E-10	growth factor	muscle/cardiac defects	[390]	NA	Y

316	Ddx24	1.27	1.59E-12	novel	unknown		NA	
317	Gipc1	1.41	3.29E-05	novel	cardiovascular	[391]	NA	N
318	Mxd4	1.03	6.55E-10	transcription factor	unknown		NA	
319	Mre11a	1.07	4.17E-14	novel	perinatal lethal by E10.5	[392]	NA	EL
320	Mapk8ip3	1.19	6.12E-09	novel	nervous system defect	[393]	NA	N
321	Rgs19	1.13	1.17E-09	novel	unknown		NA	
322	Ly6c1	1.76	4.57E-11	novel	unknown		NA	
323	4933400F03Rik	1.35	2.00E-11	novel	unknown		NA	
324	0610009O20Rik	1.19	5.85E-10	novel	unknown		NA	
325	Vegfb	1.30	0.000411	growth factor	cardiovascular	[394]	NA	N
326	Nol12	1.09	3.28E-11	novel	unknown		NA	
327	Rpl7l1	1.01	1.13E-12	novel	unknown		NA	
328	1500001M20Rik	1.02	1.91E-11	novel	unknown		NA	
329	Psmb1	1.06	1.98E-14	novel	unknown		NA	
330	Wdr59	1.10	2.41E-11	novel	unknown		NA	
331	Fance	1.01	2.78E-05	novel	unknown		NA	
332	S1pr1	1.03	4.74E-06	novel	abnormal limb and muscle development	[395]	NA	Y
333	Cad	1.06	0.000312	novel	unknown		NA	
334	Rac1	1.02	2.68E-08	novel	embryonic lethal by E9.5	[396]	NA	EL
335	S100a10	1.03	5.63E-10	Ca binding	metabolism defect	[397]	Y	N
336	Dgcr6	1.06	2.95E-09	novel	unknown		NA	
337	Amacr	1.10	1.37E-11	metabolism	metabolic defects	[398]	NA	N
338	Dapk2	1.24	7.49E-11	novel	unknown		NA	
339	Ndufa11	1.07	1.95E-12	novel	unknown		NA	
340	Ncf4	1.57	1.78E-08	novel	embryonic lethal by E10	[399]	NA	
341	Inadl	1.23	1.57E-14	novel	unknown		NA	
342	Osbpl5	1.16	3.35E-05	novel	unknown		NA	
343	2310014H01Rik	1.03	0.0004	novel	unknown		NA	
344	Slc43a1	1.03	1.00E-09	novel	unknown		NA	
345	Rps21	1.03	4.90E-16	ribosomal	unknown		NA	
346	Crls1	1.07	3.29E-15	novel	unknown		NA	
347	Rpl35a	1.12	1.33E-17	ribosomal	unknown		NA	
348	Eif4ebp1	1.20	4.55E-10	novel	metabolic defects	[400]	NA	N
349	Kri1	1.16	1.16E-09	novel	unknown		NA	
350	Sirt5	1.05	4.46E-13	novel	no defects	[401]	NA	N
351	Tradd	1.11	2.13E-05	novel	immune/hematopoietic defects	[402]	NA	N
352	Cnbp	1.07	4.91E-17	transcription factor	perinatal lethal by E10/hets have skeletal/craniofacial defects	[403]	Y	Y
353	Fam162a	1.08	3.88E-16	novel	unknown		NA	
354	Fbxw5	1.11	0.000112	novel	unknown		NA	
355	Casp3	1.11	9.83E-11	apoptosis	skeletal/craniofacial defects	[404]	NA	Y
356	Ciapin1	1.12	3.86E-10	novel	perinatal lethal by E12.5	[405]	NA	EL
358	Zranb1	1.03	1.85E-13	transcription factor	unknown		NA	
359	Ltbp3	1.02	1.30E-09	growth factor	limb/skeletal defects	[406]	NA	Y
360	Ccdc104	1.31	8.28E-13	novel	unknown		NA	
361	Man2c1	1.09	2.95E-10	novel	unknown		NA	
362	Sfrs13b	1.80	1.08E-08	novel	unknown		NA	
363	Trp53i13	1.21	3.10E-06	novel	unknown		NA	
364	Btbd2	1.32	0.0002	novel	unknown		NA	
365	Timm17a	1.04	1.99E-17	novel	unknown		NA	
366	Prune	1.05	0.00057	novel	unknown		NA	
367	Efemp2	1.25	1.18E-09	extracellular protein	cardiovascular/muscle defects	[407]	NA	Y
368	Bbs4	1.11	4.42E-12	tubulin binding	vision/eye/neurological/metabolic defects	[408]	NA	N
369	2310016C08Rik	1.15	2.81E-13	novel	unknown		NA	
370	Lypd6b	1.69	2.38E-14	novel	unknown		NA	
371	Gm5577	1.53	0.000412	novel	unknown		NA	

372	Jdp2	1.39	4.07E-06	novel	limb/digit/tail defects	[409]	NA	Y
373	1110004F10Rik	1.01	1.91E-16	novel	unknown		NA	
374	Gng13	1.01	1.79E-05	novel	unknown		NA	
375	Ptma	1.17	8.81E-15	novel	unknown		NA	
376	Rpl21	1.07	4.42E-18	novel	unknown		NA	
377	Nhp2l1	1.03	8.69E-19	novel	unknown		NA	
378	Tle3	1.18	1.06E-10	novel	unknown		NA	
379	Hexim2	1.29	1.13E-13	novel	unknown		NA	
380	Nbl1	1.46	1.78E-11	extracellular protein	skeletal defects	[410]	Y	Y
381	Tmem138	1.13	0.000291	novel	unknown		NA	
382	Txndc17	1.02	4.47E-16	novel	unknown		NA	
383	Rcvrn	1.01	3.55E-07	Ca binding	vision/eye defects	[411]	NA	N
384	Snx21	1.22	5.68E-12	novel	unknown		NA	
385	Lamb3	1.17	9.99E-08	extracellular protein	digestive system defect	[412]	NA	N
386	Efcab2	1.09	2.83E-09	novel	unknown		NA	
387	Mif	1.07	3.60E-09	growth factor	immune defect	[413]	NA	N
388	1110021L09Rik	1.03	3.94E-05	novel	unknown		NA	
389	Rps29	1.19	8.68E-20	novel	unknown		NA	
390	Wdr6	1.15	1.89E-06	novel	unknown		NA	
391	Psmd9	1.02	7.55E-09	novel	unknown		NA	
392	Rpl35	1.23	2.89E-17	novel	unknown		NA	
393	Cyb5r3	1.13	1.72E-06	ATP binding	unknown		NA	
394	Gm10244	1.28	1.29E-12	novel	unknown		NA	
395	Ube2o	1.02	0.00044	novel	unknown		NA	
396	Slc48a1	1.00	8.96E-10	membrane protein	no obvious defects		NA	N
397	Ehd3	1.03	3.32E-11	novel	unknown		NA	
398	C1qb	1.19	5.43E-06	novel	unknown		NA	
399	Creg1	1.14	9.49E-12	novel	unknown		NA	
400	Gm16517	1.03	1.59E-06	novel	male sterility/neurological defects	[414]	NA	N
401	Rad23a	1.14	0.000222	DNA repair	no obvious defects	[415]	NA	N
402	C1qc	1.24	2.89E-06	novel	unknown		NA	
403	Gpx1	1.03	8.83E-11	metabolism	digestive/metabolic defects	[416]	NA	N
404	Hsd3b6	1.33	3.80E-05	novel	unknown		NA	
405	Cited4	1.02	0.000827	transcription factor	unknown		Y	
406	Ahsp	1.13	0.000372	novel	hematopoietic/immune defect	[417]	NA	N

		E12.5 FL						
**No.**	**Gene Symbol**	**logFC**	**Adj. p-val**	**Putative Function**	**KO phenotype**	**Ref**	**In situ**	**MSK Function**
1	Myeov2	1.07	8.52E-13	novel	unknown		NA	
2	Il10rb	1.11	5.59E-07	cytokine	immune/digestive/hematopoietic defects	[418]	NA	N
3	A530047J11Rik	1.16	1.42E-07	novel	unknown		NA	
4	A2m	1.18	2.01E-06	novel	unknown		NA	
5	Ghr	1.28	3.59E-11	receptor	limb/digits/skeletal defects	[419]	NA	Y
6	Cav3	1.02	0.000115	metabolic	muscle defects	[420]	NA	Y
7	L1td1	1.15	3.06E-06	novel	unknown		NA	
8	Musk	1.36	1.10E-10	ATP binding	muscle/skeletal defects	[421]	NA	Y
9	Ift80	1.30	1.28E-19	novel	unknown		NA	
10	Zfp57*	1.02	3.18E-08	transcription factor	perinatal lethal by E14.5	[422]	Y	EL
11	Pde6d	1.22	2.57E-11	novel	unknown		NA	
12	Rpf1	1.05	1.17E-09	novel	unknown		NA	
13	Aimp2	1.04	1.04E-13	novel	death at birth/unknown causes	[423]	NA	N
14	Mettl4	1.43	1.03E-13	chromatin	unknown		NA	
15	C130050O18Rik*	1.03	1.07E-05	novel	unknown		NA	
16	Pde7b	1.11	9.06E-08	novel	unknown		NA	

17	Pdhx	1.28	5.58E-15	novel	unknown		NA	
18	Pcdh17	1.11	4.06E-06	novel	unknown		NA	
19	Ociad1	1.28	1.03E-12	novel	unknown		NA	
20	Ankle2	1.02	2.35E-12	novel	unknown		NA	
21	Nat2	1.03	5.13E-08	metabolic	vision/eye/metabolism defects	[424]	NA	N
22	Gnpda2	1.11	3.58E-09	novel	unknown		NA	
23	Kbtbd3*	1.40	9.27E-08	novel	unknown		Y	
24	Mrpl53	1.07	3.89E-12	novel	unknown		NA	
25	Slc14a1	1.03	5.66E-05	membrane	metabolic/immune/hematopoietic defects	[425]	NA	N
26	Orc1l	1.17	8.02E-13	novel	unknown		NA	
27	Mfap3l	1.09	1.04E-08	novel	unknown		NA	
28	Tmem208	1.06	2.08E-13	novel	unknown		NA	
29	Ccdc56	1.03	1.24E-11	novel	unknown		NA	
30	Srd5a2	1.40	5.43E-06	metabolic	metabolic/reproductive defects	[426]	NA	N
31	Cdc14b	1.02	1.02E-11	novel	unknown		NA	
32	Zfp354b*	1.04	4.71E-06	transcription factor	unknown		Y	
33	Filip1l	1.33	3.52E-12	novel	unknown		NA	
34	Ttc32	1.46	1.06E-11	novel	unknown		NA	
35	Ankrd29	1.59	1.79E-11	novel	unknown		NA	
36	Fam82a1	1.20	4.35E-09	novel	unknown		NA	
37	Sycp3	1.25	1.25E-11	cell cycle	reproductive/metabolic defects	[427]	NA	N
38	Rabgef1	1.12	1.29E-11	ATP binding	immune/hematopoietic defects	[428]	NA	N
39	Hpgds	1.02	2.14E-08	metabolic	immune/hematopoietic defects	[429]	NA	N
40	Prr5l	1.14	1.29E-11	novel	unknown		NA	
41	Nup35	1.09	7.56E-13	novel	unknown		NA	
42	Herc1	1.17	5.07E-07	novel	behavior/nervous system defects	[430]	NA	N
43	BC006779	1.05	5.43E-06	novel	unknown		NA	
44	Prox1	1.14	1.14E-07	transcription factor	perinatal lethal by E14.5	[431]	Y	EL
45	Ube2v2	1.05	1.48E-10	novel	unknown		NA	
46	Hps5	1.02	9.17E-09	novel	pigmentation defects		NA	N
47	Trpc1	1.46	5.19E-11	ca binding	digestive/metabolic defects	[432]	NA	N
48	Itga8	1.23	2.47E-09	cell adhesion	renal/nervous/hearing/behavior defects	[433]	NA	N
49	3110035C09Rik	1.14	3.14E-07	novel	unknown		NA	
50	Edn3	1.02	2.57E-12	extracellular	digestive/nervous/pigmentation defects	[434]	NA	N
51	Mobkl1b	1.06	3.10E-17	novel	unknown		NA	
52	Mgat3	1.29	1.26E-07	glycosylation	liver/tumorigenesis	[435]	NA	N
53	Btaf1	1.02	3.63E-14	novel	unknown		NA	
54	Dguok	1.01	1.30E-09	novel	unknown		NA	
55	Spin4	1.13	0.00022	novel	unknown		NA	
56	4933421E11Rik	1.07	9.71E-05	novel	unknown		NA	
57	Ttk	1.13	5.15E-15	novel	unknown		NA	
58	Dach1	1.03	9.84E-08	novel	behavior/nervous system defects	[436]	Y	N
59	Slc35b4	1.14	1.54E-15	novel	unknown		NA	
60	Txndc12	1.09	1.50E-13	novel	unknown		NA	
61	Nudt12	1.07	3.93E-06	novel	unknown		NA	
62	Agpat6	1.01	3.82E-10	metabolic	reproductive/metabolic defects	[437]	NA	N
63	Alkbh7	1.13	6.61E-12	novel	unknown		NA	
64	Tnnt3	1.42	1.10E-06	ca binding	unknown		NA	
65	Sstr1	1.03	5.10E-07	membrane	vision/eye/nervous system defects	[438]	NA	N
66	Ap3m1	1.09	1.28E-14	novel	unknown		NA	
67	H2-L	1.11	8.09E-08	novel	unknown		NA	
68	Hspa1b	1.67	3.42E-09	heat shock protein	cardiovascular/metabolic defects	[439]	NA	N
69	Tbc1d23	1.00	8.68E-08	novel	unknown		NA	
70	Pde3a	1.11	2.69E-11	novel	female infertility	[440]	NA	N
71	Pigh	1.07	5.34E-14	novel	unknown		NA	
72	Zfp110*	1.20	3.78E-16	transcription factor	perinatal lethal by E12.5	[441]	NA	EL

73	Exph5	1.24	4.83E-10	novel	unknown		NA	
74	Pik3c2a	1.13	9.18E-14	novel	unknown		NA	
75	Rev3l	1.18	1.08E-10	DNA repair	immune/hematopoietic defects	[442]	NA	EL
76	Slc25a31	1.28	1.04E-05	membrane	infertility/metabolic defects	[443]	NA	N
77	Trip4	1.19	8.83E-19	receptor	unknown		NA	
78	Slc26a7	1.50	2.63E-08	membrane	unknown	[444]	NA	N
79	BC011248	1.03	1.71E-10	novel	unknown		NA	
80	1810029B16Rik	1.19	2.38E-13	novel	unknown		NA	
81	Ccdc18	1.02	1.68E-09	novel	unknown		NA	
82	Srbd1	1.13	4.69E-10	novel	unknown		NA	
83	Ncapd3	1.02	4.50E-12	novel	unknown		NA	
84	Gnb1l	1.03	1.32E-09	novel	unknown		NA	
85	Epyc	1.63	7.80E-05	novel	unknown		NA	
86	Tm2d3	1.19	1.39E-12	novel	unknown		NA	
87	Agl	1.20	2.04E-07	novel	unknown		NA	
88	Pdlim5	1.15	3.03E-08	cytoskeleton	unknown		NA	
89	Fbxo30	1.03	3.63E-11	novel	unknown		NA	
90	C79407	1.20	5.66E-11	novel	unknown		NA	
91	Plk4	1.09	2.99E-14	ATP binding	perinatal lethal by E7.5	[445]	NA	EL
92	Soat1	1.19	4.45E-14	metabolic	metabolic/immune/hematopoietic defects	[446]	NA	N
93	Med7	1.09	2.15E-13	novel	unknown		NA	
94	Ddb2	1.15	8.49E-12	DNA repair	tumorigenesis/growth side defect	[447]	NA	N
95	Rab28	1.02	2.85E-15	novel	unknown		NA	
96	Trim29	1.02	1.11E-06	novel	unknown		NA	
97	1700012D01Rik	1.20	2.68E-07	novel	unknown		NA	
98	Jak1	1.12	6.14E-14	kinase	neonatal lethal/ immune/ hematopoieitic/ neurological defect	[448]	NA	N
99	Cnksr3	1.17	3.89E-11	novel	unknown		NA	
100	Ammecr1	1.10	0.000373	novel	unknown		NA	
101	Pno1	1.03	6.49E-15	novel	unknown		NA	
102	Kdm1b	1.13	9.88E-09	chromatin	female infertility	[449]	NA	N
103	2610036L11Rik	1.06	1.74E-10	novel	unknown		NA	
104	Manf	1.10	3.70E-14	novel	unknown		NA	
105	Bod1l	1.01	3.18E-10	novel	unknown		NA	
106	Txnip	1.13	9.05E-13	novel	cardiovascular/metabolic defects	[450]	NA	N
107	Osbpl3	1.36	1.42E-13	novel	unknown		NA	
108	Rapsn	1.09	6.01E-06	novel	nervous system/muscle defects	[451]	NA	Y
109	Snx13	1.30	1.39E-10	signal transduction	perinatal lethal by E12.5	[452]	NA	EL
110	Fam13a	1.01	3.99E-11	novel	unknown		NA	
111	Ddit3*	1.12	7.75E-09	novel	immune/metabolic/neurological defects	[453]	Y	N
112	Wapal	1.04	8.50E-13	novel	unknown		NA	
113	Car14	1.03	2.87E-07	membrane	no obvious defects	[454]	NA	N
114	Med8	1.05	5.64E-15	transcription factor	unknown		NA	
115	Zfp324	1.31	3.74E-10	novel	unknown		NA	
116	Dusp14	1.27	1.92E-12	novel	unknown		NA	
117	Hs3st3a1	1.05	7.16E-10	novel	unknown		NA	
118	9230114K14Rik	1.09	1.52E-07	novel	unknown		NA	
119	2700097O09Rik	1.24	3.68E-15	novel	unknown		NA	
120	Zmym1	1.09	7.64E-13	novel	unknown		NA	
121	Zfp715	1.08	3.85E-13	transcription factor	unknown		NA	
122	Calml3	1.01	0.000141	novel	unknown		NA	
123	Klf10	1.05	3.48E-10	transcription factor	skeletal/limb defects	[455]	NA	Y
124	Fgf7	1.53	1.96E-11	growth factor	kidney/immune/nervous system defects	[456]	NA	N
125	4933411K20Rik	1.01	3.75E-13	novel	unknown		NA	
126	Rarres2	1.14	3.82E-11	novel	unknown		NA	
127	Ap3b1	1.04	1.14E-15	novel	abnormal pigmentation		NA	N

128	Rsu1	1.04	1.98E-12	novel	unknown		NA	
129	Lrrc51	1.04	2.38E-06	novel	unknown		NA	
130	Bivm	1.07	7.83E-13	novel	unknown		NA	
131	Ntn4	1.08	9.95E-08	novel	unknown		NA	
132	Cops2*	1.02	6.08E-15	novel	perinatal lethal by E3	[457]	NA	EL
133	Pygo1	1.06	2.51E-09	novel	vision/eye/nervous system defects	[458]	NA	N
134	Wars2	1.09	2.45E-13	novel	unknown		NA	
135	Tmem11	1.00	2.80E-13	novel	unknown		NA	
136	Mcts2	1.05	4.13E-11	novel	unknown		NA	
137	Col11a2	1.07	0.000114	cell adhesion	craniofacial/limb/skeletal defects	[459]	NA	Y
138	Hiatl1	1.17	1.63E-13	novel	unknown		NA	
139	Comp	1.21	0.000188	cell adhesion	skeletal/limb defects	[460]	NA	Y
140	2610307P16Rik	1.24	5.43E-08	novel	unknown		NA	
141	Hspb11	1.16	8.33E-10	novel	unknown		NA	
142	Ccdc127	1.25	9.79E-15	novel	unknown		NA	
143	Myot	1.23	3.42E-05	actin binding	no obvious defects	[461]	NA	N
144	D330028D13Rik	1.01	1.27E-09	novel	unknown		NA	
145	Dcun1d1*	1.07	6.45E-12	novel	unknown		Y	
146	Gm106	1.30	6.18E-07	novel	unknown		NA	
147	1810063B05Rik	1.19	8.14E-14	novel	unknown		NA	
148	Bbs5	1.36	5.79E-14	novel	unknown		NA	
149	Hapln1	1.31	3.16E-07	extracellular	craniofacial/limb/skeletal defects	[462]	NA	Y
150	Ifit2	1.02	3.23E-09	novel	unknown		NA	
151	Ptgfr	1.08	7.06E-09	membrane	reproductive/metabolic defects	[463]	NA	N
152	Pcdhb11	1.09	8.18E-05	cell adhesion	unknown		NA	
152	Ccdc58	1.38	2.09E-17	novel	unknown		NA	
153	Setdb1	1.11	1.57E-11	chromatin	perinatal lethal by implantation	[464]	NA	EL
154	Atl2	1.26	6.39E-14	novel	unknown		NA	
155	Nebl	1.37	1.28E-08	novel	unknown		NA	
156	Sytl2	1.02	5.96E-06	novel	digestive system defects	[465]	NA	N
157	Scrg1	1.79	2.51E-08	novel	unknown		NA	
158	Apobec2	1.44	0.000524	metabolic	growth retardation and decreased BMD		NA	Y

		E13.5 FL						
**No.**	**Gene Symbol**	**logFC**	**Adj. p-val**	**Putative Function**	**KO phenotype**	**Ref**	**In situ**	**MSK Function**
1	2310067E19Rik	2.59	1.74E-10	novel	unknown		NA	
2	Mdm4	1.13	7.80E-17	novel	perinatal lethal between E9-12	[466]	NA	EL
3	BB046190	1.18	4.16E-05	novel	unknown		NA	
4	Lphn2	1.04	8.49E-18	receptor	unknown		NA	
5	Rbm25	1.60	5.16E-14	novel	unknown		NA	
6	Smc6	1.01	2.38E-13	novel	unknown		NA	
7	A630033H20Rik	1.26	1.61E-09	novel	unknown		NA	
8	Zfp760	1.24	1.43E-13	transcription factor	unknown		NA	
9	D130084N16Rik	1.96	1.93E-10	novel	unknown		NA	
10	Rtl1	2.36	4.58E-17	novel	growth retardation	[467]	NA	N
11	6430537I21Rik	1.08	0.000642	novel	unknown		NA	
12	AI428301	1.32	1.77E-05	novel	unknown		NA	
13	Ccnl2	1.50	2.56E-19	transcription factor	unknown		NA	
14	6330417A16Rik	1.42	1.86E-08	novel	unknown		NA	
15	4432414F05Rik	1.60	2.41E-09	novel	unknown		NA	
16	Ppargc1b	1.14	5.41E-08	novel	metabolic/cardiovascular/liver defects	[468]	NA	N
17	Srrm2	1.43	2.76E-07	novel	unknown		NA	
18	AW549877	1.57	2.36E-18	novel	unknown		NA	
19	Boc	1.15	7.91E-11	novel	nervous system defects	[469]	Y	N

20	4930422I07Rik	1.08	1.13E-09	novel	unknown		NA	
21	D5Ertd505e	1.07	3.69E-07	novel	unknown		NA	
22	Dmtf1*	1.02	1.19E-12	transcription factor	immune/hematopoietic/renal/nervous system	[470]	Y	N
23	2610042L04Rik	1.39	7.03E-16	novel	unknown		NA	
24	Atp11a	1.01	1.05E-09	novel	unknown		NA	
25	Sltm	1.06	1.02E-15	transcription factor	unknown		NA	
26	2610311E24Rik	1.78	8.91E-11	novel	unknown		NA	
27	Abi3bp	1.35	9.29E-09	novel	unknown		NA	
28	Nktr	1.38	2.57E-12	novel	unknown		NA	
29	Luc7l3	1.04	2.83E-17	novel	unknown		NA	
30	C80142	2.10	7.87E-13	novel	unknown		NA	
31	Tpsab1	1.05	3.60E-07	novel	immune/hematopoietic defects	[471]	NA	N
32	4930518I15Rik	1.02	1.37E-05	novel	unknown		NA	
33	BC022960	1.18	3.79E-07	novel	unknown		NA	
34	Tslp	1.23	1.12E-09	novel	immune/hematopoietic defects	[472]	NA	N
35	Scai	1.29	4.37E-09	novel	unknown		NA	
36	Scml2	1.09	1.19E-07	novel	unknown		NA	
37	AI131651	1.40	4.19E-06	novel	unknown		NA	
38	Ptprv	1.21	1.27E-09	receptor	metabolic/growth size defect	[473]	Y	N
39	Irx5*	1.01	1.18E-05	transcription factor	vision/eye/nervous system defects	[474]	Y	N
40	Zcchc7	1.30	6.79E-15	transcription factor	unknown		NA	
41	Clk1	1.30	4.61E-11	novel	unknown		NA	
42	Dse	1.25	1.92E-10	novel	growth/limb/reproductive defects	[475]	NA	Y
43	Fam82b	1.01	4.98E-10	novel	unknown		NA	
44	9430085L16Rik	1.22	3.58E-08	novel	unknown		NA	
45	Ccdc39	2.39	3.52E-14	novel	unknown		NA	
46	Med13l	1.12	1.44E-06	novel	unknown		NA	
47	Zfp826	1.90	1.66E-14	transcription factor	skeletal defectss	[476]	NA	Y
48	C230037E05Rik	1.66	1.02E-08	novel	unknown		NA	
49	6030451C04Rik	2.08	1.33E-05	novel	unknown		NA	
50	1700094D03Rik	1.16	7.99E-14	novel	unknown		NA	
51	4833414E09Rik	1.47	8.12E-07	novel	unknown		NA	
52	Ptcd3	1.05	5.87E-15	novel	unknown		NA	
53	AA415038	1.25	0.000187	novel	unknown		NA	
54	Fubp1	1.41	6.04E-07	novel	unknown		NA	
55	Krit1	1.29	2.52E-10	novel	perinatal lethal by E11	[477]	NA	EL
56	Nfat5%	1.38	6.69E-17	transcription factor	metabolic/renal defects	[478]	Y	N
57	Itih5	1.03	9.06E-06	novel	unknown		NA	
58	Adamts6	1.88	6.16E-13	novel	unknown		NA	
59	Zbed6	2.20	3.55E-08	transcription factor	unknown		NA	
60	Serinc4	1.02	9.44E-06	novel	unknown		NA	
61	Ttc14	2.27	8.86E-18	novel	unknown		NA	
62	C80068	1.05	1.66E-05	novel	unknown		NA	
63	D030002E05Rik	1.51	1.17E-07	novel	unknown		NA	
64	5031426D15Rik	1.55	4.03E-10	novel	unknown		NA	
65	9630030I15Rik	1.72	1.92E-07	novel	unknown		NA	
66	6430590A07Rik	1.83	1.08E-11	novel	unknown		NA	
67	5830407P18Rik	1.75	3.72E-08	novel	unknown		NA	
68	Zmynd11	1.04	5.14E-14	transcription factor	unknown		NA	
69	2410042D21Rik	1.07	5.68E-13	novel	unknown		NA	
70	D630030B22Rik	1.05	3.09E-05	novel	unknown		NA	
71	Gm15241	2.02	8.54E-10	novel	unknown		NA	
72	Mpp5	1.01	6.55E-11	membrane	nervous system defects	[479]	NA	N
73	Zfp280d	1.37	1.27E-13	transcription factor	unknown		NA	
74	Akap9	1.17	8.96E-09	novel	unknown		NA	
75	Cep110	1.06	3.47E-12	novel	unknown		NA	

76	Usp28	1.12	5.57E-14	novel	unknown		NA	
77	6030400A10Rik	1.10	4.32E-11	novel	unknown		NA	
78	Gm7890	1.67	3.69E-07	novel	unknown		NA	
79	Wipf1	1.23	4.69E-16	cytoskeleton	immune/hematopoietic defects	[480]	NA	N
80	Sobp	1.24	2.40E-06	novel	behavior defects	[481]	NA	N
81	Eif4a2	1.12	5.08E-13	transcription factor	unknown		NA	
82	D030041H20Rik	1.25	6.57E-05	novel	unknown		NA	
83	Sfrs18	1.80	2.24E-18	novel	unknown		NA	
84	Scara5	1.24	2.61E-06	receptor	unknown		NA	
85	Gcfc1	1.77	3.41E-14	novel	unknown		NA	
86	Caprin2	1.29	2.37E-13	novel	unknown		NA	
87	Smek2	1.07	4.71E-11	novel	unknown		NA	
88	D5Ertd798e	2.40	1.71E-09	novel	unknown		NA	
89	Fndc3c1	1.49	6.88E-16	novel	unknown		NA	
90	BB211804	1.31	5.11E-06	novel	unknown		NA	
91	2210403K04Rik	1.07	1.63E-06	novel	unknown		NA	
92	AU041975	1.15	2.98E-06	novel	unknown		NA	
93	Fbn1	1.20	5.40E-13	novel	cardiovascular/muscle/skeletal defects	[482]	NA	Y
94	D2Ertd173e	2.80	1.35E-08	novel	unknown		NA	
95	Ankrd2	1.19	0.000211	novel	muscle defects	[483]	NA	Y
96	Hmcn1	1.33	2.34E-07	novel	unknown		NA	
97	Dep1	1.34	1.25E-05	novel	unknown		NA	
98	Hcfc2	1.04	3.28E-11	novel	unknown		NA	
99	2810055G20Rik	1.45	7.12E-08	novel	unknown		NA	
100	5830474E16Rik	2.27	6.07E-10	novel	unknown		NA	
101	2810405F17Rik	1.21	8.42E-08	novel	unknown		NA	
102	9530086O07Rik	1.64	1.85E-06	novel	unknown		NA	
103	Zc3h7a	1.03	5.88E-08	novel	unknown		NA	
104	2310003F16Rik	2.84	6.29E-15	novel	unknown		NA	
105	9930017N22Rik	1.70	6.17E-06	novel	unknown		NA	
106	2700099C18Rik	1.02	2.08E-09	novel	unknown		NA	
107	Prdm2	1.02	1.66E-07	transcription factor	tumorigenesis	[484]	NA	N
108	Rhoj	1.30	6.98E-12	novel	unknown		NA	
109	9430076C15Rik	1.13	3.91E-07	novel	unknown		NA	
110	Rgag4	1.05	2.38E-07	novel	unknown		NA	
111	Lime1	1.00	1.67E-07	novel	no obvious abnormalities	[485]	NA	N
112	Rbm26	1.35	3.58E-11	novel	unknown		NA	
113	Kdm3a	1.04	1.50E-16	chromatin	metabolism/liver/muscle/reproductive	[486]	NA	Y
114	Ccdc52	1.01	1.63E-14	novel	unknown		NA	
115	Rnpc3	1.11	1.47E-06	novel	unknown		NA	
116	E330013P04Rik	1.83	4.53E-09	novel	unknown		NA	
117	AI605517	1.29	6.61E-07	novel	unknown		NA	
118	Eif2ak4	1.04	4.03E-11	transcription factor	behavior/metabolic/liver/muscle/ reproductive defects	[487]	NA	Y
119	Ankrd16	1.84	1.05E-16	novel	unknown		NA	
120	Egln3	1.08	4.99E-12	novel	craniofacial/limb defects	[488]	NA	Y
121	Mtmr1	1.31	1.62E-15	novel	no obvious abnormalities		NA	N
122	Pvrl3	1.44	4.56E-15	novel	vision/eye/reproductive defects	[489]	NA	N
123	9430047G12Rik	1.63	1.95E-09	novel	unknown		NA	
124	2610011E03Rik	1.36	1.52E-05	novel	unknown		NA	
125	Rbm39	2.33	1.97E-22	RNA binding	perinatal lethal	[490]	NA	EL
126	Ccnt2	1.43	2.86E-14	cell division	death by 4 cell stage	[491]	NA	EL
127	D430033H22Rik	2.08	2.43E-10	novel	unknown		NA	
128	Spata1	1.24	1.55E-06	novel	unknown		NA	
129	4932431P20Rik	1.05	0.000294	novel	unknown		NA	
130	D330040H18Rik	2.22	2.36E-12	novel	unknown		NA	

131	Zim1*	1.07	1.01E-09	transcription factor	unknown		Y	
132	Pax7*	1.33	9.74E-07	transcription factor	craniofacial/muscle defects in gain of function mutant	[492]	NA	Y
133	Rbm5	1.33	4.66E-17	novel	unknown		NA	
134	Moxd1	1.13	1.91E-07	novel	unknown		NA	
135	Zfp207*	1.00	3.24E-17	transcription factor	unknown		Y	
136	5430406J06Rik	1.86	1.48E-09	novel	unknown		NA	
137	Mll3*	1.21	1.15E-12	novel	growth/vision/reproductive defects	[493]	Y	N
138	5830415B17Rik	1.10	8.51E-05	novel	unknown		NA	
139	Dzip3	1.23	7.52E-07	novel	unknown		NA	
140	Fbxl3	1.25	1.49E-10	novel	behavior defects	[494]	NA	N
141	Ythdf3	1.32	1.68E-16	novel	unknown		NA	
142	AU018552	1.13	3.82E-08	novel	unknown		NA	
143	9430047L24Rik	1.83	7.40E-06	novel	unknown		NA	
144	Gls	1.24	1.29E-14	metabolic	behavior/respiratory/neurological defects	[495]	NA	N
145	Golga1	1.02	8.17E-15	novel	unknown		NA	
146	Dock4	1.13	4.63E-06	novel	unknown		NA	
147	Chrnb1	1.24	4.04E-06	novel	nervous system defects	[496]	NA	N
148	2810043O03Rik	3.61	2.71E-13	novel	unknown		NA	
149	6430537K16Rik	3.35	6.88E-15	novel	unknown		NA	
150	Gm9159	1.28	1.47E-07	novel	unknown		NA	
151	Gas5	3.61	4.92E-19	novel	unknown		NA	
152	A630026N12Rik	1.07	1.87E-07	novel	unknown		NA	
153	E030016H06Rik	2.48	6.68E-12	novel	unknown		NA	
154	Zfp788	1.25	3.43E-11	transcription factor	unknown		NA	
155	Phf20l1	1.14	2.47E-09	novel	unknown		NA	
156	4833423F13Rik	1.08	2.19E-05	novel	unknown		NA	
157	Guf1	1.22	3.71E-11	novel	unknown		NA	
158	1110006E14Rik	2.84	1.25E-17	novel	unknown		NA	
159	Cox8b	1.24	1.42E-07	novel	unknown		NA	
160	A130004G07Rik	1.03	1.65E-05	novel	unknown		NA	
161	2010111I01Rik	1.08	4.16E-15	novel	unknown		NA	
162	Irx2*	1.20	2.40E-08	transcription factor	no obvious abnormalities	[497]	Y	N
163	Ebf2	1.14	6.61E-08	transcription factor	limb/skeletal defects	[498]	Y	Y
164	B230117O15Rik	1.13	1.49E-05	novel	unknown		NA	
165	Sdpr	1.18	3.73E-06	novel	unknown		NA	
166	Wsb1	1.07	1.01E-05	novel	unknown		NA	
167	B930025B16Rik	2.20	2.34E-12	novel	unknown		NA	
168	Meg3	1.74	6.27E-13	novel	growth retardation	[499]	NA	N
169	Gm2590	1.89	7.11E-06	novel	unknown		NA	
170	Robo2	1.36	2.82E-05	axon guidance	nervous system defects	[500]	Y	N
171	C77673	1.61	1.57E-07	novel	unknown		NA	
172	Jmjd1c	1.04	0.000683	novel	unknown		NA	
173	1700012D14Rik	1.01	1.05E-08	novel	unknown		NA	
174	9930031P18Rik	1.72	3.62E-06	novel	unknown		NA	
175	9630010G10Rik	2.34	1.17E-13	novel	unknown		NA	
176	4732423E21Rik	1.73	0.000107	novel	unknown		NA	
177	C130075A20Rik	1.37	2.99E-05	novel	unknown		NA	
178	Rbbp4	2.12	1.16E-06	novel	unknown		NA	
179	9530029O12Rik	1.62	4.16E-05	novel	unknown		NA	
180	AI451458	1.37	1.63E-07	novel	unknown		NA	
181	C530014P21Rik	1.90	3.97E-09	novel	unknown		NA	
182	6720420G18Rik	1.10	1.13E-09	novel	unknown		NA	
183	Cxcl13	1.71	1.16E-05	novel	immune/hematopoietic defects	[501]	NA	N
