# Supplementary material for: Global Gene Expression Analysis of Murine Limb Development
Source: PLoS One. 2011 Dec 9;6(12):e28358. doi: 10.1371/journal.pone.0028358 (PMC3235105; doi:10.1371/journal.pone.0028358)
Supplement: Material and Methods S1 — In Situ Probe Information. (PDF) [file pone.0028358.s009.pdf]

Material and Methods S1

In Situ Probe Information

Novel Genes without available Image Clones were PCR amplified from mouse genomic DNA.

Gene	Forward Primer	Reverse Primer
BC017612	TCAAAGGGAAGCCAAAGAAA	CAAATCCCATCAAGCACAGA
C130050O18rik	GACTATGACGGCCATGTGTG	ATATGCATCCGAGTCCAGGT
Lmx1-B	CTGCTGTGCAAGGGTGACTA	GCTACTTCCGTAGGGGCTCT
Lix1	CATGAGGAGACTGTGCAGGA	CTGGTGGAGGCTACTGCTTC
C130021I20Rik	ATGAAAGTGCTCGTGGTGTG	CAGGGTGGAGACCAATGAAC
6230427J02Rik	AGGAAGAGGAGGAAGGCTTG	AGTTCATGAGGGCTGCAAAT

Gene	Accession #	Image Clone #
Fbxo41	BC070445	30536738
Tbx5	BC090639	30548234
Hoxc10	BC053405	30059581
Pitx1	BC012696	4192818
Nnat	BC036984	4981517
1200009O22Rik	BC059224	6406165
Phf6	BC057374	6826091
Cap1	BC005472	3590890
Net1	BC004699	3499258
Frg1	BC002027	3487998
Aass	BC005420	3600654
Dusp7	BC010207	3964527
Fibin	BC027250	3603588
