# Supplementary material for: Global Gene Expression Analysis of Murine Limb Development
Source: PLoS One. 2011 Dec 9;6(12):e28358. doi: 10.1371/journal.pone.0028358 (PMC3235105; doi:10.1371/journal.pone.0028358)
Supplement: References S1 — Supplemental references. (PDF) [file pone.0028358.s010.pdf]

References S1

1. Wynick D, Small CJ, Bacon A, Holmes FE, Norman M, et al. (1998) Galanin regulates prolactin release and lactotroph proliferation. Proc Natl Acad Sci U S A 95: 12671-12676.

2. Tzchori I, Day TF, Carolan PJ, Zhao Y, Wassif CA, et al. (2009) LIM homeobox transcription factors integrate signaling events that control three-dimensional limb patterning and growth. Development 136: 1375-1385.

3. Witte F, Dokas J, Neuendorf F, Mundlos S, Stricker S (2009) Comprehensive expression analysis of all Wnt genes and their major secreted antagonists during mouse limb development and cartilage differentiation. Gene Expr Patterns 9: 215-223.

4. Fromental-Ramain C, Warot X, Messadecq N, LeMeur M, Dolle P, et al. (1996) Hoxa-13 and Hoxd-13 play a crucial role in the patterning of the limb autopod. Development 122: 2997-3011.

5. Kmita M, Fraudeau N, Herault Y, Duboule D (2002) Serial deletions and duplications suggest a mechanism for the collinearity of Hoxd genes in limbs. Nature 420: 145-150.

6. Trelles RD, Leon JR, Kawakami Y, Simoes S, Izpisua Belmonte JC (2002) Expression of the chick vascular endothelial growth factor D gene during limb development. Mech Dev 116: 239-242.

7. Galceran J, Farinas I, Depew MJ, Clevers H, Grosschedl R (1999) Wnt3a-/--like phenotype and limb deficiency in Lef1(-/-)Tcf1(-/-) mice. Genes Dev 13: 709-717.

8. Bottcher RT, Wiesner S, Braun A, Wimmer R, Berna A, et al. (2009) Profilin 1 is required for abscission during late cytokinesis of chondrocytes. Embo J 28: 1157-1169.

9. van den Akker E, Fromental-Ramain C, de Graaff W, Le Mouellic H, Brulet P, et al. (2001) Axial skeletal patterning in mice lacking all paralogous group 8 Hox genes. Development 128: 1911-1921.

10. Singh MK, Petry M, Haenig B, Lescher B, Leitges M, et al. (2005) The T-box transcription factor Tbx15 is required for skeletal development. Mech Dev 122: 131-144.

11. Bandyopadhyay A, Tsuji K, Cox K, Harfe BD, Rosen V, et al. (2006) Genetic analysis of the roles of BMP2, BMP4, and BMP7 in limb patterning and skeletogenesis. PLoS Genet 2: e216.

12. Cobb J, Dierich A, Huss-Garcia Y, Duboule D (2006) A mouse model for human short-stature syndromes identifies Shox2 as an upstream regulator of Runx2 during long-bone development. Proc Natl Acad Sci U S A 103: 4511-4515.

13. Houle M, Sylvestre JR, Lohnes D (2003) Retinoic acid regulates a subset of Cdx1 function in vivo. Development 130: 6555-6567.

14. Kinoshita M, Era T, Jakt LM, Nishikawa S (2009) The novel protein kinase Vlk is essential for stromal function of mesenchymal cells. Development 136: 2069-2079.

15. Imuta Y, Nishioka N, Kiyonari H, Sasaki H (2009) Short limbs, cleft palate, and delayed formation of flat proliferative chondrocytes in mice with targeted disruption of a putative protein kinase gene, Pkdcc (AW548124). Dev Dyn 238: 210-222.

16. McIntyre DC, Rakshit S, Yallowitz AR, Loken L, Jeannotte L, et al. (2007) Hox patterning of the vertebrate rib cage. Development 134: 2981-2989.

17. Ishikawa H, Barber GN (2008) STING is an endoplasmic reticulum adaptor that facilitates innate immune signalling. Nature 455: 674-678.

18. ten Berge D, Brouwer A, Korving J, Martin JF, Meijlink F (1998) Prx1 and Prx2 in skeletogenesis: roles in the craniofacial region, inner ear and limbs. Development 125: 3831-3842.

19. Rancourt DE, Tsuzuki T, Capecchi MR (1995) Genetic interaction between hoxb-5 and hoxb-6 is revealed by nonallelic noncomplementation. Genes Dev 9: 108-122.

20. Forger NG, Prevette D, deLapeyriere O, de Bovis B, Wang S, et al. (2003) Cardiotrophin-like cytokine/cytokine-like factor 1 is an essential trophic factor for lumbar and facial motoneurons in vivo. J Neurosci 23: 8854-8858.

21. Carpenter EM, Goddard JM, Davis AP, Nguyen TP, Capecchi MR (1997) Targeted disruption of Hoxd-10 affects mouse hindlimb development. Development 124: 4505-4514.

22. Robanus-Maandag E, Giovannini M, van der Valk M, Niwa-Kawakita M, Abramowski V, et al. (2004) Synergy of Nf2 and p53 mutations in development of malignant tumours of neural crest origin. Oncogene 23: 6541-6547.

23. Swindell EC, Moeller C, Thaller C, Eichele G (2001) Cloning and expression analysis of chicken Lix1, a founding member of a novel gene family. Mech Dev 109: 405-408.

24. Nakaya MA, Habas R, Biris K, Dunty WC, Jr., Kato Y, et al. (2004) Identification and comparative expression analyses of Daam genes in mouse and Xenopus. Gene Expr Patterns 5: 97-105.

25. Murray SA, Oram KF, Gridley T (2007) Multiple functions of Snail family genes during palate development in mice. Development 134: 1789-1797.

26. Anderson WJ, Zhou Q, Alcalde V, Kaneko OF, Blank LJ, et al. (2008) Genetic targeting of the endoderm with claudin-6CreER. Dev Dyn 237: 504-512.

27. Medghalchi SM, Frischmeyer PA, Mendell JT, Kelly AG, Lawler AM, et al. (2001) Rent1, a trans-effector of nonsense-mediated mRNA decay, is essential for mammalian embryonic viability. Hum Mol Genet 10: 99-105.

28. Lewandoski M, Sun X, Martin GR (2000) Fgf8 signalling from the AER is essential for normal limb development. Nat Genet 26: 460-463.

29. Parr BA, McMahon AP (1995) Dorsalizing signal Wnt-7a required for normal polarity of D-V and A-P axes of mouse limb. Nature 374: 350-353.

30. Firulli BA, Krawchuk D, Centonze VE, Vargesson N, Virshup DM, et al. (2005) Altered Twist1 and Hand2 dimerization is associated with Saethre-Chotzen syndrome and limb abnormalities. Nat Genet 37: 373-381.

31. Stephen LJ, Fawkes AL, Verhoeve A, Lemke G, Brown A (2007) A critical role for the EphA3 receptor tyrosine kinase in heart development. Dev Biol 302: 66-79.

32. Caubit X, Core N, Boned A, Kerridge S, Djabali M, et al. (2000) Vertebrate orthologues of the Drosophila region-specific patterning gene teashirt. Mech Dev 91: 445-448.

33. Ferretti E, Villaescusa JC, Di Rosa P, Fernandez-Diaz LC, Longobardi E, et al. (2006) Hypomorphic mutation of the TALE gene Prep1 (pKnox1) causes a major reduction of Pbx and Meis proteins and a pleiotropic embryonic phenotype. Mol Cell Biol 26: 5650-5662.

34. Bell SM, Schreiner CM, Hess KA, Anderson KP, Scott WJ (2003) Asymmetric limb malformations in a new transgene insertional mutant, footless. Mech Dev 120: 597-605.

35. Werling U, Schorle H (2002) Transcription factor gene AP-2 gamma essential for early murine development. Mol Cell Biol 22: 3149-3156.

36. Zirzow S, Ludtke TH, Brons JF, Petry M, Christoffels VM, et al. (2009) Expression and requirement of T-box transcription factors Tbx2 and Tbx3 during secondary palate development in the mouse. Dev Biol 336: 145-155.

37. Wakamiya M, Blackburn MR, Jurecic R, McArthur MJ, Geske RS, et al. (1995) Disruption of the adenosine deaminase gene causes hepatocellular impairment and perinatal lethality in mice. Proc Natl Acad Sci U S A 92: 3673-3677.

38. Cifuentes-Diaz C, Frugier T, Tiziano FD, Lacene E, Roblot N, et al. (2001) Deletion of murine SMN exon 7 directed to skeletal muscle leads to severe muscular dystrophy. J Cell Biol 152: 1107-1114.

39. Conover JC, Erickson JT, Katz DM, Bianchi LM, Poueymirou WT, et al. (1995) Neuronal deficits, not involving motor neurons, in mice lacking BDNF and/or NT4. Nature 375: 235-238.

40. Boulet AM, Capecchi MR (2004) Multiple roles of Hoxa11 and Hoxd11 in the formation of the mammalian forelimb zeugopod. Development 131: 299-309.

41. Boumil RM, Letts VA, Roberts MC, Lenz C, Mahaffey CL, et al. A missense mutation in a highly conserved alternate exon of dynamin-1 causes epilepsy in fitful mice. PLoS Genet 6.

42. Oh J, Takahashi R, Adachi E, Kondo S, Kuratomi S, et al. (2004) Mutations in two matrix metalloproteinase genes, MMP-2 and MT1-MMP, are synthetic lethal in mice. Oncogene 23: 5041-5048.

43. Peters B, Kirfel J, Bussow H, Vidal M, Magin TM (2001) Complete cytolysis and neonatal lethality in keratin 5 knockout mice reveal its fundamental role in skin integrity and in epidermolysis bullosa simplex. Mol Biol Cell 12: 1775-1789.

44. Muller AJ, Baker JF, DuHadaway JB, Ge K, Farmer G, et al. (2003) Targeted disruption of the murine Bin1/Amphiphysin II gene does not disable endocytosis but results in embryonic cardiomyopathy with aberrant myofibril formation. Mol Cell Biol 23: 4295-4306.

45. Davenport TG, Jerome-Majewska LA, Papaioannou VE (2003) Mammary gland, limb and yolk sac defects in mice lacking Tbx3, the gene mutated in human ulnar mammary syndrome. Development 130: 2263-2273.

46. Boskey AL, Gelb BD, Pourmand E, Kudrashov V, Doty SB, et al. (2009) Ablation of cathepsin k activity in the young mouse causes hypermineralization of long bone and growth plates. Calcif Tissue Int 84: 229-239.

47. Yonemasu R, Minami M, Nakatsu Y, Takeuchi M, Kuraoka I, et al. (2005) Disruption of mouse XAB2 gene involved in pre-mRNA splicing, transcription and transcription-coupled DNA repair results in preimplantation lethality. DNA Repair (Amst) 4: 479-491.

48. Beverdam A, Brouwer A, Reijnen M, Korving J, Meijlink F (2001) Severe nasal clefting and abnormal embryonic apoptosis in Alx3/Alx4 double mutant mice. Development 128: 3975-3986.

49. Agarwal P, Wylie JN, Galceran J, Arkhitko O, Li C, et al. (2003) Tbx5 is essential for forelimb bud initiation following patterning of the limb field in the mouse embryo. Development 130: 623-633.

50. Lohnes D, Mark M, Mendelsohn C, Dolle P, Dierich A, et al. (1994) Function of the retinoic acid receptors (RARs) during development (I). Craniofacial and skeletal abnormalities in RAR double mutants. Development 120: 2723-2748.

51. Gawenis LR, Ledoussal C, Judd LM, Prasad V, Alper SL, et al. (2004) Mice with a targeted disruption of the AE2 Cl-/HCO3- exchanger are achlorhydric. J Biol Chem 279: 30531-30539.

52. Bensoussan V, Lallemand Y, Moreau J, Cloment CS, Langa F, et al. (2008) Generation of an Msx2-GFP conditional null allele. Genesis 46: 276-282.

53. Ihrie RA, Reczek E, Horner JS, Khachatrian L, Sage J, et al. (2003) Perp is a mediator of p53-dependent apoptosis in diverse cell types. Curr Biol 13: 1985-1990.

54. Gross MK, Moran-Rivard L, Velasquez T, Nakatsu MN, Jagla K, et al. (2000) Lbx1 is required for muscle precursor migration along a lateral pathway into the limb. Development 127: 413-424.

55. Zhang Z, Verheyden JM, Hassell JA, Sun X (2009) FGF-regulated Etv genes are essential for repressing Shh expression in mouse limb buds. Dev Cell 16: 607-613.

56. Basson MA, Akbulut S, Watson-Johnson J, Simon R, Carroll TJ, et al. (2005) Sprouty1 is a critical regulator of GDNF/RET-mediated kidney induction. Dev Cell 8: 229-239.

57. Lallemand Y, Nicola MA, Ramos C, Bach A, Cloment CS, et al. (2005) Analysis of Msx1; Msx2 double mutants reveals multiple roles for Msx genes in limb development. Development 132: 3003-3014.

58. Mukhopadhyay R, Ho YS, Swiatek PJ, Rosen BP, Bhattacharjee H (2006) Targeted disruption of the mouse Asna1 gene results in embryonic lethality. FEBS Lett 580: 3889-3894.

59. Moon YS, Smas CM, Lee K, Villena JA, Kim KH, et al. (2002) Mice lacking paternally expressed Pref-1/Dlk1 display growth retardation and accelerated adiposity. Mol Cell Biol 22: 5585-5592.

60. Ding H, Wu X, Bostrom H, Kim I, Wong N, et al. (2004) A specific requirement for PDGF-C in palate formation and PDGFR-alpha signaling. Nat Genet 36: 1111-1116.

61. Su X, Paris M, Gi YJ, Tsai KY, Cho MS, et al. (2009) TAp63 prevents premature aging by promoting adult stem cell maintenance. Cell Stem Cell 5: 64-75.

62. Imamoto A, Soriano P (1993) Disruption of the csk gene, encoding a negative regulator of Src family tyrosine kinases, leads to neural tube defects and embryonic lethality in mice. Cell 73: 1117-1124.

63. Witke W, Li W, Kwiatkowski DJ, Southwick FS (2001) Comparisons of CapG and gelsolin-null macrophages: demonstration of a unique role for CapG in receptor-mediated ruffling, phagocytosis, and vesicle rocketing. J Cell Biol 154: 775-784.

64. Ansorge HL, Meng X, Zhang G, Veit G, Sun M, et al. (2009) Type XIV Collagen Regulates Fibrillogenesis: PREMATURE COLLAGEN FIBRIL GROWTH AND TISSUE DYSFUNCTION IN NULL MICE. J Biol Chem 284: 8427-8438.

65. Zhao Q, Behringer RR, de Crombrugghe B (1996) Prenatal folic acid treatment suppresses acrania and meroanencephaly in mice mutant for the Cart1 homeobox gene. Nat Genet 13: 275-283.

66. Johnstone KA, DuBose AJ, Futtner CR, Elmore MD, Brannan CI, et al. (2006) A human imprinting centre demonstrates conserved acquisition but diverged maintenance of imprinting in a mouse model for Angelman syndrome imprinting defects. Hum Mol Genet 15: 393-404.

67. Davis AP, Capecchi MR (1994) Axial homeosis and appendicular skeleton defects in mice with a targeted disruption of hoxd-11. Development 120: 2187-2198.

68. Huntington ND, Puthalakath H, Gunn P, Naik E, Michalak EM, et al. (2007) Interleukin 15-mediated survival of natural killer cells is determined by interactions among Bim, Noxa and Mcl-1. Nat Immunol 8: 856-863.

69. Gao Y, Lan Y, Ovitt CE, Jiang R (2009) Functional equivalence of the zinc finger transcription factors Osr1 and Osr2 in mouse development. Dev Biol 328: 200-209.

70. Nara N, Nakayama Y, Okamoto S, Tamura H, Kiyono M, et al. (2007) Disruption of CXC motif chemokine ligand-14 in mice ameliorates obesity-induced insulin resistance. J Biol Chem 282: 30794-30803.

71. Khokha MK, Hsu D, Brunet LJ, Dionne MS, Harland RM (2003) Gremlin is the BMP antagonist required for maintenance of Shh and Fgf signals during limb patterning. Nat Genet 34: 303-307.

72. Lu X, Borchers AG, Jolicoeur C, Rayburn H, Baker JC, et al. (2004) PTK7/CCK-4 is a novel regulator of planar cell polarity in vertebrates. Nature 430: 93-98.

73. Gurniak CB, Perlas E, Witke W (2005) The actin depolymerizing factor n-cofilin is essential for neural tube morphogenesis and neural crest cell migration. Dev Biol 278: 231-241.

74. Bladt F, Riethmacher D, Isenmann S, Aguzzi A, Birchmeier C (1995) Essential role for the c-met receptor in the migration of myogenic precursor cells into the limb bud. Nature 376: 768-771.

75. Pettitt SJ, Liang Q, Rairdan XY, Moran JL, Prosser HM, et al. (2009) Agouti C57BL/6N embryonic stem cells for mouse genetic resources. Nat Methods 6: 493-495.

76. Wang W, Wang YG, Reginato AM, Glotzer DJ, Fukai N, et al. (2004) Groucho homologue Grg5 interacts with the transcription factor Runx2-Cbfa1 and modulates its activity during postnatal growth in mice. Dev Biol 270: 364-381.

77. Banks KG, Johnson KA, Lerner CP, Mahaffey CL, Bronson RT, et al. (2003) Retroposon compensatory mechanism hypothesis not supported: Zfa knockout mice are fertile. Genomics 82: 254-260.

78. Yamada R, Mizutani-Koseki Y, Koseki H, Takahashi N (2004) Requirement for Mab21l2 during development of murine retina and ventral body wall. Dev Biol 274: 295-307.

79. Haberland M, Mokalled MH, Montgomery RL, Olson EN (2009) Epigenetic control of skull morphogenesis by histone deacetylase 8. Genes Dev 23: 1625-1630.

80. Bruneau S, Johnson KR, Yamamoto M, Kuroiwa A, Duboule D (2001) The mouse Hoxd13(spdh) mutation, a polyalanine expansion similar to human type II synpolydactyly (SPD), disrupts the function but not the expression of other Hoxd genes. Dev Biol 237: 345-353.

81. Bagi Z, Frangos JA, Yeh JC, White CR, Kaley G, et al. (2005) PECAM-1 mediates NO-dependent dilation of arterioles to high temporal gradients of shear stress. Arterioscler Thromb Vasc Biol 25: 1590-1595.

82. Mendelsohn C, Lohnes D, Decimo D, Lufkin T, LeMeur M, et al. (1994) Function of the retinoic acid receptors (RARs) during development (II). Multiple abnormalities at various stages of organogenesis in RAR double mutants. Development 120: 2749-2771.

83. Chiang YJ, Sommers CL, Jordan MS, Gu H, Samelson LE, et al. (2004) Inactivation of c-Cbl reverses neonatal lethality and T cell developmental arrest of SLP-76-deficient mice. J Exp Med 200: 25-34.

84. Gu JL, Muller S, Mancino V, Offermanns S, Simon MI (2002) Interaction of G alpha(12) with G alpha(13) and G alpha(q) signaling pathways. Proc Natl Acad Sci U S A 99: 9352-9357.

85. Rahuel C, Filipe A, Ritie L, El Nemer W, Patey-Mariaud N, et al. (2008) Genetic inactivation of the laminin alpha5 chain receptor Lu/BCAM leads to kidney and intestinal abnormalities in the mouse. Am J Physiol Renal Physiol 294: F393-406.

86. Gerety SS, Wang HU, Chen ZF, Anderson DJ (1999) Symmetrical mutant phenotypes of the receptor EphB4 and its specific transmembrane ligand ephrin-B2 in cardiovascular development. Mol Cell 4: 403-414.

87. Yamada G, Mansouri A, Torres M, Stuart ET, Blum M, et al. (1995) Targeted mutation of the murine goosecoid gene results in craniofacial defects and neonatal death. Development 121: 2917-2922.

88. Wirth KG, Wutz G, Kudo NR, Desdouets C, Zetterberg A, et al. (2006) Separase: a universal trigger for sister chromatid disjunction but not chromosome cycle progression. J Cell Biol 172: 847-860.

89. Scholzen TE, Steinhoff M, Bonaccorsi P, Klein R, Amadesi S, et al. (2001) Neutral endopeptidase terminates substance P-induced inflammation in allergic contact dermatitis. J Immunol 166: 1285-1291.

90. Liu CY, Birk DE, Hassell JR, Kane B, Kao WW (2003) Keratocan-deficient mice display alterations in corneal structure. J Biol Chem 278: 21672-21677.

91. Stadler HS, Higgins KM, Capecchi MR (2001) Loss of Eph-receptor expression correlates with loss of cell adhesion and chondrogenic capacity in Hoxa13 mutant limbs. Development 128: 4177-4188.

92. Storm EE, Kingsley DM (1996) Joint patterning defects caused by single and double mutations in members of the bone morphogenetic protein (BMP) family. Development 122: 3969-3979.

93. Diaz F, Thomas CK, Garcia S, Hernandez D, Moraes CT (2005) Mice lacking COX10 in skeletal muscle recapitulate the phenotype of progressive mitochondrial myopathies associated with cytochrome c oxidase deficiency. Hum Mol Genet 14: 2737-2748.

94. Rahkonen O, Su M, Hakovirta H, Koskivirta I, Hormuzdi SG, et al. (2004) Mice with a deletion in the first intron of the Col1a1 gene develop age-dependent aortic dissection and rupture. Circ Res 94: 83-90.

95. Lloyd C, Yu QC, Cheng J, Turksen K, Degenstein L, et al. (1995) The basal keratin network of stratified squamous epithelia: defining K15 function in the absence of K14. J Cell Biol 129: 1329-1344.

96. Zhang H, Lawson WE, Polosukhin VV, Pozzi A, Blackwell TS, et al. (2007) Inhibitor of differentiation 1 promotes endothelial survival in a bleomycin model of lung injury in mice. Am J Pathol 171: 1113-1126.

97. Ohbayashi N, Shibayama M, Kurotaki Y, Imanishi M, Fujimori T, et al. (2002) FGF18 is required for normal cell proliferation and differentiation during osteogenesis and chondrogenesis. Genes Dev 16: 870-879.

98. Tasheva ES, Koester A, Paulsen AQ, Garrett AS, Boyle DL, et al. (2002) Mimecan/osteoglycin-deficient mice have collagen fibril abnormalities. Mol Vis 8: 407-415.

99. Ibdah JA, Paul H, Zhao Y, Binford S, Salleng K, et al. (2001) Lack of mitochondrial trifunctional protein in mice causes neonatal hypoglycemia and sudden death. J Clin Invest 107: 1403-1409.

100. Preston P, Wartosch L, Gunzel D, Fromm M, Kongsuphol P, et al. Disruption of the K+ channel beta-subunit KCNE3 reveals an important role in intestinal and tracheal Cl- transport. J Biol Chem 285: 7165-7175.

101. Orkin RW, Williams BR, Cranley RE, Poppke DC, Brown KS (1977) Defects in the cartilaginous growth plates of brachymorphic mice. J Cell Biol 73: 287-299.

102. Tsukuba T, Okamoto K, Okamoto Y, Yanagawa M, Kohmura K, et al. (2003) Association of cathepsin E deficiency with development of atopic dermatitis. J Biochem 134: 893-902.

103. McGowan KM, Tong X, Colucci-Guyon E, Langa F, Babinet C, et al. (2002) Keratin 17 null mice exhibit age- and strain-dependent alopecia. Genes Dev 16: 1412-1422.

104. Sahr KE, Lambert AJ, Ciciotte SL, Mohandas N, Peters LL (2009) Targeted deletion of the gamma-adducin gene (Add3) in mice reveals differences in alpha-adducin interactions in erythroid and nonerythroid cells. Am J Hematol 84: 354-361.

105. Brunet LJ, McMahon JA, McMahon AP, Harland RM (1998) Noggin, cartilage morphogenesis, and joint formation in the mammalian skeleton. Science 280: 1455-1457.

106. Yae K, Keng VW, Koike M, Yusa K, Kouno M, et al. (2006) Sleeping beauty transposon-based phenotypic analysis of mice: lack of Arpc3 results in defective trophoblast outgrowth. Mol Cell Biol 26: 6185-6196.

107. Ning Y, Schuller AG, Bradshaw S, Rotwein P, Ludwig T, et al. (2006) Diminished growth and enhanced glucose metabolism in triple knockout mice containing mutations of insulin-like growth factor binding protein-3, -4, and -5. Mol Endocrinol 20: 2173-2186.

108. Sock E, Schmidt K, Hermanns-Borgmeyer I, Bosl MR, Wegner M (2001) Idiopathic weight reduction in mice deficient in the high-mobility-group transcription factor Sox8. Mol Cell Biol 21: 6951-6959.

109. Jerome-Majewska LA, Achkar T, Luo L, Lupu F, Lacy E The trafficking protein Tmed2/p24beta(1) is required for morphogenesis of the mouse embryo and placenta. Dev Biol 341: 154-166.

110. Graef IA, Chen F, Chen L, Kuo A, Crabtree GR (2001) Signals transduced by Ca(2+)/calcineurin and NFATc3/c4 pattern the developing vasculature. Cell 105: 863-875.

111. Watanabe T, Sato T, Amano T, Kawamura Y, Kawamura N, et al. (2008) Dnm3os, a non-coding RNA, is required for normal growth and skeletal development in mice. Dev Dyn 237: 3738-3748.

112. Bok D, Galbraith G, Lopez I, Woodruff M, Nusinowitz S, et al. (2003) Blindness and auditory impairment caused by loss of the sodium bicarbonate cotransporter NBC3. Nat Genet 34: 313-319.

113. O'Donnell N, Zachara NE, Hart GW, Marth JD (2004) Ogt-dependent X-chromosome-linked protein glycosylation is a requisite modification in somatic cell function and embryo viability. Mol Cell Biol 24: 1680-1690.

114. Babu GJ, Bhupathy P, Timofeyev V, Petrashevskaya NN, Reiser PJ, et al. (2007) Ablation of sarcolipin enhances sarcoplasmic reticulum calcium transport and atrial contractility. Proc Natl Acad Sci U S A 104: 17867-17872.

115. Li X, Liu P, Liu W, Maye P, Zhang J, et al. (2005) Dkk2 has a role in terminal osteoblast differentiation and mineralized matrix formation. Nat Genet 37: 945-952.

116. Munroe RJ, Bergstrom RA, Zheng QY, Libby B, Smith R, et al. (2000) Mouse mutants from chemically mutagenized embryonic stem cells. Nat Genet 24: 318-321.

117. Bussen M, Petry M, Schuster-Gossler K, Leitges M, Gossler A, et al. (2004) The T-box transcription factor Tbx18 maintains the separation of anterior and posterior somite compartments. Genes Dev 18: 1209-1221.

118. Ichikawa-Shindo Y, Sakurai T, Kamiyoshi A, Kawate H, Iinuma N, et al. (2008) The GPCR modulator protein RAMP2 is essential for angiogenesis and vascular integrity. J Clin Invest 118: 29-39.

119. Nguyen-Tran VT, Kubalak SW, Minamisawa S, Fiset C, Wollert KC, et al. (2000) A novel genetic pathway for sudden cardiac death via defects in the transition between ventricular and conduction system cell lineages. Cell 102: 671-682.

120. Sekita Y, Wagatsuma H, Nakamura K, Ono R, Kagami M, et al. (2008) Role of retrotransposon-derived imprinted gene, Rtl1, in the feto-maternal interface of mouse placenta. Nat Genet 40: 243-248.

121. Hansen J, Floss T, Van Sloun P, Fuchtbauer EM, Vauti F, et al. (2003) A large-scale, gene-driven mutagenesis approach for the functional analysis of the mouse genome. Proc Natl Acad Sci U S A 100: 9918-9922.

122. Schmahl J, Raymond CS, Soriano P (2007) PDGF signaling specificity is mediated through multiple immediate early genes. Nat Genet 39: 52-60.

123. Stenvers KL, Tursky ML, Harder KW, Kountouri N, Amatayakul-Chantler S, et al. (2003) Heart and liver defects and reduced transforming growth factor beta2 sensitivity in transforming growth factor beta type III receptor-deficient embryos. Mol Cell Biol 23: 4371-4385.

124. Chen C, Bharucha V, Chen Y, Westenbroek RE, Brown A, et al. (2002) Reduced sodium channel density, altered voltage dependence of inactivation, and increased susceptibility to seizures in mice lacking sodium channel beta 2-subunits. Proc Natl Acad Sci U S A 99: 17072-17077.

125. Siegenthaler JA, Ashique AM, Zarbalis K, Patterson KP, Hecht JH, et al. (2009) Retinoic acid from the meninges regulates cortical neuron generation. Cell 139: 597-609.

126. Chen J, Bush JO, Ovitt CE, Lan Y, Jiang R (2007) The TGF-beta pseudoreceptor gene Bambi is dispensable for mouse embryonic development and postnatal survival. Genesis 45: 482-486.

127. Isono K, Fujimura Y, Shinga J, Yamaki M, J OW, et al. (2005) Mammalian polyhomeotic homologues Phc2 and Phc1 act in synergy to mediate polycomb repression of Hox genes. Mol Cell Biol 25: 6694-6706.

128. Ansel KM, Ngo VN, Hyman PL, Luther SA, Forster R, et al. (2000) A chemokine-driven positive feedback loop organizes lymphoid follicles. Nature 406: 309-314.

129. Metcalf D, Greenhalgh CJ, Viney E, Willson TA, Starr R, et al. (2000) Gigantism in mice lacking suppressor of cytokine signalling-2. Nature 405: 1069-1073.

130. Palmer S, Groves N, Schindeler A, Yeoh T, Biben C, et al. (2001) The small muscle-specific protein Csl modifies cell shape and promotes myocyte fusion in an insulin-like growth factor 1-dependent manner. J Cell Biol 153: 985-998.

131. Lorenz-Depiereux B, Guido VE, Johnson KR, Zheng QY, Gagnon LH, et al. (2004) New intragenic deletions in the Phex gene clarify X-linked hypophosphatemia-related abnormalities in mice. Mamm Genome 15: 151-161.

132. Shamblott MJ, Bugg EM, Lawler AM, Gearhart JD (2002) Craniofacial abnormalities resulting from targeted disruption of the murine Sim2 gene. Dev Dyn 224: 373-380.

133. Lai DM, Tu YK, Hsieh YH, Hsu WM, Lee CC, et al. (2007) Angiopoietin-like protein 1 expression is related to intermuscular connective tissue and cartilage development. Dev Dyn 236: 2643-2652.

134. Rodriguez TA, Sparrow DB, Scott AN, Withington SL, Preis JI, et al. (2004) Cited1 is required in trophoblasts for placental development and for embryo growth and survival. Mol Cell Biol 24: 228-244.

135. van der Weyden L, Wei L, Luo J, Yang X, Birk DE, et al. (2006) Functional knockout of the matrilin-3 gene causes premature chondrocyte maturation to hypertrophy and increases bone mineral density and osteoarthritis. Am J Pathol 169: 515-527.

136. Wellik DM, Capecchi MR (2003) Hox10 and Hox11 genes are required to globally pattern the mammalian skeleton. Science 301: 363-367.

137. Lanctot C, Moreau A, Chamberland M, Tremblay ML, Drouin J (1999) Hindlimb patterning and mandible development require the Ptx1 gene. Development 126: 1805-1810.

138. Menke DB, Guenther C, Kingsley DM (2008) Dual hindlimb control elements in the Tbx4 gene and region-specific control of bone size in vertebrate limbs. Development 135: 2543-2553.

139. Lupu F, Terwilliger JD, Lee K, Segre GV, Efstratiadis A (2001) Roles of growth hormone and insulin-like growth factor 1 in mouse postnatal growth. Dev Biol 229: 141-162.

140. Seo KW, Wang Y, Kokubo H, Kettlewell JR, Zarkower DA, et al. (2006) Targeted disruption of the DM domain containing transcription factor Dmrt2 reveals an essential role in somite patterning. Dev Biol 290: 200-210.

141. Hildebrand JD, Soriano P (2002) Overlapping and unique roles for C-terminal binding protein 1 (CtBP1) and CtBP2 during mouse development. Mol Cell Biol 22: 5296-5307.

142. Ning Y, Schuller AG, Bradshaw S, Rotwein P, Ludwig T, et al. (2006) Diminished growth and enhanced glucose metabolism in triple knockout mice containing mutations of insulin-like growth factor binding protein-3, -4, and -5. Mol Endocrinol 20: 2173-2186.

143. Meffre E, Nussenzweig MC (2002) Deletion of immunoglobulin beta in developing B cells leads to cell death. Proc Natl Acad Sci U S A 99: 11334-11339.

144. Yu H, Smallwood PM, Wang Y, Vidaltamayo R, Reed R, et al. Frizzled 1 and frizzled 2 genes function in palate, ventricular septum and neural tube closure: general implications for tissue fusion processes. Development 137: 3707-3717.

145. Bjork BC, Turbe-Doan A, Prysak M, Herron BJ, Beier DR Prdm16 is required for normal palatogenesis in mice. Hum Mol Genet 19: 774-789.

146. Gonzalez-Garcia A, Pritchard CA, Paterson HF, Mavria G, Stamp G, et al. (2005) RalGDS is required for tumor formation in a model of skin carcinogenesis. Cancer Cell 7: 219-226.

147. Caubit X, Lye CM, Martin E, Core N, Long DA, et al. (2008) Teashirt 3 is necessary for ureteral smooth muscle differentiation downstream of SHH and BMP4. Development 135: 3301-3310.

148. Ferdous A, Caprioli A, Iacovino M, Martin CM, Morris J, et al. (2009) Nkx2-5 transactivates the Ets-related protein 71 gene and specifies an endothelial/endocardial fate in the developing embryo. Proc Natl Acad Sci U S A 106: 814-819.

149. Hertveldt V, Louryan S, van Reeth T, Dreze P, van Vooren P, et al. (2008) The development of several organs and appendages is impaired in mice lacking Sp6. Dev Dyn 237: 883-892.

150. Cenci S, Weitzmann MN, Gentile MA, Aisa MC, Pacifici R (2000) M-CSF neutralization and egr-1 deficiency prevent ovariectomy-induced bone loss. J Clin Invest 105: 1279-1287.

151. Schinzel AC, Takeuchi O, Huang Z, Fisher JK, Zhou Z, et al. (2005) Cyclophilin D is a component of mitochondrial permeability transition and mediates neuronal cell death after focal cerebral ischemia. Proc Natl Acad Sci U S A 102: 12005-12010.

152. Yang LV, Radu CG, Roy M, Lee S, McLaughlin J, et al. (2007) Vascular abnormalities in mice deficient for the G protein-coupled receptor GPR4 that functions as a pH sensor. Mol Cell Biol 27: 1334-1347.

153. Wang Y, Vachon E, Zhang J, Cherepanov V, Kruger J, et al. (2005) Tyrosine phosphatase MEG2 modulates murine development and platelet and lymphocyte activation through secretory vesicle function. J Exp Med 202: 1587-1597.

154. Friedel RH, Kerjan G, Rayburn H, Schuller U, Sotelo C, et al. (2007) Plexin-B2 controls the development of cerebellar granule cells. J Neurosci 27: 3921-3932.

155. Zhang CL, McKinsey TA, Chang S, Antos CL, Hill JA, et al. (2002) Class II histone deacetylases act as signal-responsive repressors of cardiac hypertrophy. Cell 110: 479-488.

156. Enkhmandakh B, Makeyev AV, Bayarsaihan D (2006) The role of the proline-rich domain of Ssdp1 in the modular architecture of the vertebrate head organizer. Proc Natl Acad Sci U S A 103: 11631-11636.

157. Stephen LJ, Fawkes AL, Verhoeve A, Lemke G, Brown A (2007) A critical role for the EphA3 receptor tyrosine kinase in heart development. Dev Biol 302: 66-79.

158. Mocsai A, Humphrey MB, Van Ziffle JA, Hu Y, Burghardt A, et al. (2004) The immunomodulatory adapter proteins DAP12 and Fc receptor gamma-chain (FcRgamma) regulate development of functional osteoclasts through the Syk tyrosine kinase. Proc Natl Acad Sci U S A 101: 6158-6163.

159. Cullen M, Seaman S, Chaudhary A, Yang MY, Hilton MB, et al. (2009) Host-derived tumor endothelial marker 8 promotes the growth of melanoma. Cancer Res 69: 6021-6026.

160. Nada S, Yagi T, Takeda H, Tokunaga T, Nakagawa H, et al. (1993) Constitutive activation of Src family kinases in mouse embryos that lack Csk. Cell 73: 1125-1135.

161. Morello R, Bertin TK, Chen Y, Hicks J, Tonachini L, et al. (2006) CRTAP is required for prolyl 3- hydroxylation and mutations cause recessive osteogenesis imperfecta. Cell 127: 291-304.

162. Ihrie RA, Reczek E, Horner JS, Khachatrian L, Sage J, et al. (2003) Perp is a mediator of p53-dependent apoptosis in diverse cell types. Curr Biol 13: 1985-1990.

163. Nanda A, Karim B, Peng Z, Liu G, Qiu W, et al. (2006) Tumor endothelial marker 1 (Tem1) functions in the growth and progression of abdominal tumors. Proc Natl Acad Sci U S A 103: 3351-3356.

164. Abraira VE, Hyun N, Tucker AF, Coling DE, Brown MC, et al. (2007) Changes in Sef levels influence auditory brainstem development and function. J Neurosci 27: 4273-4282.

165. Wu M, Chen DF, Sasaoka T, Tonegawa S (1996) Neural tube defects and abnormal brain development in F52-deficient mice. Proc Natl Acad Sci U S A 93: 2110-2115.

166. Hoffman LM, Jensen CC, Kloeker S, Wang CL, Yoshigi M, et al. (2006) Genetic ablation of zyxin causes Mena/VASP mislocalization, increased motility, and deficits in actin remodeling. J Cell Biol 172: 771-782.

167. Kvajo M, Albrecht H, Meins M, Hengst U, Troncoso E, et al. (2004) Regulation of brain proteolytic activity is necessary for the in vivo function of NMDA receptors. J Neurosci 24: 9734-9743.

168. Ueki K, Yballe CM, Brachmann SM, Vicent D, Watt JM, et al. (2002) Increased insulin sensitivity in mice lacking p85beta subunit of phosphoinositide 3-kinase. Proc Natl Acad Sci U S A 99: 419-424.

169. Cortes VA, Curtis DE, Sukumaran S, Shao X, Parameswara V, et al. (2009) Molecular mechanisms of hepatic steatosis and insulin resistance in the AGPAT2-deficient mouse model of congenital generalized lipodystrophy. Cell Metab 9: 165-176.

170. Yong W, Bao S, Chen H, Li D, Sanchez ER, et al. (2007) Mice lacking protein phosphatase 5 are defective in ataxia telangiectasia mutated (ATM)-mediated cell cycle arrest. J Biol Chem 282: 14690-14694.

171. Trumpp A, Refaeli Y, Oskarsson T, Gasser S, Murphy M, et al. (2001) c-Myc regulates mammalian body size by controlling cell number but not cell size. Nature 414: 768-773.

172. Houle M, Sylvestre JR, Lohnes D (2003) Retinoic acid regulates a subset of Cdx1 function in vivo. Development 130: 6555-6567.

173. Szumlinski KK, Dehoff MH, Kang SH, Frys KA, Lominac KD, et al. (2004) Homer proteins regulate sensitivity to cocaine. Neuron 43: 401-413.

174. Ishida D, Kometani K, Yang H, Kakugawa K, Masuda K, et al. (2003) Myeloproliferative stem cell disorders by deregulated Rap1 activation in SPA-1-deficient mice. Cancer Cell 4: 55-65.

175. Auman HJ, Nottoli T, Lakiza O, Winger Q, Donaldson S, et al. (2002) Transcription factor AP-2gamma is essential in the extra-embryonic lineages for early postimplantation development. Development 129: 2733-2747.

176. Homeister JW, Thall AD, Petryniak B, Maly P, Rogers CE, et al. (2001) The alpha(1,3)fucosyltransferases FucT-IV and FucT-VII exert collaborative control over selectin-dependent leukocyte recruitment and lymphocyte homing. Immunity 15: 115-126.

177. Heurteaux C, Guy N, Laigle C, Blondeau N, Duprat F, et al. (2004) TREK-1, a K+ channel involved in neuroprotection and general anesthesia. Embo J 23: 2684-2695.

178. Onodera K, Shavit JA, Motohashi H, Katsuoka F, Akasaka JE, et al. (1999) Characterization of the murine mafF gene. J Biol Chem 274: 21162-21169.

179. MacLennan AJ, Benner SJ, Andringa A, Chaves AH, Rosing JL, et al. (2006) The S1P2 sphingosine 1-phosphate receptor is essential for auditory and vestibular function. Hear Res 220: 38-48.

180. Sawada A, Kiyonari H, Ukita K, Nishioka N, Imuta Y, et al. (2008) Redundant roles of Tead1 and Tead2 in notochord development and the regulation of cell proliferation and survival. Mol Cell Biol 28: 3177-3189.

181. Cullinan EB, Kwee L, Nunes P, Shuster DJ, Ju G, et al. (1998) IL-1 receptor accessory protein is an essential component of the IL-1 receptor. J Immunol 161: 5614-5620.

182. Ansorge HL, Meng X, Zhang G, Veit G, Sun M, et al. (2009) Type XIV Collagen Regulates Fibrillogenesis: PREMATURE COLLAGEN FIBRIL GROWTH AND TISSUE DYSFUNCTION IN NULL MICE. J Biol Chem 284: 8427-8438.

183. Molkentin JD, Tymitz KM, Richardson JA, Olson EN (2000) Abnormalities of the genitourinary tract in female mice lacking GATA5. Mol Cell Biol 20: 5256-5260.

184. Zirzow S, Ludtke TH, Brons JF, Petry M, Christoffels VM, et al. (2009) Expression and requirement of T-box transcription factors Tbx2 and Tbx3 during secondary palate development in the mouse. Dev Biol 336: 145-155.

185. Church C, Lee S, Bagg EA, McTaggart JS, Deacon R, et al. (2009) A mouse model for the metabolic effects of the human fat mass and obesity associated FTO gene. PLoS Genet 5: e1000599.

186. Pang J, Hoefen R, Pryhuber GS, Wang J, Yin G, et al. (2009) G-protein-coupled receptor kinase interacting protein-1 is required for pulmonary vascular development. Circulation 119: 1524-1532.

187. Chandrasekharan S, Foley NA, Jania L, Clark P, Audoly LP, et al. (2005) Coupling of COX-1 to mPGES1 for prostaglandin E2 biosynthesis in the murine mammary gland. J Lipid Res 46: 2636-2648.

188. Francois M, Caprini A, Hosking B, Orsenigo F, Wilhelm D, et al. (2008) Sox18 induces development of the lymphatic vasculature in mice. Nature 456: 643-647.

189. Yonemasu R, Minami M, Nakatsu Y, Takeuchi M, Kuraoka I, et al. (2005) Disruption of mouse XAB2 gene involved in pre-mRNA splicing, transcription and transcription-coupled DNA repair results in preimplantation lethality. DNA Repair (Amst) 4: 479-491.

190. Hayashi K, Cao T, Passmore H, Jourdan-Le Saux C, Fogelgren B, et al. (2004) Progressive hair loss and myocardial degeneration in rough coat mice: reduced lysyl oxidase-like (LOXL) in the skin and heart. J Invest Dermatol 123: 864-871.

191. Lo SH, Yu QC, Degenstein L, Chen LB, Fuchs E (1997) Progressive kidney degeneration in mice lacking tensin. J Cell Biol 136: 1349-1361.

192. Lu LY, Wood JL, Minter-Dykhouse K, Ye L, Saunders TL, et al. (2008) Polo-like kinase 1 is essential for early embryonic development and tumor suppression. Mol Cell Biol 28: 6870-6876.

193. Jiang R, Lan Y, Chapman HD, Shawber C, Norton CR, et al. (1998) Defects in limb, craniofacial, and thymic development in Jagged2 mutant mice. Genes Dev 12: 1046-1057.

194. Yuan X, Zhou Y, Casanova E, Chai M, Kiss E, et al. (2005) Genetic inactivation of the transcription factor TIF-IA leads to nucleolar disruption, cell cycle arrest, and p53-mediated apoptosis. Mol Cell 19: 77-87.

195. Sofaer JA (1969) Aspects of the tabby-crinkled-downless syndrome. II. Observations on the reaction to changes of genetic background. J Embryol Exp Morphol 22: 207-227.

196. Shindo T, Kurihara H, Kuno K, Yokoyama H, Wada T, et al. (2000) ADAMTS-1: a metalloproteinase-disintegrin essential for normal growth, fertility, and organ morphology and function. J Clin Invest 105: 1345-1352.

197. Sun Y, Boyd K, Xu W, Ma J, Jackson CW, et al. (2006) Acute myeloid leukemia-associated Mkl1 (Mrtf-a) is a key regulator of mammary gland function. Mol Cell Biol 26: 5809-5826.

198. Habib GM, Shi ZZ, Cuevas AA, Guo Q, Matzuk MM, et al. (1998) Leukotriene D4 and cystinyl-bis-glycine metabolism in membrane-bound dipeptidase-deficient mice. Proc Natl Acad Sci U S A 95: 4859-4863.

199. Danko I, Chapman V, Wolff JA (1992) The frequency of revertants in mdx mouse genetic models for Duchenne muscular dystrophy. Pediatr Res 32: 128-131.

200. Basson MA, Akbulut S, Watson-Johnson J, Simon R, Carroll TJ, et al. (2005) Sprouty1 is a critical regulator of GDNF/RET-mediated kidney induction. Dev Cell 8: 229-239.

201. Morgans CW, Zhang J, Jeffrey BG, Nelson SM, Burke NS, et al. (2009) TRPM1 is required for the depolarizing light response in retinal ON-bipolar cells. Proc Natl Acad Sci U S A 106: 19174-19178.

202. Johnson KR, Gagnon LH, Webb LS, Peters LL, Hawes NL, et al. (2003) Mouse models of USH1C and DFNB18: phenotypic and molecular analyses of two new spontaneous mutations of the Ush1c gene. Hum Mol Genet 12: 3075-3086.

203. Kobayashi Y, Watanabe M, Okada Y, Sawa H, Takai H, et al. (2002) Hydrocephalus, situs inversus, chronic sinusitis, and male infertility in DNA polymerase lambda-deficient mice: possible implication for the pathogenesis of immotile cilia syndrome. Mol Cell Biol 22: 2769-2776.

204. Mi Y, Fiete D, Baenziger JU (2008) Ablation of GalNAc-4-sulfotransferase-1 enhances reproduction by altering the carbohydrate structures of luteinizing hormone in mice. J Clin Invest 118: 1815-1824.

205. Wynick D, Small CJ, Bacon A, Holmes FE, Norman M, et al. (1998) Galanin regulates prolactin release and lactotroph proliferation. Proc Natl Acad Sci U S A 95: 12671-12676.

206. Yang G, Wu L, Jiang B, Yang W, Qi J, et al. (2008) H2S as a physiologic vasorelaxant: hypertension in mice with deletion of cystathionine gamma-lyase. Science 322: 587-590.

207. Parr BA, McMahon AP (1995) Dorsalizing signal Wnt-7a required for normal polarity of D-V and A-P axes of mouse limb. Nature 374: 350-353.

208. Lanske B, Karaplis AC, Lee K, Luz A, Vortkamp A, et al. (1996) PTH/PTHrP receptor in early development and Indian hedgehog-regulated bone growth. Science 273: 663-666.

209. Cheng A, Arumugam TV, Liu D, Khatri RG, Mustafa K, et al. (2007) Pancortin-2 interacts with WAVE1 and Bcl-xL in a mitochondria-associated protein complex that mediates ischemic neuronal death. J Neurosci 27: 1519-1528.

210. Li CY, Jepsen KJ, Majeska RJ, Zhang J, Ni R, et al. (2006) Mice lacking cathepsin K maintain bone remodeling but develop bone fragility despite high bone mass. J Bone Miner Res 21: 865-875.

211. Stone SJ, Myers HM, Watkins SM, Brown BE, Feingold KR, et al. (2004) Lipopenia and skin barrier abnormalities in DGAT2-deficient mice. J Biol Chem 279: 11767-11776.

212. Horan GS, Wu K, Wolgemuth DJ, Behringer RR (1994) Homeotic transformation of cervical vertebrae in Hoxa-4 mutant mice. Proc Natl Acad Sci U S A 91: 12644-12648.

213. Aubin J, Lemieux M, Tremblay M, Berard J, Jeannotte L (1997) Early postnatal lethality in Hoxa-5 mutant mice is attributable to respiratory tract defects. Dev Biol 192: 432-445.

214. Lu X, Le Noble F, Yuan L, Jiang Q, De Lafarge B, et al. (2004) The netrin receptor UNC5B mediates guidance events controlling morphogenesis of the vascular system. Nature 432: 179-186.

215. Tatum R, Zhang Y, Salleng K, Lu Z, Lin JJ, et al. Renal salt wasting and chronic dehydration in claudin-7-deficient mice. Am J Physiol Renal Physiol 298: F24-34.

216. Kruger M, Moser M, Ussar S, Thievessen I, Luber CA, et al. (2008) SILAC mouse for quantitative proteomics uncovers kindlin-3 as an essential factor for red blood cell function. Cell 134: 353-364.

217. Chae HJ, Kim HR, Xu C, Bailly-Maitre B, Krajewska M, et al. (2004) BI-1 regulates an apoptosis pathway linked to endoplasmic reticulum stress. Mol Cell 15: 355-366.

218. Soriano P, Montgomery C, Geske R, Bradley A (1991) Targeted disruption of the c-src proto-oncogene leads to osteopetrosis in mice. Cell 64: 693-702.

219. Muto A, Tashiro S, Nakajima O, Hoshino H, Takahashi S, et al. (2004) The transcriptional programme of antibody class switching involves the repressor Bach2. Nature 429: 566-571.

220. Tse E, Smith AJ, Hunt S, Lavenir I, Forster A, et al. (2004) Null mutation of the Lmo4 gene or a combined null mutation of the Lmo1/Lmo3 genes causes perinatal lethality, and Lmo4 controls neural tube development in mice. Mol Cell Biol 24: 2063-2073.

221. Fu L, Patel MS, Bradley A, Wagner EF, Karsenty G (2005) The molecular clock mediates leptin-regulated bone formation. Cell 122: 803-815.

222. Christie GR, Williams DJ, Macisaac F, Dickinson RJ, Rosewell I, et al. (2005) The dual-specificity protein phosphatase DUSP9/MKP-4 is essential for placental function but is not required for normal embryonic development. Mol Cell Biol 25: 8323-8333.

223. Qiu M, Bulfone A, Ghattas I, Meneses JJ, Christensen L, et al. (1997) Role of the Dlx homeobox genes in proximodistal patterning of the branchial arches: mutations of Dlx-1, Dlx-2, and Dlx-1 and -2 alter morphogenesis of proximal skeletal and soft tissue structures derived from the first and second arches. Dev Biol 185: 165-184.

224. Rancourt DE, Tsuzuki T, Capecchi MR (1995) Genetic interaction between hoxb-5 and hoxb-6 is revealed by nonallelic noncomplementation. Genes Dev 9: 108-122.

225. Charrier E, Mosinger B, Meissirel C, Aguera M, Rogemond V, et al. (2006) Transient alterations in granule cell proliferation, apoptosis and migration in postnatal developing cerebellum of CRMP1-/- mice. Genes Cells 11: 1337-1352.

226. Zhao Q, Behringer RR, de Crombrugghe B (1996) Prenatal folic acid treatment suppresses acrania and meroanencephaly in mice mutant for the Cart1 homeobox gene. Nat Genet 13: 275-283.

227. Bell SM, Schreiner CM, Hess KA, Anderson KP, Scott WJ (2003) Asymmetric limb malformations in a new transgene insertional mutant, footless. Mech Dev 120: 597-605.

228. Esworthy RS, Mann JR, Sam M, Chu FF (2000) Low glutathione peroxidase activity in Gpx1 knockout mice protects jejunum crypts from gamma-irradiation damage. Am J Physiol Gastrointest Liver Physiol 279: G426-436.

229. Gainetdinov RR, Bohn LM, Walker JK, Laporte SA, Macrae AD, et al. (1999) Muscarinic supersensitivity and impaired receptor desensitization in G protein-coupled receptor kinase 5-deficient mice. Neuron 24: 1029-1036.

230. Arthur JS, Elce JS, Hegadorn C, Williams K, Greer PA (2000) Disruption of the murine calpain small subunit gene, Capn4: calpain is essential for embryonic development but not for cell growth and division. Mol Cell Biol 20: 4474-4481.

231. Hsu CY, Chang NC, Lee MW, Lee KH, Sun DS, et al. (2008) LUZP deficiency affects neural tube closure during brain development. Biochem Biophys Res Commun 376: 466-471.

232. Chen MH, Li YJ, Kawakami T, Xu SM, Chuang PT (2004) Palmitoylation is required for the production of a soluble multimeric Hedgehog protein complex and long-range signaling in vertebrates. Genes Dev 18: 641-659.

233. Chao JR, Parganas E, Boyd K, Hong CY, Opferman JT, et al. (2008) Hax1-mediated processing of HtrA2 by Parl allows survival of lymphocytes and neurons. Nature 452: 98-102.

234. Lichtenauer UD, Duchniewicz M, Kolanczyk M, Hoeflich A, Hahner S, et al. (2007) Pre-B-cell transcription factor 1 and steroidogenic factor 1 synergistically regulate adrenocortical growth and steroidogenesis. Endocrinology 148: 693-704.

235. Takeshima H, Iino M, Takekura H, Nishi M, Kuno J, et al. (1994) Excitation-contraction uncoupling and muscular degeneration in mice lacking functional skeletal muscle ryanodine-receptor gene. Nature 369: 556-559.

236. Tedford K, Nitschke L, Girkontaite I, Charlesworth A, Chan G, et al. (2001) Compensation between Vav-1 and Vav-2 in B cell development and antigen receptor signaling. Nat Immunol 2: 548-555.

237. Chang M, Jin W, Sun SC (2009) Peli1 facilitates TRIF-dependent Toll-like receptor signaling and proinflammatory cytokine production. Nat Immunol 10: 1089-1095.

238. Stoller JZ, Huang L, Tan CC, Huang F, Zhou DD, et al. Ash2l interacts with Tbx1 and is required during early embryogenesis. Exp Biol Med (Maywood) 235: 569-576.

239. Yuan W, Rao Y, Babiuk RP, Greer JJ, Wu JY, et al. (2003) A genetic model for a central (septum transversum) congenital diaphragmatic hernia in mice lacking Slit3. Proc Natl Acad Sci U S A 100: 5217-5222.

240. de Geest N, Bonten E, Mann L, de Sousa-Hitzler J, Hahn C, et al. (2002) Systemic and neurologic abnormalities distinguish the lysosomal disorders sialidosis and galactosialidosis in mice. Hum Mol Genet 11: 1455-1464.

241. Rantakari P, Lagerbohm H, Kaimainen M, Suomela JP, Strauss L, et al. Hydroxysteroid (17{beta}) dehydrogenase 12 is essential for mouse organogenesis and embryonic survival. Endocrinology 151: 1893-1901.

242. Wang G, Zhang J, Moskophidis D, Mivechi NF (2003) Targeted disruption of the heat shock transcription factor (hsf)-2 gene results in increased embryonic lethality, neuronal defects, and reduced spermatogenesis. Genesis 36: 48-61.

243. Yan W, Assadi AH, Wynshaw-Boris A, Eichele G, Matzuk MM, et al. (2003) Previously uncharacterized roles of platelet-activating factor acetylhydrolase 1b complex in mouse spermatogenesis. Proc Natl Acad Sci U S A 100: 7189-7194.

244. Humbert PO, Rogers C, Ganiatsas S, Landsberg RL, Trimarchi JM, et al. (2000) E2F4 is essential for normal erythrocyte maturation and neonatal viability. Mol Cell 6: 281-291.

245. Andre P, Prasad KS, Denis CV, He M, Papalia JM, et al. (2002) CD40L stabilizes arterial thrombi by a beta3 integrin--dependent mechanism. Nat Med 8: 247-252.

246. Contos JJ, Fukushima N, Weiner JA, Kaushal D, Chun J (2000) Requirement for the lpA1 lysophosphatidic acid receptor gene in normal suckling behavior. Proc Natl Acad Sci U S A 97: 13384-13389.

247. Nonaka S, Tanaka Y, Okada Y, Takeda S, Harada A, et al. (1998) Randomization of left-right asymmetry due to loss of nodal cilia generating leftward flow of extraembryonic fluid in mice lacking KIF3B motor protein. Cell 95: 829-837.

248. Tu CF, Yan YT, Wu SY, Djoko B, Tsai MT, et al. (2008) Domain and functional analysis of a novel platelet-endothelial cell surface protein, SCUBE1. J Biol Chem 283: 12478-12488.

249. Jaehnig EJ, Heidt AB, Greene SB, Cornelissen I, Black BL (2006) Increased susceptibility to isoproterenol-induced cardiac hypertrophy and impaired weight gain in mice lacking the histidine-rich calcium-binding protein. Mol Cell Biol 26: 9315-9326.

250. Drusco A, Zanesi N, Roldo C, Trapasso F, Farber JL, et al. (2005) Knockout mice reveal a tumor suppressor function for Testin. Proc Natl Acad Sci U S A 102: 10947-10951.

251. Meier H, Chai CK (1970) Spastic, an hereditary neurological mutation in the mouse characterized by vertebral arthropathy and leptomeningeal cyst formation. Exp Med Surg 28: 24-38.

252. Houghtaling S, Timmers C, Noll M, Finegold MJ, Jones SN, et al. (2003) Epithelial cancer in Fanconi anemia complementation group D2 (Fancd2) knockout mice. Genes Dev 17: 2021-2035.

253. Lyon JB, Jr. (1970) The X-chromosome and the enzymes controlling muscle glycogen: phosphorylase kinase. Biochem Genet 4: 169-185.

254. Maeda A, Maeda T, Imanishi Y, Sun W, Jastrzebska B, et al. (2006) Retinol dehydrogenase (RDH12) protects photoreceptors from light-induced degeneration in mice. J Biol Chem 281: 37697-37704.

255. Karolyi IJ, Burrows HL, Ramesh TM, Nakajima M, Lesh JS, et al. (1999) Altered anxiety and weight gain in corticotropin-releasing hormone-binding protein-deficient mice. Proc Natl Acad Sci U S A 96: 11595-11600.

256. Irie HY, Mong MS, Itano A, Crooks ME, Littman DR, et al. (1998) The cytoplasmic domain of CD8 beta regulates Lck kinase activation and CD8 T cell development. J Immunol 161: 183-191.

257. Roest HP, Baarends WM, de Wit J, van Klaveren JW, Wassenaar E, et al. (2004) The ubiquitin-conjugating DNA repair enzyme HR6A is a maternal factor essential for early embryonic development in mice. Mol Cell Biol 24: 5485-5495.

258. Terasawa Y, Ladha Z, Leonard SW, Morrow JD, Newland D, et al. (2000) Increased atherosclerosis in hyperlipidemic mice deficient in alpha -tocopherol transfer protein and vitamin E. Proc Natl Acad Sci U S A 97: 13830-13834.

259. Chen H, Mocsai A, Zhang H, Ding RX, Morisaki JH, et al. (2003) Role for plastin in host defense distinguishes integrin signaling from cell adhesion and spreading. Immunity 19: 95-104.

260. Elrick MJ, Pacheco CD, Yu T, Dadgar N, Shakkottai VG, et al. Conditional Niemann-Pick C mice demonstrate cell autonomous Purkinje cell neurodegeneration. Hum Mol Genet 19: 837-847.

261. Tsunoda T, Takashima Y, Tanaka Y, Fujimoto T, Doi K, et al. Immune-related zinc finger gene ZFAT is an essential transcriptional regulator for hematopoietic differentiation in blood islands. Proc Natl Acad Sci U S A 107: 14199-14204.

262. Elms P, Siggers P, Napper D, Greenfield A, Arkell R (2003) Zic2 is required for neural crest formation and hindbrain patterning during mouse development. Dev Biol 264: 391-406.

263. Reimold AM, Grusby MJ, Kosaras B, Fries JW, Mori R, et al. (1996) Chondrodysplasia and neurological abnormalities in ATF-2-deficient mice. Nature 379: 262-265.

264. Katayama K, Zine A, Ota M, Matsumoto Y, Inoue T, et al. (2009) Disorganized innervation and neuronal loss in the inner ear of Slitrk6-deficient mice. PLoS One 4: e7786.

265. Thomas T, Voss AK, Chowdhury K, Gruss P (2000) Querkopf, a MYST family histone acetyltransferase, is required for normal cerebral cortex development. Development 127: 2537-2548.

266. Kenner L, Hoebertz A, Beil T, Keon N, Karreth F, et al. (2004) Mice lacking JunB are osteopenic due to cell-autonomous osteoblast and osteoclast defects. J Cell Biol 164: 613-623.

267. Kang X, Qi Y, Zuo Y, Wang Q, Zou Y, et al. SUMO-specific protease 2 is essential for suppression of polycomb group protein-mediated gene silencing during embryonic development. Mol Cell 38: 191-201.

268. Schnell S, Demolliere C, van den Berk P, Jacobs H (2006) Gimap4 accelerates T-cell death. Blood 108: 591-599.

269. Takano J, Tomioka M, Tsubuki S, Higuchi M, Iwata N, et al. (2005) Calpain mediates excitotoxic DNA fragmentation via mitochondrial pathways in adult brains: evidence from calpastatin mutant mice. J Biol Chem 280: 16175-16184.

270. Vessey JP, Macchi P, Stein JM, Mikl M, Hawker KN, et al. (2008) A loss of function allele for murine Staufen1 leads to impairment of dendritic Staufen1-RNP delivery and dendritic spine morphogenesis. Proc Natl Acad Sci U S A 105: 16374-16379.

271. Golonzhka O, Liang X, Messaddeq N, Bornert JM, Campbell AL, et al. (2009) Dual role of COUP-TF-interacting protein 2 in epidermal homeostasis and permeability barrier formation. J Invest Dermatol 129: 1459-1470.

272. Jackowski S, Rehg JE, Zhang YM, Wang J, Miller K, et al. (2004) Disruption of CCTbeta2 expression leads to gonadal dysfunction. Mol Cell Biol 24: 4720-4733.

273. Ueta M, Hamuro J, Ueda E, Katoh N, Yamamoto M, et al. (2008) Stat6-independent tissue inflammation occurs selectively on the ocular surface and perioral skin of IkappaBzeta-/- mice. Invest Ophthalmol Vis Sci 49: 3387-3394.

274. Hartmann D, de Strooper B, Serneels L, Craessaerts K, Herreman A, et al. (2002) The disintegrin/metalloprotease ADAM 10 is essential for Notch signalling but not for alpha-secretase activity in fibroblasts. Hum Mol Genet 11: 2615-2624.

275. Brancho D, Ventura JJ, Jaeschke A, Doran B, Flavell RA, et al. (2005) Role of MLK3 in the regulation of mitogen-activated protein kinase signaling cascades. Mol Cell Biol 25: 3670-3681.

276. Kriz V, Mares J, Wentzel P, Funa NS, Calounova G, et al. (2007) Shb null allele is inherited with a transmission ratio distortion and causes reduced viability in utero. Dev Dyn 236: 2485-2492.

277. Vickers CA, Stephens B, Bowen J, Arbuthnott GW, Grant SG, et al. (2006) Neurone specific regulation of dendritic spines in vivo by post synaptic density 95 protein (PSD-95). Brain Res 1090: 89-98.

278. Yu L, Ji W, Zhang H, Renda MJ, He Y, et al. SENP1-mediated GATA1 deSUMOylation is critical for definitive erythropoiesis. J Exp Med 207: 1183-1195.

279. Singer JD, Gurian-West M, Clurman B, Roberts JM (1999) Cullin-3 targets cyclin E for ubiquitination and controls S phase in mammalian cells. Genes Dev 13: 2375-2387.

280. Renckens R, Roelofs JJ, de Waard V, Florquin S, Lijnen HR, et al. (2005) The role of plasminogen activator inhibitor type 1 in the inflammatory response to local tissue injury. J Thromb Haemost 3: 1018-1025.

281. Nunez Rodriguez N, Lee IN, Banno A, Qiao HF, Qiao RF, et al. (2006) Characterization of R-ras3/m-ras null mice reveals a potential role in trophic factor signaling. Mol Cell Biol 26: 7145-7154.

282. Qu X, Yu J, Bhagat G, Furuya N, Hibshoosh H, et al. (2003) Promotion of tumorigenesis by heterozygous disruption of the beclin 1 autophagy gene. J Clin Invest 112: 1809-1820.

283. Lu Q, Lemke G (2001) Homeostatic regulation of the immune system by receptor tyrosine kinases of the Tyro 3 family. Science 293: 306-311.

284. Gosling KM, Makaroff LE, Theodoratos A, Kim YH, Whittle B, et al. (2007) A mutation in a chromosome condensin II subunit, kleisin beta, specifically disrupts T cell development. Proc Natl Acad Sci U S A 104: 12445-12450.

285. Nozaki M, Ohishi K, Yamada N, Kinoshita T, Nagy A, et al. (1999) Developmental abnormalities of glycosylphosphatidylinositol-anchor-deficient embryos revealed by Cre/loxP system. Lab Invest 79: 293-299.

286. Schreiber-Agus N, Meng Y, Hoang T, Hou H, Jr., Chen K, et al. (1998) Role of Mxi1 in ageing organ systems and the regulation of normal and neoplastic growth. Nature 393: 483-487.

287. Cox KB, Hamm DA, Millington DS, Matern D, Vockley J, et al. (2001) Gestational, pathologic and biochemical differences between very long-chain acyl-CoA dehydrogenase deficiency and long-chain acyl-CoA dehydrogenase deficiency in the mouse. Hum Mol Genet 10: 2069-2077.

288. Horan GS, Kovacs EN, Behringer RR, Featherstone MS (1995) Mutations in paralogous Hox genes result in overlapping homeotic transformations of the axial skeleton: evidence for unique and redundant function. Dev Biol 169: 359-372.

289. Liu L, Brown D, McKee M, Lebrasseur NK, Yang D, et al. (2008) Deletion of Cavin/PTRF causes global loss of caveolae, dyslipidemia, and glucose intolerance. Cell Metab 8: 310-317.

290. Pouillon V, Hascakova-Bartova R, Pajak B, Adam E, Bex F, et al. (2003) Inositol 1,3,4,5-tetrakisphosphate is essential for T lymphocyte development. Nat Immunol 4: 1136-1143.

291. Suzuki N, Labosky PA, Furuta Y, Hargett L, Dunn R, et al. (1996) Failure of ventral body wall closure in mouse embryos lacking a procollagen C-proteinase encoded by Bmp1, a mammalian gene related to Drosophila tolloid. Development 122: 3587-3595.

292. Li L, Keverne EB, Aparicio SA, Ishino F, Barton SC, et al. (1999) Regulation of maternal behavior and offspring growth by paternally expressed Peg3. Science 284: 330-333.

293. Li X, Ominsky MS, Niu QT, Sun N, Daugherty B, et al. (2008) Targeted deletion of the sclerostin gene in mice results in increased bone formation and bone strength. J Bone Miner Res 23: 860-869.

294. Mukhopadhyay M, Shtrom S, Rodriguez-Esteban C, Chen L, Tsukui T, et al. (2001) Dickkopf1 is required for embryonic head induction and limb morphogenesis in the mouse. Dev Cell 1: 423-434.

295. Uemura T, Lee SJ, Yasumura M, Takeuchi T, Yoshida T, et al. Trans-synaptic interaction of GluRdelta2 and Neurexin through Cbln1 mediates synapse formation in the cerebellum. Cell 141: 1068-1079.

296. van Ree JH, Hofker MH, van den Broek WJ, van Deursen JM, van der Boom H, et al. (1995) Increased response to cholesterol feeding in apolipoprotein C1-deficient mice. Biochem J 305 (Pt 3): 905-911.

297. Shiow LR, Roadcap DW, Paris K, Watson SR, Grigorova IL, et al. (2008) The actin regulator coronin 1A is mutant in a thymic egress-deficient mouse strain and in a patient with severe combined immunodeficiency. Nat Immunol 9: 1307-1315.

298. Arimitsu N, Akimitsu N, Kotani N, Takasaki S, Kina T, et al. (2003) Glycophorin A requirement for expression of O-linked antigens on the erythrocyte membrane. Genes Cells 8: 769-777.

299. Khanna R, Chang SH, Andrabi S, Azam M, Kim A, et al. (2002) Headpiece domain of dematin is required for the stability of the erythrocyte membrane. Proc Natl Acad Sci U S A 99: 6637-6642.

300. Kioka N, Ito T, Yamashita H, Uekawa N, Umemoto T, et al. Crucial role of vinexin for keratinocyte migration in vitro and epidermal wound healing in vivo. Exp Cell Res 316: 1728-1738.

301. Colgan J, Asmal M, Neagu M, Yu B, Schneidkraut J, et al. (2004) Cyclophilin A regulates TCR signal strength in CD4+ T cells via a proline-directed conformational switch in Itk. Immunity 21: 189-201.

302. Vartanian V, Lowell B, Minko IG, Wood TG, Ceci JD, et al. (2006) The metabolic syndrome resulting from a knockout of the NEIL1 DNA glycosylase. Proc Natl Acad Sci U S A 103: 1864-1869.

303. Kimura H, Miyashita H, Suzuki Y, Kobayashi M, Watanabe K, et al. (2009) Distinctive localization and opposed roles of vasohibin-1 and vasohibin-2 in the regulation of angiogenesis. Blood 113: 4810-4818.

304. Fejes-Toth G, Frindt G, Naray-Fejes-Toth A, Palmer LG (2008) Epithelial Na+ channel activation and processing in mice lacking SGK1. Am J Physiol Renal Physiol 294: F1298-1305.

305. Nakamichi Y, Shukunami C, Yamada T, Aihara K, Kawano H, et al. (2003) Chondromodulin I is a bone remodeling factor. Mol Cell Biol 23: 636-644.

306. Pennacchio LA, Bouley DM, Higgins KM, Scott MP, Noebels JL, et al. (1998) Progressive ataxia, myoclonic epilepsy and cerebellar apoptosis in cystatin B-deficient mice. Nat Genet 20: 251-258.

307. Oliver ER, Saunders TL, Tarle SA, Glaser T (2004) Ribosomal protein L24 defect in belly spot and tail (Bst), a mouse Minute. Development 131: 3907-3920.

308. Zhou X, Solaroli N, Bjerke M, Stewart JB, Rozell B, et al. (2008) Progressive loss of mitochondrial DNA in thymidine kinase 2-deficient mice. Hum Mol Genet 17: 2329-2335.

309. Grisendi S, Bernardi R, Rossi M, Cheng K, Khandker L, et al. (2005) Role of nucleophosmin in embryonic development and tumorigenesis. Nature 437: 147-153.

310. Yang Y, Dieter MZ, Chen Y, Shertzer HG, Nebert DW, et al. (2002) Initial characterization of the glutamate-cysteine ligase modifier subunit Gclm(-/-) knockout mouse. Novel model system for a severely compromised oxidative stress response. J Biol Chem 277: 49446-49452.

311. Hayashi M, Kim SW, Imanaka-Yoshida K, Yoshida T, Abel ED, et al. (2004) Targeted deletion of BMK1/ERK5 in adult mice perturbs vascular integrity and leads to endothelial failure. J Clin Invest 113: 1138-1148.

312. Condac E, Silasi-Mansat R, Kosanke S, Schoeb T, Towner R, et al. (2007) Polycystic disease caused by deficiency in xylosyltransferase 2, an initiating enzyme of glycosaminoglycan biosynthesis. Proc Natl Acad Sci U S A 104: 9416-9421.

313. Wempe F, De-Zolt S, Koli K, Bangsow T, Parajuli N, et al. Inactivation of sestrin 2 induces TGF-beta signaling and partially rescues pulmonary emphysema in a mouse model of COPD. Dis Model Mech 3: 246-253.

314. Nyman LR, Cox KB, Hoppel CL, Kerner J, Barnoski BL, et al. (2005) Homozygous carnitine palmitoyltransferase 1a (liver isoform) deficiency is lethal in the mouse. Mol Genet Metab 86: 179-187.

315. Jell J, Merali S, Hensen ML, Mazurchuk R, Spernyak JA, et al. (2007) Genetically altered expression of spermidine/spermine N1-acetyltransferase affects fat metabolism in mice via acetyl-CoA. J Biol Chem 282: 8404-8413.

316. Changolkar LN, Costanzi C, Leu NA, Chen D, McLaughlin KJ, et al. (2007) Developmental changes in histone macroH2A1-mediated gene regulation. Mol Cell Biol 27: 2758-2764.

317. Tominaga K, Kirtane B, Jackson JG, Ikeno Y, Ikeda T, et al. (2005) MRG15 regulates embryonic development and cell proliferation. Mol Cell Biol 25: 2924-2937.

318. Yeh WC, Pompa JL, McCurrach ME, Shu HB, Elia AJ, et al. (1998) FADD: essential for embryo development and signaling from some, but not all, inducers of apoptosis. Science 279: 1954-1958.

319. Vranka JA, Pokidysheva E, Hayashi L, Zientek K, Mizuno K, et al. Prolyl 3-hydroxylase 1 null mice display abnormalities in fibrillar collagen-rich tissues such as tendons, skin, and bones. J Biol Chem 285: 17253-17262.

320. Takaluoma K, Hyry M, Lantto J, Sormunen R, Bank RA, et al. (2007) Tissue-specific changes in the hydroxylysine content and cross-links of collagens and alterations in fibril morphology in lysyl hydroxylase 1 knock-out mice. J Biol Chem 282: 6588-6596.

321. Finn RD, McLaughlin LA, Ronseaux S, Rosewell I, Houston JB, et al. (2008) Defining the in Vivo Role for cytochrome b5 in cytochrome P450 function through the conditional hepatic deletion of microsomal cytochrome b5. J Biol Chem 283: 31385-31393.

322. Azam M, Andrabi SS, Sahr KE, Kamath L, Kuliopulos A, et al. (2001) Disruption of the mouse mu-calpain gene reveals an essential role in platelet function. Mol Cell Biol 21: 2213-2220.

323. Li S, Zhou D, Lu MM, Morrisey EE (2004) Advanced cardiac morphogenesis does not require heart tube fusion. Science 305: 1619-1622.

324. Kirn-Safran CB, Oristian DS, Focht RJ, Parker SG, Vivian JL, et al. (2007) Global growth deficiencies in mice lacking the ribosomal protein HIP/RPL29. Dev Dyn 236: 447-460.

325. Stauffer EA, Scarborough JD, Hirono M, Miller ED, Shah K, et al. (2005) Fast adaptation in vestibular hair cells requires myosin-1c activity. Neuron 47: 541-553.

326. Xu ZP, Wawrousek EF, Piatigorsky J (2002) Transketolase haploinsufficiency reduces adipose tissue and female fertility in mice. Mol Cell Biol 22: 6142-6147.

327. Suzuki T, Li W, Zhang Q, Karim A, Novak EK, et al. (2002) Hermansky-Pudlak syndrome is caused by mutations in HPS4, the human homolog of the mouse light-ear gene. Nat Genet 30: 321-324.

328. Dasgupta B, Milbrandt J (2009) AMP-activated protein kinase phosphorylates retinoblastoma protein to control mammalian brain development. Dev Cell 16: 256-270.

329. Li JP, Gong F, Hagner-McWhirter A, Forsberg E, Abrink M, et al. (2003) Targeted disruption of a murine glucuronyl C5-epimerase gene results in heparan sulfate lacking L-iduronic acid and in neonatal lethality. J Biol Chem 278: 28363-28366.

330. Yazdanpanah B, Wiegmann K, Tchikov V, Krut O, Pongratz C, et al. (2009) Riboflavin kinase couples TNF receptor 1 to NADPH oxidase. Nature 460: 1159-1163.

331. Mao C, Wang M, Luo B, Wey S, Dong D, et al. Targeted mutation of the mouse Grp94 gene disrupts development and perturbs endoplasmic reticulum stress signaling. PLoS One 5: e10852.

332. Lopez-Molina L, Conquet F, Dubois-Dauphin M, Schibler U (1997) The DBP gene is expressed according to a circadian rhythm in the suprachiasmatic nucleus and influences circadian behavior. Embo J 16: 6762-6771.

333. Cox S, Smith L, Bogani D, Cheeseman M, Siggers P, et al. (2006) Sexually dimorphic expression of secreted frizzled-related (SFRP) genes in the developing mouse Mullerian duct. Mol Reprod Dev 73: 1008-1016.

334. Perarnau B, Saron MF, Reina San Martin B, Bervas N, Ong H, et al. (1999) Single H2Kb, H2Db and double H2KbDb knockout mice: peripheral CD8+ T cell repertoire and anti-lymphocytic choriomeningitis virus cytolytic responses. Eur J Immunol 29: 1243-1252.

335. Sirotkin AM, Edelmann W, Cheng G, Klein-Szanto A, Kucherlapati R, et al. (1995) Mice develop normally without the H1(0) linker histone. Proc Natl Acad Sci U S A 92: 6434-6438.

336. Andra K, Lassmann H, Bittner R, Shorny S, Fassler R, et al. (1997) Targeted inactivation of plectin reveals essential function in maintaining the integrity of skin, muscle, and heart cytoarchitecture. Genes Dev 11: 3143-3156.

337. Anderson SJ, Lauritsen JP, Hartman MG, Foushee AM, Lefebvre JM, et al. (2007) Ablation of ribosomal protein L22 selectively impairs alphabeta T cell development by activation of a p53-dependent checkpoint. Immunity 26: 759-772.

338. Couldrey C, Carlton MB, Nolan PM, Colledge WH, Evans MJ (1999) A retroviral gene trap insertion into the histone 3.3A gene causes partial neonatal lethality, stunted growth, neuromuscular deficits and male sub-fertility in transgenic mice. Hum Mol Genet 8: 2489-2495.

339. Huang QQ, Hossain MM, Wu K, Parai K, Pope RM, et al. (2008) Role of H2-calponin in regulating macrophage motility and phagocytosis. J Biol Chem 283: 25887-25899.

340. Carter MG, Johns MA, Zeng X, Zhou L, Zink MC, et al. (2000) Mice deficient in the candidate tumor suppressor gene Hic1 exhibit developmental defects of structures affected in the Miller-Dieker syndrome. Hum Mol Genet 9: 413-419.

341. Cheng CH, Kikuchi T, Chen YH, Sabbagha NG, Lee YC, et al. (2009) Mutations in the SLC2A10 gene cause arterial abnormalities in mice. Cardiovasc Res 81: 381-388.

342. Venuti JM, Morris JH, Vivian JL, Olson EN, Klein WH (1995) Myogenin is required for late but not early aspects of myogenesis during mouse development. J Cell Biol 128: 563-576.

343. Sanjo H, Takeda K, Tsujimura T, Ninomiya-Tsuji J, Matsumoto K, et al. (2003) TAB2 is essential for prevention of apoptosis in fetal liver but not for interleukin-1 signaling. Mol Cell Biol 23: 1231-1238.

344. Enkhmandakh B, Makeyev AV, Erdenechimeg L, Ruddle FH, Chimge NO, et al. (2009) Essential functions of the Williams-Beuren syndrome-associated TFII-I genes in embryonic development. Proc Natl Acad Sci U S A 106: 181-186.

345. Fan Y, Nikitina T, Morin-Kensicki EM, Zhao J, Magnuson TR, et al. (2003) H1 linker histones are essential for mouse development and affect nucleosome spacing in vivo. Mol Cell Biol 23: 4559-4572.

346. Wang L, Yang L, Debidda M, Witte D, Zheng Y (2007) Cdc42 GTPase-activating protein deficiency promotes genomic instability and premature aging-like phenotypes. Proc Natl Acad Sci U S A 104: 1248-1253.

347. Ye X, Wang Y, Cahill H, Yu M, Badea TC, et al. (2009) Norrin, frizzled-4, and Lrp5 signaling in endothelial cells controls a genetic program for retinal vascularization. Cell 139: 285-298.

348. Rethinasamy P, Muthuchamy M, Hewett T, Boivin G, Wolska BM, et al. (1998) Molecular and physiological effects of alpha-tropomyosin ablation in the mouse. Circ Res 82: 116-123.

349. Mitchell KJ, Pinson KI, Kelly OG, Brennan J, Zupicich J, et al. (2001) Functional analysis of secreted and transmembrane proteins critical to mouse development. Nat Genet 28: 241-249.

350. Yu D, Zhang H, Blanpied TA, Smith E, Zhan X Cortactin is implicated in murine zygotic development. Exp Cell Res 316: 848-858.

351. Wagner DS, Gan L, Klein WH (1998) Expression of a gene trap reporter construct in a subset of cells in embryonic sites of hematopoiesis: evidence for alternative rRNA production in hematopoietic cells. Biochem Biophys Res Commun 250: 674-681.

352. Skaar JR, Arai T, DeCaprio JA (2005) Dimerization of CUL7 and PARC is not required for all CUL7 functions and mouse development. Mol Cell Biol 25: 5579-5589.

353. Xie W, Samoriski GM, McLaughlin JP, Romoser VA, Smrcka A, et al. (1999) Genetic alteration of phospholipase C beta3 expression modulates behavioral and cellular responses to mu opioids. Proc Natl Acad Sci U S A 96: 10385-10390.

354. Botto M, Dell'Agnola C, Bygrave AE, Thompson EM, Cook HT, et al. (1998) Homozygous C1q deficiency causes glomerulonephritis associated with multiple apoptotic bodies. Nat Genet 19: 56-59.

355. Newhall KJ, Criniti AR, Cheah CS, Smith KC, Kafer KE, et al. (2006) Dynamic anchoring of PKA is essential during oocyte maturation. Curr Biol 16: 321-327.

356. Tanaka T, Soriano MA, Grusby MJ (2005) SLIM is a nuclear ubiquitin E3 ligase that negatively regulates STAT signaling. Immunity 22: 729-736.

357. Bonaldo P, Braghetta P, Zanetti M, Piccolo S, Volpin D, et al. (1998) Collagen VI deficiency induces early onset myopathy in the mouse: an animal model for Bethlem myopathy. Hum Mol Genet 7: 2135-2140.

358. Shu Z, Smith S, Wang L, Rice MC, Kmiec EB (1999) Disruption of muREC2/RAD51L1 in mice results in early embryonic lethality which can Be partially rescued in a p53(-/-) background. Mol Cell Biol 19: 8686-8693.

359. Murakami H, Okawa A, Yoshida H, Nishikawa S, Moriya H, et al. (2002) Elbow knee synostosis (Eks): a new mutation on mouse Chromosome 14. Mamm Genome 13: 341-344.

360. Fernandez-Marcos PJ, Pantoja C, Gonzalez-Rodriguez A, Martin N, Flores JM, et al. Normal proliferation and tumorigenesis but impaired pancreatic function in mice lacking the cell cycle regulator sei1. PLoS One 5: e8744.

361. Pan Y, Zvaritch E, Tupling AR, Rice WJ, de Leon S, et al. (2003) Targeted disruption of the ATP2A1 gene encoding the sarco(endo)plasmic reticulum Ca2+ ATPase isoform 1 (SERCA1) impairs diaphragm function and is lethal in neonatal mice. J Biol Chem 278: 13367-13375.

362. Tsukahara T, Okamura M, Suzuki S, Iwata H, Miura T, et al. (1991) Enhanced expression of fibronectin by cmd/cmd chondrocytes and its modulation by exogenously added proteoglycan. J Cell Sci 100 (Pt 2): 387-395.

363. Yamao T, Noguchi T, Takeuchi O, Nishiyama U, Morita H, et al. (2002) Negative regulation of platelet clearance and of the macrophage phagocytic response by the transmembrane glycoprotein SHPS-1. J Biol Chem 277: 39833-39839.

364. Lefebvre L, Viville S, Barton SC, Ishino F, Keverne EB, et al. (1998) Abnormal maternal behaviour and growth retardation associated with loss of the imprinted gene Mest. Nat Genet 20: 163-169.

365. Lindsay EA, Botta A, Jurecic V, Carattini-Rivera S, Cheah YC, et al. (1999) Congenital heart disease in mice deficient for the DiGeorge syndrome region. Nature 401: 379-383.

366. Ryu KY, Maehr R, Gilchrist CA, Long MA, Bouley DM, et al. (2007) The mouse polyubiquitin gene UbC is essential for fetal liver development, cell-cycle progression and stress tolerance. Embo J 26: 2693-2706.

367. Hopfer U, Fukai N, Hopfer H, Wolf G, Joyce N, et al. (2005) Targeted disruption of Col8a1 and Col8a2 genes in mice leads to anterior segment abnormalities in the eye. Faseb J 19: 1232-1244.

368. Du H, Duanmu M, Witte D, Grabowski GA (1998) Targeted disruption of the mouse lysosomal acid lipase gene: long-term survival with massive cholesteryl ester and triglyceride storage. Hum Mol Genet 7: 1347-1354.

369. Bianco SD, Peng JB, Takanaga H, Suzuki Y, Crescenzi A, et al. (2007) Marked disturbance of calcium homeostasis in mice with targeted disruption of the Trpv6 calcium channel gene. J Bone Miner Res 22: 274-285.

370. Akama TO, Nakagawa H, Sugihara K, Narisawa S, Ohyama C, et al. (2002) Germ cell survival through carbohydrate-mediated interaction with Sertoli cells. Science 295: 124-127.

371. Wirth KG, Ricci R, Gimenez-Abian JF, Taghybeeglu S, Kudo NR, et al. (2004) Loss of the anaphase-promoting complex in quiescent cells causes unscheduled hepatocyte proliferation. Genes Dev 18: 88-98.

372. Pretsch W, Favor J (2007) Genetic, biochemical, and molecular characterization of nine glyceraldehyde-3-phosphate dehydrogenase mutants with reduced enzyme activity in Mus musculus. Mamm Genome 18: 686-692.

373. Fowler KJ, Hudson DF, Salamonsen LA, Edmondson SR, Earle E, et al. (2000) Uterine dysfunction and genetic modifiers in centromere protein B-deficient mice. Genome Res 10: 30-41.

374. Mijimolle N, Velasco J, Dubus P, Guerra C, Weinbaum CA, et al. (2005) Protein farnesyltransferase in embryogenesis, adult homeostasis, and tumor development. Cancer Cell 7: 313-324.

375. Ikeda S, Shiva N, Ikeda A, Smith RS, Nusinowitz S, et al. (2000) Retinal degeneration but not obesity is observed in null mutants of the tubby-like protein 1 gene. Hum Mol Genet 9: 155-163.

376. Bilinski P, Roopenian D, Gossler A (1998) Maternal IL-11Ralpha function is required for normal decidua and fetoplacental development in mice. Genes Dev 12: 2234-2243.

377. Takemoto-Kimura S, Ageta-Ishihara N, Nonaka M, Adachi-Morishima A, Mano T, et al. (2007) Regulation of dendritogenesis via a lipid-raft-associated Ca2+/calmodulin-dependent protein kinase CLICK-III/CaMKIgamma. Neuron 54: 755-770.

378. Shimano H, Shimomura I, Hammer RE, Herz J, Goldstein JL, et al. (1997) Elevated levels of SREBP-2 and cholesterol synthesis in livers of mice homozygous for a targeted disruption of the SREBP-1 gene. J Clin Invest 100: 2115-2124.

379. Sumara G, Formentini I, Collins S, Sumara I, Windak R, et al. (2009) Regulation of PKD by the MAPK p38delta in insulin secretion and glucose homeostasis. Cell 136: 235-248.

380. Rao P, Hayden MS, Long M, Scott ML, West AP, et al. IkappaBbeta acts to inhibit and activate gene expression during the inflammatory response. Nature 466: 1115-1119.

381. Kwon MC, Koo BK, Moon JS, Kim YY, Park KC, et al. (2008) Crif1 is a novel transcriptional coactivator of STAT3. Embo J 27: 642-653.

382. Yoshida T, Yamanaka K, Atsumi S, Tsumura H, Sasaki R, et al. (1994) A novel hypothyroid 'growth-retarded' mouse derived from Snell's dwarf mouse. J Endocrinol 142: 435-446.

383. Le Cam L, Lacroix M, Ciemerych MA, Sardet C, Sicinski P (2004) The E4F protein is required for mitotic progression during embryonic cell cycles. Mol Cell Biol 24: 6467-6475.

384. Steingrimsson E, Tessarollo L, Pathak B, Hou L, Arnheiter H, et al. (2002) Mitf and Tfe3, two members of the Mitf-Tfe family of bHLH-Zip transcription factors, have important but functionally redundant roles in osteoclast development. Proc Natl Acad Sci U S A 99: 4477-4482.

385. Kasarskis A, Manova K, Anderson KV (1998) A phenotype-based screen for embryonic lethal mutations in the mouse. Proc Natl Acad Sci U S A 95: 7485-7490.

386. Sollars VE, McEntee BJ, Engiles JB, Rothstein JL, Buchberg AM (2002) A novel transgenic line of mice exhibiting autosomal recessive male-specific lethality and non-alcoholic fatty liver disease. Hum Mol Genet 11: 2777-2786.

387. Bornstein P, McKinney CE, LaMarca ME, Winfield S, Shingu T, et al. (1995) Metaxin, a gene contiguous to both thrombospondin 3 and glucocerebrosidase, is required for embryonic development in the mouse: implications for Gaucher disease. Proc Natl Acad Sci U S A 92: 4547-4551.

388. Shi ZT, Afzal V, Coller B, Patel D, Chasis JA, et al. (1999) Protein 4.1R-deficient mice are viable but have erythroid membrane skeleton abnormalities. J Clin Invest 103: 331-340.

389. Brenner R, Chen QH, Vilaythong A, Toney GM, Noebels JL, et al. (2005) BK channel beta4 subunit reduces dentate gyrus excitability and protects against temporal lobe seizures. Nat Neurosci 8: 1752-1759.

390. Hansen J, Floss T, Van Sloun P, Fuchtbauer EM, Vauti F, et al. (2003) A large-scale, gene-driven mutagenesis approach for the functional analysis of the mouse genome. Proc Natl Acad Sci U S A 100: 9918-9922.

391. Chittenden TW, Claes F, Lanahan AA, Autiero M, Palac RT, et al. (2006) Selective regulation of arterial branching morphogenesis by synectin. Dev Cell 10: 783-795.

392. Buis J, Wu Y, Deng Y, Leddon J, Westfield G, et al. (2008) Mre11 nuclease activity has essential roles in DNA repair and genomic stability distinct from ATM activation. Cell 135: 85-96.

393. Iwanaga A, Sato T, Sugihara K, Hirao A, Takakura N, et al. (2007) Neural-specific ablation of the scaffold protein JSAP1 in mice causes neonatal death. Neurosci Lett 429: 43-48.

394. Bellomo D, Headrick JP, Silins GU, Paterson CA, Thomas PS, et al. (2000) Mice lacking the vascular endothelial growth factor-B gene (Vegfb) have smaller hearts, dysfunctional coronary vasculature, and impaired recovery from cardiac ischemia. Circ Res 86: E29-35.

395. Liu Y, Wada R, Yamashita T, Mi Y, Deng CX, et al. (2000) Edg-1, the G protein-coupled receptor for sphingosine-1-phosphate, is essential for vascular maturation. J Clin Invest 106: 951-961.

396. Glogauer M, Marchal CC, Zhu F, Worku A, Clausen BE, et al. (2003) Rac1 deletion in mouse neutrophils has selective effects on neutrophil functions. J Immunol 170: 5652-5657.

397. Svenningsson P, Chergui K, Rachleff I, Flajolet M, Zhang X, et al. (2006) Alterations in 5-HT1B receptor function by p11 in depression-like states. Science 311: 77-80.

398. Savolainen K, Kotti TJ, Schmitz W, Savolainen TI, Sormunen RT, et al. (2004) A mouse model for alpha-methylacyl-CoA racemase deficiency: adjustment of bile acid synthesis and intolerance to dietary methyl-branched lipids. Hum Mol Genet 13: 955-965.

399. Ellson C, Davidson K, Anderson K, Stephens LR, Hawkins PT (2006) PtdIns3P binding to the PX domain of p40phox is a physiological signal in NADPH oxidase activation. Embo J 25: 4468-4478.

400. Tsukiyama-Kohara K, Poulin F, Kohara M, DeMaria CT, Cheng A, et al. (2001) Adipose tissue reduction in mice lacking the translational inhibitor 4E-BP1. Nat Med 7: 1128-1132.

401. Lombard DB, Alt FW, Cheng HL, Bunkenborg J, Streeper RS, et al. (2007) Mammalian Sir2 homolog SIRT3 regulates global mitochondrial lysine acetylation. Mol Cell Biol 27: 8807-8814.

402. Chen NJ, Chio, II, Lin WJ, Duncan G, Chau H, et al. (2008) Beyond tumor necrosis factor receptor: TRADD signaling in toll-like receptors. Proc Natl Acad Sci U S A 105: 12429-12434.

403. Chen W, Liang Y, Deng W, Shimizu K, Ashique AM, et al. (2003) The zinc-finger protein CNBP is required for forebrain formation in the mouse. Development 130: 1367-1379.

404. Miura M, Chen XD, Allen MR, Bi Y, Gronthos S, et al. (2004) A crucial role of caspase-3 in osteogenic differentiation of bone marrow stromal stem cells. J Clin Invest 114: 1704-1713.

405. Shibayama H, Takai E, Matsumura I, Kouno M, Morii E, et al. (2004) Identification of a cytokine-induced antiapoptotic molecule anamorsin essential for definitive hematopoiesis. J Exp Med 199: 581-592.

406. Dabovic B, Chen Y, Colarossi C, Obata H, Zambuto L, et al. (2002) Bone abnormalities in latent TGF-[beta] binding protein (Ltbp)-3-null mice indicate a role for Ltbp-3 in modulating TGF-[beta] bioavailability. J Cell Biol 156: 227-232.

407. Horiguchi M, Inoue T, Ohbayashi T, Hirai M, Noda K, et al. (2009) Fibulin-4 conducts proper elastogenesis via interaction with cross-linking enzyme lysyl oxidase. Proc Natl Acad Sci U S A 106: 19029-19034.

408. Nishimura DY, Fath M, Mullins RF, Searby C, Andrews M, et al. (2004) Bbs2-null mice have neurosensory deficits, a defect in social dominance, and retinopathy associated with mislocalization of rhodopsin. Proc Natl Acad Sci U S A 101: 16588-16593.

409. Pan J, Nakade K, Huang YC, Zhu ZW, Masuzaki S, et al. Suppression of cell-cycle progression by Jun dimerization protein-2 (JDP2) involves downregulation of cyclin-A2. Oncogene 29: 6245-6256.

410. Dionne MS, Skarnes WC, Harland RM (2001) Mutation and analysis of Dan, the founding member of the Dan family of transforming growth factor beta antagonists. Mol Cell Biol 21: 636-643.

411. Sampath AP, Strissel KJ, Elias R, Arshavsky VY, McGinnis JF, et al. (2005) Recoverin improves rod-mediated vision by enhancing signal transmission in the mouse retina. Neuron 46: 413-420.

412. Kuster JE, Guarnieri MH, Ault JG, Flaherty L, Swiatek PJ (1997) IAP insertion in the murine LamB3 gene results in junctional epidermolysis bullosa. Mamm Genome 8: 673-681.

413. Bozza M, Satoskar AR, Lin G, Lu B, Humbles AA, et al. (1999) Targeted disruption of migration inhibitory factor gene reveals its critical role in sepsis. J Exp Med 189: 341-346.

414. Campbell PK, Waymire KG, Heier RL, Sharer C, Day DE, et al. (2002) Mutation of a novel gene results in abnormal development of spermatid flagella, loss of intermale aggression and reduced body fat in mice. Genetics 162: 307-320.

415. Ng JM, Vermeulen W, van der Horst GT, Bergink S, Sugasawa K, et al. (2003) A novel regulation mechanism of DNA repair by damage-induced and RAD23-dependent stabilization of xeroderma pigmentosum group C protein. Genes Dev 17: 1630-1645.

416. Flentjar NJ; Crack PJ; Boyd R; Malin M; de Haan JB; Hertzog P; Kola I; Iannello R (2002) Mice lacking glutathione peroxidase-1 activity show increased tunel staining and an accelerated inflammatory response in brain following a cold-induced injury.

417. Kong Y, Zhou S, Kihm AJ, Katein AM, Yu X, et al. (2004) Loss of alpha-hemoglobin-stabilizing protein impairs erythropoiesis and exacerbates beta-thalassemia. J Clin Invest 114: 1457-1466.

418. Spencer SD, Di Marco F, Hooley J, Pitts-Meek S, Bauer M, et al. (1998) The orphan receptor CRF2-4 is an essential subunit of the interleukin 10 receptor. J Exp Med 187: 571-578.

419. Barclay JL, Kerr LM, Arthur L, Rowland JE, Nelson CN, et al. In vivo targeting of the growth hormone receptor (GHR) Box1 sequence demonstrates that the GHR does not signal exclusively through JAK2. Mol Endocrinol 24: 204-217.

420. Schubert W, Sotgia F, Cohen AW, Capozza F, Bonuccelli G, et al. (2007) Caveolin-1(-/-)- and caveolin-2(-/-)-deficient mice both display numerous skeletal muscle abnormalities, with tubular aggregate formation. Am J Pathol 170: 316-333.

421. Chevessier F, Girard E, Molgo J, Bartling S, Koenig J, et al. (2008) A mouse model for congenital myasthenic syndrome due to MuSK mutations reveals defects in structure and function of neuromuscular junctions. Hum Mol Genet 17: 3577-3595.

422. Li X, Ito M, Zhou F, Youngson N, Zuo X, et al. (2008) A maternal-zygotic effect gene, Zfp57, maintains both maternal and paternal imprints. Dev Cell 15: 547-557.

423. Kim JY, Kang YS, Lee JW, Kim HJ, Ahn YH, et al. (2002) p38 is essential for the assembly and stability of macromolecular tRNA synthetase complex: implications for its physiological significance. Proc Natl Acad Sci U S A 99: 7912-7916.

424. Wakefield L, Cornish V, Long H, Griffiths WJ, Sim E (2007) Deletion of a xenobiotic metabolizing gene in mice affects folate metabolism. Biochem Biophys Res Commun 364: 556-560.

425. Guo L, Zhao D, Song Y, Meng Y, Zhao H, et al. (2007) Reduced urea flux across the blood-testis barrier and early maturation in the male reproductive system in UT-B-null mice. Am J Physiol Cell Physiol 293: C305-312.

426. Mahendroo MS, Cala KM, Hess DL, Russell DW (2001) Unexpected virilization in male mice lacking steroid 5 alpha-reductase enzymes. Endocrinology 142: 4652-4662.

427. Yuan L, Liu JG, Zhao J, Brundell E, Daneholt B, et al. (2000) The murine SCP3 gene is required for synaptonemal complex assembly, chromosome synapsis, and male fertility. Mol Cell 5: 73-83.

428. Tam SY, Tsai M, Snouwaert JN, Kalesnikoff J, Scherrer D, et al. (2004) RabGEF1 is a negative regulator of mast cell activation and skin inflammation. Nat Immunol 5: 844-852.

429. Trivedi SG, Newson J, Rajakariar R, Jacques TS, Hannon R, et al. (2006) Essential role for hematopoietic prostaglandin D2 synthase in the control of delayed type hypersensitivity. Proc Natl Acad Sci U S A 103: 5179-5184.

430. Mashimo T, Hadjebi O, Amair-Pinedo F, Tsurumi T, Langa F, et al. (2009) Progressive Purkinje cell degeneration in tambaleante mutant mice is a consequence of a missense mutation in HERC1 E3 ubiquitin ligase. PLoS Genet 5: e1000784.

431. Wigle JT, Oliver G (1999) Prox1 function is required for the development of the murine lymphatic system. Cell 98: 769-778.

432. Liu X, Cheng KT, Bandyopadhyay BC, Pani B, Dietrich A, et al. (2007) Attenuation of store-operated Ca2+ current impairs salivary gland fluid secretion in TRPC1(-/-) mice. Proc Natl Acad Sci U S A 104: 17542-17547.

433. Muller U, Wang D, Denda S, Meneses JJ, Pedersen RA, et al. (1997) Integrin alpha8beta1 is critically important for epithelial-mesenchymal interactions during kidney morphogenesis. Cell 88: 603-613.

434. Baynash AG, Hosoda K, Giaid A, Richardson JA, Emoto N, et al. (1994) Interaction of endothelin-3 with endothelin-B receptor is essential for development of epidermal melanocytes and enteric neurons. Cell 79: 1277-1285.

435. Yang X, Tang J, Rogler CE, Stanley P (2003) Reduced hepatocyte proliferation is the basis of retarded liver tumor progression and liver regeneration in mice lacking N-acetylglucosaminyltransferase III. Cancer Res 63: 7753-7759.

436. Backman M, Machon O, Van Den Bout CJ, Krauss S (2003) Targeted disruption of mouse Dach1 results in postnatal lethality. Dev Dyn 226: 139-144.

437. Beigneux AP, Vergnes L, Qiao X, Quatela S, Davis R, et al. (2006) Agpat6--a novel lipid biosynthetic gene required for triacylglycerol production in mammary epithelium. J Lipid Res 47: 734-744.

438. Catalani E, Cervia D, Martini D, Bagnoli P, Simonetti E, et al. (2007) Changes in neuronal response to ischemia in retinas with genetic alterations of somatostatin receptor expression. Eur J Neurosci 25: 1447-1459.

439. Hunt CR, Dix DJ, Sharma GG, Pandita RK, Gupta A, et al. (2004) Genomic instability and enhanced radiosensitivity in Hsp70.1- and Hsp70.3-deficient mice. Mol Cell Biol 24: 899-911.

440. Masciarelli S, Horner K, Liu C, Park SH, Hinckley M, et al. (2004) Cyclic nucleotide phosphodiesterase 3A-deficient mice as a model of female infertility. J Clin Invest 114: 196-205.

441. Casademunt E, Carter BD, Benzel I, Frade JM, Dechant G, et al. (1999) The zinc finger protein NRIF interacts with the neurotrophin receptor p75(NTR) and participates in programmed cell death. Embo J 18: 6050-6061.

442. Kajiwara K, J OW, Sakurai T, Yamashita S, Tanaka M, et al. (2001) Sez4 gene encoding an elongation subunit of DNA polymerase zeta is required for normal embryogenesis. Genes Cells 6: 99-106.

443. Brower JV, Rodic N, Seki T, Jorgensen M, Fliess N, et al. (2007) Evolutionarily conserved mammalian adenine nucleotide translocase 4 is essential for spermatogenesis. J Biol Chem 282: 29658-29666.

444. Xu J, Song P, Nakamura S, Miller M, Barone S, et al. (2009) Deletion of the chloride transporter slc26a7 causes distal renal tubular acidosis and impairs gastric acid secretion. J Biol Chem 284: 29470-29479.

445. Hudson JW, Kozarova A, Cheung P, Macmillan JC, Swallow CJ, et al. (2001) Late mitotic failure in mice lacking Sak, a polo-like kinase. Curr Biol 11: 441-446.

446. Molne K, Brabrand G (1968) Spontaneous adrenocortical lipid depletion in mice. Relationship to general growth, degeneration of adrenal X zone, and maturation of seminiferous eptthelium. Acta Pathol Microbiol Scand 72: 478-490.

447. Itoh T, Cado D, Kamide R, Linn S (2004) DDB2 gene disruption leads to skin tumors and resistance to apoptosis after exposure to ultraviolet light but not a chemical carcinogen. Proc Natl Acad Sci U S A 101: 2052-2057.

448. Rodig SJ, Meraz MA, White JM, Lampe PA, Riley JK, et al. (1998) Disruption of the Jak1 gene demonstrates obligatory and nonredundant roles of the Jaks in cytokine-induced biologic responses. Cell 93: 373-383.

449. Ciccone DN, Su H, Hevi S, Gay F, Lei H, et al. (2009) KDM1B is a histone H3K4 demethylase required to establish maternal genomic imprints. Nature 461: 415-418.

450. Yoshioka J, Imahashi K, Gabel SA, Chutkow WA, Burds AA, et al. (2007) Targeted deletion of thioredoxin-interacting protein regulates cardiac dysfunction in response to pressure overload. Circ Res 101: 1328-1338.

451. Gautam M, Noakes PG, Mudd J, Nichol M, Chu GC, et al. (1995) Failure of postsynaptic specialization to develop at neuromuscular junctions of rapsyn-deficient mice. Nature 377: 232-236.

452. Zheng B, Tang T, Tang N, Kudlicka K, Ohtsubo K, et al. (2006) Essential role of RGS-PX1/sorting nexin 13 in mouse development and regulation of endocytosis dynamics. Proc Natl Acad Sci U S A 103: 16776-16781.

453. Li G, Mongillo M, Chin KT, Harding H, Ron D, et al. (2009) Role of ERO1-alpha-mediated stimulation of inositol 1,4,5-triphosphate receptor activity in endoplasmic reticulum stress-induced apoptosis. J Cell Biol 186: 783-792.

454. Shah GN, Ulmasov B, Waheed A, Becker T, Makani S, et al. (2005) Carbonic anhydrase IV and XIV knockout mice: roles of the respective carbonic anhydrases in buffering the extracellular space in brain. Proc Natl Acad Sci U S A 102: 16771-16776.

455. Bensamoun SF, Hawse JR, Subramaniam M, Ilharreborde B, Bassillais A, et al. (2006) TGFbeta inducible early gene-1 knockout mice display defects in bone strength and microarchitecture. Bone 39: 1244-1251.

456. Qiao J, Uzzo R, Obara-Ishihara T, Degenstein L, Fuchs E, et al. (1999) FGF-7 modulates ureteric bud growth and nephron number in the developing kidney. Development 126: 547-554.

457. Lykke-Andersen K, Schaefer L, Menon S, Deng XW, Miller JB, et al. (2003) Disruption of the COP9 signalosome Csn2 subunit in mice causes deficient cell proliferation, accumulation of p53 and cyclin E, and early embryonic death. Mol Cell Biol 23: 6790-6797.

458. Schwab KR, Patterson LT, Hartman HA, Song N, Lang RA, et al. (2007) Pygo1 and Pygo2 roles in Wnt signaling in mammalian kidney development. BMC Biol 5: 15.

459. Li SW, Takanosu M, Arita M, Bao Y, Ren ZX, et al. (2001) Targeted disruption of Col11a2 produces a mild cartilage phenotype in transgenic mice: comparison with the human disorder otospondylomegaepiphyseal dysplasia (OSMED). Dev Dyn 222: 141-152.

460. Pirog-Garcia KA, Meadows RS, Knowles L, Heinegard D, Thornton DJ, et al. (2007) Reduced cell proliferation and increased apoptosis are significant pathological mechanisms in a murine model of mild pseudoachondroplasia resulting from a mutation in the C-terminal domain of COMP. Hum Mol Genet 16: 2072-2088.

461. Moza M, Mologni L, Trokovic R, Faulkner G, Partanen J, et al. (2007) Targeted deletion of the muscular dystrophy gene myotilin does not perturb muscle structure or function in mice. Mol Cell Biol 27: 244-252.

462. Czipri M, Otto JM, Cs-Szabo G, Kamath RV, Vermes C, et al. (2003) Genetic rescue of chondrodysplasia and the perinatal lethal effect of cartilage link protein deficiency. J Biol Chem 278: 39214-39223.

463. Sugimoto Y, Yamasaki A, Segi E, Tsuboi K, Aze Y, et al. (1997) Failure of parturition in mice lacking the prostaglandin F receptor. Science 277: 681-683.

464. Dodge JE, Kang YK, Beppu H, Lei H, Li E (2004) Histone H3-K9 methyltransferase ESET is essential for early development. Mol Cell Biol 24: 2478-2486.

465. Saegusa C, Tanaka T, Tani S, Itohara S, Mikoshiba K, et al. (2006) Decreased basal mucus secretion by Slp2-a-deficient gastric surface mucous cells. Genes Cells 11: 623-631.

466. Filicori M, Bolelli G, Franceschetti F, Lafisca S (1979) The ultradian pulsatile release of gonadotropins in normal female subjects. Acta Eur Fertil 10: 29-33.

467. Sekita Y, Wagatsuma H, Nakamura K, Ono R, Kagami M, et al. (2008) Role of retrotransposon-derived imprinted gene, Rtl1, in the feto-maternal interface of mouse placenta. Nat Genet 40: 243-248.

468. Lelliott CJ, Medina-Gomez G, Petrovic N, Kis A, Feldmann HM, et al. (2006) Ablation of PGC-1beta results in defective mitochondrial activity, thermogenesis, hepatic function, and cardiac performance. PLoS Biol 4: e369.

469. Okada A, Charron F, Morin S, Shin DS, Wong K, et al. (2006) Boc is a receptor for sonic hedgehog in the guidance of commissural axons. Nature 444: 369-373.

470. Inoue K, Wen R, Rehg JE, Adachi M, Cleveland JL, et al. (2000) Disruption of the ARF transcriptional activator DMP1 facilitates cell immortalization, Ras transformation, and tumorigenesis. Genes Dev 14: 1797-1809.

471. Shin K, Nigrovic PA, Crish J, Boilard E, McNeil HP, et al. (2009) Mast cells contribute to autoimmune inflammatory arthritis via their tryptase/heparin complexes. J Immunol 182: 647-656.

472. Li M, Hener P, Zhang Z, Ganti KP, Metzger D, et al. (2009) Induction of thymic stromal lymphopoietin expression in keratinocytes is necessary for generating an atopic dermatitis upon application of the active vitamin D3 analogue MC903 on mouse skin. J Invest Dermatol 129: 498-502.

473. Lee NK, Sowa H, Hinoi E, Ferron M, Ahn JD, et al. (2007) Endocrine regulation of energy metabolism by the skeleton. Cell 130: 456-469.

474. Cheng CW, Chow RL, Lebel M, Sakuma R, Cheung HO, et al. (2005) The Iroquois homeobox gene, Irx5, is required for retinal cone bipolar cell development. Dev Biol 287: 48-60.

475. Maccarana M, Kalamajski S, Kongsgaard M, Magnusson SP, Oldberg A, et al. (2009) Dermatan sulfate epimerase 1-deficient mice have reduced content and changed distribution of iduronic acids in dermatan sulfate and an altered collagen structure in skin. Mol Cell Biol 29: 5517-5528.

476. Schmahl J, Raymond CS, Soriano P (2007) PDGF signaling specificity is mediated through multiple immediate early genes. Nat Genet 39: 52-60.

477. Whitehead KJ, Plummer NW, Adams JA, Marchuk DA, Li DY (2004) Ccm1 is required for arterial morphogenesis: implications for the etiology of human cavernous malformations. Development 131: 1437-1448.

478. Lopez-Rodriguez C, Antos CL, Shelton JM, Richardson JA, Lin F, et al. (2004) Loss of NFAT5 results in renal atrophy and lack of tonicity-responsive gene expression. Proc Natl Acad Sci U S A 101: 2392-2397.

479. Kim S, Lehtinen MK, Sessa A, Zappaterra MW, Cho SH, et al. (2010) The apical complex couples cell fate and cell survival to cerebral cortical development. Neuron 66: 69-84.

480. Anton IM, de la Fuente MA, Sims TN, Freeman S, Ramesh N, et al. (2002) WIP deficiency reveals a differential role for WIP and the actin cytoskeleton in T and B cell activation. Immunity 16: 193-204.

481. Calderon A, Derr A, Stagner BB, Johnson KR, Martin G, et al. (2006) Cochlear developmental defect and background-dependent hearing thresholds in the Jackson circler (jc) mutant mouse. Hear Res 221: 44-58.

482. Charbonneau NL, Carlson EJ, Tufa S, Sengle G, Manalo EC, et al. (2010) In vivo studies of mutant fibrillin-1 microfibrils. J Biol Chem 285: 24943-24955.

483. Barash IA, Bang ML, Mathew L, Greaser ML, Chen J, et al. (2007) Structural and regulatory roles of muscle ankyrin repeat protein family in skeletal muscle. Am J Physiol Cell Physiol 293: C218-227.

484. Steele-Perkins G, Fang W, Yang XH, Van Gele M, Carling T, et al. (2001) Tumor formation and inactivation of RIZ1, an Rb-binding member of a nuclear protein-methyltransferase superfamily. Genes Dev 15: 2250-2262.

485. Gregoire C, Simova S, Wang Y, Sansoni A, Richelme S, et al. (2007) Deletion of the LIME adaptor protein minimally affects T and B cell development and function. Eur J Immunol 37: 3259-3269.

486. Tateishi K, Okada Y, Kallin EM, Zhang Y (2009) Role of Jhdm2a in regulating metabolic gene expression and obesity resistance. Nature 458: 757-761.

487. Anthony TG, McDaniel BJ, Byerley RL, McGrath BC, Cavener DR, et al. (2004) Preservation of liver protein synthesis during dietary leucine deprivation occurs at the expense of skeletal muscle mass in mice deleted for eIF2 kinase GCN2. J Biol Chem 279: 36553-36561.

488. Takeda K, Aguila HL, Parikh NS, Li X, Lamothe K, et al. (2008) Regulation of adult erythropoiesis by prolyl hydroxylase domain proteins. Blood 111: 3229-3235.

489. Inagaki M, Irie K, Ishizaki H, Tanaka-Okamoto M, Morimoto K, et al. (2005) Roles of cell-adhesion molecules nectin 1 and nectin 3 in ciliary body development. Development 132: 1525-1537.

490. Chichinadze N (1975) [Nina Aleksandrovna Dzhavakhishvili (on her 60th birthday)]. Arkh Anat Gistol Embriol 69: 119-125.

491. Kohoutek J, Li Q, Blazek D, Luo Z, Jiang H, et al. (2009) Cyclin T2 is essential for mouse embryogenesis. Mol Cell Biol 29: 3280-3285.

492. Keller C, Hansen MS, Coffin CM, Capecchi MR (2004) Pax3:Fkhr interferes with embryonic Pax3 and Pax7 function: implications for alveolar rhabdomyosarcoma cell of origin. Genes Dev 18: 2608-2613.

493. Lee S, Lee DK, Dou Y, Lee J, Lee B, et al. (2006) Coactivator as a target gene specificity determinant for histone H3 lysine 4 methyltransferases. Proc Natl Acad Sci U S A 103: 15392-15397.

494. Godinho SI, Maywood ES, Shaw L, Tucci V, Barnard AR, et al. (2007) The after-hours mutant reveals a role for Fbxl3 in determining mammalian circadian period. Science 316: 897-900.

495. Masson J, Darmon M, Conjard A, Chuhma N, Ropert N, et al. (2006) Mice lacking brain/kidney phosphate-activated glutaminase have impaired glutamatergic synaptic transmission, altered breathing, disorganized goal-directed behavior and die shortly after birth. J Neurosci 26: 4660-4671.

496. Friese MB, Blagden CS, Burden SJ (2007) Synaptic differentiation is defective in mice lacking acetylcholine receptor beta-subunit tyrosine phosphorylation. Development 134: 4167-4176.

497. Lebel M, Agarwal P, Cheng CW, Kabir MG, Chan TY, et al. (2003) The Iroquois homeobox gene Irx2 is not essential for normal development of the heart and midbrain-hindbrain boundary in mice. Mol Cell Biol 23: 8216-8225.

498. Kieslinger M, Folberth S, Dobreva G, Dorn T, Croci L, et al. (2005) EBF2 regulates osteoblast-dependent differentiation of osteoclasts. Dev Cell 9: 757-767.

499. Schuster-Gossler K, Simon-Chazottes D, Guenet JL, Zachgo J, Gossler A (1996) Gtl2lacZ, an insertional mutation on mouse chromosome 12 with parental origin-dependent phenotype. Mamm Genome 7: 20-24.

500. Andrews W, Barber M, Hernadez-Miranda LR, Xian J, Rakic S, et al. (2008) The role of Slit-Robo signaling in the generation, migration and morphological differentiation of cortical interneurons. Dev Biol 313: 648-658.

501. Ansel KM, Ngo VN, Hyman PL, Luther SA, Forster R, et al. (2000) A chemokine-driven positive feedback loop organizes lymphoid follicles. Nature 406: 309-314.
